# Substrate-restricted methanogenesis and limited volatile organic compound degradation in highly diverse and heterogeneous municipal landfill microbial communities

**DOI:** 10.1038/s43705-022-00141-4

**Published:** 2022-07-13

**Authors:** Alexandra H. Sauk, Laura A. Hug

**Affiliations:** Department of Biology, University of Waterloo, 200 University Ave, Waterloo, ON N2L 3G1 Canada

**Keywords:** Environmental microbiology, Metagenomics, Applied microbiology

## Abstract

Microbial communities in landfills transform waste and generate methane in an environment unique from other built and natural environments. Landfill microbial diversity has predominantly been observed at the phylum level, without examining the extent of shared organismal diversity across space or time. We used 16S rRNA gene amplicon and shotgun metagenomic sequencing to examine the taxonomic and functional diversity of the microbial communities inhabiting a Southern Ontario landfill. The microbial capacity for volatile organic compound degradation in leachate and groundwater samples was correlated with geochemical conditions. Across the landfill, 25 bacterial and archaeal phyla were present at >1% relative abundance within at least one landfill sample, with *Patescibacteria*, *Bacteroidota*, *Firmicutes*, and *Proteobacteria* dominating. Methanogens were neither numerous nor particularly abundant, and were predominantly constrained to either acetoclastic or methylotrophic methanogenesis. The landfill microbial community was highly heterogeneous, with 90.7% of organisms present at only one or two sites within this interconnected system. Based on diversity measures, the landfill is a microbial system undergoing a constant state of disturbance and change, driving the extreme heterogeneity observed. Significant differences in geochemistry occurred across the leachate and groundwater wells sampled, with calcium, iron, magnesium, boron, meta and para xylenes, ortho xylenes, and ethylbenzene concentrations contributing most strongly to observed site differences. Predicted microbial degradation capacities indicated a heterogeneous community response to contaminants, including identification of novel proteins implicated in anaerobic degradation of key volatile organic compounds.

## Introduction

The environmental impact and monetary cost of municipal solid waste (MSW) storage and management are growing concerns for municipalities and countries around the world. MSW generation has increased exponentially with rising populations, increased development, and urbanization [1, 2]. By 2025, the global annual production of waste will reach an estimated 2.2 billion tons, and is not predicted to hit a maximum in this century, reaching 4 billion tons annually in 2100 [1, 3]. This is an unsustainable rate of increase.

Landfills are the most common end point for MSW in many countries, including Canada, the United States, and China. Landfills are the third largest contributor to anthropogenic methane emissions, contributing 11% of annual global methane emissions and making them a focus area for mitigating climate change [3–5]. Waste degradation in landfills is controlled by the microbial communities within the landfill and the built characteristics of the landfill, such as leachate collection systems and cover soils [4, 6]. The first three steps of the general waste decomposition process are reliant on bacteria: hydrolysis; acidogenesis, including both fermentation and beta oxidation; and acetogenesis [6]. The last step, methanogenesis, is dependent on methanogenic archaea [6]. Landfill deposits are diverse, both chemically and physically, which can inhibit or prevent these microbial degradation processes [7]. Volatile organic compounds (VOCs) like chlorinated ethenes and hydrocarbons commonly contaminate landfill waste, due to improper dumping or as legacy waste deposited prior to regulations on disposal. Microbially mediated volatile organic compound (VOC) degradation in landfills impacts landfill emissions as well as contaminant fate when VOCs are leaked into the surrounding groundwater and terrestrial environment. Understanding the ecology and diversity of the bacterial and archaeal community structure in landfills will strengthen our understanding of the MSW decomposition process, allowing for better control of methane production and more efficient waste management and contaminant mitigation strategies.

Despite recent interest in landfill microbial diversity [7–12], much is still unknown about landfill-associated microbial communities and their distributed functions. Most early research focused on specific aspects of waste degradation in landfills and the microbes responsible, with particular interest in methane cycling [13, 14] and cellulose degradation [15, 16] (See [17] and references within for a review of direct landfill surveys and bioreactor-based examinations). With the advent of high-throughput sequencing techniques like 16S rRNA gene amplicon sequencing, overall landfill microbial diversity and community composition have also been examined [7, 11]. The most abundant phyla consistently identified from landfills include the *Firmicutes, Bacteroidota, Campylobacterota* (formerly *Epsilonproteobacteria* [18]) and *Proteobacteria* [6, 7, 10, 11]. Methanogenic archaea in landfills are typically predominantly hydrogenotrophs, with *Methanobacteriales* and *Methanomicrobiales* frequently at high abundance earlier in the landfill lifecycle [19]. Methanogens with the capacity for acetoclastic, hydrogenotrophic, or multiple methanogenic pathways are also common in landfills [19–21]. A number of rare and/or unclassified microorganisms have also been found in recent landfill studies, with some at high abundance [6, 7]. These studies allow for functions to be inferred for microorganisms with well-characterized relatives, but the 16S rRNA gene cannot be used to infer functions for unclassified microorganisms that are uncultivated or newly described [6, 11]. There is a recognized need for metagenome-level community profiling from landfills [20].

Several environmental or geochemical factors that influence microbial community composition and heterogeneity have been identified. The age of landfilled waste has been correlated with microbial community composition characteristics [6, 7, 11, 22, 23]. Community composition was also correlated with moisture [10, 11] and ammonium concentration [6, 10]. Other chemicals that showed a link to microbial community composition included barium, chloride, sulfate, and copper [7, 10]. Other chemical factors seem to affect microbial communities in a site-specific manner, and their effects will depend on the types of waste deposited and other geochemical conditions at each site of interest [7, 22, 24, 25].

**Fig. 1:**
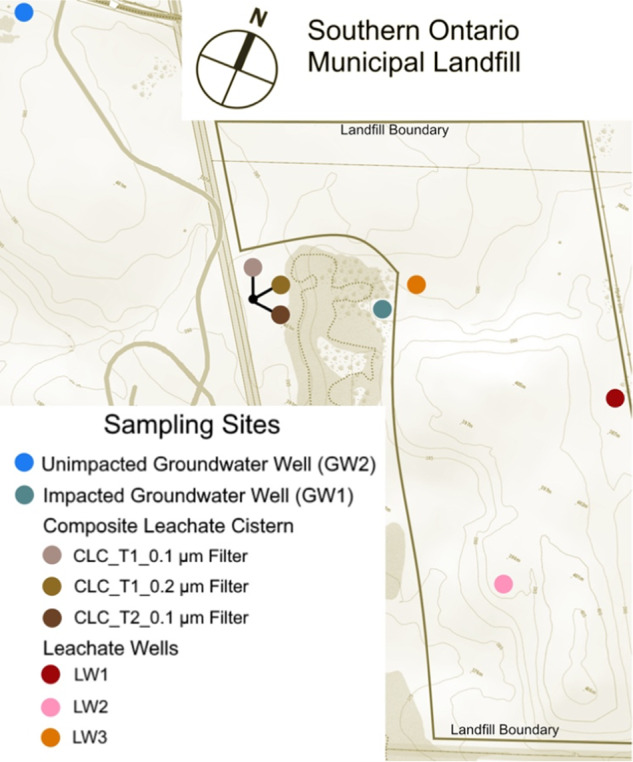
Map of landfill sampling locations at the Southern Ontario landfill. Two groundwater wells accessing the adjacent aquifer were sampled. The three samples from the leachate collecting cistern were all sampled from the same cistern at two time points. Two filter sizes were used for collecting microbial biomass on July 14, 2016 (CLC_T1_0.1 µm filter and CLC_T1_0.2 µm filter) and one filter size was used on July 20, 2016 (CLC_T2_0.1 µm filter). The three leachate wells are located within the active landfill, and leachate from these wells is pumped to the leachate collecting cistern. The catchment area for LW3 has an active leak infiltrating into the groundwater near GW1. GW1, the impacted groundwater well, shows geochemical evidence of leachate infiltration into the aquifer, where GW2 is upstream and shows no leachate chemical signature in the groundwater. The topographic map was modified from maps provided by the Ontario Ministry of Natural Resources and Forestry.

The study site for this research was a municipal waste landfill in southern Ontario, Canada that opened in 1972. This landfill is a conventional sanitary landfill with onsite waste sorting, compacting, and daily soil covers. The landfill is well-instrumented, with over 100 leachate wells (LW) across the site as well as three composite leachate cisterns (CLC). The leachate wells are routinely sampled by regional waste management staff to determine the chemical composition of the leachate. There are also groundwater wells (GW) bordering the landfill for monitoring the conditions of the adjacent aquifer and any leachate leaks (e.g., there is on-going leachate infiltration from the area near LW3 into the aquifer near GW1, Fig. 1). In order to understand waste degradation processes, methane emission profiles, and the transformation and movement of contaminants within the site, it is important to understand microbial community heterogeneity as well as biodegradation capacity for contaminants of concern across the landfill. Here, we combine metagenomic and 16S rRNA gene amplicon sequencing techniques to characterize the distribution, heterogeneity, and diversity of the microbial communities in a Southern Ontario municipal landfill. We additionally investigate how the predicted microbial degradative capacities connect with geochemical conditions across the site.

## Materials and methods

### Sample collection

In the initial sampling event on July 14, 2016, a sample was collected from the composite leachate cistern by filtering the leachate through a 0.2 μm poly-ethersulfone filter followed by a 0.1 μm poly-ethersulfone filter in series (CLC_T1_0.2 and CLC_T1_0.1, respectively). Both filters were kept for DNA extractions. On July 20, 2016, a larger-scale sampling was conducted, sampling the composite leachate cistern (CLC_T2), three leachate wells (LW1, LW2, LW3), and two groundwater wells (GW1, GW2). Leachate and groundwater samples were collected by pumping liquid through a filter apparatus with a 3 μm glass fiber pre-filter in series with a 0.1 μm poly-ethersulfone filter until filters clogged. The pre-filter was discarded while the 0.1 μm filters with microbial biomass were kept. All filters were frozen on dry ice in the field and transferred to a −80 °C freezer until processed. DNA was extracted from the biomass using the Powersoil DNA extraction kit (MoBio) following the manufacturer’s instructions with one modification: filters were sliced into pieces and added to the bead tube in place of a soil sample.

Relevant measurements for volatile and non-volatile compound concentrations at the leachate and groundwater wells are conducted each year in October and April by a contracted consulting company. For 2016, the average values for these two sampling points were used to estimate compound concentrations in July, the time of microbial biomass sampling (Supplementary Table 1). The impacted groundwater well, GW1, did not have current non-volatile concentration measurements available. For this well, measurements from 2011 were included for comparison purposes only (Supplementary Table 1). No geochemical measurements were available for the composite leachate cistern.

### Sequencing

All eight samples were sent to the US Department of Energy’s Joint Genome Institute (JGI) for 16S rRNA gene amplicon sequencing: LW1, LW2, LW3, CLC_T1 0.1 μm and 0.2 μm filters, CLC_T2, GW1, and GW2. The JGI amplified the V4 region of the 16S rRNA gene using the forward primer 515 F (Parada) (5’-GTGYCAGCMGCCGCGGTAA-3’) and the reverse primer 806 R (Apprill) (5’- GGACTACNVGGGTWTCTAA -3’) using in-house protocols (as described here, but with the above listed primers: https://jgi.doe.gov/wp-content/uploads/2016/06/DOE-JGI-iTagger-methods.pdf). Amplicons were sequenced on the MiSeq platform (Illumina) with extraction negative controls, amplification negative controls, and positive controls, and reads were quality control checked using the iTagger pipeline [26].

Six DNA samples were sent to the JGI for metagenomic sequencing, assembly, and annotation: LW1, LW2, LW3, CLC_T1 (0.2 μm filter), CLC_T2, and GW1. The CLC_T1 and GW2 0.1 μm filters resulted in insufficient DNA and were not shot-gun sequenced. Metagenomes were sequenced as paired-end 150 bp reads using the HiSeq platform (Illumina) and annotated using the DOE-JGI Metagenome Annotation Pipeline (MAP v.4) [27].

### Phylogenetic trees

All assembled and annotated 16S rRNA genes in the landfill metagenomes were downloaded from the JGI Integrated Microbial Genomics (IMG) server (IMG Genome IDs: CLC_T1: 3300014203, CLC_T2: 3300014206, LW1: 3300014204, LW2: 3300015214, LW3: 3300014205, GW1: 3300014208). Genes were sorted by length in Geneious 11.0.5 (https://www.geneious.com) and curated to a minimum length of 600 bp. The landfill metagenome-derived 16S rRNA genes as well as a reference set of 16S rRNA genes from known organisms were aligned with the SILVA SINA algorithm [28]. Unaligned bases at the ends of the genes were removed and sequences below 70% identity to a reference sequence were automatically removed from the dataset by SINA. To curate the SINA alignment, columns containing 97% or more gaps were removed, a region of poor alignment was manually trimmed from the 3’ end, and sequences falling below 600 bp post-trimming were removed. A phylogenetic tree was inferred using FastTree in Geneious to check for poorly aligned or divergent sequences. In this processing, 195 sequences were removed that did not meet quality standards. The final 16S rRNA gene alignment included 1903 reference sequences and 2306 sequences from the metagenome samples, and had 1521 positions. A high-quality phylogenetic tree was inferred from the curated final alignment using RAxML-HPC2 8.2.12 [29] on CIPRES [30] under model GTRCAT, with 100 alternative bootstrap iterations run from 100 starting trees. The full tree topology is presented in Supplementary File 1.

All amino acid sequences for 16 syntenic, universally-present, single copy ribosomal protein genes (RpL2, L3, L4, L5, L6, L14, L15, L16, L18, L22, L24 and RpS3, S8, S10, S17, S19) for the landfill metagenomes were downloaded from the JGI IMG server using annotation keyword-based identification [31]. Ribosomal protein datasets were screened for the Archaeal/Eukaryotic type, which were removed, as were short (<45 aa) sequences. Each individual protein set was aligned with a reference set of genes [32] using MAFFT 7.402 [33] on CIPRES. Alignment columns containing ≥95% gaps were removed using Geneious. IMG-derived sequence names were trimmed to 8 digits after the metagenome code (e.g., Ga0172377_100004578 → Ga0172377_10000457) to remove gene-specific identifiers and allow for concatenation by scaffold name. The protein gene alignments were concatenated in numeric order (L2 → L24, followed by S3 → S19). Concatenated sequences that contained less than 50% of the total expected number of aligned amino acids were removed. The final alignment was 3452 columns long and contained 2914 reference organisms and 1265 scaffolds from the metagenome samples. A phylogenetic tree was inferred using RAxML-HPC Blackbox on CIPRES using the following parameters: sequence type - protein; protein substitution matrix – LG; and estimate proportion of invariable sites (GTRGAMMA + I) – yes [29, 30]. The full tree topology is presented in Supplementary File 1.

### 16S rRNA amplicon sequence analyses

The demultiplexed and barcode-trimmed 16S rRNA gene amplicons from the JGI were analyzed using QIIME2 [34]. Forward and reverse reads were separated using khmer [35]. Primers were trimmed from the forward and reverse reads using cutadapt in QIIME2 [36]. The forward reads were truncated at 231 base pairs and the reverse reads at 230 base pairs based on the quality score visualization produced by QIIME2 in the demux summary step. Reads were denoised using paired denoising in DADA2 within the QIIME2 platform which also merges the reads [37]. Sequence variants were determined using DADA2 and summarized using feature-table summarize in QIIME2. Taxonomic assignment of the 16S rRNA gene amplicons was based on a phylogenetic tree produced by QIIME2 in which the taxonomy classifier was trained with the SILVA 99% taxonomy classification for the 16S rRNA gene from the April 2018 SILVA 132 release [38]. Phylum names were updated as per the GTDB database taxonomy changes by Parks et al. (2018) for diversity comparisons.

### Metagenomic binning

All scaffolds >2500 bp were included in the binning process. The binning algorithm CONCOCT [39] was used in Anvi’o [40] to automatically cluster each metagenome’s scaffolds using a combination of scaffold tetranucleotide frequencies and read-mapped coverage data from all six metagenomes. Gene annotations were imported from the JGI annotations, overriding the automated annotation pipeline in Anvi’o. The bins were manually refined for the six metagenomes using Anvi’o, focusing on completion and quality metrics to guide bin refinements. High quality bins were considered those with greater than 70% completion and less than 10% redundancy. Read mapping was used to calculate coverage for McrA and VOC-degradation gene-encoding scaffolds, restricted to scaffolds 2.5 kb or longer.

### Diversity analyses

Diversity analyses on the 16S rRNA gene amplicon sequence variants (ASVs) identified by QIIME2 [34] included the alpha diversity metrics Faith’s phylogenetic diversity [41] and Pielou’s evenness [42], calculated based on four sample types: impacted groundwater well, unimpacted groundwater well, leachate well, and composite leachate cistern. A Shannon diversity index analysis with rarified sequence depth of 53,518 was conducted using QIIME2 and visualized using phyloseq [43] in R. A Chao1 statistic was not calculated, as data processing with QIIME2 and DADA2 removes all singleton ASVs, which the Chao1 statistic requires. For beta diversity measures, full ASV and taxonomy tables were input to unweighted and weighted UniFrac distances principle coordinate analyses, calculated using phyloseq and visualized in R for all samples. The prevalence across samples of ASVs with a count of 2 or more and belonging to phyla with relative abundance greater than 1% or present in multiple sites was determined using phyloseq and visualized in R. The phyla with ASVs present in five or more sites was visualized using ggplot2 in R.

A principal component analysis (PCA) was conducted using vegan [44] in R for all 16S rRNA gene amplicon ASVs and the16S rRNA gene amplicon ASVs present at five or more sites. The ASV count data was Hellinger transformed to reduce the weight of ASVs with low counts and zeros. The leachate wells and the two groundwater well samples were included in the PCA to allow for comparison with environmental parameters, which are available for those sites. Environmental data was not available for the composite leachate cistern site and so CLC samples were included in the analysis only for comparison with the other samples.

Metagenome-derived sequences were classified at the phylum level based on their placement within reference clades on the 16S rRNA and concatenated ribosomal protein phylogenetic trees. Metagenome sequences placing outside of or between phyla were assigned to either “Unclassified Archaea” or “Unclassified Bacteria” as appropriate. Phylum names were updated from the NCBI taxonomy to conform to the GTDB database taxonomy by Parks et al. (2018). Bins were identified at the phylum level using the scaffold assignments from the 16S rRNA gene and concatenated ribosomal protein phylogenetic trees. Bin abundances were determined using the average fold coverage data for all scaffolds in the bin. Phylum abundance per sample was calculated by summing the average fold coverage data for each scaffold on the tree assigned to the phylum, where the scaffold acts as a proxy for the underlying microbial population. Microbial diversity comparisons were visualized using stacked bar plots produced using ggplot2 in R [45].

### Chemical data analyses

Chemical measurements provided by the Southern Ontario landfill 2016 annual report were used to determine variance of non-volatile and volatile compounds over time for the three leachate wells and the unimpacted groundwater well. GW1 only has non-volatile compound measurements for one time point in 2011 and so variance could not be calculated. Non-detects, where a compound, if present, is below the detection limit, were treated as zeros. The measurements were log transformed and visualized in a heatmap using heatmap3 [46] in R. Metal and volatile compounds detected in a majority of samples were used for further analysis. The measurements from April and October of 2016 were averaged to estimate the concentrations at the time of microbial biomass sampling.

PCA for the metals and volatile compounds were conducted using vegan [44] in R. The metal and volatile compound concentrations were square root transformed to reduce the range of the values as different compounds differed in concentration by orders of magnitude (Supplementary Table 2). Data for leachate wells and the two groundwater well samples were included for the volatile analysis, but GW1 was excluded from the non-volatile compound analysis as no data were available for that site in 2016. A PCA was also conducted using vegan in R for the other geochemical parameters measured at the sites that are not characterized as non-volatile or volatile compounds (e.g., total dissolved solids (TDS)).

### Methanogenesis and VOC degradation capacity

KEGG KO numbers for *mcrA* and key anaerobic degradation enzymes for the dominant VOCs detected at the Southern Ontario landfill were searched from the annotations for all six metagenomes. Reductive dehalogenases’ catalytic subunits (RdhA), responsible for chlorinated ethene, ethane, and benzene degradation, were annotated by pfam13486 instead of a KO. AbcA, the carboxylase associated with anaerobic benzene degradation [47] does not belong to a KO, and instead was searched using manual BLASTp [48] using three characterized enzymes as queries (ADJ94002.1, WP_011237597, GI10697123) with an initial threshold of e < 1e^−30^.

Annotated proteins were screened through a combination of phylogenetic placement and/or in-depth annotation using BlastKoala [49] and NCBI’s conserved domains feature [50]. All hits were required to have a minimum length of 250 amino acids or a length at least 50% that of the reference sequences if that minimum was below 250 aa (i.e., >200 aa for RdhA, >204 aa for AbcA). Outgroup proteins were derived from literature for each protein of interest (see Table 1 for outgroup protein names and references).

**Table 1:** Detection of anaerobic volatile organic compound degradation proteins.

Cells for LW1,2, and 3 and GW1 are shaded based on concentration of the relevant VOC at that site, with absence in white and gradation of grey (light = 1–10 µg/L, medium = 10–50 µg/L, dark = >50 µg/L). Geochemical information was not available for the CLC site. Dioxane degradation proteins are both obligately aerobic – no anaerobic degradation pathway has been identified to date. Supplementary Data File 2 includes aerobic degradation options as well as accessions for all curated proteins in this table. *PFL* pyruvate formate lyase.

Proteins were aligned to reference and outgroup sequences using Muscle SRC v. 3.8.1551, columns containing >97% gaps were trimmed using Geneious Prime v. 2021.2.2, and phylogenetic trees inferred using FastTree2 [51]. Metagenome-derived protein sequences that passed the length threshold, affiliated with the correct clade in phylogenetic trees, and had consistent annotations to the functions of interest from BlastKoala or the Conserved Domains Database were kept (Table 1). Connection to high quality MAGs was determined based on scaffold IDs. MAG taxonomy was based on GTDB-tk [52]. Relative distribution compared to VOC concentration at each sampling site was assessed (Table 1).

RdhA and AbcA were selected for deeper examination. Using the CIPRES phylogenomics webserver [30], alignments were tested for the best model of evolution under ModelTest-NG v.0.1.5 [53] and inference of maximum likelihood trees conducted using RAxML-HPC v. 8.2.12 [54] under the best-fit model (LG + G + I for AbcA; VT + G + I for RdhA), and with automatic bootstopping to identify the appropriate number of bootstrap resamplings. For reductive dehalogenases, a reference set from [55] was used to confirm reductive dehalogenase protein annotations, phylogenetic affiliation, and potential substrate specificities. For AbcA, the three proteins associated with tigrfam TIGR02723 were included as positive controls, with reference sequences for UbiD carboxylase (pfam01977) included as an outgroup [56].

## Results

### Phylum level diversity

Solid waste sampling of the landfill was not possible, as disruption to the landfill cover was not permitted. Instead, we sampled leachate from monitoring wells to gain insight to the planktonic microbial community circulating within the landfill. Samples were collected from three leachate wells (LW1, LW2, and LW3), two samples from a composite leachate cistern at time points separated by one week (CLC_T1 and CLC_T2), and samples from two groundwater wells (GW1 and GW2) adjacent to the landfill (Fig. 1). 16S rRNA amplicons and shotgun metagenomes were generated and processed as discussed in the methods.

**Fig. 2:**
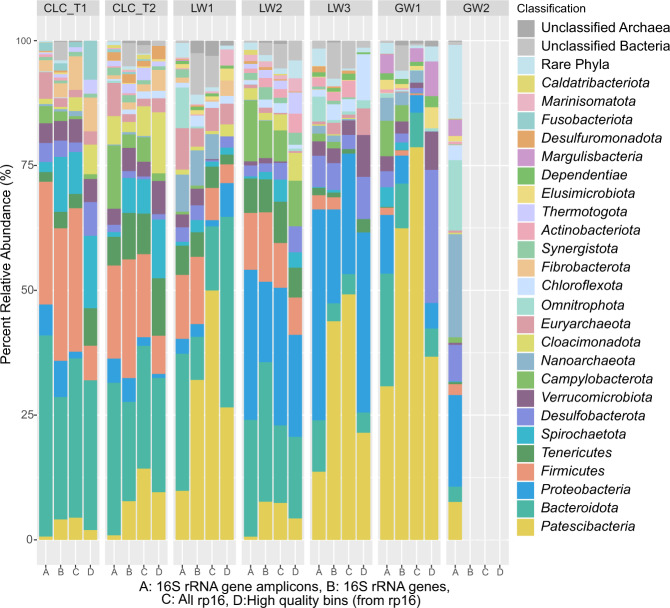
Relative abundances for phyla present at greater than 1% abundance in at least one sample. **A**) 16 S rRNA gene amplicons; **B**) 16 S rRNA genes derived from the assembled metagenomes; **C**) concatenation of 16 syntenic ribosomal proteins derived from the assembled metagenomes, with scaffold coverage as a proxy for abundance; and **D**) high quality bins (containing the 16 concatenated ribosomal proteins, and with abundances calculated from the ribosomal protein-encoding scaffolds’ coverages). The Composite Leachate Cistern at timepoint 1 (CLC_T1) is represented by the 0.2 µm filter’s sequence information, as the 0.1 µm filter showed highly similar results. Site GW2, the unimpacted groundwater well, did not yield sufficient biomass for metagenomic sequencing, thus only 16 S rRNA gene amplicons are reported here.

The 16S rRNA amplicon sequences were taxonomically classified and relative abundances were determined using QIIME2 [34]. From the 16S rRNA gene analysis, 8030 amplicon sequence variants (ASVs) were identified across the sampled sites with an average of 1147 ASVs per site. In tandem, metagenomic scaffolds were identified to the phylum level via placement on phylogenetic trees inferred based on the 16S rRNA gene and a suite of sixteen concatenated ribosomal proteins. Phylogenetic trees included 1265 and 2306 metagenome-derived sequences for the ribosomal protein and 16S rRNA gene trees, respectively. The total number of medium or higher quality metagenome assembled genomes (MAGs, >70% completeness, <10% contamination) resolved from the six metagenomes was 503. Taxonomy information was combined with scaffold coverage data to determine the relative abundances of phyla present in the landfill metagenomes. Twenty-five phyla were present at greater than 1% relative abundance in at least one landfill sample (Fig. 2). Phylum level profiles were relatively consistent between the 16S rRNA gene amplicon and metagenomic sequencing data (Fig. 2). A notable exception was the *Patescibacteria* (Candidate Phylum Radiation), which make up a comparatively reduced proportion of the 16S rRNA gene amplicon results (max relative abundance of 30.78%, in GW1) but exhibit the highest relative abundances in the metagenomic data (mean relative abundance of 34% and max relative abundance of 79%, in GW1, based on the coverage of the ribosomal protein-encoding scaffolds). The *Bacteroidota* (mean: 16%, max: 31.89% in CLC_T1), *Firmicutes* (mean: 10.19%, max: 28.74% in CLC_T1), and *Proteobacteria* (mean: 10%, max: 28% in LW2) were also highly abundant across the landfill sites.

### Alpha and beta diversity metrics

**Fig. 3:**
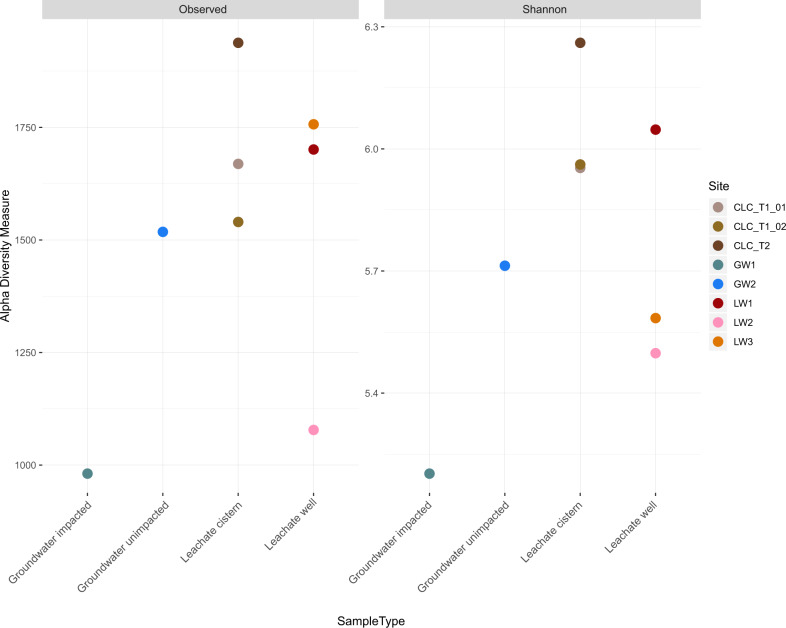
Observed diversity and Shannon index for the eight landfill-associated samples. Observed 16 S rRNA gene amplicon ASVs fall between 986-1998 total ASVs for each sample, indicating similar levels of microbial community heterogeneity across sites. All sites show a high level of diversity, with Shannon indices greater than 5. Samples are grouped by sample type on the *x* axis.

Alpha and beta diversity metrics were calculated based on the 16S rRNA gene amplicon sequences using QIIME2 and the phyloseq package in R [43]. All of the landfill samples had a Shannon index above 5.0 for the 16S rRNA gene amplicon data (Fig. 3). There was no significant difference between the sample types (groundwater, leachate wells, leachate cisterns) when considering Faith’s phylogenetic diversity (Supplementary Fig. 1A). The eight samples also exhibited high Pielou’s evenness (J’ > 0.74) with no significant differences between sample types (Supplementary Fig. 1B).

Principle coordinates analysis (PCoA) plots using weighted and unweighted UniFrac distances based on 16S rRNA gene amplicon ASVs showed separation of the samples by type (Supplementary Fig. 2). The inclusion of abundance data in the weighted UniFrac analysis increased the explained variation on axes 1 and 2 by a combined 24.1%, suggesting that presence/absence and phylogenetic distance data implemented in the unweighted UniFrac are not sufficient to resolve the differences in beta diversity between sites in two dimensions. The inclusion of differences in abundance and overlap of ASVs between sites increased separation of the samples by type.

### Diversity of ASVs

**Fig. 4:**
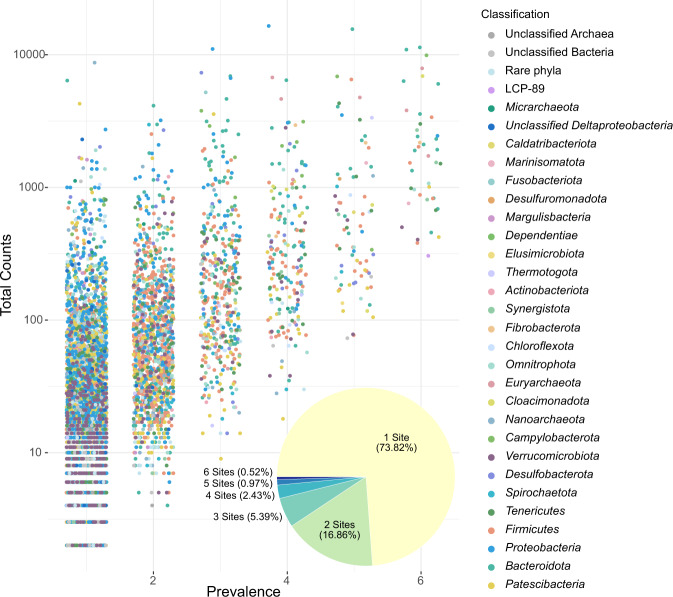
Prevalence of 16 S rRNA amplicon ASVs across sampling sites. ASVs with individual counts greater than two from the top 25 most abundant phyla or present in four or more sites were included. The pie chart insert shows the proportion of ASVs present in one to six sites. Data from CLC_T1 0.1 and 0.2 µm filters were combined, because these samples represent the same biomass from the same site and sampling time.

The prevalence of 16S rRNA ASVs was determined using phyloseq and visualized in ggplot2 [45] in R (Fig. 4). The abundance of ASVs present at 5 or more sites was summarized by phylum (Supplementary Fig. 3). Although phylum level diversity was relatively consistent across the composite leachate cistern, leachate wells, and GW1 sample, the diversity at the ASV level is nearly entirely non-overlapping. The majority of ASVs identified from the top 25 phyla are present in only a single sample (Fig. 4) with only 121 of 8030 ASVs present across five or more samples (Fig. 4 and Supplementary Fig. 3). In addition to the top 25 phyla, ASVs belonging to LCP-89, *Micrarchaeota*, and an unclassified group of *Deltaproteobacteria* were also present in five or more sites. The abundance of phyla with populations across 5 or more phyla ranges by several orders of magnitude from 134 total ASV counts for *Elusimicrobiota* to 83,545 total ASV counts for *Bacteroidota* (Supplementary Fig. 3). Of the 8030 ASVs, 73.82% were found in only one sample and the number of ASVs shared between any two sites is at maximum 1165 ASVs (Fig. 4 and Supplementary Table 1). Principle component analysis (PCA) for all ASVs showed separation of composite leachate cisterns, leachate wells, and groundwater wells is driven by highly abundant ASVs (Supplementary Fig. 4A). When considering only ASVs present at five or more sites, LW2 is separated from LW1 and LW3 along PC2 and GW1 is separated from all other sites along PC1 (Supplementary Fig. 4B).

### Microbial diversity at groundwater wells

There are marked differences in the groundwater microbial communities from GW1 and GW2, the leachate-impacted and unimpacted wells, respectively. GW1 has a high abundance of *Patescibacteria* while also sharing a more similar phylum-level profile to the leachate wells than to GW2 (Fig. 2). The sample from GW2 had insufficient microbial biomass for metagenomic sequencing, but 16S rRNA gene amplicon sequencing showed that GW2 has a distinct microbial community compared to all other sites, including a higher relative abundance of *Nanoarchaeaota* (20.7%) and *Omnitrophota* (14.1%) (Fig. 2). The difference in microbial community composition between GW1 and GW2 is also reflected in their alpha diversity metrics. GW1 has the lowest Shannon index of the eight samples (Fig. 3) as well as the lowest Faith’s phylogenetic diversity (Supplementary Fig. 1A). A lower richness and evenness are expected in GW1, as the mixing of leachate and groundwater creates a suboptimal environment for microorganisms adapted to either environment [57].

### Analysis of geochemical parameters

**Fig. 5:**
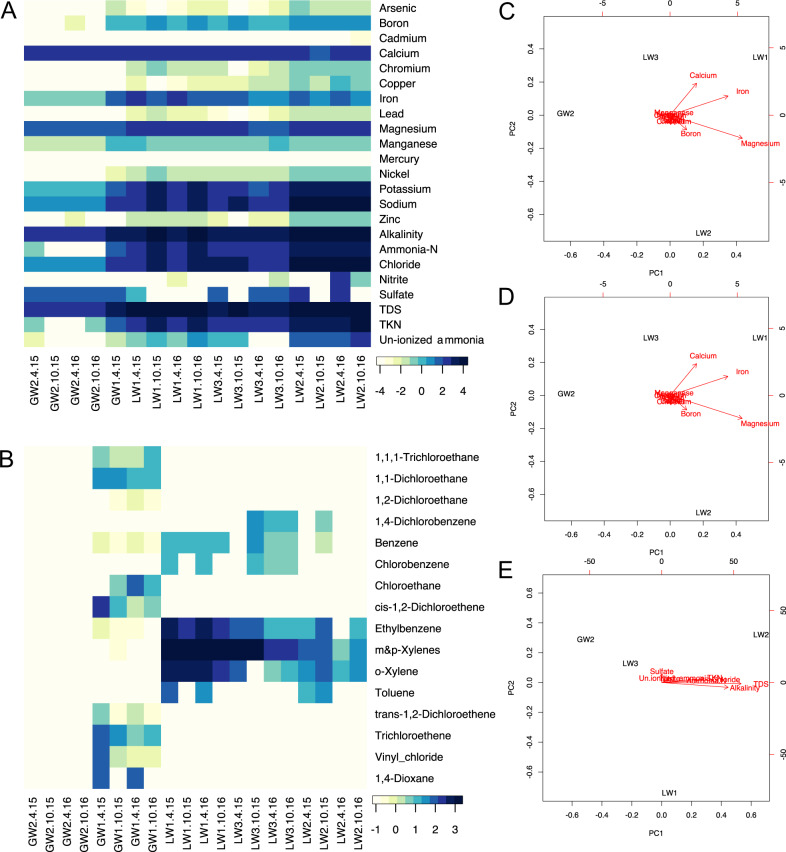
Environmental variation between landfill and aquifer sites for non-volatile (mg/L) and volatile (µg/L) compounds. **A** Heat map of non-volatile compound concentrations and other site parameters (log10 transformed) across four dates for the three leachate wells and the unimpacted groundwater well. Only one date (April 2011) was available for the impacted groundwater well (GW1). Stars indicate low levels of cadmium and mercury not visible on the heatmap that may be biologically relevant. **B** Heat map of volatile compound concentrations (log10 transformed) across four dates for the leachate and groundwater wells. **C** Principal component analysis of metal concentrations at the three leachate wells and the unimpacted groundwater well. Metal concentration data was square root transformed. PC1 explains 79.6% of variation and PC2 explains 18.4% of variation. **D** Principal component analysis of volatile compound concentrations at the three leachate wells and the unimpacted groundwater well. Volatile compound concentration data were square root transformed. PC1 explains 97.4% of variation and PC2 explains 2.4% of variation. **E** Principal component analysis for non-metal, non-volatile compounds, derived from measurements at the three leachate wells and the unimpacted groundwater well. Concentration data was square root transformed. PC1 explains 99% of the variation.

Geochemical parameters, including concentrations of volatile and non-volatile compounds, are measured quarterly by a contracted monitoring company. The statistical power available for analysis of geochemical parameters in the landfill was limited by the availability of data. Non-volatile compound measurements were only available for four sites and volatile compound measurements for five sites (Supplementary Table 2). Non-volatile and volatile compound concentrations varied significantly between sites when compared using an ANOVA (*p* < 9.14e^−14^ and *p* < 2e^−16^, respectively), with a large range between sites for several non-volatile and volatile compounds (Fig. 5). The date of measurement was not significant for either volatiles or non-volatiles when compared using an ANOVA (*p* = 0.56 and *p* = 0.73, respectively). The April and October 2016 measurements for the PCA analysis were averaged to estimate conditions during the July sampling for microbial biomass. Sodium and potassium were removed as outliers because their excessively high concentrations in LW2 (Supplementary Table 2) caused their variance to mask any differences in other compounds in the analyses. From the PCA, calcium, iron, magnesium, and to a lesser degree, boron contributed to the differences between the leachate wells and GW2 (Fig. 5C). For the volatile compounds, nearly all of the observed variation is explained by PC1 (97.4%), largely due to the punctuated presence of m- & p- xylenes in LW1 and LW3, and of o. xylenes and ethylbenzene in LW1 (Fig. 5B, D).

### Methanogen populations

The potential for methanogenesis was determined using annotations for the alpha subunit of methyl-coenzyme M reductase (McrA; K00399). A total of 94 McrA protein-coding sequences were identified from the six metagenomes, ranging from 1 (GW1) to 25 (CLC_T2) per metagenome (Table 1). Of these, 31 were encoded on scaffolds >2.5 kb, and 17 were binned into high quality MAGs (Table 2). The taxonomic affiliations of the *mcrA*-encoding MAGs include nine methylotrophic members of the *Methanomethylphilaceae* as well as seven acetoclastic MAGs from the *Methanoregulaceae* (3), *Methanotrichaceae* (3), and *Methanocullaceae* (1) (Table 2). The final *mcrA*-encoding MAG is classified as a *Methanofastidiosaceae*, predicted to use methylated thiols as input to the methanogenesis pathway. McrA-encoding scaffolds and MAGs were moderately abundant, with an average scaffold coverage of 17.72 (dataset average: 13.5, median = 7.4), and MAG average coverages from 6.36–42.32 (Table 2, average coverage of all MAGs: 14.95–31.02 across the six metagenomes).

**Table 2:** Genome information for MAGs carrying VOC degradation genes or McrA as a marker for methanogenesis.

MAG	JGI gene accession	MAG cov.	MAG comp.	MAG cont.	Relevant protein	MAG Phylum	Lowest named taxonomic classification
VOC degradation							
LW3_42	Ga0172380_100000381	17.09	96.89	1.45	EbdA	*Gammaproteobacteria*	F: *Rhodocyclaceae*
	Ga0172380_10000181129						
	Ga0172380_1000070814						
	Ga0172380_1000072710						
	Ga0172380_100025954						
	Ga0172380_100042083						
	Ga0172380_100073127						
LW3_67	Ga0172380_1000040665	50.62	95.16	6.85	EbdA	*Gammaproteobacteria*	G: *Sterolibacterium*
	Ga0172380_1000040668						
	Ga0172380_100008082						
LW3_68	Ga0172380_100006492	16.83	90.1	6.08	AbcA	*Gammaproteobacteria*	G: *Sterolibacterium*
LW2_26	Ga0172382_100080076	118.64	97.01	0	DxmA	*Actinobacteriota*	F: *Solirubrobacteraceae*
Methanogenesis							
CLC_T1_10	Ga0172378_10000287	16.39	98.43	1.15	McrA	*Thermoplasmatota*	F: *Methanomethylophilaceae*
CLC_T1_33	Ga0172378_10005622	8.03	89.11	0.81	McrA	*Thermoplasmatota*	G: *Methanomethylophilus*
CLC_T1_68	Ga0172378_10009832	39.94	86.02	0.81	McrA	*Thermoplasmatota*	F: *Methanomethylophilaceae*
CLC_T1_8	Ga0172378_10005098	14.81	92.34	0	McrA	*Thermoplasmatota*	F: *Methanomethylophilaceae*
CLC_T2_100	Ga0172377_10002184	8.72	92.08	0.81	McrA	*Thermoplasmatota*	F: *Methanomethylophilaceae*
CLC_T2_32	Ga0172377_10000074	9.59	87.9	0.81	McrA	*Thermoplasmatota*	F: *Methanomethylophilaceae*
CLC_T2_5	Ga0172377_10038920	14.03	73.39	5.24	McrA	*Thermoplasmatota*	F: *Methanomethylophilaceae*
CLC_T2_58	Ga0172377_10004413	11.28	85.96	5.88	McrA	*Halobacterota*	G: *Methanoculleus*
CLC_T2_72	Ga0172377_10009827	6.36	74.06	0.31	McrA	*Thermoplasmatota*	G: *Methanomethylophilus*
LW1_13	Ga0172381_10006707	15.69	95.1	1.96	McrA	*Halobacterota*	G: *Methanothrix*
LW1_26	Ga0172381_10000056	42.32	96.93	1.31	McrA	*Halobacterota*	G: *Methanoregula*
LW1_61	Ga0172381_10028869	18.16	72.69	3.59	McrA	*Halobacterota*	G: *Methanoregula*
LW2_109	Ga0172382_10001117	10.44	94.37	3.47	McrA	*Euryarchaeota*	G: *Methanofastidiosum*
LW2_65	Ga0172382_10003740	6.99	77.24	0	McrA	*Thermoplasmatota*	F: *Methanomethylophilaceae*
LW2_73	Ga0172382_10006116	14.38	99.34	0	McrA	*Halobacterota*	G: *Methanothrix*
LW3_11	Ga0172380_10022908	10.66	71.14	2.66	McrA	*Halobacterota*	G: *Methanothrix*
LW3_55	Ga0172380_10015880	7.69	70.57	1.99	McrA	*Halobacterota*	G: *Methanoregula*

*MAG cov*. average coverage of scaffolds within the MAG based on read mapping, *MAG comp.* MAG completion as calaculated by CheckM, *MAG cont.* MAG contamination as calculated by CheckM. Taxonomy is based on GTDB-Tk taxonomic assignment.

### VOC degradation capacity

An annotation-based screen was conducted to assess the potential capacity for volatile organic compound degradation, focusing on anaerobic degradation of chlorinated solvents (ethenes, ethanes, benzenes), BTEX compounds, 1,4-dichlorobenzene and chlorobenzene, and 1,4-dioxane as the predominant VOCs impacting the site. Following curation of annotated proteins for phylogenetic consistency and homology to characterized VOC degrading proteins, 111 protein-coding genes with VOC-degradation relevance were identified (Table 1), 12 of which were associated with high quality MAGs (Table 2).

**Fig. 6:**
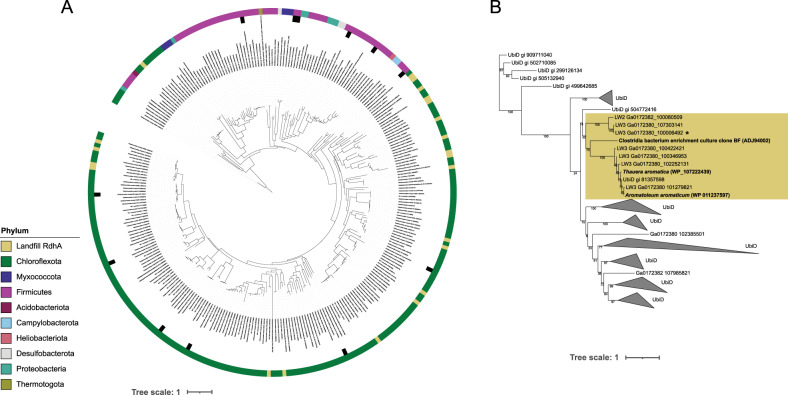
Maximum likelihood trees placing landfill-derived reductive dehalogenases (RdhA) and anaerobic benzene decarboxylases (AbcA) within their respective gene families. **A** RdhA ML tree, with outer ring indicating phylum of origin for the RdhA and inner ring denoting the characterized RdhA with known substrates in black. Landfill sequences are highlighted in yellow. The tree was imaged using iTol [88] and the muted colour-blind friendly palette designed by Paul Tol. **B** AbcA ML tree with UbiD outgroup. AbcA clade is highlighted with yellow with reference sequences bolded; all collapsed clades are UbiD sequences exclusively. The AbcA sequence associated with a high quality MAG is starred. For both trees, bootstrap values are present as numeric values on the nodes.

For the reductive dehalogenases, 76 genes were detected, but only 22 passed the length threshold for phylogenetic placement and substrate specificity examination. All reductive dehalogenase genes, including ones too short for placement, were identified from the landfill metagenome samples. The metagenome for GW1 did not contain any reductive dehalogenase genes, despite this being the only site where chlorinated solvents were detected in the geochemical analyses (68 µg/L total concentration, Supplementary Data 2). Reductive dehalogenases have been identified from a diverse suite of organisms with organohalide respiration capacity [55]. All RdhA genes detected at the southern Ontario landfill are most closely related to those from the *Chloroflexota* organisms *Dehalococcoides* and *Dehalogenimonas* (Fig. 6). One partial protein gene from LW3 has high homology to TceA, the *Dehalococcoides*-encoded RdhA involved in degradation of trichloroethene (TCE) to dichloroethene (DCE) [58]. In our screen, there were no homologs to VcrA and BvcA, the two known proteins that can dechlorinate vinyl chloride (VC) to non-toxic ethene [59, 60], indicating VC degradation may be limited or absent. No other landfill-derived sequences were associated with reference sequences with known substrate specificities (Fig. 6).

For anaerobic benzene degradation, 183 AbcA hits were identified via BLASTp, with 177 passing the length requirement. Based on the phylogeny containing AbcA and UbiD representative proteins, 7 of these sequences place within or next to the AbcA clade, and were scored as potential AbcA in the landfill metagenomes (Table 1, Fig. 6). AbcA genes were identified from LW2 and LW3, while benzene was detected at LW1, LW3, and GW1. One AbcA gene was associated with a MAG from the gammaproteobacterial genus *Sterolibacterium* (LW3_68). Current characterized Benzene degraders with AbcA are from the genera *Thauera* and *Aromatoleum*, making this an expansion of the taxonomic as well as sequence diversity of this recently described remediation-relevant protein family.

Anaerobic xylene and toluene degradation was screened based on presence of benzylsuccinate synthase (BssA) [61, 62]. An initial 147 annotated proteins were curated to 26 based on length requirements and phylogenetic affiliations, with BlastKoala confirming annotation for 22 of these as well as an additional 14 proteins (Supplementary Data 2). The 40 proteins passing tree-based curation and/or BlastKoala annotation are reported in Table 1. Unlike for chlorinated solvents, xylene and toluene degradative capacity tracks with contaminant concentrations: LW1 and LW3 have both the highest concentration of xylene and toluene, as well as the highest count of predicted benzylsuccinate synthases (LW1: 1307 µg/L, 10 BssA; LW3: 211.2 µg/L, 26 BssA). LW2 and GW1 have lower concentrations of xylene and toluene, and fewer detected BssA (LW2: 43.3 µg/L, 3 BssA; GW1: 0.1 µg/L, 1 BssA). No *bssA* genes were detected in the CLC metagenomes.

Ethylbenzene degradation capacity was examined through ethylbenzene dehydrogenase (EbdA), a member of the dimethylsulfoxide (DMSO) reductase family [63]. An initial 112 sequences were cut to 41 following length filtration, with 40 placing as putative EbdAs on a tree rooted with nitrate reductases, dimethylsulfoxide dehydrogenases, selenate reductases, perchlorate reductases, and chlorate reductases, following the tree in [64]. Of these, ten were associated with two MAGs, one member of the *Rhodocyclaceae* encoded seven EbdA proteins (LW3_42), and one member of the *Sterolibacterium* encoded 3 (LW3_67; not the same MAG as encoded the AbcA). Ethylbenzene was detected at all four sites with geochemical data. LW1 had the highest concentration (275 µg/L), with no detected EbdA from the corresponding metagenome. LW2 and LW3 had moderate levels of ethylbenzene, and strong EbdA counts (LW2: 8.25 µg/L, 19 EbdA; LW3: 17.5 µg/L, 19 EbdA). The remaining two EbdA were identified from CLC_T2, while GW1, which had trace ethylbenzene (0.1 µg/L) and CLC_T1 had no EbdA detected.

Anaerobic degradation of chlorobenzene and 1,4-dichlorobenzene is catalyzed by chlorobenzene dihydrodiol dehydrogenase (TcbB). Only 4 hits were identified based on KO annotations, all from LW2. Of these, only 1 had >75% ID at the amino acid level to chlorobenzene dihydrodiol dehydrogenase as its closest database homolog. The other three were more closely related to cis-2,3-dihydrobiphenyl-2,3-diol dehydrogenases. None were assigned to the correct annotation using BlastKoala. The one putative TcbB is reported here. LW1 and LW3 have detectable chlorobenzenes in the leachate (6.0 and 15.3 µg/L net chlorobenzenes, respectively), while LW2, the sample with a putative TcbB, did not have any detectable chlorobenzenes.

1,4-dioxane degradation was included in this screen as a contaminant of interest for the site engineers. There are currently no known anaerobic 1,4-dioxane degradation pathways. To examine the latent potential for aerobic dioxane degradation, we focused on DxmA and PrmA, the two enzymes capable of 1,4-dioxane degradation without requiring induction from a co-contaminant (e.g., toluene, propane) [65–69]. From an initial set of 20 putative DxmA and 33 PrmA, only one DxmA, from LW2, passed length requirements and phylogenetic tree-based curation. Clustering with the DxmA from *Pseudonocardia dioxanivorans*, this protein is annotated with an aromatic and alkene monooxygenase hydroxylase domain by NCBI’s conserved domain database, and was encoded on a high quality MAG (LW2_26). Notably, LW2_26, from an unclassified genus within the family *Solirubrobacteraceae*, is the 14th most abundant MAG across all metagenomes (average coverage = 118.64; Table 2). 1,4-Dioxane was only detected at GW1 (26 µg/L), whose paired metagenome contained no identified dioxane degradative capacity.

The curated VOC degradation proteins were moderately abundant, with average scaffold coverages for genes on scaffolds over 2.5 kb ranging from 10.2–26.94 (BssA = 10.2; RdhA = 11.6; TcbB (one gene) = 17.4; DxmA (one gene) = 19.4; EbdA = 24.6; AbcA = 26.9; all scaffolds’ average coverage = 13.5, median = 7.4).

## Discussion

### Phylum level diversity

The phylum level diversity in this landfill is generally consistent with other studies, where *Bacteroidota*, *Firmicutes*, and *Proteobacteria* are frequently detected as the most abundant bacterial phyla in landfills [7, 10, 11]. Interestingly, our study uncovered a high abundance of *Patescibacteria* in the landfill that had not been found in previous studies [7, 11]. Previous landfill microbial diversity studies have relied on 16S rRNA gene amplicon sequencing, which may have systematically underestimated the abundance of *Patescibacteria* due to primer mismatches and long insertions in the rRNA genes [70]. *Patescibacteria* can be more robustly identified using metagenomic techniques [71].

### Alpha diversity

Shannon Indices above 5 for each site indicates that the landfill sites each have relatively high levels of microbial richness and evenness. This is greater than seen in some soil microbial communities, such as those in the Canadian prairies (1 < *H* < 4.5), and similar to others, such as in forest and agricultural land in Burgundy, France (4.5 < *H* < 6.1) [72, 73]. Soil microbial communities show variation in richness and evenness with latitude and temperature as well as nutrient inputs into the system [74]. In comparison, there were no significant differences among our samples, however, GW1 showed lower richness and overall phylogenetic distance than the other samples, but similar evenness with GW2. The alpha diversity of these groundwater wells is greater than has been reported for other groundwater aquifers, with Shannon Index values typically reported as below 4 and as low as 0.47 for some [75, 76]. The landfill leachate well and composite leachate cistern sample diversities are consistent with the findings of Stamps et al. (2016), who showed high richness and evenness across the 19 U.S. landfills in their study. Similarly, the leachate richness and evenness is consistent with municipal wastewater values in Belgium (4.71 < *H* < 5.26 for bacteria) and China (5.80 < *H* < 6.23) [77, 78]. The implication of these high alpha diversity values is that the landfill microbial communities consist of phylogenetically diverse microorganisms that have relatively equal abundances at the species level regardless of the presence of dominant phyla.

### Diversity of amplicon sequence variants

Although leachate does not provide a complete representation of the microbial community present within the landfill [79], we were specifically interested in shared microbial populations. The circulating leachate would thus potentially over-estimate prevalence of microbial populations across the site. This makes the observation that the majority (73.82%) of the ASVs are limited to one sample, even more striking. Of the 2102 ASVs shared between at least 2 sites, 1135 are shared between CLC_T1 and CLC_T2, suggesting that there is some proportion of the community in the composite leachate cistern that is maintained over at least a one-week interval or otherwise continually entering the cistern from the leachate wells. LW1 and LW2 consistently share more ASVs with CLC_T1 and CLC_T2 than LW3, suggesting LW1 and LW2 contribute greater amounts of leachate to the composite leachate cistern (Supplementary Table 1). Interestingly, GW2, the unimpacted well, shares more ASVs in common with the leachate wells and the composite leachate cistern than GW1, the impacted well, with 399 shared ASVs to 186 ASVs, respectively (Supplementary Table 1). This is in contrast to the phylum-level differences seen for GW2 compared to landfill samples, and suggests there is some interconnectivity even between the unimpacted groundwater and the leachate—most likely caused by groundwater infiltrating the landfill. Leachate near the LW3 location is known to impact groundwater near GW1 and this is supported by the higher number of shared ASVs between GW1 and LW3 than any of the other sites (Supplementary Table 1). The high number of ASVs present at only one site indicate rare operational taxonomic units, as described by Köchling et al. (2015) and Cardinali-Rezende et al. (2016), may play an oversized role in landfill microbial communities. Rare or non-prevalent organisms are hypothesized to act as seeder or starter communities during environmental changes or disturbances. Under this hypothesis, the landfill is undergoing a constant state of disturbance and change, driving the extreme heterogeneity observed.

### Microbial diversity of groundwater wells

The two groundwater wells allow a comparison between a natural groundwater environment and a leachate contaminated environment. GW1 is situated closer to the active landfill, and leachate from the region around LW3 is leaking into the groundwater near GW1 (Fig. 1). GW2 is further from the active landfill and is embedded in a region of the aquifer that shows no evidence of contamination from the landfill leachate (Fig. 1, top left corner). Landfill leachate solubilizes a number of potentially harmful chemicals [7], and can be enriched with carbon and nitrogen [57]. Leachate leakage is reflected in the geochemistry data, with a number of metals and volatile compounds detected at GW1 but not GW2 (Fig. 5). The higher concentration of 1,4-dioxane, vinyl chloride, and chloroethane compounds in GW1 in comparison to the leachate wells (Fig. 5) may be due to loss of the landfill microorganisms capable of degrading those compounds in the aquifer near GW1. Lu et al. (2012) showed that when landfill leachate contaminates groundwater, the landfill microbes are unable to survive in the more dilute groundwater, and the addition of chemicals from the leachate negatively impacts the native groundwater microorganism diversity. Some natural attenuation of contaminants can occur in aquifers with leachate plumes, but more information regarding the functional abilities of these microbial communities is needed to understand the dynamics of these polluted systems [57, 80].

### Methanogen populations

The methanogen populations identified spanned three phyla and six families, but no sample contained more than five methanogen MAGs, and none were particularly abundant. No *Methanobacteriales* were identified, and only four *Methanomicrobiales* MAGs were identified, with a larger proportion from the *Methanomethylophilaceae*, a group less frequently associated with landfills. Only two MAGs were markedly more abundant than the average in the datasets—a *Methanomethylophilaceae* from CLC_T1 (average coverage = 39.9) and a *Methanoregula* from LW1 (average coverage = 42.3) (Table 2). No *mcrA*-containing MAGs associated with the anaerobic methane oxidizing ANME lineages were identified, in contrast to a survey of solid waste along a depth/age transect in a Chinese landfill [81].

All identified methanogens were predicted to be exclusively acetoclastic or methylotrophic methanogens, with the exception of one *Methanofastidiosaceae* predicted to use methylated thiols. The observed distribution of methanogenesis pathways suggests the landfill has a restricted availability of substrates to support methanogenesis, a hallmark of older waste. No cosmopolitan methanogens, capable of using multiple substrates for methane production, were identified. Methanogens were a small fraction of the total microbial community (~1.6% of total assembled and binned reads), which also suggests the landfill has moved past the rapid methanogenesis phase and into the decelerating methanogenesis phase, or phase IV of the landfill life cycle [82].

### VOC degradation capacity

Examination of the VOC degradation capacity of the landfill sites identified a diverse suite of microbial mechanisms for contaminant degradation. We expected to see either (i) an inverse trend wherein sites with degradation capacity had lower contaminant levels due to active degradation or (ii) a positive correlation, where sites with higher contaminant levels supported higher abundances of degraders who were then represented in the metagenomes. What was observed was a mix of these two scenarios. RdhA, BssA, and EbdA were present at high enough counts to consider trends across sites. For RdhA, catalyzing dehalogenation of chlorinated solvents, scenario (i) was observed, where GW1 is the only site with detected contaminants and is also the only site with no relevant degradation genes in its metagenome. For BssA, the opposite was observed, fitting scenario (ii), with the highest counts of BssA associated with sites where concentrations of benzene were significantly higher. EbdA, catalyzing initial ethylbenzene degradation, showed a mixed trend. The site with highest ethylbenzene concentrations, LW1, had no detected EbdA, and other sites with moderate concentrations of ethylbenzene hosted high numbers of EbdA (LW2, LW3). The site with only trace ethylbenzene had no detected EbdA (GW1). From this, it is clear different processes are controlling contaminant concentrations at the site. Detection of novel VOC degradation genes expands these gene families, and provides new targets for characterization. This is particularly important for AbcA, a relatively recently identified protein [47, 56], where the seven new sequences represent an important addition to this gene family (Fig. 6), including a new taxonomic affiliation for this activity with one AbcA encoded on a unclassified *Solirubrobacteraceae* genome (Phylum *Actinobacteriota*). Also of note is the identification of a DxmA protein encoded on a highly abundant *Sterolibacterium* MAG, suggesting this activity may be selected for within the landfill. 1,4-dioxane degradation activity is highly sequence-specific [69], and this predicted DxmA requires confirmation via enrichment or biochemical assays.

GW1 is notable as the site with the broadest contaminant profile with the lowest numbers of contaminant degradation genes identified—further indication this is a disturbed, impacted microbial community and with implications for contaminant fate in the aquifer. In particular, the absence of reductive dehalogenases in GW1 is interesting on two counts. First, GW1 is the only surveyed site with detected chlorinated solvents, with a total concentration of 68.02 µg/L (made up of chloroethane, 1,1-dichloroethane, trichloroethane, TCE, and <5 µg/L of six other chlorinated solvents, Supplementary Data 2). Second, reductive dehalogenases were detected in LW3, GW1’s paired geographic sample across the landfill boundary, and the known source of leachate infiltration into the aquifer. The capacity to degrade chlorinated solvents in GW1 was below our detection limit or absent, indicating that the solvents transferring across the landfill barrier are persisting in the environment, while the microorganisms capable of degradation are either not moving into or are not persisting in the disturbed leachate/groundwater mixed environment. Further study across time may clarify whether VOC-degrading organisms eventually establish activity in the aquifer or if more targeted remediation efforts will be needed.

For the leachate wells and potential fate of chlorinated solvents within the landfill, it is important to note that the substrates of most reductive dehalogenases are unknown, and so, while the number of chlorinated compounds that can be degraded in the leachate is likely quite high based on diversity of reductive dehalogenases identified (Fig. 6), it is not possible to identify these substrates without targeted experiments. *Dehalococcoides* and *Dehalogenimonas* are lineages of obligate organohalide respirers – they are only able to survive and grow on chlorinated solvents [55, 83]. These organisms are thus highly useful in remediation efforts because their activity is tightly targeted [84, 85], and the novel landfill-derived RDases represent potentially interesting remediation tools.

Contrasting the metabolic potential for methanogenesis with the potential for VOC degradation, methanogenesis is the more abundant function at the site. The VOC degradation capacities examined, in aggregate, share approximately the same prevalence, abundance, and association with MAGs as McrA alone. None of the functions were specifically abundant with the exception of the putative DxmA. VOC degradation genes were present at similar proportions to *mcrA*s on short versus >2.5 kb scaffolds (36.3% and 32.9%, respectively), as well as on MAGs (11% and 18% respectively). Their scaffold coverages and host MAG coverages were near to the mean for the datasets, indicating these functions are not dominant or enriched within the landfill environments surveyed.

## Conclusions

The phylum level profiles for the composite leachate cistern, leachate wells, and GW1 are consistent with previous landfill microbial community studies, with *Bacteroidota, Firmicutes*, and *Proteobacteria* among the most abundant phyla. Using metagenomic sequencing, we additionally identified *Patescibacteria* as one of the dominant phyla in the landfill, a group that may have been missed in previous studies relying on 16S rRNA gene amplicon sequencing. Methanogens were only moderately abundant in the landfill, with limited substrate specificities, indicating the landfill is in phase 4 of a landfill lifecycle, with decelerated methane production occurring. At the species/ASV level, microbial heterogeneity is markedly higher than previously reported for landfill environments, with little overlap between communities separated by short distances or one week in time. Geochemical conditions showed high variance across the site, and were generally uncorrelated with microbial community memberships and anaerobic volatile organic compound degradation capacities. A suite of novel contaminant degradation genes were identified from the landfill, including reductive dehalogenases and anaerobic benzene carboxylases. Taken together, our findings have implications for waste management strategies, including targeted remediation efforts, as establishing or supporting populations and activities of interest will be challenging given the dynamic nature of the landfill microbial communities.

## Supplementary information

Supplementary information

Supplemental data file 1

Supplemental data file 2

## Data Availability

The assembled and annotated Southern Ontario metagenomes are deposited on IMG with the following IMG Genome IDs (Taxon Object IDs): 3300014203 (CLC1_T1), 3300014206 (CLC1_T2), 3300014204 (LW1), 3300015214 (LW2), 3300014205 (LW3), and 3300014208 (GW1). Specific genes associated with methanogenesis and VOC degradation are listed by JGI accession number and gene category in Supplementary File 2. The 16S rRNA amplicon sequences have been deposited in the NCBI SRA archive under the bioproject PRJNA706007 and Biosamples SAMN18111220-7.
