# Supplementary material for: Substrate-restricted methanogenesis and limited volatile organic compound degradation in highly diverse and heterogeneous municipal landfill microbial communities
Source: ISME Commun. 2022 Jul 13;2:58. doi: 10.1038/s43705-022-00141-4 (PMC9723747; doi:10.1038/s43705-022-00141-4)
Supplement: Supplementary file 1 — Supplementary information [file 43705_2022_141_MOESM1_ESM.pdf]

Substrate-restricted methanogenesis and limited volatile organic compound degradation in highly diverse and heterogeneous municipal landfill microbial communities

Alexandra H. Sauk[1] and Laura A. Hug[1]

Department of Biology, University of Waterloo, Waterloo, ON, Canada

Supplemental Tables and Figures

Supplemental Table 1: Number of shared 16S rRNA gene amplicon ASVs between samples.

	CLC_T1	CLC_T2	LW1	LW2	LW3	GW1	GW2
CLC_T1		1135	384	301	223	27	280
CLC_T2	1135		431	486	234	13	295
LW1	384	431		195	476	59	132
LW2	301	486	195		119	8	142
LW3	223	234	476	119		131	116
GW1	27	13	59	8	131		46
GW2	280	295	132	142	116	46	

Supplemental Table 2: Averaged environmental variable concentrations based on measurements from April and October 2016 for the groundwater wells and the three leachate wells. Additional variables not included in the PCA analyses are presented in Supplemental Data Sheet 1.

Environmental Parameter	*units*	*GW1**	*GW2*	*LW1*	*LW2*	*LW3*
Arsenic	mg/L	0.02	0	0	0.04	0
Boron	mg/L	0.79	0.01	2.10	7.53	1.45
Cadmium	mg/L	0	0	$0.10e^{-3}$	0	0
Calcium	mg/L	240	86.50	175	93.25	155
Chromium	mg/L	0	0	0.04	0.40	0.02
Copper	mg/L	0	0	0.01	0.36	0.03
Iron	mg/L	40	0.37	56.50	11.78	10.55
Lead	mg/L	0	0	0.01	0.05	0
Magnesium	mg/L	38	24	145.50	145	58.50
Manganese	mg/L	1.20	0.03	0.32	0.29	0.67
Mercury	mg/L	0	0	0	$0.30e^{-3}$	0
Nickel	mg/L	0.01	0	0.05	0.36	0.02
Potassium	mg/L	16	1.40	345	917.50	79.50
Sodium	mg/L	76	4.55	615	2525	200
Zinc	mg/L	0	0	0.06	0.28	0.01
Alkalinity total (as $CaCO_3$)	mg/L	687	250	2800	7025	1200
Ammonia-N	mg/L	18	0	275	1375	123.50
Chloride	mg/L	180	6.70	655	3050	255
Nitrite	mg/L	0	0	0.01	46.24	0.01
Sulfate	mg/L	17	60.50	0	70	51.50
Total dissolved solids	mg/L	1040	315	3255	9657.50	1345
Total Kjeldahl nitrogen	mg/L	20	0.11	315	1925	135
Un-ionized ammonia	mg/L	0.03	0	2.32	99.88	0
1,4-Dichlorobenzene	ug/L	0	0	0	0	11.50
Benzene	ug/L	0.69	0	13.50	0	5.50
Chlorobenzene	ug/L	0	0	6	0	3.80
Ethylbenzene	ug/L	0.13	0	275	8.25	17.50
m&p-Xylenes	ug/L	0.07	0.06	1040	26.25	200
o-Xylene	ug/L	0.06	0	250	17.05	11.20
Toluene	ug/L	0	0	17	0	0

*All measurements for GW1 except the volatile compounds (1,4-dichlorobenzene to toluene) are from April 2011 and are for comparison only. These values were not included in the PCA analysis.

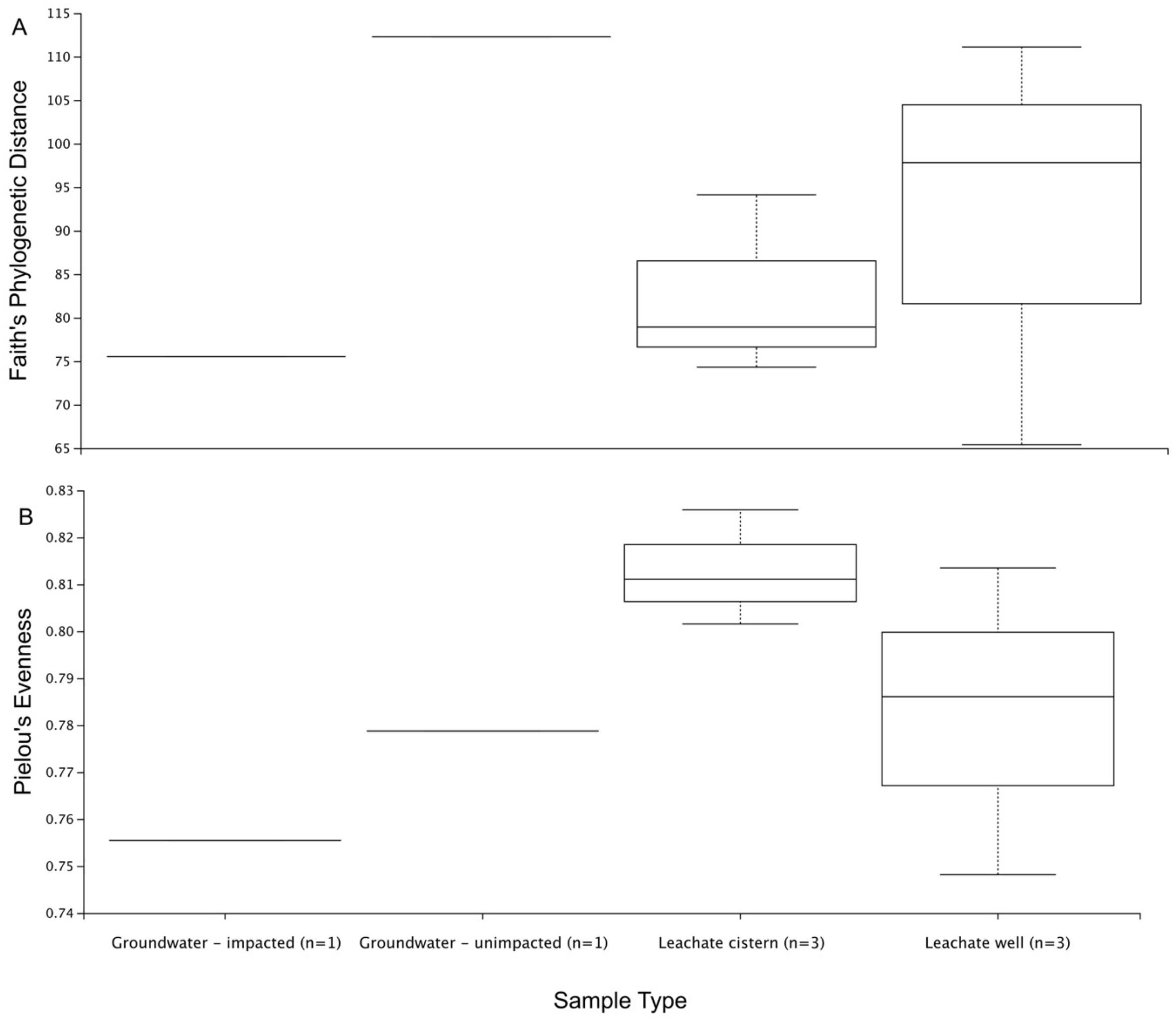

Supplemental Figure 1: Alpha diversity analyses by sample type for Faith's Phylogenetic Diversity and Pielou's Evenness. A) Faith's phylogenetic diversity. A Kruskal-Wallis test for all groups is not significant (H=2.77 and p=0.43). Kruskal-Wallis pairwise tests are also not significant for all pairs (p≥0.18). B) Pielou's evenness. All sample types show high levels of evenness among their respective species (J' > 0.74). A Kruskal-Wallis test for all groups is not significant (H=3.22 and p=0.36). Kruskal-Wallis pairwise tests are also not significant for all pairs (p≥0.18).

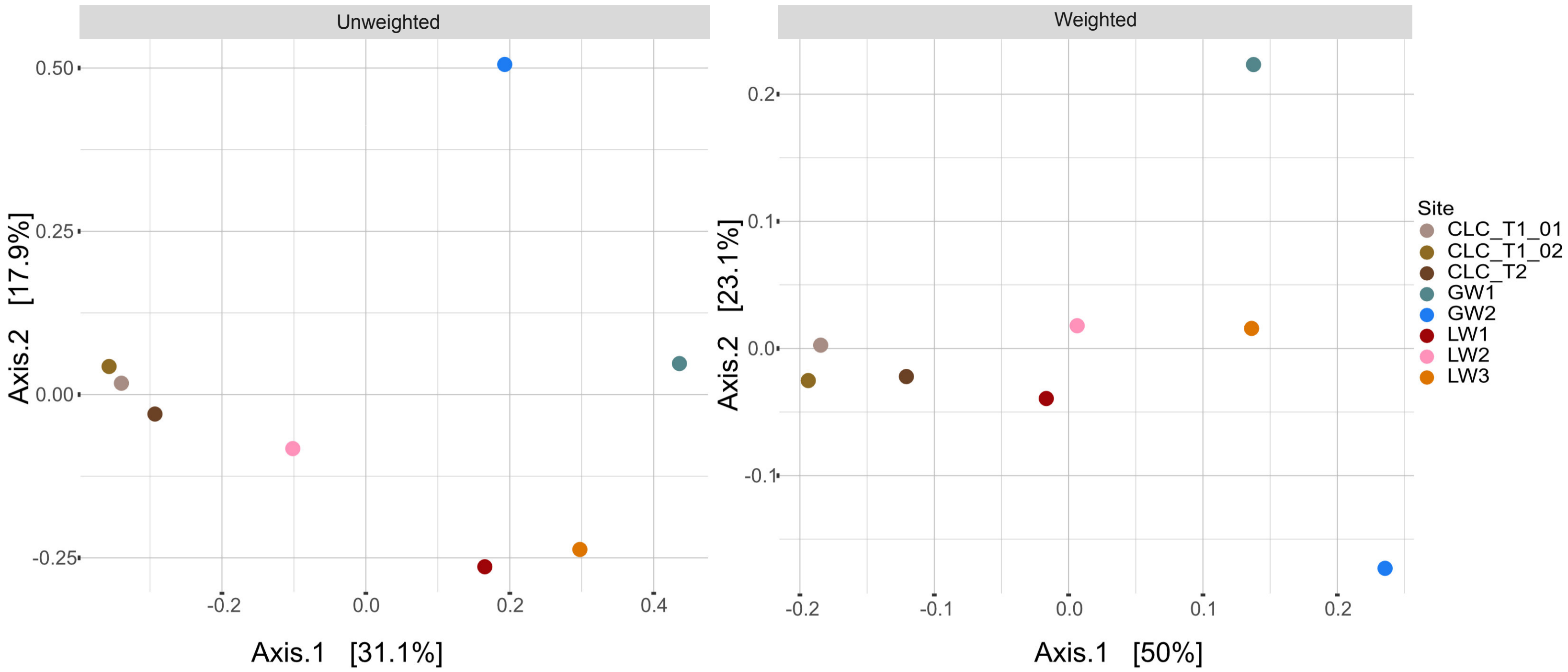

Supplemental Figure 2: PCoA using unweighted and weighted UniFrac distances based on 16S rRNA ASVs for all sites.

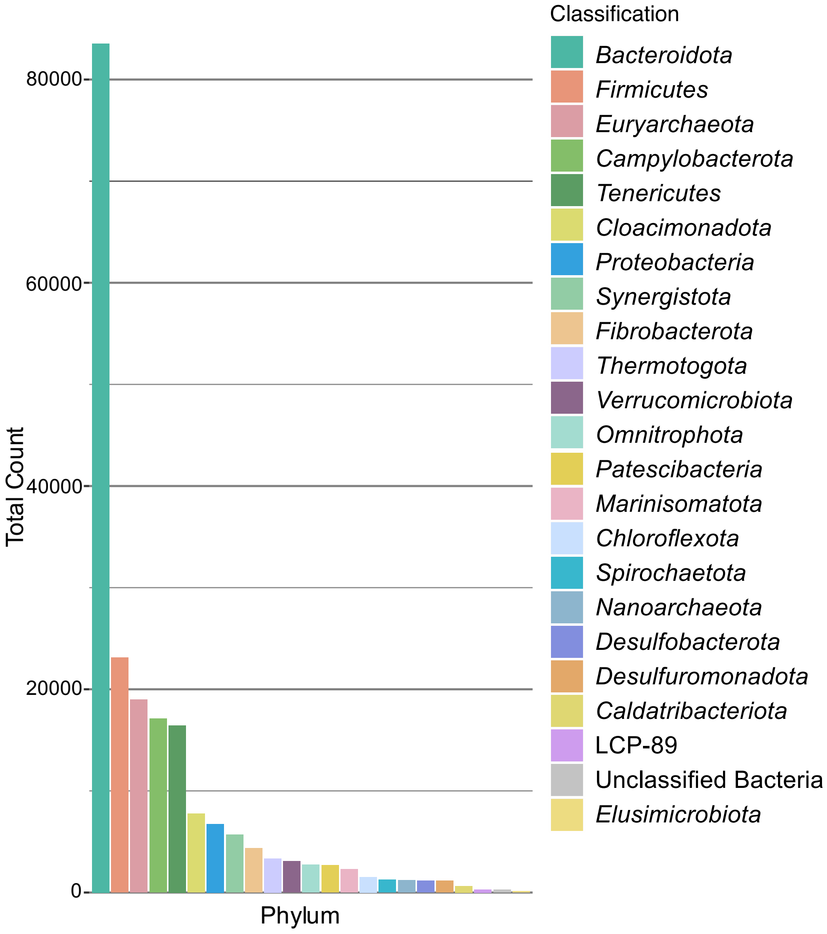

Supplemental Figure 3: 16S rRNA gene amplicon ASVs in five or more sites by total sequence count and phylum.

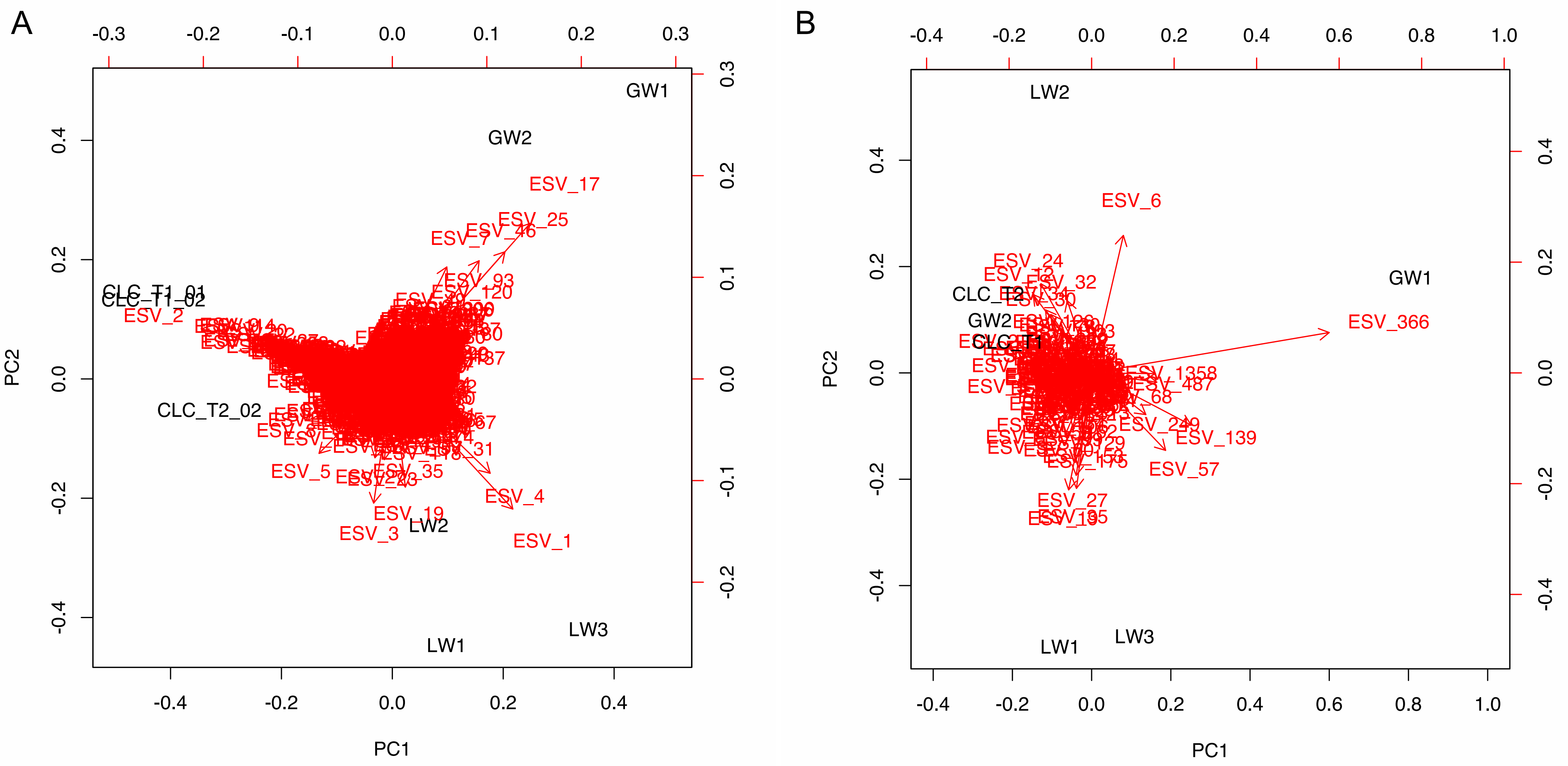

Supplemental Figure 4: Principal component analysis for (A) all 16S rRNA gene amplicon ASVs and (B) those present at five or more sites. ASV count data was Hellinger transformed.
