# Supplementary material for: Substrate-restricted methanogenesis and limited volatile organic compound degradation in highly diverse and heterogeneous municipal landfill microbial communities
Source: ISME Commun. 2022 Jul 13;2:58. doi: 10.1038/s43705-022-00141-4 (PMC9723747; doi:10.1038/s43705-022-00141-4)
Supplement: Supplementary file 2 — Supplemental data file 1 [file 43705_2022_141_MOESM2_ESM.rtf]

Sauk & Hug newick files for treesConcatenated ribosomal protein tree(((((((((((((((((((((((((((((((((((((((((((((Ga0172378_10013392:0.06995885887901387,Ga0172378_10026062:0.07087093237088293):0.03823480930208767[92],Ga0172378_10000622:0.12040431803937501):0.10299517114153645[100],Ga0172378_10031982:0.24512128627097063):0.033470706963159014[53],(Ga0172378_10000007:0.000001,Ga0172377_10000023:0.000001):0.17027416649971672[100]):0.20182711245468088[100],((Ga0172378_10008164:0.16700710509362082,Ga0172378_10009877:0.1581804511955367):0.05066487196854874[94],Ga0172378_10023392:0.16525899007609057):0.2056771254912233[100]):0.05175685812296171[42],((((((Ga0172381_10007869:0.028964524188412444,Ga0172378_10014771:0.013850797194109088):0.021116641284651294[97],Ga0172381_10034283:0.028628071735131932):0.013038752487763539[49],Ga0172381_10003582:0.0583904244666229):0.07961801437109184[100],Ga0172382_10000066:0.20603885492828766):0.10798719121493283[100],Ga0172377_10005041:0.25053199942560456):0.04438072615108224[41],(Ga0172377_10024285:0.23391960926268185,Ga0172382_10004647:0.19309172680824638):0.08539356115774321[100]):0.029816313128802285[22]):0.038803726397903304[54],((((Ga0172377_10007899:0.001700019776957884,Ga0172378_10008850:0.000956):0.000452[65],Ga0172381_10001651:0.000398):0.18574090872780502[100],Ga0172378_10003808:0.17158115543396057):0.05702598173975604[74],Ga0172382_10046844:0.3350609634146151):0.0881290524846241[97]):0.0732188410037149[100],((((((Ga0172378_10003832:0.000563,Ga0172377_10003059:0.000001):0.028779475875476024[100],Ga0172378_10020723:0.029698612037850403):0.01714785690429199[63],(Ga0172378_10045213:0.000001,Ga0172377_10024635:0.000001):0.057736697659175995[100]):0.024846198761800053[97],(Ga0172378_10023907:0.000001,Ga0172377_10029010:0.000001):0.10498328018037206[100]):0.1334832254174394[100],Ga0172378_10009932:0.22597823533551775):0.1578591882407019[100],((((Ga0172378_10000665:0.000731,Ga0172377_10010354:0.000001):0.0579019595410708[100],Ga0172378_10004898:0.05929988955249854):0.056680982165524973[100],Ga0172378_10005459:0.13279378079767845):0.04077680476725032[100],((Ga0172378_10000503:0.000411,Ga0172377_10000418:0.000430):0.2019121292628263[100],Ga0172378_10001160:0.14317097812915414):0.034497456572563756[89]):0.28452397374586846[100]):0.17498141525289945[100]):0.08937659020325261[100],(((Ga0172378_10022191:0.00948312310148225,Ga0172381_10013465:0.007486781373124973):0.012578279080634136[51],Ga0172377_10005223:0.018617356414594166):0.1769660026881099[100],Ga0172377_10009447:0.17468513082889814):0.11688171060832353[100]):0.11314312296948925[100],(((((Bacteria_Firmicutes:0.08779702441528103,Bacteria_Firmicutes:0.1059283796670365):0.21416916806911912[100],Bacteria_Firmicutes:0.10374329342977973):0.05863447138634337[100],Bacteria_Firmicutes:0.2511548499172389):0.03934345962708585[93],(Bacteria_Firmicutes:0.09931427113441371,Bacteria_Firmicutes:0.1230763700615034):0.036757218761694244[84]):0.032260947551768115[61],(((Bacteria_Firmicutes:0.04434627182858364,Bacteria_Firmicutes:0.08171091925698404):0.027257273876916788[75],Bacteria_Firmicutes:0.06950212873262851):0.05840510775967411[100],Bacteria_Firmicutes:0.18035996769875862):0.09962436117313933[100]):0.1160620525908449[100]):0.030213221728956352[48],(((((Bacteria_Firmicutes:0.000001,Bacteria_Firmicutes:0.000001):0.09534022427983979[100],Bacteria_Firmicutes:0.08753088541816822):0.0522115154946432[100],Bacteria_Firmicutes:0.09130662026052283):0.02939626298194664[99],Bacteria_Firmicutes:0.13157285354414006):0.04549862014066841[100],(Bacteria_Firmicutes:0.06650033634230201,Bacteria_Firmicutes:0.12088745815014956):0.039403557006580936[66]):0.170611191400027[100]):0.04179984483255739[95],(((Bacteria_Tenericutes:0.5133811740976939,Bacteria_Tenericutes:0.5814598452355697):0.08790765951289181[76],Bacteria_Tenericutes:0.5156687583959889):0.13290982965659204[100],((Bacteria_Tenericutes:0.1293772324312652,Bacteria_Tenericutes:0.15881434920119375):0.06647456027282095[100],Bacteria_Tenericutes:0.13419983946958025):0.23295409156641345[100]):0.10109512320508163[100]):0.04592978292230976[60],((((Ga0172377_10034961:0.03229043438630086,Ga0172378_10039617:0.02784699924638856):0.20238348458316358[100],Ga0172377_10025275:0.2064514207268271):0.05630893157863026[87],(Ga0172382_10006801:0.25783652488600595,Ga0172382_10003838:0.27374056515605005):0.03631808832420269[47]):0.04436322906114132[47],(Ga0172377_10004405:0.14610979608086438,Ga0172377_10002821:0.14347303876972584):0.2771003694004124[100]):0.1737699977946745[100]):0.04211478533290158[90],(((((((((Ga0172381_10043160:0.0019587113472745443,Ga0172380_10016158:0.004417583461642227):0.1966372991329317[100],Ga0172382_10001638:0.10265476743261281):0.022197372739137577[51],(Ga0172382_10001443:0.000001,Ga0172377_10009351:0.0018266002646614155):0.22272305104482193[100]):0.033764776866111124[94],(Ga0172381_10006092:0.000768,Ga0172377_10006082:0.0015308270234459798):0.15415500461869724[100]):0.029459111496429102[70],(((Ga0172378_10025812:0.000001,Ga0172377_10020019:0.000001):0.000603[58],Ga0172378_10007826:0.004766887812953868):0.31530963868444184[100],((Ga0172378_10003263:0.000001,Ga0172377_10000519:0.000001):0.19275015515017735[100],Ga0172382_10002354:0.1047107181338518):0.04115967388885844[86]):0.045422313753277876[67]):0.030326091064009297[47],((Ga0172381_10003581:0.0024503714759727124,Ga0172378_10018193:0.000001):0.17492038783501762[100],Ga0172377_10002543:0.3093944951183176):0.07476452748799822[100]):0.08726750636218261[100],Ga0172377_10000652:0.28434171941290076):0.07641945876292722[100],((((((Ga0172377_10000615:0.000627,Ga0172381_10020709:0.000001):0.000987[78],Ga0172380_10039600:0.000001):0.15181099418152444[100],Ga0172377_10044021:0.09447466980752717):0.033206392260357376[93],(Ga0172377_10022778:0.11165675572513356,Ga0172377_10048138:0.08993028474963616):0.03810679729618771[97]):0.045243782891009054[98],((Ga0172377_10004552:0.053229811860354026,Ga0172382_10056612:0.04194385388260091):0.02668075512235646[96],Ga0172382_10004349:0.05076141010446156):0.11154332701897385[100]):0.1362213992446617[100],(Bacteria_Tenericutes:0.1322345282148265,Bacteria_Tenericutes:0.14493481847836032):0.11731100639118797[100]):0.05220160018430153[100]):0.02586797244886707[76],((((((Ga0172381_10007580:0.0015340863319459608,Ga0172377_10037729:0.000856):0.06432352503921823[100],Ga0172382_10011354:0.06845161066309258):0.03082305398857965[99],(Ga0172382_10011582:0.1316780415427452,Ga0172382_10003866:0.04251790742556283):0.0185448345391932[89]):0.03945253639801649[97],(Ga0172378_10002944:0.000001,Ga0172377_10038329:0.000850):0.11286390958587544[100]):0.09842642577581229[100],(Bacteria_Tenericutes:0.366214033910194,Bacteria_Tenericutes:0.24079756283324638):0.0366579386833874[92]):0.06112614994002685[100],(((((Ga0172377_10030487:0.000001,Ga0172378_10000575:0.000789):0.000001[87],Ga0172382_10036507:0.000708):0.22734199316204684[100],((Ga0172382_10000390:0.000001,Ga0172377_10039017:0.0018288776697792386):0.000940[83],Ga0172377_10008355:0.000001):0.2432788589644712[100]):0.05110404183188333[100],Ga0172382_10010073:0.14034053299393934):0.07165076119498659[100],Ga0172382_10016986:0.15476666855781573):0.038759987295469145[96]):0.153338293629528[100]):0.05438168106126762[100]):0.048010125073099985[99],Bacteria_unclassified:0.20441053763186856):0.03187522594634995[51],Bacteria_Firmicutes:0.18306308372653124):0.09479774177490619[99],((Bacteria_Firmicutes:0.03914304694513904,Bacteria_Firmicutes:0.048591389642389515):0.04137767953129101[100],Bacteria_Firmicutes:0.11219940820762231):0.03084969859886222[98]):0.017715956430031987[22],((((((((((((Bacteria_Firmicutes:0.08127423010060308,Bacteria_Firmicutes:0.018641225254692806):0.026842391564574353[100],Bacteria_Firmicutes:0.1343074950211216):0.01885968727531928[99],(Bacteria_Firmicutes:0.1080366980808174,Bacteria_Firmicutes:0.1475134090689343):0.09299398993673735[100]):0.016317243299394324[33],Bacteria_Firmicutes:0.0873851755988091):0.04238004599433376[93],Bacteria_Firmicutes:0.0893447205857556):0.020674421449407898[44],(((Bacteria_Firmicutes:0.12114713457234849,Bacteria_Firmicutes:0.10952928956301955):0.08336598992379907[100],Bacteria_Firmicutes:0.2522880145243782):0.06980782399225394[100],(Bacteria_Firmicutes:0.11712719981921449,Bacteria_Firmicutes:0.06958757893006862):0.03125404911154828[100]):0.08188811394633566[100]):0.01944727698669979[46],(((((Bacteria_Firmicutes:0.10108447479450389,Bacteria_Firmicutes:0.06981203528201885):0.06693626137107511[100],Bacteria_Firmicutes:0.24565793966243987):0.057331073221544404[100],Bacteria_Firmicutes:0.10391192686920148):0.08162944884217183[100],Bacteria_Firmicutes:0.1533382392522893):0.05177015129341678[99],Bacteria_Firmicutes:0.31332274622722167):0.06599928163845759[95]):0.014419792966922529[53],(((((Bacteria_Firmicutes:0.000001,Bacteria_Firmicutes:0.000001):0.07905396031040945[100],Bacteria_Firmicutes:0.08341336078082717):0.034509542077173716[88],(Bacteria_Firmicutes:0.11780921738493744,Bacteria_Firmicutes:0.10545598594896433):0.03341238495035581[99]):0.08184972209104746[100],Bacteria_Firmicutes:0.24091525389979607):0.02713662460702615[68],((Bacteria_Firmicutes:0.1401043615203661,Bacteria_Firmicutes:0.10056308874807529):0.05876200616649152[100],Bacteria_Firmicutes:0.09352565182553496):0.03490088538484626[97]):0.029311743018094916[87]):0.07300939863154676[100],(Bacteria_Firmicutes:0.16722561295965965,Bacteria_Firmicutes:0.0986729099917123):0.037829427969941154[72]):0.03476511491726786[100],((((Bacteria_Firmicutes:0.08286331063744079,Bacteria_Firmicutes:0.10013802628417201):0.038028899461088006[84],Bacteria_Firmicutes:0.16807331084976518):0.09528242894350392[100],(Bacteria_Firmicutes:0.10930674737110868,Bacteria_Firmicutes:0.06792588201304905):0.044280960429775806[99]):0.04572200523534731[97],Bacteria_Firmicutes:0.20646223837054656):0.07346806023304708[100]):0.019617043418738156[47],(((((Bacteria_Firmicutes:0.05196866606000983,Bacteria_Firmicutes:0.027500118093091608):0.010195565581918142[69],Bacteria_Firmicutes:0.07687658376697293):0.02404877060305788[99],(Bacteria_Firmicutes:0.09650224513867167,Bacteria_Firmicutes:0.04050604771822108):0.015030415804022645[94]):0.025382960016659517[100],(Bacteria_Firmicutes:0.09782657042653575,Bacteria_Firmicutes:0.07870407550533587):0.025143953417636755[93]):0.019080072247936286[90],(Bacteria_Firmicutes:0.0954852527370269,Bacteria_Firmicutes:0.07507497852076073):0.032718813493018306[100]):0.05023640108586669[100]):0.03083656815504643[99],Bacteria_Firmicutes:0.1249285438318064):0.016415205144399092[38]):0.026107529074173907[82],Bacteria_Firmicutes:0.19733099789258324):0.015108104796982325[33],((((((((Bacteria_Firmicutes:0.04736150707887932,Bacteria_Firmicutes:0.07599370062811905):0.016739687019215133[69],Bacteria_Firmicutes:0.09680354466135022):0.016000524798453597[60],(Bacteria_Firmicutes:0.03032231424470755,Bacteria_Firmicutes:0.029896952732355597):0.052301406032181585[100]):0.028991595875085263[100],Bacteria_Firmicutes:0.09769345151219833):0.018805643364088187[53],((Bacteria_Firmicutes:0.14487516188576777,Bacteria_Firmicutes:0.06935413629981602):0.026990088833092507[49],Bacteria_Firmicutes:0.048461572079694815):0.014375454557919998[25]):0.015134563509056065[20],(((Bacteria_Firmicutes:0.13137074347991984,Bacteria_Firmicutes:0.05580159224765957):0.04508281098342337[100],Bacteria_Firmicutes:0.056097862518057706):0.026212231370108974[98],Bacteria_Firmicutes:0.10828923121829392):0.019878127291482972[70]):0.025239908953557322[29],(Bacteria_Firmicutes:0.07893541701853346,Bacteria_Firmicutes:0.08330042622880596):0.0901245500155552[100]):0.06576315109542197[100],((Bacteria_Firmicutes:0.14798049327128915,Bacteria_Firmicutes:0.14534164130546223):0.06823704314174694[100],Bacteria_Firmicutes:0.14776284355176017):0.03681737060529722[99]):0.028655032393217983[69]):0.019309203692805976[29],(Bacteria_Firmicutes:0.09814991230525161,Bacteria_Firmicutes:0.16795474219408302):0.07078305106159721[100]):0.013192690376140792[17],Bacteria_Firmicutes:0.0990050203416093):0.07550998835864187[99],(Bacteria_Firmicutes:0.12063204002098882,Bacteria_Firmicutes:0.15536570200987976):0.04937156102134477[99]):0.019399265619891803[32],(Bacteria_Firmicutes:0.10227669101189063,Bacteria_Firmicutes:0.2038968621900441):0.03750458725319916[56]):0.017335203625821194[29],((Bacteria_Firmicutes:0.1433536015249124,Bacteria_Firmicutes:0.1022738807687662):0.048972784388610524[99],Bacteria_Firmicutes:0.12740508417215946):0.08858587290194508[100]):0.014486659395675616[37],(((Bacteria_Firmicutes:0.1074610344035265,Bacteria_Firmicutes:0.04840974610166793):0.03541966861786516[100],Bacteria_Firmicutes:0.1306203175335141):0.026046454366551952[56],Bacteria_Firmicutes:0.09889442484856437):0.0951101451497034[100]):0.04291719920963688[90],Bacteria_Firmicutes:0.1848054063181639):0.041367659104296184[57],(Bacteria_Firmicutes:0.17072102190702543,Bacteria_Firmicutes:0.20548854780016512):0.05916061832437958[100]):0.09298656330208654[99],((Bacteria_Firmicutes:0.25054442430392365,Bacteria_Firmicutes_:0.35107077436013334):0.046543220966537735[80],Bacteria_Firmicutes:0.23023345606135237):0.031769058209377476[35]):0.027696769070315153[38],(((((((((Ga0172382_10021920:0.12812596479184934,Bacteria_Firmicutes:0.0747482225369911):0.09084139641492595[100],Bacteria_Firmicutes:0.12597732154016006):0.03771859124224575[92],Bacteria_Firmicutes:0.16549895268999526):0.04079636426416888[94],((Bacteria_Firmicutes:0.1332975543505004,Bacteria_Firmicutes:0.256575609758277):0.039038035540718496[65],Bacteria_Firmicutes:0.27926281105158957):0.02696955444387017[58]):0.02923386859964383[57],Bacteria_Firmicutes:0.2171326392332622):0.030774690771852686[98],Bacteria_Firmicutes:0.19209828305359355):0.02708941300953649[85],((Bacteria_Firmicutes:0.13623514709875062,Bacteria_Firmicutes:0.1330101182493637):0.09158370846125896[100],Bacteria_Firmicutes:0.27526605427814266):0.05173250027801446[96]):0.026996816921517564[51],((((((Ga0172378_10012438:0.186743222743889,Bacteria_Firmicutes:0.08473836294974335):0.027151231684551025[65],Bacteria_Firmicutes:0.1159695519761037):0.08289379333608737[100],(Bacteria_Firmicutes:0.14275596885585484,Bacteria_Firmicutes:0.09445663378778768):0.07045023977893417[100]):0.11694491601199619[100],(Bacteria_Firmicutes:0.18030996356575413,Ga0172378_10007709:0.3763458294381987):0.03990825456754221[53]):0.03170421040922955[65],(Bacteria_Firmicutes:0.12217028269354113,Bacteria_Firmicutes:0.2289668731893575):0.16066239474993305[100]):0.02021898969687319[38],((Bacteria_Firmicutes:0.2364994180808795,Ga0172377_10009484:0.261880947261397):0.05161952317397711[99],Bacteria_Firmicutes:0.26833595262283216):0.03719308913324326[55]):0.020146542545018242[29]):0.03739141412298386[98],((((((((Bacteria_Firmicutes:0.024233034868159198,Bacteria_Firmicutes:0.05073956797770274):0.02236163819886583[96],Bacteria_Firmicutes:0.13242904590893678):0.05836940421988501[100],Bacteria_Firmicutes:0.09890414122687075):0.02513078448679451[62],((Bacteria_Firmicutes:0.000429,Bacteria_Firmicutes:0.000001):0.06740600577115474[100],Ga0172378_10009370:0.07054785660393925):0.042424294287628506[100]):0.08910819294773953[100],(((Bacteria_Firmicutes:0.04788038558529939,Bacteria_Firmicutes:0.09391625222726274):0.12575756411253503[100],Bacteria_Firmicutes:0.14869161726860725):0.04103217063265241[76],(Bacteria_Firmicutes:0.12285124157756311,Bacteria_Firmicutes:0.08726846716540093):0.20834582836853688[100]):0.09988763555734526[100]):0.03242297298705443[40],(((((Bacteria_Firmicutes:0.1184520220029186,Ga0172377_10039300:0.07245421068759406):0.035466277660499035[99],Bacteria_Firmicutes:0.10629194970679823):0.07154808974481597[100],Bacteria_Firmicutes:0.12688265429416212):0.08428427877274869[100],(Bacteria_Firmicutes:0.12587185096509934,Bacteria_Firmicutes:0.12510236630953742):0.02104339970747704[38]):0.01692596303416094[33],((Bacteria_Firmicutes:0.0021676046401255533,Bacteria_Firmicutes:0.000001):0.12083803964624007[100],Bacteria_Firmicutes:0.11795801516811188):0.037987638614636055[99]):0.022345432118808795[47]):0.029068844725203213[31],Bacteria_Firmicutes:0.09553499770537899):0.041038613363854726[47],Ga0172379_10000459:0.25601904692065336):0.14238185931452296[100]):0.030856619763462767[65]):0.03311288073391516[31],(((((((((((((((((Bacteria_Firmicutes:0.0910323359973213,Bacteria_Firmicutes:0.14103459552319153):0.026812561583153816[87],Bacteria_Firmicutes:0.12999209541830536):0.07174080848069941[100],((Ga0172377_10003049:0.000001,Ga0172378_10000421:0.000851):0.21004265894553908[100],Bacteria_Firmicutes:0.1795205683449672):0.04584196903976068[81]):0.030225984149277352[67],(((Bacteria_Firmicutes:0.009436248173353867,Bacteria_Firmicutes:0.004234061875184381):0.07456932685819329[100],Bacteria_Firmicutes:0.1540046888246689):0.026690617580071407[80],(Bacteria_Firmicutes:0.06124548953121245,Bacteria_Firmicutes:0.11030010373235344):0.03603390869039602[100]):0.0376026727610399[86]):0.014243927043756877[46],(Bacteria_Firmicutes:0.11370952268663403,Bacteria_Bacteroidet:0.07572323056835417):0.05782567339557243[100]):0.014191417395538863[46],(((Bacteria_Firmicutes:0.053615479870973815,Bacteria_Firmicutes:0.06382476925066571):0.02212896719847146[93],Bacteria_Firmicutes:0.14049455688979773):0.023424751583517978[78],(Bacteria_Firmicutes:0.0811974171593266,Bacteria_Firmicutes:0.0351775447996463):0.04289703278401635[100]):0.021113434734248315[81]):0.03236457912669266[97],Bacteria_Firmicutes:0.14669886390434206):0.03231692610443915[94],Bacteria_Firmicutes:0.13411252264436335):0.089714717366566[100],(Bacteria_Firmicutes:0.16458625923667336,Bacteria_Firmicutes:0.11571672665243815):0.10924634352808571[100]):0.1418568156692328[100],(((((Ga0172377_10044035:0.000001,Ga0172377_10038354:0.000001):0.19030394410106233[100],(Ga0172378_10015010:0.08426063815860063,Ga0172377_10030764:0.099489423597527):0.12186222867399632[100]):0.2040120767709488[100],(Ga0172377_10001707:0.000001,Ga0172378_10012740:0.000001):0.31047671550685996[100]):0.08009927925175697[97],(((Ga0172381_10024890:0.3383732123701235,Ga0172378_10000057:0.265199491731932):0.09367438379980753[100],Ga0172381_10003306:0.31357450907996354):0.039328409870487135[56],Ga0172378_10020553:0.37617248124999136):0.03698849993792397[31]):0.09606372986348344[100],((((Ga0172382_10053850:0.0019477639259948276,Ga0172378_10025081:0.000001):0.0015089080798982302[63],Ga0172382_10044103:0.0028251486380828084):0.3388672337892005[100],(Ga0172377_10010794:0.21684080185721033,Ga0172378_10000085:0.2524614193983852):0.0381024027424135[68]):0.09221736033129391[100],((Ga0172377_10020765:0.001594137307559862,Ga0172378_10015713:0.000001):0.3697928377031068[100],Ga0172378_10022067:0.22194187893371753):0.06464406585459992[89]):0.040765315320386186[78]):0.14861878188313504[100]):0.04669677726971733[79],((((((((Ga0172378_10034915:0.2415631970564407,Ga0172378_10002033:0.15439150315995231):0.07767906908911293[99],Ga0172377_10005585:0.28072896406718506):0.026449908570409963[47],Ga0172378_10011178:0.21135344850671167):0.07184930993499394[98],(((Bacteria_Firmicutes:0.000001,Bacteria_Firmicutes:0.000436):0.10920586366139817[100],Bacteria_Firmicutes:0.17201690796586533):0.10545928832345819[100],Bacteria_Firmicutes:0.1723624789554279):0.06691650471638422[100]):0.027806228902667485[44],(((Ga0172377_10020375:0.21492748213213497,Bacteria_Firmicutes:0.13694700546943528):0.029883284714546487[59],(Ga0172381_10020435:0.10359742256949867,Bacteria_Firmicutes:0.12050219906628978):0.08845354863709431[100]):0.020752956195943195[23],(Bacteria_Firmicutes:0.0922546394019843,Bacteria_Firmicutes:0.21085441322792242):0.12786360797128316[100]):0.06284032934303907[98]):0.07946083757140254[100],Ga0172382_10004429:0.2148026247171737):0.04885047256214614[100],(Ga0172378_10026773:0.2605772691313608,Ga0172377_10038494:0.2605402251138047):0.13282442684732665[100]):0.03453468007136573[83],((((((Ga0172378_10005737:0.000001,Ga0172381_10028199:0.000817):0.17390241215563051[100],Ga0172381_10006112:0.16796408325480483):0.09649883335870557[100],(Ga0172382_10000329:0.15456026901483533,Ga0172377_10026555:0.1857790735293734):0.05531754772421582[98]):0.05996414057747401[97],(Ga0172382_10049418:0.07245663596761931,Ga0172377_10021884:0.000001):0.34555844647912126[81]):0.10996358946162177[100],(Bacteria_Firmicutes:0.044116038493373644,Bacteria_Firmicutes:0.08218849721253951):0.13523867294430092[100]):0.04175459333416143[80],((((Ga0172378_10037146:0.000001,Ga0172382_10042926:0.000001):0.000915[60],Ga0172377_10015775:0.000001):0.000880[74],Ga0172381_10026953:0.000001):0.0848986254977353[100],Ga0172381_10009006:0.13251194439094682):0.26663930172691996[100]):0.035188704618817734[85]):0.03236292546123343[73]):0.04085925923127309[87],(((((((((((Ga0172377_10036717:0.06826171068863651,Ga0172382_10000070:0.10802458390405567):0.019169478149340335[74],Ga0172382_10028140:0.06264026006648749):0.056322117085062295[100],Ga0172378_10031996:0.16499694774382245):0.026987483674687507[63],((Ga0172382_10040974:0.044737843651838194,Ga0172382_10015634:0.04537645498022602):0.10152206531520891[89],Bacteria_Firmicutes:0.08834598430881613):0.03993711920094922[47]):0.034828548420065[51],Bacteria_Firmicutes:0.20801999849983588):0.03500145136117183[46],Ga0172382_10005462:0.18996837670643796):0.15064425610756071[100],'Bacteria_Firmicutes:0.24701592649797854):0.0571636483754272[78],(((((Bacteria_Firmicutes:0.048007951647684344,Bacteria_Firmicutes:0.05208036212682554):0.04216424127208329[100],Bacteria_Firmicutes:0.042791550633833886):0.049318429908035366[100],Bacteria_Firmicutes:0.11643764897819775):0.10093286929250755[100],((Bacteria_Firmicutes:0.15489534916991277,Bacteria_Firmicutes:0.16059297202120915):0.035204762693606284[83],Bacteria_Firmicutes:0.23645153466247004):0.08761686265266677[100]):0.06570036930625633[100],(Bacteria_Firmicutes:0.09905383417987368,Bacteria_Firmicutes:0.12063412363993287):0.0856719979564935[100]):0.027856401436664235[61]):0.03657719078309052[78],((((((((Bacteria_Firmicutes:0.19985713537698402,Bacteria_Firmicutes:0.2755609917084638):0.06219900469416162[97],Bacteria_Firmicutes:0.2985795931227271):0.08109448818552867[100],(Bacteria_Firmicutes:0.1907395448321334,Bacteria_Firmicutes:0.1890468182098899):0.05351913508899386[96]):0.03946107545563171[74],Bacteria_Firmicutes:0.2806031365421169):0.07264286002718867[100],(Bacteria_Firmicutes:0.10678180322649933,Ga0172382_10020107:0.14229242730742842):0.043776628342981905[88]):0.05671101056515537[100],(Bacteria_Firmicutes:0.09129935711173065,Bacteria_Firmicutes:0.11008316467946289):0.046687624903241964[100]):0.03500359596781211[92],Bacteria_Firmicutes:0.14794040156930732):0.025199025354402327[77],((Ga0172382_10007313:0.000001,Ga0172377_10004579:0.000528):0.07512894064854425[100],'Bacteria_Firmicutes:0.06678227828969208):0.186382953649614[100]):0.042971347952329264[100]):0.03339815995618789[78],(((Bacteria_Firmicutes:0.03736205413103155,Ga0172378_10011394:0.03007631859628468):0.0867048027391304[100],(Bacteria_Firmicutes:0.04621314224830364,Bacteria_Firmicutes:0.1677436406138635):0.050994935407399744[93]):0.12210678004035636[100],Bacteria_Firmicutes:0.25670931101180194):0.12056230608798302[100]):0.03087525323951601[77],(((Ga0172382_10008686:0.02030372580821105,Ga0172382_10016011:0.0239727556189675):0.08906626744573964[100],Ga0172382_10032378:0.11120648201825256):0.13390354466657017[100],Ga0172377_10001455:0.25919911444488486):0.03664406852310176[66]):0.02376600009428076[54]):0.034312607870531586[69],(((((Bacteria_Firmicutes:0.1346458551231713,Ga0172378_10002647:0.17871417761647335):0.02933638708357833[74],(Bacteria_Firmicutes:0.11269147593872075,Bacteria_Firmicutes:0.08155633895508707):0.10027461544903549[100]):0.05455978106539927[100],Bacteria_Firmicutes:0.3491888334395812):0.08128097840308657[100],((Bacteria_Firmicutes:0.11190387322702255,Bacteria_Firmicutes:0.06988972151100503):0.031217398878394143[85],Bacteria_Firmicutes:0.09032900726910542):0.09704583694258462[100]):0.08728163898537211[100],((Bacteria_Firmicutes:0.1514544342856654,Ga0172377_10003125:0.24497024470255147):0.0587935756975968[97],Bacteria_Firmicutes:0.24676847374499422):0.045058627768816084[94]):0.024939578130351414[47]):0.027110717206078494[69],(((Bacteria_Firmicutes:0.03766539851000772,Bacteria_Firmicutes:0.05675116164461036):0.05978896141489187[100],Bacteria_Firmicutes:0.143364421079891):0.09036657901248679[100],Bacteria_Firmicutes:0.17563223931300298):0.08106929962212561[100]):0.02384770785289314[38],Bacteria_Firmicutes:0.29376056349026136):0.031489122891933174[94],((Bacteria_Firmicutes:0.03185205162015592,Bacteria_Firmicutes:0.05940717169151455):0.09046642602794419[100],Bacteria_Firmicutes:0.17037923438395763):0.11262413493789758[100]):0.045659070646896005[100],(((Bacteria_Firmicutes:0.25431006215699536,Bacteria_Firmicutes:0.37435091832353606):0.06171195636737847[79],Bacteria_Firmicutes:0.2861584751933508):0.03395111252778271[71],Ga0172377_10014216:0.43065015770934423):0.04099092533164406[70]):0.0170928889235773[5]):0.04004583831136177[37],(((Ga0172377_10011623:0.322725980324841,Ga0172377_10021580:0.31967067473610555):0.044260562562112415[31],Ga0172382_10000613:0.3299892256515946):0.05763215668087529[84],(Ga0172382_10008840:0.35762731723505947,Ga0172377_10028148:0.26573062700511674):0.06635959135678382[99]):0.04307177638934245[49]):0.028374089980892414[51],(((((Bacteria_Firmicutes:0.11222459591522549,Bacteria_Firmicutes:0.09452586563527321):0.07942390754611806[100],Bacteria_Firmicutes:0.16976195933049398):0.0689225145349397[92],Bacteria_Firmicutes:0.21142512076382847):0.09400965188266452[100],(Bacteria_Firmicutes:0.22961505101170987,Bacteria_Firmicutes:0.15299688498593067):0.08604909793711002[100]):0.11599781876194859[100],Ga0172382_10011007:0.45973237768639175):0.04517950896624168[44]):0.05002333254422453[45],(((((((((((((((((((((((((((((((((((Bacteria_Actinobacteria_Actinobacteria_Actinobacteridae_Actinomycetales_Corynebacterineae_Nocardiaceae_Nocardia_brasiliensis_ATCC_700358:0.059781222753352736,Bacteria_Actinobacteria_Actinobacteria_Actinobacteridae_Actinomycetales_Corynebacterineae_Nocardiaceae_Smaragdicoccus_niigatensis_DSM_44881:0.10968651839240762):0.027711423123180978[98],'Bacteria_Actinobacteria_Actinobacteria_Actinobacteridae_Actinomycetales_Corynebacterineae_Nocardiaceae_Rhodococcus_sp._BCP1':0.053319607634446875):0.024039423760862988[99],(Ga0172382_10006313:0.07532097070054578,Bacteria_Actinobacteria_Actinobacteria_Actinobacteridae_Actinomycetales_Corynebacterineae_Mycobacteriaceae_Mycobacterium_abscessus_bolletii_50594:0.04855475056071068):0.08786742171980633[100]):0.01803452632510094[82],((Bacteria_Actinobacteria_Actinobacteria_Corynebacteriales_Gordoniaceae_Gordonia_bronchialis_DSM_43247:0.017636139665606176,'Bacteria_Actinobacteria_Actinobacteria_Corynebacteriales_Gordoniaceae_Gordonia_sp._KTR9':0.018911134959414433):0.06541915400520582[100],Bacteria_Actinobacteria_Actinobacteria_Actinobacteridae_Actinomycetales_Corynebacterineae_Tsukamurellaceae_Tsukamurella_paurometabola_DSM_20162:0.11171395316465782):0.021494091542511917[97]):0.018601393613129513[68],((Bacteria_Actinobacteria_Actinobacteria_Actinobacteridae_Actinomycetales_Corynebacterineae_Corynebacteriaceae_Corynebacterium_argentoratense_DSM_44202:0.08438750174289433,Bacteria_Actinobacteria_Actinobacteria_Actinobacteridae_Bifidobacteriales_unclassified_Bifidobacteriales_Turicella_otitidis_ATCC_51513:0.16961097166335337):0.11911114793121547[100],Bacteria_Actinobacteria_Actinobacteria_Actinobacteridae_Actinomycetales_Corynebacterineae_Dietziaceae_Dietzia_cinnamea_P4_contig00104:0.12447312903037222):0.050551776527202996[100]):0.023458890436840907[81],Bacteria_Actinobacteria_Actinobacteria_Actinobacteridae_Actinomycetales_Corynebacterineae_Mycobacteriaceae_Amycolicicoccus_subflavus_DQS3_9A1:0.11236384770075514):0.04730153105983659[100],Bacteria_Actinobacteria_Actinobacteria_Actinobacteridae_Actinomycetales_Corynebacterineae_Segniliparaceae_Segniliparus_rotundus_DSM_44985:0.2048831234870181):0.04428749933126497[99],Bacteria_Actinobacteria_Actinobacteria_Actinobacteridae_Actinomycetales_Pseudonocardineae_Pseudonocardiaceae_Actinomycetospora_chiangmaiensis_DSM_45062:0.19635578136763598):0.03190348896058426[50],(((((((Bacteria_Actinobacteria_Actinobacteria_Actinobacteridae_Actinomycetales_Pseudonocardineae_Pseudonocardiaceae_Saccharomonospora_viridis_DSM_43017:0.06244431067519374,Bacteria_Actinobacteria_Actinobacteria_Actinobacteridae_Actinomycetales_Pseudonocardineae_Pseudonocardiaceae_Prauserella_rugosa_DSM_43194:0.07563101011007545):0.014977072917797596[58],Bacteria_Actinobacteria_Actinobacteria_Actinobacteridae_Actinomycetales_Pseudonocardineae_Pseudonocardiaceae_Thermocrispum_agreste_DSM_44070:0.11702638118690922):0.018300923194555896[96],Bacteria_Actinobacteria_Actinobacteria_Actinobacteridae_Actinomycetales_Pseudonocardineae_Pseudonocardiaceae_Amycolatopsis_mediterranei_RB:0.06964496404178933):0.04543128590018064[100],Bacteria_Actinobacteria_Actinobacteria_Actinobacteridae_Actinomycetales_Pseudonocardineae_Pseudonocardiaceae_Sciscionella_marina_DSM_45152:0.14916659216810446):0.023465457080719343[96],(Bacteria_Actinobacteria_Actinobacteria_Actinobacteridae_Actinomycetales_Actinopolysporineae_Actinopolysporaceae_Actinopolyspora_mortivallis_HS_1_DSM_44261:0.10960123016773204,Bacteria_Actinobacteria_Actinobacteria_Actinobacteridae_Actinomycetales_Pseudonocardineae_Pseudonocardiaceae_Saccharopolyspora_erythraea_NRRL_2338:0.05961061498071363):0.0859178425011673[100]):0.01814380939677518[70],(((Bacteria_Actinobacteria_Actinobacteria_Actinobacteridae_Actinomycetales_Pseudonocardineae_Pseudonocardiaceae_Actinokineospora_inagensis_DSM_44258:0.08149848105156243,Bacteria_Actinobacteria_Actinobacteria_Actinobacteridae_Actinomycetales_Pseudonocardineae_Pseudonocardiaceae_Kutzneria_albida_DSM_43870:0.07753714223497976):0.016070012186558458[42],(Bacteria_Actinobacteria_Actinobacteria_Actinobacteridae_Actinomycetales_Pseudonocardineae_Pseudonocardiaceae_Actinosynnema_mirum_DSM_43827:0.028261176385504072,Bacteria_Actinobacteria_Actinobacteria_Actinobacteridae_Actinomycetales_Pseudonocardineae_Pseudonocardiaceae_Saccharothrix_espanaensis_DSM_44229:0.021976811613560354):0.05440747565682402[100]):0.021386742160330208[82],Bacteria_Actinobacteria_Actinobacteria_Actinobacteridae_Actinomycetales_Pseudonocardineae_Pseudonocardiaceae_Actinoalloteichus_cyanogriseus_DSM_43889:0.11820474867449438):0.02087353676849313[56]):0.03129064497815426[92],Bacteria_Actinobacteria_Actinobacteria_Actinobacteridae_Actinomycetales_Pseudonocardineae_Pseudonocardiaceae_Pseudonocardia_dioxanivorans_CB1190:0.15304354093021422):0.02070148309893849[38]):0.03753597897201466[96],Bacteria_Actinobacteria_Actinobacteria_Actinobacteridae_Actinomycetales_Frankineae_Nakamurellaceae_Nakamurella_multipartita_DSM_44233:0.1821189288077325):0.03475565464715036[98],(Bacteria_Actinobacteria_Actinobacteria_Actinobacteridae_Actinomycetales_Frankineae_Geodermatophilaceae_Geodermatophilus_obscurus_DSM_43160:0.03601461576773879,Bacteria_Actinobacteria_Actinobacteria_Actinobacteridae_Actinomycetales_Frankineae_Geodermatophilaceae_Blastococcus_saxobsidens_DD2:0.04191425585138253):0.14255707834922848[100]):0.0262708440984456[76],((((((((Bacteria_Actinobacteria_Actinobacteria_Actinobacteridae_Actinomycetales_Micromonosporineae_Micromonosporaceae_Salinispora_arenicola_CNS_205:0.03744567554226208,Bacteria_Actinobacteria_Actinobacteria_Actinobacteridae_Actinomycetales_Micromonosporineae_Micromonosporaceae_Verrucosispora_maris_AB_18_032:0.02166548176854688):0.009257641808686046[51],'Bacteria_Actinobacteria_Actinobacteria_Actinobacteridae_Actinomycetales_Micromonosporineae_Micromonosporaceae_Micromonospora_sp._L5':0.016939893461192845):0.030249879533231994[100],Bacteria_Actinobacteria_Actinobacteria_Actinobacteridae_Actinomycetales_Micromonosporineae_Micromonosporaceae_Actinoplanes_missouriensis_NBRC_102363:0.07156729843563792):0.02243975836235146[100],(Bacteria_Actinobacteria_Actinobacteria_Actinobacteridae_Actinomycetales_Micromonosporineae_Micromonosporaceae_Hamadaea_tsunoensis_DSM_44101:0.07195917044626032,Bacteria_Actinobacteria_Actinobacteria_Actinobacteridae_Actinomycetales_Micromonosporineae_Micromonosporaceae_Catelliglobosispora_koreensis_DSM_44566:0.07873132519061432):0.036150749233398116[100]):0.027309781191546367[100],Bacteria_Actinobacteria_Actinobacteria_Actinobacteridae_Actinomycetales_Micromonosporineae_Micromonosporaceae_Micromonosporaceae_bacterium_URHE0070:0.10907732949094529):0.02062660493799573[87],Bacteria_Actinobacteria_Actinobacteria_Actinobacteridae_Actinomycetales_Micromonosporineae_Micromonosporaceae_Longispora_albida_DSM_44784:0.10651056690449767):0.05926296442026269[100],((Bacteria_Actinobacteria_Actinobacteria_Actinobacteridae_Actinomycetales_Glycomycineae_Glycomycetaceae_Haloglycomyces_albus_DSM_45210:0.13634874227269878,Bacteria_Actinobacteria_Actinobacteria_Actinobacteridae_Actinomycetales_Glycomycineae_Glycomycetaceae_Glycomyces_arizonensis_DSM_44726:0.07183315615393626):0.15398376363599953[100],Bacteria_Actinobacteria_Actinobacteria_Actinobacteridae_Actinomycetales_Glycomycineae_Glycomycetaceae_Stackebrandtia_nassauensis_DSM_44728:0.13871776265303692):0.08702699927440438[100]):0.05067588366348996[100],Bacteria_Actinobacteria_Actinobacteria_Actinobacteridae_Actinomycetales_Frankineae_Cryptosporangiaceae_Cryptosporangium_arvum_YU_629_21_DSM_44712:0.15741446519882496):0.029850846457216917[98]):0.019430773452680405[71],Bacteria_Actinobacteria_Actinobacteria_Actinobacteridae_Actinomycetales_Frankineae_Frankiaceae_Frankia_alni_ACN14a:0.19266395659923496):0.026225158172273666[54],(((((Bacteria_Actinobacteria_Actinobacteria_Actinobacteridae_Actinomycetales_Streptosporangineae_Nocardiopsaceae_Thermobifida_fusca_YX:0.09725806482389121,Bacteria_Actinobacteria_Actinobacteria_Actinobacteridae_Actinomycetales_Streptosporangineae_Nocardiopsaceae_Marinactinospora_thermotolerans_DSM_45154:0.046746415731116375):0.019386984870700452[60],Bacteria_Actinobacteria_Actinobacteria_Actinobacteridae_Actinomycetales_Streptosporangineae_Nocardiopsaceae_Nocardiopsis_alba_ATCC_BAA_2165:0.07067513304136686):0.09937518722075378[100],(Bacteria_Actinobacteria_Actinobacteria_Actinobacteridae_Actinomycetales_Streptosporangineae_Thermomonosporaceae_Actinomadura_madurae_LIID_AJ290:0.09011707467152297,Bacteria_Actinobacteria_Actinobacteria_Actinobacteridae_Actinomycetales_Streptosporangineae_Thermomonosporaceae_Thermomonospora_curvata_DSM_43183:0.08362713307779934):0.06267072948436647[100]):0.027263728088569472[73],((('Bacteria_Actinobacteria_Actinobacteria_Actinobacteridae_Actinomycetales_Streptosporangineae_Streptosporangiaceae_Microbispora_sp._ATCC_PTA_5024':0.03295556151064183,Bacteria_Actinobacteria_Actinobacteria_Actinobacteridae_Actinomycetales_Streptosporangineae_Streptosporangiaceae_Streptosporangium_roseum_DSM_43021:0.055567798777959254):0.019945839867813397[81],Bacteria_Actinobacteria_Actinobacteria_Actinobacteridae_Actinomycetales_Streptosporangineae_Streptosporangiaceae_Nonomuraea_coxensis_DSM_45129:0.049570021026267064):0.034109155484638[99],Bacteria_Actinobacteria_Actinobacteria_Actinobacteridae_Actinomycetales_Pseudonocardineae_Pseudonocardiaceae_Thermobispora_bispora_DSM_43833:0.06685267954512142):0.08191939521984981[100]):0.06570302826490027[100],Bacteria_Actinobacteria_Actinobacteria_Actinobacteridae_Actinomycetales_Frankineae_Acidothermaceae_Acidothermus_cellulolyticus_11B:0.20080132650182003):0.020163354074236306[19]):0.028763759285796553[31],(Ga0172380_10033476:0.26751570164507044,Bacteria_Actinobacteria_Actinobacteria_Actinobacteridae_Actinomycetales_Frankineae_Sporichthyaceae_Sporichthya_polymorpha_DSM_43042:0.1343777659419496):0.045014686823351635[71]):0.014970518723134152[13],((((Bacteria_Actinobacteria_Actinobacteria_Actinobacteridae_Actinomycetales_Streptomycineae_Streptomycetaceae_Streptomycetaceae_bacterium_MP113_05:0.0655248236905126,Bacteria_Actinobacteria_Actinobacteria_Actinobacteridae_Actinomycetales_Streptomycineae_Streptomycetaceae_Streptomyces_coelicolor_A32:0.03918727567258928):0.031348722417441355[100],(Bacteria_Actinobacteria_Actinobacteria_Actinobacteridae_Actinomycetales_Streptomycineae_Streptomycetaceae_Kitasatospora_setae_KM_6054_NBRC_14216:0.0728958310356469,Bacteria_Actinobacteria_Actinobacteria_Actinobacteridae_Actinomycetales_Streptomycineae_Streptomycetaceae_Streptacidiphilus_oryzae_TH49:0.056607110913383885):0.028692891764450668[100]):0.08654039496196297[100],(Bacteria_Actinobacteria_Actinobacteria_Actinobacteridae_Actinomycetales_Catenulisporineae_Catenulisporaceae_Catenulispora_acidiphila_DSM_44928:0.1180386838872538,Bacteria_Actinobacteria_Actinobacteria_Actinobacteridae_Actinomycetales_Catenulisporineae_Actinospicaceae_Actinospica_robiniae_DSM_44927:0.09600512364616431):0.0651251627093048[100]):0.03268299509630612[98],(((Ga0172380_10040890:0.025199794097845007,Ga0172380_10011589:0.03263557180752086):0.02173063539731901[67],Ga0172380_10017912:0.043589384784012086):0.09504151248703163[100],Ga0172380_10012249:0.17904549159657668):0.06685702747431232[100]):0.027214497829284312[68]):0.016705555848759435[37],((((((((((Bacteria_Actinobacteria_Actinobacteria_Actinobacteridae_Actinomycetales_Propionibacterineae_Propionibacteriaceae_Propionibacterium_acnes_KPA171202:0.000554,Bacteria_Actinobacteria_Actinobacteria_Propionibacteriales_Propionibacteriaceae_Propionibacterium_CG_Propioni_01:0.000001):0.1313746826205464[100],Bacteria_Actinobacteria_Actinobacteria_Actinobacteridae_Actinomycetales_Propionibacterineae_Propionibacteriaceae_Propionimicrobium_lymphophilum_DSM_4903:0.18073664421365576):0.038952465693984806[91],Bacteria_Actinobacteria_Actinobacteria_Actinobacteridae_Actinomycetales_Propionibacterineae_Propionibacteriaceae_Aestuariimicrobium_kwangyangense_DSM_21549:0.12321259771993853):0.04084871922746647[99],Ga0172382_10058975:0.15333090919762382):0.013207733672973898[56],Bacteria_Actinobacteria_Actinobacteria_Actinobacteridae_Actinomycetales_Propionibacterineae_Nocardioidaceae_Propionicicella_superfundia_DSM_22317:0.1568980038364023):0.044531793156838084[100],Bacteria_Actinobacteria_Actinobacteria_Actinobacteridae_Actinomycetales_Propionibacterineae_Propionibacteriaceae_Microlunatus_phosphovorus_NM_1:0.1183769212125223):0.08337753256231917[100],Bacteria_Actinobacteria_Actinobacteria_Actinobacteridae_Actinomycetales_Propionibacterineae_Nocardioidaceae_Kribbella_flavida_DSM_17836:0.13585641982681373):0.030426459053974675[85],((('Bacteria_Actinobacteria_Actinobacteria_Actinobacteridae_Actinomycetales_Propionibacterineae_Nocardioidaceae_Nocardioides_sp._JS614':0.05177333899427783,'Bacteria_Actinobacteria_Actinobacteria_Actinobacteridae_Actinomycetales_Propionibacterineae_Nocardioidaceae_Nocardioidaceae_bacterium_Broad_1_cont1.61':0.10401562621748273):0.03112195602245027[96],'Bacteria_Actinobacteria_Actinobacteria_Actinobacteridae_Actinomycetales_Propionibacterineae_Nocardioidaceae_Marmoricola_sp._URHB0036':0.08068467559253945):0.059492875489811414[100],Bacteria_Actinobacteria_Actinobacteria_Actinobacteridae_Actinomycetales_Propionibacterineae_Nocardioidaceae_Aeromicrobium_marinum_DSM_15272:0.1515179381911338):0.04328145198026334[99]):0.030453201460737045[92],Bacteria_Actinobacteria_Actinobacteria_Actinobacteridae_Actinomycetales_Propionibacterineae_Nocardioidaceae_Actinopolymorpha_alba_DSM_45243:0.18976443965465295):0.035063260708938504[85],Bacteria_Actinobacteria_Actinobacteria_Actinobacteridae_Actinomycetales_Pseudonocardineae_Pseudonocardiaceae_Jiangella_gansuensis_YIM_002_DSM_44835:0.1527404277948632):0.03612341787136497[92]):0.05624328711198601[43],Bacteria_Actinobacteria_Actinobacteria_Actinobacteridae_Actinomycetales_Kineosporiineae_Kineosporiaceae_Kineococcus_radiotolerans_SRS30216:0.170232828125505):0.02640314997229387[27],((((('Bacteria_Actinobacteria_Actinobacteria_Actinobacteridae_Actinomycetales_Micrococcineae_Intrasporangiaceae_Janibacter_sp._HTCC2649':0.0704976060358864,Bacteria_Actinobacteria_Actinobacteria_Actinobacteridae_Actinomycetales_Micrococcineae_Intrasporangiaceae_Tetrasphaera_elongata_DSM_14184:0.07219167610897692):0.04319429447553702[100],('Bacteria_Actinobacteria_Actinobacteria_Actinobacteridae_Actinomycetales_Micrococcineae_Intrasporangiaceae_Terracoccus_sp._273MFTsu3.1':0.030892010002594894,Bacteria_Actinobacteria_Actinobacteria_Actinobacteridae_Actinomycetales_Micrococcineae_Intrasporangiaceae_Intrasporangium_calvum_DSM_43043:0.02502719490709593):0.06127938283983392[100]):0.024750026146061366[77],Bacteria_Actinobacteria_Actinobacteria_Actinobacteridae_Actinomycetales_Micrococcineae_Intrasporangiaceae_Arsenicicoccus_bolidensis_DSM_15745:0.10366873261157705):0.0176770358127194[39],(((Bacteria_Actinobacteria_Actinobacteria_Actinobacteridae_Actinomycetales_Micrococcineae_Dermatophilaceae_Kineosphaera_limosa_NBRC_100340:0.07613446285059888,Bacteria_Actinobacteria_Actinobacteria_Actinobacteridae_Actinomycetales_Micrococcineae_Dermatophilaceae_Mobilicoccus_pelagius_NBRC_104925:0.09092592491819573):0.021006451219545585[80],Bacteria_Actinobacteria_Actinobacteria_Actinobacteridae_Actinomycetales_Micrococcineae_Dermatophilaceae_Dermatophilus_congolensis_DSM_44180:0.1234494669003463):0.020773901664705274[85],Bacteria_Actinobacteria_Actinobacteria_Actinobacteridae_Actinomycetales_Micrococcineae_Dermatophilaceae_Austwickia_chelonae_NBRC_105200:0.09062010852694247):0.03131970318028454[100]):0.017334593527270403[62],(((Bacteria_Actinobacteria_Actinobacteria_Actinobacteridae_Actinomycetales_Micrococcineae_Intrasporangiaceae_Serinicoccus_marinus_MCCC_1A05965:0.06573212925412086,Bacteria_Actinobacteria_Actinobacteria_Actinobacteridae_Actinomycetales_Micrococcineae_Intrasporangiaceae_Ornithinimicrobium_pekingense_DSM_21552:0.05223364407319808):0.08471673629405796[100],Bacteria_Actinobacteria_Actinobacteria_Actinobacteridae_Actinomycetales_Micrococcineae_Dermacoccaceae_Kytococcus_sedentarius_DSM_20547:0.13570916084552476):0.020418765527243643[78],(Bacteria_Actinobacteria_Actinobacteria_Actinobacteridae_Actinomycetales_Micrococcineae_Dermacoccaceae_Demetria_terragena_DSM_11295:0.09513396255032625,Bacteria_Actinobacteria_Actinobacteria_Actinobacteridae_Actinomycetales_Micrococcineae_Dermacoccaceae_Dermacoccus_nishinomiyaensis_M25:0.08124192698949173):0.025636400198339793[88]):0.0232296061881736[82]):0.046034172949054764[100]):0.019329934382794622[18],(((((((((((((Bacteria_Actinobacteria_Actinobacteria_Actinobacteridae_Actinomycetales_Micrococcineae_Microbacteriaceae_Glaciibacter_superstes_DSM_21135:0.034430874222397545,'Bacteria_Actinobacteria_Actinobacteria_Actinobacteridae_Actinomycetales_Micrococcineae_Microbacteriaceae_Cryobacterium_sp._MLB_32':0.06322186570411503):0.03302719174060309[100],('Bacteria_Actinobacteria_Actinobacteria_Actinobacteridae_Actinomycetales_Micrococcineae_Microbacteriaceae_Clavibacter_michiganensis_subsp._michiganensis_NCPPB_382':0.08922515520240815,Bacteria_Actinobacteria_Actinobacteria_Actinobacteridae_Actinomycetales_Micrococcineae_Microbacteriaceae_Salinibacterium_amurskyense_PAMC_21357:0.12099929658404118):0.02184357902082512[47]):0.016218935413562896[40],'Bacteria_Actinobacteria_Actinobacteria_Actinobacteridae_Actinomycetales_Micrococcineae_Microbacteriaceae_Herbiconiux_sp._YR403':0.07020882684477447):0.019144564789044693[43],('Bacteria_Actinobacteria_Actinobacteria_Actinobacteridae_Actinomycetales_Micrococcineae_Microbacteriaceae_Cryocola_sp._340MFSha3.1':0.022212673154737406,Bacteria_Actinobacteria_Actinobacteria_Actinobacteridae_Actinomycetales_Micrococcineae_Microbacteriaceae_Leifsonia_xyli_xyli_CTCB07:0.03266881553697276):0.04096914116301953[100]):0.010740133943584418[12],Bacteria_Actinobacteria_Actinobacteria_Actinobacteridae_Actinomycetales_Micrococcineae_Microbacteriaceae_Agromyces_subbeticus_DSM_16689:0.07357817709066117):0.015260478344755857[61],((Bacteria_Actinobacteria_Actinobacteria_Actinobacteridae_Actinomycetales_Micrococcineae_Microbacteriaceae_Curtobacterium_ginsengisoli_DSM_22003:0.08746954274236618,Bacteria_Actinobacteria_Actinobacteria_Actinobacteridae_Actinomycetales_Micrococcineae_Microbacteriaceae_Humibacter_albus_DSM_18994:0.05199430910372049):0.018411979879404683[51],Bacteria_Actinobacteria_Actinobacteria_Actinobacteridae_Actinomycetales_Micrococcineae_Microbacteriaceae_Rathayibacter_toxicus_DSM_7488:0.11259578263778902):0.013591481280159634[38]):0.01765897996447663[41],Bacteria_Actinobacteria_Actinobacteria_Actinobacteridae_Actinomycetales_Micrococcineae_Microbacteriaceae_Microbacterium_paraoxydans_DH1b:0.11362975627168925):0.01844385073148347[40],(Bacteria_Actinobacteria_Actinobacteria_Actinobacteridae_Actinomycetales_Micrococcineae_Microbacteriaceae_Curtobacterium_flaccumfaciens_UCD_AKU:0.02545957316291947,'Bacteria_Actinobacteria_Actinobacteria_Actinobacteridae_Actinomycetales_Micrococcineae_Microbacteriaceae_Curtobacterium_sp._9128':0.014021954722963414):0.09242238282739912[100]):0.018276618066501715[49],((Bacteria_Actinobacteria_Actinobacteria_Actinobacteridae_Actinomycetales_Micrococcineae_Microbacteriaceae_Leucobacter_chromiiresistens_JG_31:0.0471272229457349,Ga0172382_10001624:0.04192748526157564):0.08849865991086503[100],Bacteria_Actinobacteria_Actinobacteria_Actinobacteridae_Actinomycetales_Micrococcineae_Microbacteriaceae_Agrococcus_lahaulensis_DSM_17612:0.12672936221649378):0.03102428985680339[83]):0.01770778569487863[51],(Bacteria_Actinobacteria_Actinobacteria_Actinobacteridae_Actinomycetales_Micrococcineae_Microbacteriaceae_Gulosibacter_molinativorax_DSM_13485:0.07486304981462455,Bacteria_Actinobacteria_Actinobacteria_Micrococcales_Microbacteriaceae_Zimmermannella_faecalis_ATCC_13722:0.05396341983117914):0.12342438624992225[100]):0.04066673304480428[95],(Bacteria_Actinobacteria_marine_actinobacterium_IMCC13023:0.18013810523751816,'Bacteria_Actinobacteria_Actinobacteria_Micrococcales_Microbacteriaceae_Candidatus_Rhodoluna_lacicola_MWH_Ta8_Sequence_finished_Aug._2010':0.1326287154142003):0.06362220368071014[100]):0.06544869086925065[100],(((('Bacteria_Actinobacteria_Actinobacteria_Actinobacteridae_Actinomycetales_Micrococcineae_Micrococcaceae_Citricoccus_sp._CH26A':0.05448725214340655,Bacteria_Actinobacteria_Actinobacteria_Actinobacteridae_Actinomycetales_Micrococcineae_Micrococcaceae_Micrococcus_luteus_NCTC_2665:0.06244020593901434):0.035404042341227626[100],(Bacteria_Actinobacteria_Actinobacteria_Actinobacteridae_Actinomycetales_Micrococcineae_Yaniellaceae_Yaniella_halotolerans_DSM_15476:0.1646090687991153,Bacteria_Actinobacteria_Actinobacteria_Actinobacteridae_Actinomycetales_Micrococcineae_Micrococcaceae_Nesterenkonia_alba_DSM_19423:0.1325963587325396):0.03571682726423031[96]):0.04525967862674829[100],(Bacteria_Actinobacteria_Actinobacteria_Actinobacteridae_Actinomycetales_Micrococcineae_Micrococcaceae_Kocuria_rhizophila_DC2201:0.09049712624836914,Bacteria_Actinobacteria_Actinobacteria_Micrococcales_Micrococcaceae_Rothia_dentocariosa_ATCC_17931:0.11690668723797248):0.033661284592284346[99]):0.017232097053118345[62],((Bacteria_Actinobacteria_Actinobacteria_Actinobacteridae_Actinomycetales_Micrococcineae_Micrococcaceae_Renibacterium_salmoninarum_ATCC_33209:0.06035942670925509,Bacteria_Actinobacteria_Actinobacteria_Actinobacteridae_Actinomycetales_Micrococcineae_Micrococcaceae_Acaricomes_phytoseiuli_DSM_14247:0.060475293359389504):0.020005154364537425[50],Bacteria_Actinobacteria_Actinobacteria_Actinobacteridae_Actinomycetales_Micrococcineae_Micrococcaceae_Arthrobacter_phenanthrenivorans_Sphe3:0.0563003951657981):0.049597344230166485[100]):0.054214003212202044[100]):0.03326590206284541[100],Bacteria_Actinobacteria_Actinobacteria_Actinobacteridae_Actinomycetales_Micrococcineae_Brevibacteriaceae_Brevibacterium_linens_BL2:0.1822962983169063):0.03401956220519109[100]):0.019167229158600474[15],('Bacteria_Actinobacteria_Actinobacteria_Actinobacteridae_Actinomycetales_Micrococcineae_Dermabacteraceae_Dermabacter_sp._HFH0086':0.107597250675739,Bacteria_Actinobacteria_Actinobacteria_Actinobacteridae_Actinomycetales_Micrococcineae_Dermabacteraceae_Brachybacterium_faecium_DSM_4810:0.0904200270045794):0.10406890104529642[100]):0.02445052433508721[29],(((((((Bacteria_Actinobacteria_Actinobacteria_Actinobacteridae_Actinomycetales_Micrococcineae_Promicromonosporaceae_Xylanimonas_cellulosilytica_DSM_15894:0.04448780485438464,Bacteria_Actinobacteria_Actinobacteria_Actinobacteridae_Actinomycetales_Micrococcineae_Promicromonosporaceae_Promicromonospora_kroppenstedtii_RS16_DSM_19349:0.06536096821916848):0.011499499939384705[53],Bacteria_Actinobacteria_Actinobacteria_Actinobacteridae_Actinomycetales_Micrococcineae_Promicromonosporaceae_Isoptericola_variabilis_225:0.04567163158841758):0.018477102708046367[76],Bacteria_Actinobacteria_Actinobacteria_Actinobacteridae_Actinomycetales_Micrococcineae_Cellulomonadaceae_Paraoerskovia_marina_DSM_21750:0.06971298656564517):0.010236200484730062[71],Bacteria_Actinobacteria_Actinobacteria_Actinobacteridae_Actinomycetales_Micrococcineae_Promicromonosporaceae_Cellulosimicrobium_cellulans_J36_ICCI:0.041906914314814525):0.015311187214875233[99],(Bacteria_Actinobacteria_Actinobacteria_Actinobacteridae_Actinomycetales_Micrococcineae_Jonesiaceae_Jonesia_denitrificans_DSM_20603:0.12144472236277881,Bacteria_Actinobacteria_Actinobacteria_Actinobacteridae_Actinomycetales_Micrococcineae_Sanguibacteraceae_Sanguibacter_keddieii_DSM_10542:0.04778411275900485):0.02757723732051609[99]):0.01414706045945735[100],(Bacteria_Actinobacteria_Actinobacteria_Actinobacteridae_Actinomycetales_Micrococcineae_Cellulomonadaceae_Actinotalea_ferrariae_CF5_4:0.07389767209288367,Bacteria_Actinobacteria_Actinobacteria_Actinobacteridae_Actinomycetales_Micrococcineae_Cellulomonadaceae_Cellulomonas_flavigena_DSM_20109:0.09058887263395476):0.018458590758399396[87]):0.03702466015841521[100],((Bacteria_Actinobacteria_Actinobacteria_Actinobacteridae_Actinomycetales_Micrococcineae_unclassified_Micrococcineae_Ruania_albidiflava_DSM_18029:0.10542713941346671,Bacteria_Actinobacteria_Actinobacteria_Actinobacteridae_Actinomycetales_Micrococcineae_Beutenbergiaceae_Beutenbergia_cavernae_DSM_12333:0.09747550358344137):0.027615629685342036[94],'Bacteria_Actinobacteria_Actinobacteria_Actinobacteridae_Actinomycetales_Micrococcineae_Bogoriellaceae_Georgenia_sp._SUBG003':0.08885461099509273):0.021303334795975015[54]):0.0426081295144054[78]):0.04573127183681569[37],(Bacteria_Actinobacteria_Actinobacteria_Actinobacteridae_Actinomycetales_Actinomycineae_Actinomycetaceae_Actinomyces_naeslundii_Howell_279_ATCC_12104:0.000982,'Bacteria_Actinobacteria_Actinobacteria_Actinobacteridae_Actinomycetales_Actinomycineae_Actinomycetaceae_Actinomyces_sp._oral_taxon_170_str._F0386':0.007083043308770076):0.15699052659983348[100]):0.03374299268071379[31],((Bacteria_Actinobacteria_Actinobacteria_Actinobacteridae_Actinomycetales_Actinomycineae_Actinomycetaceae_Arcanobacterium_haemolyticum_DSM_20595:0.10355173625764102,Bacteria_Actinobacteria_Actinobacteria_Actinobacteridae_Actinomycetales_Actinomycineae_Actinomycetaceae_Trueperella_pyogenes_TP6375:0.1432581821891481):0.042342558530826135[79],Bacteria_Actinobacteria_Actinobacteria_Actinobacteridae_Actinomycetales_Actinomycineae_Actinomycetaceae_Actinobaculum_schaalii_CCUG_27420:0.15410880043519493):0.07851068883475598[100]):0.023113894300638815[26],(Bacteria_Actinobacteria_Actinobacteria_Actinobacteridae_Actinomycetales_Actinomycineae_Actinomycetaceae_MobiluncusFalcivibrio_group_Mobiluncus_curtisii_ATCC_43063:0.21540123584492976,Bacteria_Actinobacteria_Actinobacteria_Actinobacteridae_Actinomycetales_Actinomycineae_Actinomycetaceae_Varibaculum_cambriense_DSM_15806:0.21405847197820638):0.05797653813115833[97]):0.05098617061396871[37],((('Bacteria_Actinobacteria_Actinobacteria_Actinobacteridae_Bifidobacteriales_Bifidobacteriaceae_Scardovia_inopinata_F0304_genomic_scaffold_supercont1.1':0.05247863485033699,Bacteria_Actinobacteria_Actinobacteria_Actinobacteridae_Bifidobacteriales_Bifidobacteriaceae_Parascardovia_denticolens_DSM_10105_Contig1484:0.05446775829914996):0.042219739242249865[100],Bacteria_Actinobacteria_Actinobacteria_Actinobacteridae_Bifidobacteriales_Bifidobacteriaceae_Alloscardovia_omnicolens_DSM_21503:0.07583148543902984):0.025222213847938324[75],(Bacteria_Actinobacteria_Actinobacteria_Actinobacteridae_Bifidobacteriales_Bifidobacteriaceae_Gardnerella_vaginalis_409_05:0.06934804519148763,Bacteria_Actinobacteria_Actinobacteria_Actinobacteridae_Bifidobacteriales_Bifidobacteriaceae_Bifidobacterium_animalis_animalis_ATCC_25527:0.0813284786440649):0.0360437909082183[100]):0.24111220805860967[100]):0.17836940316789995[100],(((Ga0172382_10001311:0.21320855931491378,Bacteria_Actinobacteria_Actinobacteria_Acidimicrobidae_Acidimicrobiales_Acidimicrobineae_Acidimicrobiaceae_Ilumatobacter_coccineum_YM16_304:0.2939910798016694):0.04105490508916043[29],Bacteria_Actinobacteria_Actinobacteria_unclassified_Actinobacteria_Candidatus_Microthrix_parvicella_Bio17_1:0.2544595595251362):0.0945447950617293[100],(Bacteria_Actinobacteria_Actinobacteria_Acidimicrobidae_Acidimicrobiales_Acidimicrobineae_Acidimicrobiaceae_Acidimicrobium_ferrooxidans_DSM_10331:0.20276438089527327,Bacteria_Actinobacteria_Actinobacteria_Acidimicrobidae_Acidimicrobiales_Acidimicrobineae_Acidimicrobiaceae_Ferrimicrobium_acidiphilum_DSM_19497:0.2300901073933348):0.25285822616052256[100]):0.13110559972411817[100]):0.03469821295466513[26],Bacteria_Actinobacteria_Actinobacteria_Nitriliruptoridae_Nitriliruptorales_Nitriliruptoraceae_Nitriliruptor_alkaliphilus_DSM_45188:0.434891494339813):0.08916011500349352[100],(((((((Bacteria_Actinobacteria_Actinobacteria_Coriobacteridae_Coriobacteriales_Coriobacterineae_Coriobacteriaceae_Eggerthella_lenta_DSM_2243:0.04562101901173721,'Bacteria_Actinobacteria_Actinobacteria_Coriobacteridae_Coriobacteriales_Coriobacterineae_Coriobacteriaceae_Eggerthella_sp._YY7918':0.05323969292328412):0.011920056582702454[40],'Bacteria_Actinobacteria_Actinobacteria_Coriobacteridae_Coriobacteriales_Coriobacterineae_Coriobacteriaceae_Gordonibacter_pamelaeae_7_10_1_b_draft_genome.':0.044949088790412706):0.015627868242206855[58],Bacteria_Actinobacteria_Coriobacteriia_Coriobacteriales_Coriobacteriaceae_Senegalemassilia_anaerobia_JC110:0.09346120332297359):0.01909513323679901[79],((Bacteria_Actinobacteria_Actinobacteria_Coriobacteridae_Coriobacteriales_Coriobacterineae_Coriobacteriaceae_Enterorhabdus_mucosicola_DSM_19490:0.012817999293123883,Bacteria_Actinobacteria_Actinobacteria_Coriobacteridae_Coriobacteriales_Coriobacterineae_Coriobacteriaceae_Enterorhabdus_caecimuris_B7:0.0094150810459106):0.009715732167644209[75],Bacteria_Actinobacteria_Actinobacteria_Coriobacteridae_Coriobacteriales_Coriobacterineae_Coriobacteriaceae_Adlercreutzia_equolifaciens_DSM_19450:0.015853375323892305):0.12488818731262841[100]):0.0204645330303852[90],(Bacteria_Actinobacteria_Actinobacteria_Coriobacteridae_Coriobacteriales_Coriobacterineae_Coriobacteriaceae_Denitrobacterium_detoxificans_DSM_21843:0.06219954548515538,Bacteria_Actinobacteria_Actinobacteria_Coriobacteridae_Coriobacteriales_Coriobacterineae_Coriobacteriaceae_Cryptobacterium_curtum_DSM_15641:0.0933459525404885):0.07121081466171919[100]):0.03218645508997531[97],(Bacteria_Actinobacteria_Actinobacteria_Coriobacteridae_Coriobacteriales_Coriobacterineae_Coriobacteriaceae_Slackia_piriformis_YIT_12062:0.07542270417502372,Bacteria_Actinobacteria_Actinobacteria_Coriobacteridae_Coriobacteriales_Coriobacterineae_Coriobacteriaceae_Slackia_heliotrinireducens_DSM_20476:0.09533152839385783):0.04895018367759496[100]):0.10024058835151273[100],(((Bacteria_Actinobacteria_Actinobacteria_Coriobacteridae_Coriobacteriales_Coriobacterineae_Coriobacteriaceae_Atopobium_rimae_ATCC_49626:0.04940448665080854,Bacteria_Actinobacteria_Actinobacteria_Coriobacteridae_Coriobacteriales_Coriobacterineae_Coriobacteriaceae_Atopobium_parvulum_DSM_20469:0.050052403965181735):0.07960627768507722[100],(Bacteria_Actinobacteria_Actinobacteria_Coriobacteridae_Coriobacteriales_Coriobacterineae_Coriobacteriaceae_Olsenella_uli_DSM_7084:0.07216446861106052,'Bacteria_Actinobacteria_Actinobacteria_Coriobacteridae_Coriobacteriales_Coriobacterineae_Coriobacteriaceae_Olsenella_sp.OF0356':0.06853409813453437):0.048282532907956366[100]):0.09421747128233449[100],((Bacteria_Actinobacteria_Coriobacteriia_Coriobacteriales_Coriobacteriaceae_Enorma_massiliensis_phI:0.06118771358506869,Bacteria_Actinobacteria_Actinobacteria_Coriobacteridae_Coriobacteriales_Coriobacterineae_Coriobacteriaceae_Coriobacterium_glomerans_PW2_DSM_20642:0.11558049164454198):0.03174371789968777[90],Bacteria_Actinobacteria_Actinobacteria_Coriobacteridae_Coriobacteriales_Coriobacterineae_Coriobacteriaceae_Collinsella_intestinalis_DSM_13280:0.078470238192478):0.1151731880376583[100]):0.10192699370010594[100]):0.21512286691963922[100]):0.05599409702389124[69],(((('Bacteria_Actinobacteria_Actinobacteria_Rubrobacteridae_Solirubrobacterales_Patulibacteraceae_Patulibacter_sp._I11':0.08698428120155066,Ga0172382_10000044:0.09321405171476327):0.14553886898871848[100],(Bacteria_Actinobacteria_Actinobacteria_Rubrobacteridae_Solirubrobacterales_Solirubrobacteraceae_Solirubrobacter_soli_DSM_22325:0.2201732798156124,Bacteria_Actinobacteria_Actinobacteria_Rubrobacteridae_Solirubrobacterales_Conexibacteraceae_Conexibacter_woesei_DSM_14684:0.17963148127194062):0.046061999880450344[67]):0.12104562928500018[100],Bacteria_Actinobacteria_Actinobacteria_Rubrobacteridae_Thermoleophilales_Thermoleophilaceae_Thermoleophilum_album_ATCC_35263:0.3198111295639352):0.1861567332030285[100],Bacteria_Actinobacteria_Actinobacteria_Rubrobacteridae_Rubrobacterales_Rubrobacterineae_Rubrobacteraceae_Rubrobacter_xylanophilus_DSM_9941:0.43762099233222207):0.1118837655207856[100]):0.021566276352234404[47],Ga0172380_10051164:0.5758967275894298):0.03319352322787683[21],(Bacteria_Actinobacteria_Actinobacteria_Rubrobacteridae_Solirubrobacterales_CG_Solirubrobact_01:0.3104532310676418,Bacteria_CP_novel_BJP_IG2158_Novel_57_21:0.4851174066005277):0.05540098952738459[32]):0.07351187862032216[83],Ga0172382_10000341:0.6021618487988096):0.029000135908011426[8],(((((((((((((((((Ga0172382_10010591:0.000001,Ga0172381_10004567:0.000849):0.000001[75],Ga0172377_10001215:0.000830):0.000865[73],(Ga0172382_10012162:0.000845,Ga0172381_10006826:0.000001):0.000001[74]):0.23116433790876822[100],((Ga0172382_10017196:0.007336491590237415,Ga0172377_10045113:0.0019285624095322085):0.10922002045306067[100],Ga0172377_10015625:0.08075629005859719):0.2188527573172787[100]):0.05327858809322228[94],((((Ga0172382_10035374:0.000001,Ga0172382_10029739:0.002651553079417557):0.01172578113685363[96],(Ga0172380_10011587:0.000001,Bacteria_Chloroflexi_Dehalococcoidetes_Dehalococcoidales_Dehalococcoidaceae_Dehalococcoides_mccartyi_CBDB1:0.000001):0.020724974390468276[100]):0.26877716038355404[100],Bacteria_Chloroflexi_Dehalococcoidetes_Dehalogenimonas_lykanthroporepellens_BL_DC_9:0.2928706314769891):0.0851913187889708[100],Ga0172381_10035165:0.2546865714700868):0.031164938586027535[57]):0.03371892067266691[58],Bacteria_Chloroflexi_RBG_13_Chloroflexi_56_8b:0.2273727507867842):0.014915564046087848[21],Ga0172382_10055033:0.331447850654937):0.017118881975056066[22],(((((((((Bacteria_Chloroflexi_RBG_13_Chloroflexi_51_52:0.050452878569554827,Bacteria_Chloroflexi_RBG_13_Chloroflexi_51_18:0.06346660341601407):0.02630246821179183[98],Bacteria_Chloroflexi_RBG_16_Chloroflexi_50_11:0.05087266730325268):0.0433237057702307[100],Bacteria_Chloroflexi_RBG_13_Chloroflexi_52_12:0.07238899394771403):0.07224701014095336[100],(Bacteria_Chloroflexi_RBG_13_Chloroflexi_57_8:0.14174125352137867,Bacteria_Chloroflexi_RBG_16_Chloroflexi_56_11:0.12098284233986245):0.02687265517052051[63]):0.018628600395564465[63],(Bacteria_Chloroflexi_RBG_16_Chloroflexi_60_22:0.11515474061688868,Bacteria_Chloroflexi_RBG_16_Chloroflexi_58_8:0.09388724123182701):0.026283998929984342[74]):0.023373493977439264[71],Ga0172377_10029908:0.19719867232033916):0.053457322708887034[100],Bacteria_Chloroflexi_RBG_16_Chloroflexi_50_9:0.1525129551597737):0.08965608388906743[100],Bacteria_Chloroflexi_RBG_13_Chloroflexi_46_9:0.3041523212530435):0.033652811323128695[46],((Bacteria_Chloroflexi_RBG_16_Chloroflexi_51_9:0.17183144567665387,Bacteria_Chloroflexi_RBG_13_Chloroflexi_46_14:0.19251818134685328):0.061017967623472114[100],Bacteria_Chloroflexi_RBG_16_Chloroflexi_57_8:0.2760380322708107):0.039058810781063436[83]):0.014414412751002192[10]):0.08288463101510057[100],(((((Bacteria_Chloroflexi_RBG_13_Chloroflexi_48_17:0.06089847995379172,Bacteria_Chloroflexi_RBG_19FT_COMBO_Chloroflexi_47_15:0.03984823351215594):0.02501454891286281[100],(Bacteria_Chloroflexi_RBG_19FT_COMBO_Chloroflexi_48_23:0.06873025227900342,Bacteria_Chloroflexi_RBG_13_Chloroflexi_50_10:0.06572023713061581):0.01494734737320913[78]):0.10602559585998605[100],(Ga0172380_10001079:0.26157766274322247,Bacteria_Chloroflexi_RBG_16_Chloroflexi_48_7:0.3157751953992358):0.061511199610660316[99]):0.0882680645174232[100],(((Bacteria_Chloroflexi_Dehalococcoidia_CG_Dehalo_01:0.213646226013275,Bacteria_Chloroflexi_RBG_13_Chloroflexi_51_36:0.18983169190434257):0.041503948352779485[82],Ga0172380_10037441:0.3979577224617392):0.02862079395993522[40],Bacteria_Chloroflexi_Dehalococcoidia_CG_Dehalo_02:0.2365232528720691):0.09344905289639671[100]):0.08345350852109323[100],(Bacteria_Chloroflexi_RBG_13_Chloroflexi_54_8:0.18606832153102237,Bacteria_Chloroflexi_RBG_13_Chloroflexi_53_26:0.2426146277246466):0.15436144667250273[100]):0.037435264710314[81]):0.039502112589389515[80],((Bacteria_Chloroflexi_Dehalococcoidia_uncultured_DG_18:0.1571221575237307,Ga0172380_10006211:0.26932146115157174):0.11941058841817842[100],Bacteria_Chloroflexi_RBG_13_Chloroflexi_52_14:0.3007902238256075):0.0358256287863723[71]):0.044548946273301304[88],Bacteria_Chloroflexi_RBG_13_Chloroflexi_54_9:0.2624032898863278):0.0712735805211353[100],((((Bacteria_Chloroflexi_Dehalococcoidia_uncultured_DG_22:0.035668379074641265,Bacteria_Chloroflexi_Dehalococcoidia_uncultured_SMTZ_28_1:0.04119408863575158):0.03659834719499466[100],Bacteria_Chloroflexi_Dehalococcoidia_uncultured_SMTZ_28_2:0.07038407363148425):0.14485077750222786[100],Bacteria_Chloroflexi_RBG_16_Chloroflexi_64_32:0.295957885363503):0.09245257951978392[100],(((Ga0172380_10003525:0.15707339567275946,Ga0172380_10016628:0.11889713508062894):0.12687667509022793[100],Ga0172380_10004060:0.192214479369746):0.2694899842164724[100],Bacteria_Chloroflexi_RBG_16_Chloroflexi_68_14:0.28219096732289684):0.056559321607370094[96]):0.10247245507945113[100]):0.0971725998078834[100],('Bacteria_Chloroflexi_Ktedonobacteria_Thermogemmatisporales_Thermogemmatisporaceae_Thermogemmatispora_sp._PM5':0.14760329477879353,Bacteria_Chloroflexi_Ktedonobacteria_Ktedonobacterales_Ktedonobacteraceae_Ktedonobacter_racemifer_DSM_44963_Krac_Contig206:0.1925744402740066):0.29304852695648576[100]):0.036974759243014965[56],(((((((((((((((Bacteria_Chloroflexi_RBG_13_Chloroflexi_50_21:0.05084666863594389,Bacteria_Chloroflexi_RBG_16_Chloroflexi_54_11:0.04211044586262114):0.00838946231515747[39],Bacteria_Chloroflexi_RBG_16_Chloroflexi_47_49:0.03743099690255747):0.0096300851875184[61],Bacteria_Chloroflexi_RBG_19FT_COMBO_Chloroflexi_49_13:0.03370627963137007):0.012643490022121284[78],Bacteria_Chloroflexi_RBG_19FT_COMBO_Chloroflexi_47_9:0.03829522645724559):0.1106065277841708[100],Bacteria_Chloroflexi_RBG_19FT_COMBO_Chloroflexi_56_12:0.12799999892105474):0.07706790605623137[100],(((Bacteria_Chloroflexi_RBG_16_Chloroflexi_57_11:0.12140582339094586,Bacteria_Chloroflexi_RBG_16_Chloroflexi_54_18:0.14262263564992939):0.04860194115807914[100],Bacteria_Chloroflexi_RBG_16_Chloroflexi_52_11:0.15545878388276746):0.02061448536385324[55],Bacteria_Chloroflexi_RBG_19FT_COMBO_Chloroflexi_55_16:0.1602824723607963):0.022405664634557176[89]):0.044266228561789855[100],((((Bacteria_Chloroflexi_RBG_16_Chloroflexi_51_16:0.2177216677653,Bacteria_Chloroflexi_RIFOXYC12_FULL_Chloroflexi_59_14:0.12225099230086789):0.05752559828419024[100],Bacteria_Chloroflexi_Anaerolinea_Anaerolineales_Anaerolineaceae_CG_Anaero_03:0.16540502244788025):0.03807839321273265[96],(Bacteria_Chloroflexi_GWC2_Chloroflexi_49_37:0.14885955480293944,Bacteria_Chloroflexi_Anaerolineae_CG_Anaero_01:0.10333308159577159):0.045885737735627874[100]):0.04519340022052898[100],(Bacteria_Chloroflexi_GWB2_Chloroflexi_54_36:0.1941467595347075,Bacteria_Chloroflexi_Anaerolineae_Anaerolineales_Anaerolineaceae_Anaerolinea_thermophila_UNI_1:0.13529844861422857):0.06524666390312062[99]):0.037952404811795404[90]):0.043576022347196375[85],Ga0172382_10000963:0.35539205670741447):0.061046166971672644[99],((Bacteria_Chloroflexi_Anaerolineae_uncultured_SMTZ_63:0.15824944668927943,Bacteria_Chloroflexi_RBG_16_Chloroflexi_48_8:0.17304385347294637):0.08642217572998323[100],(Bacteria_Chloroflexi_RBG_13_Chloroflexi_68_17:0.12977236950697701,Bacteria_Chloroflexi_RBG_13_Chloroflexi_66_10:0.15361710329384115):0.04777349985112034[98]):0.0931737262233816[100]):0.024545308481662254[58],((Bacteria_Chloroflexi_RBG_13_Chloroflexi_60_9:0.20304708996789955,Bacteria_Chloroflexi_RBG_16_Chloroflexi_64_43:0.15456178678577093):0.1447041463359824[100],Bacteria_Chloroflexi_RBG_16_Chloroflexi_63_12:0.21406303039029462):0.06460946419922964[96]):0.11433443556754641[100],Bacteria_Chloroflexi_Anaerolineae_uncultured_SG8_19:0.35600885560692763):0.05015910745932883[95],'Bacteria_Chloroflexi_Thermoflexia_Thermoflexales_Thermoflexaceae_Bacterium_sp._JAD2_Draft_Genome':0.35746542461499065):0.05164145797245112[96],(Bacteria_Chloroflexi_Caldilineae_Caldilineales_Caldilineaceae_Caldilinea_aerophila_STL_6_O1_DSM_14535:0.3254697016396051,Bacteria_Chloroflexi_Anaerolineae_CG_Anaero_02:0.30333060134091516):0.08088460500836092[99]):0.044806000664042056[86],((Bacteria_Chloroflexi_RBG_16_Chloroflexi_56_8:0.375199721853412,Bacteria_Chloroflexi_RBG_16_Chloroflexi_57_9:0.23066478389142286):0.06362367269541958[96],(Bacteria_Chloroflexi_RBG_13_Chloroflexi_56_8:0.34240289486367326,Bacteria_Chloroflexi_Anaerolineae_uncultured_SMTZ_84:0.27339771228688603):0.06854182237399531[97]):0.05410674506686153[100]):0.09396536643358377[100],((((((Bacteria_Chloroflexi_Chloroflexi_Chloroflexales_Chloroflexaceae_Chloroflexus_aurantiacus_J_10_fl:0.09722136176560658,Bacteria_Chloroflexi_Chloroflexi_Chloroflexales_Oscillochloridaceae_Oscillochloris_trichoides_DG6_OSCTcontig_003:0.1290107922615391):0.06619982669101088[100],Bacteria_Chloroflexi_unclassified_Chloroflexi_Candidatus_Chlorothrix_halophila:0.22833878226010507):0.0321358490458028[90],Bacteria_Chloroflexi_Chloroflexi_Chloroflexales_Chloroflexaceae_Roseiflexus_castenholzii_DSM_13941:0.18661583257548342):0.05829006298409212[100],(Bacteria_Chloroflexi_Chloroflexi_Herpetosiphonales_Herpetosiphonaceae_Herpetosiphon_aurantiacus_DSM_785:0.2806942865441977,Bacteria_bacterium_JKG1:0.20407135910684415):0.051184591042044314[77]):0.15342202946712558[100],((Bacteria_Chloroflexi_Thermomicrobia_Sphaerobacteridae_Sphaerobacterales_Sphaerobacterineae_Sphaerobacteraceae_Nitrolancetus_hollandicus_Lb:0.16802705358781855,Bacteria_Chloroflexi_Thermomicrobia_Sphaerobacteridae_Sphaerobacterales_Sphaerobacterineae_Sphaerobacteraceae_Sphaerobacter_thermophilus_DSM_20745_1:0.08529806887063174):0.09007014416057446[100],Bacteria_Chloroflexi_Thermomicrobia_Thermomicrobiales_Thermomicrobiaceae_Thermomicrobium_roseum_DSM_5159:0.22631386719837776):0.17213586936885727[100]):0.0380336485678221[40],Bacteria_unclassified_Bacteria_Thermobaculum_terrenum_ATCC_BAA_798_1:0.361386743087444):0.06465735567949871[99]):0.038415666623246914[80]):0.03181064867637495[75],((((Bacteria_RIF_CHLX_RBG_16_RIF_CHLX_69_14:0.09276147199200624,Bacteria_RIF_CHLX_RBG_16_RIF_CHLX_70_13:0.12398103828552776):0.03644754751727053[95],Bacteria_RIF_CHLX_RBG_16_RIF_CHLX_72_14:0.1380658972256792):0.07593337572467274[100],Bacteria_RIF_CHLX_GWC2_RIF_CHLX_73_18:0.18759010723783298):0.2667879282240242[100],Bacteria_Chloroflexi_RIFCSPLOWO2_12_FULL_RIF_CHLX_71_12:0.5643361160232172):0.12775299488986214[100]):0.06160704930176397[100],((Bacteria_Armatimonadetes_13_1_40CM_Armatimonadetes_64_14:0.21622892595544219,Bacteria_Armatimonadetes_RBG_19FT_COMBO_Armatimonadetes_69_19:0.15919329539617522):0.1009305847730495[100],(Bacteria_CP_Armatimonadetes_CSP1_3:0.21765144288295835,Bacteria_Armatimonadetes_RBG_16_Armatimonadetes_67_12:0.26464593829834504):0.0510498548710574[89]):0.26068822194258257[100]):0.03331511628427597[63],((((Bacteria_Armatimonadetes_Chthonomonadetes_Chthonomonadales_Chthonomonadaceae_Chthonomonas_calidirosea_T49_EMBL_Bank_version_circular_IHQD:0.2339873006095985,Bacteria_Armatimonadetes_Armatimonadetes_bacterium_JGI_0000077_K19_TAbiofilm_001_240:0.295675859012023):0.11714068688601831[100],Ga0172377_10010803:0.36961276839505697):0.05918649341335103[85],Bacteria_Armatimonadetes_Fimbriimonas_ginsengisoli_Gsoil_348:0.5578306523059178):0.10432874107811951[100],((Bacteria_Armatimonadetes_CG_Arma_01:0.41830780678020574,Bacteria_Armatimonadetes_CG_Arma_02:0.37737125557607465):0.1235302305319701[100],Bacteria_KD3_62_uncultured_DG_56:0.5011216179607958):0.09129697712823104[100]):0.06732591918043251[79]):0.04205603018037296[27]):0.02999927926614987[9],(((((((((((((((((((((Bacteria_Cyanobacteria_Nostocales_Nostocaceae_Raphidiopsis_brookii_D9_D9_7:0.012376613457231844,Bacteria_Cyanobacteria_Nostocales_Nostocaceae_Cylindrospermopsis_raciborskii_509_upload_fresh_set:0.001008912886840907):0.0635137517832649[100],(Bacteria_Cyanobacteria_Nostocales_Nostocaceae_Aphanizomenon_flos_aquae_NIES_81:0.021070818184532936,Bacteria_Cyanobacteria_Nostocales_Nostocaceae_Anabaena_circinalis_AWQC131C_Submitted_file_with_automatic_translation_by_Kostas:0.018810852846971482):0.03932250320987407[100]):0.0074798173907346666[40],(Bacteria_Cyanobacteria_Nostocales_Nostocaceae_Anabaena_cylindrica_PCC_7122:0.030562261466401175,Bacteria_Cyanobacteria_Nostocales_Nostocaceae_Nostoc_azollae:0.023051627213023007):0.010129201399308396[60]):0.008577450797702113[63],(('Bacteria_Cyanobacteria_Nostocales_Microchaetaceae_Microchaete_sp._PCC_7126':0.05010555833855701,Bacteria_Cyanobacteria_Nostocales_Microchaetaceae_Fremyella_diplosiphon_UTEX_481:0.03298746693680066):0.010027469839845704[72],Bacteria_Cyanobacteria_Nostocales_Nostocaceae_Cylindrospermum_stagnale_PCC_7417:0.045546478668618384):0.0075288861983597855[40]):0.012306204910587759[74],Bacteria_Cyanobacteria_Nostocales_Nostocaceae_Nodularia_spumigena_CCY9414:0.0749277467025653):0.012522847073507481[69],'Bacteria_Cyanobacteria_Nostocales_Nostocaceae_Nostoc_sp._PCC_7524':0.058141945407189866):0.022590815990693347[100],'Bacteria_Cyanobacteria_Nostocales_Rivulariaceae_Calothrix_sp._PCC_6303':0.12149499415372933):0.012489202199511329[40],((Bacteria_Cyanobacteria_Stigonematales_Chlorogloeposis_fritschii_PCC_6912_CLC_Draft_2:0.03766515509746782,Bacteria_Cyanobacteria_Stigonematales_Fischerella_muscicola_SAG_1427_1:0.04657515536893797):0.01869263015999234[97],Bacteria_Cyanobacteria_Stigonematales_Mastigocladopsis_repens_MORA_PCC_10914:0.06231983879241865):0.014633008729576957[82]):0.011303937137291609[35],Bacteria_Cyanobacteria_Nostocales_Nostocaceae_Richelia_intracellularis_HH01:0.13259543838555077):0.02002400405655269[30],'Bacteria_Cyanobacteria_Nostocales_Rivulariaceae_Rivularia_sp._PCC_7116':0.13888365265415548):0.04007083116653121[100],Bacteria_Cyanobacteria_Pleurocapsales_Chroococcidiopsis_thermalis_PCC_7203:0.12087208085055234):0.04633715096155511[100],(Bacteria_Cyanobacteria_Oscillatoriales_Crinalium_epipsammum_PCC_9333:0.07801930102640187,'Bacteria_Cyanobacteria_Chroococcales_Chamaesiphon_sp._PCC_6605':0.19193705098782976):0.04061419097110708[99]):0.017846452526363965[82],((((((((Bacteria_Cyanobacteria_Chroococcales_Crocosphaera_watsonii_WH_8501:0.0691786907907912,Bacteria_Cyanobacteria_Chroococcales_Cyanobacterium_UCYN_A:0.12245036354209438):0.06794038812457082[100],'Bacteria_Cyanobacteria_Chroococcales_Synechocystis_sp._PCC_6803':0.15242847574626284):0.024140796894367877[55],'Bacteria_Cyanobacteria_Pleurocapsales_Pleurocapsa_sp._PCC_7327':0.09840007645923432):0.026728539725133782[85],(Bacteria_Cyanobacteria_Prochlorales_Prochloraceae_Prochloron_didemni_P3_Solomon:0.127943945472198,'Bacteria_Cyanobacteria_Chroococcales_Gloeocapsa_sp._PCC_73106':0.1796284820001648):0.03211544034001257[74]):0.01113097885498604[51],((('Bacteria_Cyanobacteria_Pleurocapsales_Xenococcus_sp._PCC_7305':0.12816026650351908,'Bacteria_Cyanobacteria_Pleurocapsales_Myxosarcina_sp._GI1':0.12248901028912318):0.034587723737191656[96],Bacteria_Cyanobacteria_Pleurocapsales_Stanieria_cyanosphaera_PCC_7437:0.07517887525499667):0.04309374242348074[100],('Bacteria_Cyanobacteria_Chroococcales_Synechocystis_sp._PCC_6308':0.07214005380302879,Bacteria_Cyanobacteria_Chroococcales_Cyanobacterium_stanieri_PCC_7202:0.060131116909705185):0.14595954499126984[100]):0.03169597474476449[65]):0.02564098372547452[76],(('Bacteria_Cyanobacteria_Chroococcales_Halothece_cluster_Halothece_sp._PCC_7418':0.04343839132371263,Bacteria_Cyanobacteria_Chroococcales_Dactylococcopsis_salina_PCC_8305:0.04554981125812807):0.11864049711784919[100],Bacteria_Cyanobacteria_Chroococcales_Rubidibacter_lacunae_KORDI_51_2_k51_img_annotated_130412:0.19405122796185514):0.08245934991745862[100]):0.015775650794140006[61],Bacteria_Cyanobacteria_Oscillatoriophycideae_Oscillatoriales_Spirulina_major_PCC_6313:0.18319641552192367):0.038984788265171044[100],(('Bacteria_Cyanobacteria_Oscillatoriales_Microcoleus_sp._PCC_7113':0.08480133282491753,Bacteria_Cyanobacteria_Oscillatoriales_Lyngbya_majuscula_3L:0.10954001998765017):0.020686705321945276[49],Bacteria_Cyanobacteria_Oscillatoriales_Microcoleus_chthonoplastes_PCC_7420_scf_1103659003820_genomic_scaffold:0.10054358523216855):0.031977966002044944[100]):0.021107154614639967[84]):0.018006446563191947[74],((((Bacteria_Cyanobacteria_Oscillatoriales_Arthrospira_platensis_NIES_39:0.08491839822072089,'Bacteria_Cyanobacteria_Oscillatoriales_Lyngbya_sp._PCC_8106_unfinished_sequence':0.11095179549107259):0.019246160318773242[52],Bacteria_Cyanobacteria_Oscillatoriales_Planktothrix_agardhii_NIVA_CYA_34:0.11717599745637308):0.028941121523729407[99],Bacteria_Cyanobacteria_Oscillatoriales_Trichodesmium_erythraeum_IMS101:0.17400272661856953):0.020179487698492427[54],('Bacteria_Cyanobacteria_Oscillatoriales_Oscillatoria_sp._PCC_6506':0.06878963746292266,'Bacteria_Cyanobacteria_Oscillatoriales_Oscillatoria_sp._PCC_7112':0.08642342899979027):0.05718796096102752[100]):0.04143925913341473[100]):0.022624140222046396[85],((((('Bacteria_Cyanobacteria_Prochlorales_Prochlorococcaceae_Prochlorococcus_marinus_str._MIT_9301':0.21697775053982715,'Bacteria_Cyanobacteria_Chroococcales_Synechococcus_sp._CC9902':0.08868880912865151):0.05601799355240411[96],Bacteria_Cyanobacteria_Chroococcales_Cyanobium_gracile_PCC_6307:0.07262223591365125):0.2722981796694359[100],Bacteria_Cyanobacteria_Prochlorales_Prochlorotrichaceae_Prochlorothrix_hollandica_PCC_9006:0.15436814278820243):0.06476950385183056[98],('Bacteria_Cyanobacteria_Oscillatoriales_Leptolyngbya_sp._PCC_7104':0.15690175009087737,'Bacteria_Cyanobacteria_Oscillatoriales_Oscillatoriales_sp._JSC_1':0.11886173233808695):0.029339084033747742[49]):0.01804408265446078[22],'Bacteria_Cyanobacteria_Oscillatoriales_Geitlerinema_sp._PCC_7407':0.11846227414109256):0.020120304182344118[46]):0.029487680978827147[46],(('Bacteria_Cyanobacteria_Chroococcales_Cyanothece_sp._PCC_7425':0.14104265792052306,Bacteria_Cyanobacteria_Chroococcales_Thermosynechococcus_elongatus_BP_1:0.15963078381954876):0.038564740142279064[93],Bacteria_Cyanobacteria_Chroococcales_Acaryochloris_marina_MBIC11017:0.16992053169835053):0.03939498948252407[66]):0.05284897542687794[44],'Bacteria_Cyanobacteria_Oscillatoriales_Pseudanabaena_sp._PCC_7367':0.26258924295291175):0.0999724305871208[100],Bacteria_Cyanobacteria_Gloeobacteria_Gloeobacterales_Gloeobacter_violaceus_PCC_7421:0.2990756935968726):0.1855943573175729[100],Bacteria_CP_novel_BJP_IG2069_Novel_57_5:0.5482545742151035):0.05466056432536037[58],((((((((((Bacteria_Melainabacteria_Gastranaerophilaceae_MH_37:0.000854,Bacteria_Melainabacteria_MEL_C1:0.000001):0.007362564120524606[100],Bacteria_Melainabacteria_Gastranaerophilales_YS2_Zagget_bin_1:0.0061305482345548334):0.020541481418880903[98],Bacteria_Melainabacteria_MEL_A1:0.045703347038280384):0.04791504734914698[100],Bacteria_Melainabacteria_Gastranaerophilaceae_Zag_111:0.08841908445674518):0.015923647132050966[51],Bacteria_Melainabacteria_Gastranaerophilales_YS2_Zagget_bin_221:0.09098235271975108):0.028873450254280897[54],Bacteria_Melainabacteria_MEL_B1:0.12014870672001399):0.03604162165750857[99],Ga0172381_10002205:0.10356205054589385):0.07255121736128523[100],Bacteria_Melainabacteria_MEL_B2:0.14266065396235872):0.11795978045628974[100],(((Bacteria_Melainabacteria_GWF2_Melainabacteria_32_7:0.000942,Bacteria_Melainabacteria_RIFOXYA2_FULL_Melainabacteria_32_9:0.000001):0.05300186936539708[100],Bacteria_Melainabacteria_ACD20:0.053627005685547235):0.1274231759849105[100],(Bacteria_Melainabacteria_GWF2_Melainabacteria_37_15:0.1664379182383393,Bacteria_Melainabacteria_GWA2_Melainabacteria_34_9:0.1411982756877732):0.07663478675368429[100]):0.05848604427410509[99]):0.22459162465607152[100],((((Ga0172379_10021151:0.0011640086659832072,Ga0172380_10000269:0.000619):0.007031365615775442[94],Ga0172379_10009165:0.0031498007607679845):0.11190071641151578[100],Bacteria_Melainabacteria_Obscuribacteriales_Obscuribacter_phosphatis_Mle1_12:0.08561893335121518):0.3126561388405591[100],(Bacteria_Melainabacteria_RIFCSPLOWO2_12_FULL_Melainabacteria_35_11:0.17808239681320748,Bacteria_Melainabacteria_RIFCSPHIGHO2_02_FULL_Melainabacteria_34_12:0.1267825103064606):0.40772974379052407[100]):0.05997138951775005[69]):0.08100430458559371[97]):0.08522494029959793[100],(((((((Bacteria_WOR_1_RIFOXYA2_FULL_WOR_1_51_19:0.0761390759464664,Ga0172379_10002152:0.08648144546800962):0.19529281780294694[100],Ga0172379_10004630:0.3206418119223131):0.04739591090606998[72],(Bacteria_WOR_1_RIFCSPHIGHO2_01_FULL_WOR_1_53_15:0.18849025311900114,Ga0172379_10013867:0.2176044876496701):0.07844511560090917[100]):0.02060588570069033[33],Bacteria_WOR_1_RIFOXYB2_FULL_WOR_1_48_7:0.27987396226781946):0.11600238500247473[100],(((Ga0172379_10024418:0.2642762338299547,Ga0172379_10011153:0.2400229294640832):0.04569608328719266[61],(Bacteria_WOR_1_RIFCSPLOWO2_02_FULL_WOR_1_46_20:0.22786226888858652,Bacteria_WOR_1_uncultured_DG_54_3:0.1873272949593514):0.033022007915649354[62]):0.047293734768128814[79],Bacteria_WOR_1_RIFOXYC2_FULL_WOR_1_41_25:0.31710449036054067):0.09258453901607444[100]):0.10403349920424265[100],(((Bacteria_WOR_1_RIFOXYA2_FULL_WOR_1_36_21:0.18969268925003835,Bacteria_WOR_1_RIFOXYB2_FULL_WOR_1_37_13:0.15657748656806048):0.2791846023277209[100],Bacteria_WOR_1_RIFOXYA12_FULL_WOR_1_43_27:0.47601339393419106):0.05598570486140675[69],Ga0172379_10023595:0.4078272232735798):0.0664273342124635[100]):0.17387915722560088[100],(Bacteria_RBX1_GWF2_RBX1_35_9:0.624776477166129,Bacteria_RBX1_GWF2_RBX1_38_17:0.4428653524257471):0.15751811278551653[100]):0.09048848470634407[100]):0.06307385028223589[81]):0.022654729644922877[1]):0.04006246001903424[30],((((((((((Bacteria_Aquificae_Aquificae_Aquificales_Aquificaceae_Hydrogenobacter_thermophilus_TK_6:0.14014477532274094,Bacteria_Aquificae_Aquificae_Aquificales_Aquificaceae_Thermocrinis_albus_DSM_14484:0.10846985533383124):0.04728731186133439[99],'Bacteria_Aquificae_Aquificae_Aquificales_Aquificaceae_Hydrogenobaculum_sp._HO_Finished_QAed':0.3443261211563593):0.05313050395041685[99],Bacteria_Aquificae_Aquificae_Aquificales_Aquificaceae_Aquifex_aeolicus_VF5:0.20953259589270523):0.1777712286694655[100],(Bacteria_Aquificae_Aquificae_Aquificales_Hydrogenothermaceae_Sulfurihydrogenibium_azorense_Az_Fu1:0.1531432687146883,Bacteria_Aquificae_Aquificae_Aquificales_Hydrogenothermaceae_Persephonella_marina_EX_H1:0.12011197620570835):0.1627768722353169[100]):0.14205590213168007[100],(Bacteria_Aquificae_Aquificae_Aquificales_Desulfurobacteriaceae_Desulfurobacterium_thermolithotrophum_DSM_11699:0.0574045013834521,Bacteria_Aquificae_Aquificae_Aquificales_Desulfurobacteriaceae_Thermovibrio_ammonificans_HB_1:0.04946874262617085):0.2601016842054267[100]):0.0703347403172403[94],(((Bacteria_CP_EM_19_candidate_division_EM_19_bacterium_JGI_0000106_J16_GBS_C_001_289:0.00362501788070535,Bacteria_CP_EM_19_candidate_division_EM_19_bacterium_JGI_0000106_G12_Combined_Assembly_EM19_1__EM19:0.000001):0.0013591852288565853[88],Bacteria_CP_EM_19_candidate_division_EM_19_bacterium_JGI_0000106_N7_GBS_C_001_292:0.000001):0.007191978977042268[99],Bacteria_CP_EM_19_candidate_division_EM_19_bacterium_SCGC_AAA471_M6_GBS_N_001_25:0.000173):0.7916701551147787[100]):0.06815179929910498[88],((((Ga0172382_10041883:0.029414088171059394,Ga0172382_10041895:0.013150594850998232):0.1505498995882384[100],Bacteria_Deinococcus_Thermus_Deinococci_Deinococcales_Trueperaceae_Truepera_radiovictrix_DSM_17093:0.13268188162009942):0.20982024403318178[100],Bacteria_Deinococcus_Thermus_Deinococci_Deinococcales_Deinococcaceae_Deinococcus_geothermalis_DSM_11300:0.24760471183973554):0.09719362757274386[100],((Bacteria_Deinococcus_Thermus_Deinococci_Thermales_Thermaceae_Oceanithermus_profundus_DSM_14977:0.15383704507898033,Bacteria_Deinococcus_Thermus_Deinococci_Thermales_Thermaceae_Marinithermus_hydrothermalis_T1_DSM_14884:0.08550234664921907):0.05364868214531482[98],(Bacteria_Deinococcus_Thermus_Deinococci_Thermales_Thermaceae_Meiothermus_ruber_DSM_1279:0.21950862336544974,Bacteria_Deinococcus_Thermus_Deinococci_Thermales_Thermaceae_Thermus_oshimai_JL_2:0.1250298175290938):0.02992867202984817[60]):0.1280771599823498[100]):0.3166876512833512[100]):0.04865911200552597[44],(((((((((Ga0172377_10010471:0.000001,Ga0172382_10003098:0.000001):0.000389[63],Ga0172378_10001014:0.000460):0.11829839410821741[100],Bacteria_Thermotogae_Thermotogae_Thermotogales_Thermotogaceae_Petrotoga_mobilis_SJ95:0.12231725903230917):0.19478505103229748[100],Bacteria_Thermotogae_Thermotogae_Thermotogales_Thermotogaceae_Marinitoga_piezophila_KA3:0.14960833516529393):0.10332615634264553[100],(((Ga0172377_10000457:0.000001,Ga0172382_10010317:0.000438):0.027950092624914546[100],'Bacteria_Thermotogae_Thermotogae_Thermotogales_Thermotogales_sp._mesG1.Ag.4.2':0.015924947609104123):0.20276557665179418[100],'Bacteria_Thermotogae_Thermotogae_Thermotogales_Thermotogaceae_Kosmotoga_olearia_TBF_19.5.1':0.08062664418112231):0.12901280178666719[100]):0.03755649720692844[94],((Bacteria_Thermotogae_Thermotogae_Thermotogales_Thermotogaceae_Thermosipho_africanus_TCF52B:0.13700511191992248,Bacteria_Thermotogae_Thermotogae_Thermotogales_Thermotogaceae_Fervidobacterium_nodosum_Rt17_B1:0.17547841777277595):0.08095233648721045[100],(Bacteria_Thermotogae_Thermotogae_Thermotogales_Thermotogaceae_Thermotoga_thermarum_LA3_DSM_5069:0.2013589724277809,Bacteria_Thermotogae_Thermotogae_Thermotogales_Thermotogaceae_Thermotoga_maritima_MSB8:0.13513710116567346):0.03860973754057939[87]):0.0591505880116201[100]):0.0720404497760967[99],Bacteria_Thermotogae_Thermotogae_Thermotogales_unclassified_Thermotogales_Laodecianella_thermoacidophila_DSM_25116:0.4080765454930946):0.13363142547290696[100],Bacteria_KB1_RBG_16_KB1_OP1_55_9:0.7164874056793891):0.05776688856382428[79],((Bacteria_Caldiserica_Caldisericia_Caldisericales_Caldisericaceae_Caldisericum_exile_AZM16c01_NBRC_104410:0.15300196349409978,Bacteria_Caldiserica_Caldisericia_Caldisericales_Caldisericaceae_Caldisericum_CG_Caldi_01:0.2441440607433587):0.47206641258432036[100],(Bacteria_Dictyoglomi_Dictyoglomia_Dictyoglomales_Dictyoglomaceae_Dictyoglomus_thermophilum_H_6_12:0.01735869305450599,Bacteria_Dictyoglomi_Dictyoglomia_Dictyoglomales_Dictyoglomaceae_Dictyoglomus_turgidum_DSM_6724:0.02086889454446661):0.3589535403320321[100]):0.10681606381046516[99]):0.04312160905739093[49]):0.04996466196313332[46],((((((((Ga0172378_10039572:0.000001,Ga0172380_10015276:0.000001):0.010188125091484146[100],Ga0172382_10006975:0.009370197746661368):0.0668823404039518[100],Bacteria_Synergistetes_Synergistia_Synergistales_Synergistaceae_Cloacibacillus_evryensis_158_DSM_19522:0.08119044295127065):0.11688720435767586[100],(Bacteria_Synergistetes_Synergistia_Synergistales_Synergistaceae_Thermanaerovibrio_acidaminovorans_DSM_6589:0.09482562459059451,Bacteria_Synergistetes_Synergistia_Synergistales_Synergistaceae_Aminomonas_paucivorans_GLU_3_DSM_12260:0.09370320264877874):0.05379107983573972[100]):0.08835902520893812[100],Bacteria_Synergistetes_Synergistia_Synergistales_Synergistaceae_Aminiphilus_circumscriptus_DSM_16581:0.2234605968232639):0.049485908904344456[100],((Ga0172382_10026496:0.000001,Ga0172377_10020474:0.000001):0.1616801499784657[100],Bacteria_Synergistetes_Synergistia_Synergistales_Synergistaceae_Thermovirga_lienii_Cas60314_DSM_17291:0.11629329618718165):0.06420486795564306[100]):0.028237464113336053[42],(((((Ga0172377_10000648:0.000001,Ga0172378_10001645:0.000001):0.04662622860034782[100],Bacteria_Synergistetes_Synergistia_Synergistales_Synergistaceae_Aminobacterium_colombiense_DSM_12261:0.036240242000241896):0.11404327926513069[100],Ga0172382_10005875:0.17555995338483177):0.046907741886932364[68],'Bacteria_Synergistetes_Synergistetes_sp._SGP1_draft_genome.':0.24378653848265897):0.05956659655344998[100],((Bacteria_Synergistetes_Synergistia_Synergistales_Synergistaceae_Pyramidobacter_piscolens_W5455_contig00040:0.14188410462572776,Bacteria_Synergistetes_Synergistia_Synergistales_Synergistaceae_Jonquetella_anthropi_E3_33_E1:0.2061038648836906):0.124947687019211[100],Bacteria_Synergistetes_Synergistia_Synergistales_Synergistaceae_Dethiosulfovibrio_peptidovorans_SEBR_4207_DSM_11002:0.18926607163635278):0.08849095995207756[100]):0.052426058901561934[99]):0.04727472147959011[89],Bacteria_Synergistetes_Synergistia_Synergistales_Synergistaceae_Anaerobaculum_mobile_NGA_DSM_13181:0.2580563976752783):0.25466014133058756[100]):0.026464241450498527[6],(((((((Ga0172377_10008156:0.003420389472120533,Ga0172378_10007743:0.005124432813139457):0.16610961879623654[100],Bacteria_Fusobacteria_Fusobacteriia_Fusobacteriales_Fusobacteriaceae_Fusobacterium_nucleatum_nucleatum_ATCC_25586:0.13004162917329598):0.030078182554941435[47],(Bacteria_Fusobacteria_Fusobacteriia_Fusobacteriales_Fusobacteriaceae_Cetobacterium_somerae_ATCC_BAA_474:0.008773838808738166,'Bacteria_Fusobacteria_Fusobacteriia_Fusobacteriales_Fusobacteriaceae_Cetobacterium_sp._ZWU0022':0.0073950705812069195):0.0683321108609487[100]):0.031294115181816906[96],Bacteria_Fusobacteria_Fusobacteriia_Fusobacteriales_Fusobacteriaceae_Ilyobacter_polytropus_DSM_2926:0.08707163640200077):0.03035781428512907[74],Bacteria_Fusobacteria_Fusobacteriia_Fusobacteriales_Fusobacteriaceae_Psychrilyobacter_atlanticus_DSM_19335:0.15365460228935524):0.1305228999247494[100],((Bacteria_Fusobacteria_Fusobacteriia_Fusobacteriales_Leptotrichiaceae_Leptotrichia_goodfellowii_F0264_contig00021:0.09256391896846639,Bacteria_Fusobacteria_Fusobacteriia_Fusobacteriales_Leptotrichiaceae_Sebaldella_termitidis_ATCC_33386:0.1513798657718355):0.04682289501542103[57],Bacteria_Fusobacteria_Fusobacteriia_Fusobacteriales_Leptotrichiaceae_Streptobacillus_moniliformis_DSM_12112:0.1650770129096082):0.1450665405881999[100]):0.3392831725687917[100],((((Bacteria_CP_ACD39_BJP_IG2103_ACD39_50_23:0.01734297397416462,Bacteria_ACD39_GWC2_ACD39_50_8:0.006370621579765867):0.028638149157897086[100],Bacteria_CP_ACD39_BJP_IG2102_CP_ACD39_51_10:0.026065151957993304):0.1815449158779474[100],Bacteria_Bayareabacteria_CG_ACD39_01:0.2208720184260562):0.45348717233095526[100],(((Ga0172382_10019232:0.10930762435626873,Ga0172382_10019060:0.20994539825053504):0.0030527470510763877[51],Ga0172382_10055435:0.14557820825110568):0.38013833977208566[100],Bacteria_OP9X_GWC2_OP9X_49_35:0.49979003037332115):0.0625827968132695[81]):0.07393348400373956[90]):0.0432266229056153[16]):0.024274796985496394[10]):0.019956610356889115[15],((((((Bacteria_CP_OP9_candidate_division_OP9_bacterium_SCGC_AAA255_G05_SAK_001_130:0.004120900240581982,Bacteria_CP_OP9_candidate_division_OP9_bacterium_SCGC_AAA255_N14_SAK_001_136:0.010466375714854426):0.03687525425173943[100],Bacteria_Atribacteria_RBG_19FT_COMBO_JS1_35_14:0.039664897656157994):0.06255271309451649[100],Bacteria_OP9_CG_OP9_01:0.09710767050541058):0.036270035746297946[87],Bacteria_CP_JS1_candidate_division_JS1_bacterium_SCGC_5329:0.14580430887405793):0.14297903338260598[100],((Ga0172377_10003362:0.11117778228276132,Bacteria_CP_OP9_candidate_division_OP9_bacterium_JGI_0000059_I14_TAbiofilm_001_167:0.08760858059830978):0.0631537005873084[100],(Ga0172377_10000949:0.000979,Ga0172382_10002783:0.000704):0.13098785738788132[100]):0.18653576886218737[100]):0.24182844896974576[100],(Bacteria_Atribacteria_OP9_Candidatus_Caldatribacterium_saccharofermentans_OP9_77CS:0.006741768964347639,Bacteria_unclassified_Bacteria_Atribacteria_Candidatus_Caldatribacterium_californiense_OP9_cSCG:0.015325933755391485):0.4699686496748985[100]):0.07976491072910896[75]):0.021895654687285493[12],(((Bacteria_unclassified_Bacteria_Poribacteria_Candidatus_Poribacteria_WGA_3G_final_clean_version:0.14292742675928372,'Bacteria_unclassified_Bacteria_Poribacteria_Candidatus_Poribacteria_sp._WGA_4C_final_clean_version':0.13903151138228242):0.39626747498947257[100],Bacteria_RIF8_RBG_13_RIF08_66_14:0.6703622264001527):0.07105388018414738[64],(Bacteria_CP_BRC1_BJP_IG2102_BRC1_58_13:0.41486153437747353,Bacteria_BRC1_uncultured_SMTZ_51:0.4093675474157963):0.21855665034754335[100]):0.06262283096699983[40]):0.023681376022657297[3],(((((((((((((((((((((((((((((Ga0172378_10000601:0.0012451375545401433,Ga0172377_10001167:0.000401):0.03124389340931666[100],(Ga0172377_10004407:0.000001,Ga0172378_10000045:0.000001):0.02092852231855824[100]):0.06421345452988847[100],(Ga0172382_10008394:0.026097170883214282,Bacteria_BacteroidetesChlorobi_group_Bacteroidetes_Bacteroidia_Bacteroidales_Porphyromonadaceae_Proteiniphilum_acetatigenes_DSM_18083:0.007717517258881923):0.024111691671722557[99]):0.011890437400364107[68],((((Ga0172378_10035098:0.000001,Ga0172380_10009144:0.011817168553153845):0.000001[88],Ga0172381_10029999:0.000001):0.004657528120009413[100],Ga0172380_10017507:0.0022915008235919743):0.04213884408162727[100],Ga0172382_10008573:0.04346117195283261):0.017001432172870867[97]):0.029986931333279898[100],(Ga0172378_10036712:0.07908316736383636,Ga0172378_10011947:0.12897436084938363):0.0173943773121481[67]):0.025089439551463588[90],(((Ga0172377_10007439:0.000001,Ga0172378_10011165:0.000430):0.000846[87],Ga0172382_10005656:0.000001):0.000454[58],Ga0172381_10002328:0.000367):0.12714319112161432[100]):0.05628153278753967[100],'Bacteria_BacteroidetesChlorobi_group_Bacteroidetes_Bacteroidia_Bacteroidales_Porphyromonadaceae_Dysgonomonas_gadei_ATCC_BAA_286_cont1.32':0.0919051693735713):0.0349828947105526[99],(((((Ga0172377_10008178:0.000001,Ga0172378_10013700:0.000001):0.0714278153552006[100],Bacteria_BacteroidetesChlorobi_group_Bacteroidetes_Bacteroidia_Bacteroidales_Porphyromonadaceae_Parabacteroides_distasonis_ATCC_8503:0.02362006937690797):0.01680668120665363[82],(Ga0172378_10002555:0.000001,Ga0172377_10000471:0.0015458018357819014):0.07059616478348874[100]):0.021984119780336098[96],Bacteria_BacteroidetesChlorobi_group_Bacteroidetes_Bacteroidia_Bacteroidales_Porphyromonadaceae_Tannerella_forsythensis_ATCC_43037:0.1332332906220608):0.026938354326272673[96],Bacteria_BacteroidetesChlorobi_group_Bacteroidetes_Bacteroidia_Bacteroidales_Porphyromonadaceae_Porphyromonas_gingivalis_W83:0.1544163007649817):0.024483998705266963[86]):0.015646147688515466[54],(Ga0172377_10025497:0.16312367628655888,'Bacteria_BacteroidetesChlorobi_group_Bacteroidetes_Bacteroidia_Bacteroidales_Candidatus_Azobacteroides_pseudotrichonymphae_genomovar._CFP2':0.2813985326519588):0.03277955509939279[49]):0.018786073831090988[55],(Bacteria_BacteroidetesChlorobi_group_Bacteroidetes_Bacteroidia_Bacteroidales_Porphyromonadaceae_Barnesiella_viscericola_C46_DSM_18177:0.07767771608076401,Bacteria_BacteroidetesChlorobi_group_Bacteroidetes_Bacteroidia_Bacteroidales_Porphyromonadaceae_Coprobacter_fastidiosus_NSB1:0.06299362846497569):0.045068438856552095[100]):0.011150784495423505[44],(((((((((Ga0172378_10011255:0.000001,Ga0172377_10029186:0.000001):0.002544132727478665[96],Ga0172377_10036581:0.000001):0.05801737069230395[100],(Bacteria_BacteroidetesChlorobi_group_Bacteroidetes_Bacteroidia_Bacteroidales_Prevotellaceae_Hallella_seregens_ATCC_51272:0.000001,Bacteria_BacteroidetesChlorobi_group_Bacteroidetes_Bacteroidia_Bacteroidales_Prevotellaceae_Prevotella_dentalis_ES_2772_DSM_3688:0.000001):0.04000245956713222[100]):0.03402886637233893[100],((Ga0172377_10007903:0.041476919020495995,Ga0172377_10033333:0.12454526469084204):0.019295717321468064[85],Ga0172378_10002015:0.05635013692099822):0.012096162015942102[57]):0.013488221435124181[85],Ga0172378_10006159:0.07270749329611848):0.031142862451005726[100],(Ga0172377_10001111:0.000696,Ga0172378_10014365:0.000001):0.05979200473064372[100]):0.07965848375281759[100],((Ga0172378_10019343:0.000662,Ga0172377_10003434:0.000001):0.11641423951364605[100],Bacteria_BacteroidetesChlorobi_group_Bacteroidetes_Bacteroidia_Bacteroidales_Prevotellaceae_Prevotella_tannerae_ATCC_51259:0.15741431541355988):0.03919992856429122[99]):0.022170658563350987[95],'Bacteria_BacteroidetesChlorobi_group_Bacteroidetes_Bacteroidia_Bacteroidales_Prevotellaceae_Paraprevotella_xylaniphila_YIT_11841_P_xylaniphilaYIT11841_1.0_Cont168.1':0.07132089415717457):0.05443065553157034[100],((((((Ga0172378_10001526:0.002034321133920436,Ga0172377_10017067:0.000243):0.1291690141299795[100],(Ga0172378_10002171:0.000001,Ga0172377_10000558:0.000831):0.16497080467071257[100]):0.02788156567902922[72],((Ga0172378_10004221:0.000001,Ga0172377_10003982:0.000001):0.09288633711684957[100],Bacteria_BacteroidetesChlorobi_group_Bacteroidetes_Bacteroidia_Bacteroidales_Bacteroidaceae_Bacteroides_fragilis_NCTC_9343:0.030288354136891904):0.008960797908762252[50]):0.01846624663794838[82],Ga0172378_10010589:0.0534694305253014):0.013676257025578398[63],Ga0172378_10004372:0.06386609040619096):0.011680176764107042[47],((Ga0172378_10000263:0.000001,Ga0172377_10035667:0.000001):0.1003401923224625[100],(Ga0172377_10030263:0.005569297318010591,Ga0172378_10019218:0.01577085203943218):0.12623078798139575[100]):0.021337957131115637[95]):0.01386844068507509[53]):0.06362940410352103[100]):0.025382758769194602[85],(((((Ga0172377_10005006:0.000001,Ga0172382_10040507:0.004498789393623692):0.09788583309473431[100],Ga0172379_10030958:0.032220028556253055):0.036052410053155626[99],((Bacteria_BacteroidetesChlorobi_group_Bacteroidetes_Bacteroidia_Bacteroidales_Porphyromonadaceae_Paludibacter_propionicigenes_WB4:0.030933126840790415,Ga0172378_10010101:0.04352713721752943):0.008370337638177627[51],Ga0172379_10004727:0.061507347864528494):0.04350233445242013[100]):0.07334527752595621[100],(Ga0172377_10028444:0.09630129911616336,Ga0172381_10011348:0.11338842660761861):0.01994947267277203[49]):0.020372991244183236[53],(Ga0172378_10014305:0.028930613597179633,Ga0172378_10005815:0.017974522297544482):0.11519790263936969[100]):0.014336001444206214[29]):0.10520661692015265[100],((((Bacteria_BacteroidetesChlorobi_group_Bacteroidetes_Bacteroidia_Bacteroidales_Marinilabiaceae_Anaerophaga_thermohalophila_Fru22_DSM_12881:0.03220384706138324,Bacteria_BacteroidetesChlorobi_group_Bacteroidetes_Bacteroidia_Bacteroidales_Marinilabiaceae_Marinilabilia_salmonicolor_JCM_21150:0.058927431560267696):0.032254889334507375[90],'Bacteria_BacteroidetesChlorobi_group_Bacteroidetes_Bacteroidia_Bacteroidales_Marinilabiaceae_Anaerophaga_sp._HS1':0.05823459855175983):0.06820344657103261[100],Bacteria_BacteroidetesChlorobi_group_Bacteroidetes_Bacteroidia_Bacteroidales_Marinilabiaceae_Alkaliflexus_imshenetskii_Z_7010_DSM_15055:0.09527617324068682):0.03735107449264019[97],Bacteria_BacteroidetesChlorobi_group_Bacteroidetes_Cytophagia_Cytophagales_Cytophagaceae_Cytophaga_fermentans_IAM_14302_DSM_9555:0.12362890791349557):0.05595688470267568[100]):0.038874083917215696[100],(Bacteria_BacteroidetesChlorobi_group_Bacteroidetes_Bacteroidia_Bacteroidales_Porphyromonadaceae_Butyricimonas_virosa_DSM_23226:0.06777792156953044,Bacteria_BacteroidetesChlorobi_group_Bacteroidetes_Bacteroidia_Bacteroidales_Porphyromonadaceae_Odoribacter_splanchnicus_DSM_20712:0.085359589744471):0.161556538951666[100]):0.02160444226002145[38],((((((((Ga0172378_10017245:0.000001,Ga0172381_10019755:0.000599):0.000915[50],Ga0172381_10004298:0.000001):0.000001[22],Ga0172382_10039329:0.000001):0.000001[41],Ga0172377_10007667:0.000001):0.000001[65],Ga0172378_10025224:0.000001):0.07884129581734145[100],Ga0172379_10001333:0.1006613821600193):0.14664634163911927[100],((Ga0172379_10000822:0.06268749208107494,Ga0172379_10025205:0.04297091986259405):0.13756435859133154[100],Ga0172382_10015316:0.2583543838328217):0.034105525687071925[65]):0.02660470900888745[46],((((Ga0172382_10000656:0.07677190250541877,Ga0172377_10039841:0.09258338803519939):0.037471679521097645[100],Bacteria_BacteroidetesChlorobi_group_Bacteroidetes_Bacteroidia_Bacteroidales_Prolixibacteraceae_Draconibacterium_orientale_FH5:0.07923118229977621):0.05702698935767536[100],(Ga0172382_10013206:0.10671206407995237,Ga0172379_10045267:0.12651480661862857):0.025751104118752366[95]):0.04147524666661262[99],Bacteria_BacteroidetesChlorobi_group_Bacteroidetes_unclassified_Bacteroidetes_Prolixibacter_bellariivorans_ATCC_BAA_1284:0.11664365180308289):0.0676105666532485[100]):0.02887536953860037[48]):0.0341909234668023[60],(((((((((Ga0172377_10067332:0.000001,Ga0172378_10041433:0.000001):0.001655674153976161[99],Ga0172380_10010289:0.001646913459760757):0.0016473045691656196[87],Ga0172381_10021210:0.000001):0.0569920651411806[100],(((Ga0172382_10000234:0.000001,Ga0172377_10000943:0.000001):0.000001[100],Ga0172378_10001640:0.0018130113345353749):0.026952503059280986[100],Ga0172380_10002704:0.0334526892227327):0.01620531926473534[62]):0.01961674632854793[82],(Ga0172382_10014012:0.000001,Ga0172377_10000964:0.000001):0.06080679182556592[100]):0.1014828789932598[99],(((Ga0172377_10034333:0.000742,Ga0172378_10030639:0.006836943056821365):0.0833889433874937[100],(Ga0172377_10003239:0.21348917654659405,Ga0172377_10023845:0.10878212947350674):0.026010348847710585[62]):0.03734658160816684[89],(Ga0172381_10001258:0.07518025725642596,Ga0172377_10014929:0.12609805312350408):0.03821840998250847[91]):0.05294210149126233[96]):0.15519013547200888[91],(Bacteria_BacteroidetesChlorobi_group_Bacteroidetes_Bacteroidia_Bacteroidales_Rikenellaceae_Rikenella_microfusus_Q_1_DSM_15922:0.12915630458634464,Bacteria_BacteroidetesChlorobi_group_Bacteroidetes_Bacteroidia_Bacteroidales_Rikenellaceae_Alistipes_finegoldii_AHN_2437_DSM_17242:0.15263532974989946):0.07005577785081796[100]):0.000001[37],(((((Ga0172380_10000264:0.000001,Ga0172381_10000169:0.000847):0.031069141112621157[100],Ga0172378_10011434:0.031563964439969894):0.014580088523739931[82],((Ga0172382_10010321:0.000001,Ga0172377_10003953:0.000001):0.02624590789449277[100],Ga0172377_10004887:0.07578683764184824):0.06771770116860365[100]):0.03122266238133209[86],(Ga0172380_10003768:0.000001,Ga0172378_10002710:0.000816):0.05181448172364833[100]):0.08741707731971804[99],(Ga0172378_10000808:0.20078290543866073,Ga0172377_10000464:0.13232154269682272):0.03418845607133969[58]):0.07622176129868752[88]):0.04770671914597502[96],Ga0172381_10018247:0.21077008406948083):0.039335456239699695[93]):0.057969056560761345[100],((((((((Ga0172381_10010470:0.000754,Ga0172377_10015759:0.000001):0.0012783766834982835[100],Ga0172378_10014114:0.000001):0.014493053732406391[99],Ga0172378_10017781:0.05341598793295965):0.018731900986567407[67],Ga0172377_10036529:0.04814562532774813):0.022247613235629515[87],(Ga0172378_10039416:0.000001,Ga0172377_10003634:0.0013534502843475238):0.04361406699112891[100]):0.17730098248641113[100],(((Ga0172377_10022076:0.000001,Ga0172382_10014987:0.004686394994294574):0.002622140530591288[88],Ga0172382_10008148:0.000705):0.04165781846738437[100],((Ga0172378_10005666:0.000001,Ga0172382_10011342:0.000001):0.000001[46],Ga0172377_10000416:0.000001):0.045616571174369014[100]):0.19568499787271998[100]):0.048164576605431275[97],((((Ga0172381_10000160:0.061964313175972396,Ga0172381_10000051:0.06548964276416447):0.1601229004496676[100],Ga0172381_10002610:0.16419996243621426):0.022828686425440115[40],(Ga0172377_10053847:0.000574,Ga0172378_10001162:0.005761914542995683):0.18040147990645128[100]):0.03571669305371161[92],(Ga0172377_10021385:0.17283215016533227,Ga0172382_10001343:0.20618307040573258):0.04413468797412001[56]):0.022384930386083912[28]):0.03416234421427111[48],((Ga0172382_10003961:0.000001,Ga0172377_10001467:0.000410):0.4278166349045027[100],Ga0172377_10000306:0.20303376830121067):0.0489877093927209[37]):0.05703384863504457[97]):0.03171298894169494[94],((((((((((((('Bacteria_BacteroidetesChlorobi_group_Bacteroidetes_Flavobacteriia_Flavobacteriales_Flavobacteriaceae_Salegentibacter_sp._Hel_I_6':0.051389803211697505,Bacteria_BacteroidetesChlorobi_group_Bacteroidetes_Flavobacteriia_Flavobacteriales_Flavobacteriaceae_Zunongwangia_profunda_SM_A87:0.06318908124959188):0.01581779373786185[54],Bacteria_BacteroidetesChlorobi_group_Bacteroidetes_Flavobacteriia_Flavobacteriales_Flavobacteriaceae_Gramella_forsetii_KT0803:0.04837754031537056):0.025838519056108566[98],(Bacteria_BacteroidetesChlorobi_group_Bacteroidetes_Flavobacteriia_Flavobacteriales_Flavobacteriaceae_Salinimicrobium_xinjiangense_DSM_19287:0.05008057111160058,'Bacteria_BacteroidetesChlorobi_group_Bacteroidetes_Flavobacteriia_Flavobacteriales_Flavobacteriaceae_Gillisia_sp._CAL575':0.06694048714378331):0.012004923053450689[42]):0.019145112010729992[92],((Bacteria_BacteroidetesChlorobi_group_Bacteroidetes_Flavobacteriia_Flavobacteriales_Flavobacteriaceae_Psychroflexus_gondwanensis_ACAM_44:0.20491557824181816,Bacteria_BacteroidetesChlorobi_group_Bacteroidetes_Flavobacteriia_Flavobacteriales_Flavobacteriaceae_Croceibacter_atlanticus_HTCC2559_Re_annotation_of_existing_genome_that_was_not_originally_submitted_by_me:0.09988999013975874):0.031766092509402544[54],Bacteria_BacteroidetesChlorobi_group_Bacteroidetes_Flavobacteriia_Flavobacteriales_Flavobacteriaceae_Mesonia_mobilis_DSM_19841:0.07895621927937313):0.026319111961389474[56]):0.024740735579541706[56],((Bacteria_BacteroidetesChlorobi_group_Bacteroidetes_Flavobacteriia_Flavobacteriales_Flavobacteriaceae_Leeuwenhoekiella_blandensis_MED217:0.07368039037425467,'Bacteria_BacteroidetesChlorobi_group_Bacteroidetes_Flavobacteriia_Flavobacteriales_Flavobacteriaceae_Krokinobacter_sp._4H_3_7_5':0.10793092534881144):0.04102723134151587[100],Bacteria_BacteroidetesChlorobi_group_Bacteroidetes_Flavobacteriia_Flavobacteriales_Flavobacteriaceae_Donghaeana_dokdonensis_DSW_6:0.1856837620687437):0.016417568963242424[42]):0.012149381988129981[35],Bacteria_BacteroidetesChlorobi_group_Bacteroidetes_Flavobacteriia_Flavobacteriales_Flavobacteriaceae_Aquimarina_megaterium_XH134:0.07271651237285015):0.01762691395809668[40],((((((Bacteria_BacteroidetesChlorobi_group_Bacteroidetes_Flavobacteriia_Flavobacteriales_Flavobacteriaceae_Mesoflavibacter_zeaxanthinifaciens_DSM_18436:0.028889392641759493,'Bacteria_BacteroidetesChlorobi_group_Bacteroidetes_Flavobacteriia_Flavobacteriales_Flavobacteriaceae_Olleya_sp._Hel_I_94':0.030720898241821892):0.015330866211054506[100],'Bacteria_BacteroidetesChlorobi_group_Bacteroidetes_Flavobacteriia_Flavobacteriales_Flavobacteriaceae_Lacinutrix_sp._5H_3_7_4':0.03390734895268244):0.01973851626949319[99],(Bacteria_BacteroidetesChlorobi_group_Bacteroidetes_Flavobacteriia_Flavobacteriales_Flavobacteriaceae_Flavobacteriaceae_bacterium_P7_3_5:0.037444008590995725,Bacteria_BacteroidetesChlorobi_group_Bacteroidetes_Flavobacteriia_Flavobacteriales_Flavobacteriaceae_Gaetbulibacter_saemankumensis_DSM_17032:0.043951272853410384):0.016719796568875456[100]):0.005705211981103631[24],(Bacteria_BacteroidetesChlorobi_group_Bacteroidetes_Flavobacteriia_Flavobacteriales_Flavobacteriaceae_Bizionia_argentinensis_JUB59_DSM_19628:0.06938797427402976,Bacteria_BacteroidetesChlorobi_group_Bacteroidetes_Flavobacteriia_Flavobacteriales_Flavobacteriaceae_Formosa_agariphila_KMM_3901:0.04404255705198379):0.01105319015134798[44]):0.010054756932739384[29],((Bacteria_BacteroidetesChlorobi_group_Bacteroidetes_Flavobacteriia_Flavobacteriales_Flavobacteriaceae_Gelidibacter_mesophilus_DSM_14095:0.0722464261962088,'Bacteria_BacteroidetesChlorobi_group_Bacteroidetes_Flavobacteriia_Flavobacteriales_Flavobacteriaceae_Sediminibacter_sp._Hel_I_10':0.05887360415964915):0.019111377329141366[76],Bacteria_BacteroidetesChlorobi_group_Bacteroidetes_Flavobacteriia_Flavobacteriales_Flavobacteriaceae_Winogradskyella_psychrotolerans_RS_3:0.08202060116970333):0.01780516240387442[85]):0.03057139265509523[100],Bacteria_BacteroidetesChlorobi_group_Bacteroidetes_Flavobacteriia_Flavobacteriales_Flavobacteriaceae_Aequorivita_sublithincola_QSSC9_3_DSM_14238:0.10412886489260309):0.022468468755555193[93]):0.020165191448809683[97],((((((Bacteria_BacteroidetesChlorobi_group_Bacteroidetes_Flavobacteriia_Flavobacteriales_Flavobacteriaceae_Zobellia_uliginosa_MAR_2009_138:0.06243857708769829,Bacteria_BacteroidetesChlorobi_group_Bacteroidetes_Flavobacteriia_Flavobacteriales_Flavobacteriales_bacterium_HTCC2170:0.0584154389351057):0.024131776859676712[99],Bacteria_BacteroidetesChlorobi_group_Bacteroidetes_Flavobacteriia_Flavobacteriales_Flavobacteriaceae_Eudoraea_adriatica_DSM_19308:0.08005951711783776):0.00916454285192847[40],(Bacteria_BacteroidetesChlorobi_group_Bacteroidetes_Flavobacteriia_Flavobacteriales_Flavobacteriaceae_Arenibacter_certesii_DSM_19833:0.0815740452088396,Bacteria_BacteroidetesChlorobi_group_Bacteroidetes_Flavobacteriia_Flavobacteriales_Flavobacteriaceae_Cellulophaga_lytica_HI1:0.060755920396861285):0.01915038598576979[97]):0.018248384151981156[56],(Bacteria_BacteroidetesChlorobi_group_Bacteroidetes_Flavobacteriia_Flavobacteriales_Flavobacteriaceae_Robiginitalea_biformata_HTCC2501:0.09010512576043084,Bacteria_BacteroidetesChlorobi_group_Bacteroidetes_Flavobacteriia_Flavobacteriales_Flavobacteriaceae_Muricauda_ruestringensis_B1_DSM_13258:0.08338314790087109):0.0177863215579932[42]):0.047127543543116435[100],((((Bacteria_BacteroidetesChlorobi_group_Bacteroidetes_Flavobacteriia_Flavobacteriales_Flavobacteriaceae_Capnocytophaga_ochracea_DSM_7271:0.13059710963297055,Bacteria_BacteroidetesChlorobi_group_Bacteroidetes_Flavobacteriia_Flavobacteriales_Flavobacteriaceae_Imtechella_halotolerans_K1:0.05171860742095813):0.02405040540629466[100],'Bacteria_BacteroidetesChlorobi_group_Bacteroidetes_Flavobacteriia_Flavobacteriales_Flavobacteriaceae_Sinomicrobium_oceani_CGMCC_1.12145':0.06353517218720484):0.0188890353212936[99],Bacteria_BacteroidetesChlorobi_group_Bacteroidetes_Flavobacteriia_Flavobacteriales_Flavobacteriaceae_Zhouia_amylolytica_AD3:0.062070083488283245):0.013833940802893796[62],('Bacteria_BacteroidetesChlorobi_group_Bacteroidetes_Flavobacteriia_Flavobacteriales_Flavobacteriaceae_Galbibacter_sp._ck_I2_15':0.05281765320831422,Bacteria_BacteroidetesChlorobi_group_Bacteroidetes_Flavobacteriia_Flavobacteriales_Flavobacteriaceae_Joostella_marina_En5_DSM_19592:0.03500487569193078):0.03800420404904514[100]):0.016290009838179298[97]):0.018379126995833328[99],Bacteria_BacteroidetesChlorobi_group_Bacteroidetes_Flavobacteriia_Flavobacteriales_Flavobacteriaceae_Kordia_algicida_OT_1_unfinished_sequence:0.08030897206381171):0.016780106564614705[98]):0.02388254464295647[97],(((Ga0172382_10004094:0.03816156129978987,Ga0172382_10014595:0.0344390292620278):0.047046271846518106[100],Bacteria_BacteroidetesChlorobi_group_Bacteroidetes_Flavobacteriia_Flavobacteriales_Flavobacteriaceae_Flavobacterium_psychrophilum_JIP0286:0.08277710406443894):0.022887925401074316[98],Bacteria_BacteroidetesChlorobi_group_Bacteroidetes_Flavobacteriia_Flavobacteriales_Flavobacteriaceae_Myroides_odoratimimus_CCUG_12700:0.07780167448609632):0.06125387211095523[100]):0.030479100082871913[97],(('Bacteria_BacteroidetesChlorobi_group_Bacteroidetes_Flavobacteriia_Flavobacteriales_Flavobacteriaceae_Polaribacter_sp._MED152':0.051996729840688705,'Bacteria_BacteroidetesChlorobi_group_Bacteroidetes_Flavobacteriia_Flavobacteriales_Flavobacteriaceae_Lutibacter_sp._Hel_I_33_5':0.03925477842136349):0.02292590402345862[87],Bacteria_BacteroidetesChlorobi_group_Bacteroidetes_Flavobacteriia_Flavobacteriales_Flavobacteriaceae_Tenacibaculum_ovolyticum_DSM_18103:0.061165516415385035):0.08758769951846368[100]):0.0768735175712898[100],(((((((Ga0172382_10003112:0.09619129894742118,Ga0172382_10000282:0.057934581224389436):0.04240269500236682[100],Ga0172382_10000406:0.077702208434272):0.017359498296769527[58],Ga0172382_10000599:0.04820519465135309):0.05154467987704647[100],(Bacteria_BacteroidetesChlorobi_group_Bacteroidetes_Flavobacteriia_Flavobacteriales_Flavobacteriaceae_Empedobacter_brevis_ATCC_43319:0.06500607934724334,Bacteria_BacteroidetesChlorobi_group_Bacteroidetes_Flavobacteriia_Flavobacteriales_Flavobacteriaceae_Weeksella_virosa_DSM_16922:0.07063770668162794):0.04408242981176702[100]):0.06637214115790036[100],Bacteria_BacteroidetesChlorobi_group_Bacteroidetes_Flavobacteriia_Flavobacteriales_Flavobacteriaceae_Ornithobacterium_rhinotracheale_DSM_15997:0.17499167879004052):0.030454814839866895[88],((((Bacteria_BacteroidetesChlorobi_group_Bacteroidetes_Flavobacteriia_Flavobacteriales_Flavobacteriaceae_Epilithonimonas_tenax_DSM_16811:0.06010153635751747,Bacteria_BacteroidetesChlorobi_group_Bacteroidetes_Flavobacteriia_Flavobacteriales_Flavobacteriaceae_Chryseobacterium_gregarium_DSM_19109:0.0463483459857259):0.027281368375908333[100],Bacteria_BacteroidetesChlorobi_group_Bacteroidetes_Flavobacteriia_Flavobacteriales_Flavobacteriaceae_Elizabethkingia_anophelis_NUH6:0.056076191892459715):0.018758914835273366[87],Bacteria_BacteroidetesChlorobi_group_Bacteroidetes_Flavobacteriia_Flavobacteriales_Flavobacteriaceae_Bergeyella_zoohelcum_CCUG_30536:0.06492722176382282):0.02239006654392517[83],Bacteria_BacteroidetesChlorobi_group_Bacteroidetes_Flavobacteriia_Flavobacteriales_Flavobacteriaceae_Riemerella_anatipestifer_RA_CH_2:0.03826047111647668):0.14555340487520896[100]):0.04153124561294863[88],'Bacteria_BacteroidetesChlorobi_group_Bacteroidetes_Flavobacteriia_Flavobacteriales_Blattabacteriaceae_Blattabacterium_sp._Blattella_germanica_str._Bge':0.6032642136181763):0.04344223226503674[99]):0.04977779129383286[100],Bacteria_BacteroidetesChlorobi_group_Bacteroidetes_Flavobacteriia_Flavobacteriales_Cryomorphaceae_Owenweeksia_Owenweeksia_hongkongensis_DSM_17368:0.2523819027553107):0.05421737795497039[99],((Bacteria_BacteroidetesChlorobi_group_Bacteroidetes_Flavobacteriia_Flavobacteriales_Cryomorphaceae_Fluviicola_taffensis_RW262_DSM_16823:0.08001624846821498,Ga0172380_10000442:0.10422595074363983):0.11789543685938053[100],Bacteria_BacteroidetesChlorobi_group_Bacteroidetes_Flavobacteriia_Flavobacteriales_Cryomorphaceae_Crocinitomix_catalasitica_ATCC_23190:0.1852170412314713):0.114056848333568[100]):0.04242167286492737[100]):0.044269401024719546[97],((((((Bacteria_BacteroidetesChlorobi_group_Bacteroidetes_Sphingobacteriia_Sphingobacteriales_Sphingobacteriaceae_Pseudosphingobacterium_domesticum_DSM_18733:0.034798022623964364,'Bacteria_BacteroidetesChlorobi_group_Bacteroidetes_Sphingobacteriia_Sphingobacteriales_Sphingobacteriaceae_Sphingobacterium_sp._21':0.014858249823338276):0.0275922086965954[100],Bacteria_BacteroidetesChlorobi_group_Bacteroidetes_Sphingobacteriia_Sphingobacteriales_Sphingobacteriaceae_Olivibacter_sitiensis_DSM_17696:0.05541117001739071):0.042844252027635754[100],(Bacteria_BacteroidetesChlorobi_group_Bacteroidetes_Sphingobacteriia_Sphingobacteriales_Sphingobacteriaceae_Arcticibacter_svalbardensis_MN12_7:0.08770664633155123,Bacteria_BacteroidetesChlorobi_group_Bacteroidetes_Sphingobacteriia_Sphingobacteriales_Sphingobacteriaceae_Mucilaginibacter_paludis_TPT56_DSM_18603:0.07466566365711325):0.025447190115340135[100]):0.028833256875294033[99],Bacteria_BacteroidetesChlorobi_group_Bacteroidetes_Sphingobacteriia_Sphingobacteriales_Sphingobacteriaceae_Pedobacter_saltans_DSM_12145:0.09873312666143352):0.0474464547221487[100],Bacteria_BacteroidetesChlorobi_group_Bacteroidetes_Sphingobacteriia_Sphingobacteriales_Sphingobacteriaceae_Solitalea_canadensis_USAM_9D_DSM_3403:0.1304826843378737):0.08495497945795538[100],(Ga0172380_10000024:0.14986720091762296,Ga0172379_10000205:0.17214043525588973):0.1249070452029839[100]):0.042329066648864266[97]):0.03232070850261781[77],(((((Bacteria_BacteroidetesChlorobi_group_Bacteroidetes_Sphingobacteriia_Sphingobacteriales_Chitinophagaceae_Terrimonas_ferruginea_DSM_30193:0.06949999164294818,Bacteria_BacteroidetesChlorobi_group_Bacteroidetes_Sphingobacteriia_Sphingobacteriales_Chitinophagaceae_Niabella_soli_JS13_8_DSM_19437:0.12113134407925807):0.07170066572858724[100],Bacteria_BacteroidetesChlorobi_group_Bacteroidetes_Sphingobacteriia_Sphingobacteriales_Chitinophagaceae_Niastella_koreensis_GR20_10_DSM_17620:0.13982759867090122):0.027207951540170416[74],Bacteria_BacteroidetesChlorobi_group_Bacteroidetes_Sphingobacteriia_Sphingobacteriales_Chitinophagaceae_Segetibacter_koreensis_DSM_18137:0.10493330301383352):0.062285772088986224[100],Bacteria_BacteroidetesChlorobi_group_Bacteroidetes_Sphingobacteriia_Sphingobacteriales_Chitinophagaceae_Chitinophaga_pinensis_DSM_2588:0.11674972852455268):0.2350211271601852[100],((Bacteria_BacteroidetesChlorobi_group_Bacteroidetes_Sphingobacteriia_Sphingobacteriales_Saprospiraceae_Saprospira_grandis_Lewin:0.1772161667425789,'Bacteria_BacteroidetesChlorobi_group_Bacteroidetes_Sphingobacteriia_Sphingobacteriales_Saprospiraceae_Aureispira_sp._CCB_QB1':0.20679539202017772):0.12507278540045697[100],(Bacteria_BacteroidetesChlorobi_group_Bacteroidetes_Sphingobacteriia_Sphingobacteriales_Saprospiraceae_Lewinella_cohaerens_DSM_23179:0.20265832840725162,Bacteria_BacteroidetesChlorobi_group_Bacteroidetes_Sphingobacteriia_Sphingobacteriales_Saprospiraceae_Haliscomenobacter_hydrossis_O_DSM_1100:0.21224184982787353):0.06807116867446394[98]):0.10506502762867287[100]):0.06674008975435886[97]):0.035936349995183026[53],(((((((((((((Bacteria_BacteroidetesChlorobi_group_Bacteroidetes_Cytophagia_Cytophagales_Cyclobacteriaceae_Indibacter_alkaliphilus_LW1_Draft1:0.05529926093821924,Bacteria_BacteroidetesChlorobi_group_Bacteroidetes_Cytophagia_Cytophagales_Cyclobacteriaceae_Aquiflexum_balticum_BA160_DSM_16537:0.04487041611122944):0.01309979485347057[52],Bacteria_BacteroidetesChlorobi_group_Bacteroidetes_Cytophagia_Cytophagales_Cyclobacteriaceae_Cecembia_lonarensis_LW9:0.038037969673300864):0.011725836141399881[71],Bacteria_BacteroidetesChlorobi_group_Bacteroidetes_Cytophagia_Cytophagales_Cyclobacteriaceae_Mariniradius_saccharolyticus_AK6:0.0911623305897038):0.020863673172303976[94],(Bacteria_BacteroidetesChlorobi_group_Bacteroidetes_Cytophagia_Cytophagales_Cyclobacteriaceae_Nitritalea_halalkaliphila_LW7_Draft1:0.09620785221749051,'Bacteria_BacteroidetesChlorobi_group_Bacteroidetes_Cytophagia_Cytophagales_Cyclobacteriaceae_Algoriphagus_sp._PR1_unfinished_sequence':0.08354981207663359):0.015087375434831962[56]):0.01333843738742857[40],(Bacteria_BacteroidetesChlorobi_group_Bacteroidetes_Cytophagia_Cytophagales_Cyclobacteriaceae_Belliella_baltica_BA134_DSM_15883:0.04435524950135461,Bacteria_BacteroidetesChlorobi_group_Bacteroidetes_Cytophagia_Cytophagales_Cytophagaceae_Rhodonellum_psychrophilum_DSM_17998:0.07133859433989809):0.01538997829478328[96]):0.018839485587708982[73],Bacteria_BacteroidetesChlorobi_group_Bacteroidetes_Cytophagia_Cytophagales_Cytophagaceae_Anditalea_andensis_LY1:0.06510172458624686):0.01441169640364004[62],Bacteria_BacteroidetesChlorobi_group_Bacteroidetes_Cytophagia_Cytophagales_Cyclobacteriaceae_Cyclobacterium_marinum_DSM_745:0.12087450637097508):0.017512174935513602[46],Bacteria_BacteroidetesChlorobi_group_Bacteroidetes_Cytophagia_Cytophagales_Cyclobacteriaceae_Echinicola_vietnamensis_KMM_6221_DSM_17526:0.06699903749257041):0.11259464969303812[100],(((Bacteria_BacteroidetesChlorobi_group_Bacteroidetes_Cytophagia_Cytophagales_Flammeovirgaceae_Nafulsella_turpanensis_ZLM_10:0.12364903708108477,Bacteria_BacteroidetesChlorobi_group_Bacteroidetes_Cytophagia_Cytophagales_Flammeovirgaceae_Cesiribacter_andamanensis_AMV16:0.14360115392164463):0.07523687610118568[100],(Bacteria_BacteroidetesChlorobi_group_Bacteroidetes_Cytophagia_Cytophagales_Flammeovirgaceae_Marivirga_tractuosa_DSM_4126:0.1924665755000139,Bacteria_BacteroidetesChlorobi_group_Bacteroidetes_Cytophagia_Cytophagales_Flammeovirgaceae_Fulvivirga_imtechensis_AK7:0.14077807116524355):0.03402867750480354[75]):0.021978638350040658[38],(Bacteria_BacteroidetesChlorobi_group_Bacteroidetes_Cytophagia_Cytophagales_Flammeovirgaceae_Flexithrix_dorotheae_DSM_6795:0.22468423622247968,Bacteria_BacteroidetesChlorobi_group_Bacteroidetes_Cytophagia_Cytophagales_Amoebophilaceae_Candidatus_Amoebophilus_asiaticus_5a2:0.35235678849707286):0.03862311073216507[22]):0.02273376258380333[26]):0.030693391374764456[29],(((((Bacteria_BacteroidetesChlorobi_group_Bacteroidetes_Cytophagia_Cytophagales_Cytophagaceae_Dyadobacter_fermentans_DSM_18053:0.12097486876987862,Ga0172382_10006921:0.1637722987850161):0.036007843591557176[95],Bacteria_BacteroidetesChlorobi_group_Bacteroidetes_Cytophagia_Cytophagales_Cytophagaceae_Runella_slithyformis_LSU4_DSM_19594:0.14617005496952462):0.03144660760621276[80],((Bacteria_BacteroidetesChlorobi_group_Bacteroidetes_Cytophagia_Cytophagales_Cytophagaceae_Leadbetterella_byssophila_DSM_17132:0.1715568997372796,Bacteria_BacteroidetesChlorobi_group_Bacteroidetes_Cytophagia_Cytophagales_Cytophagaceae_Emticicia_oligotrophica_GPTSA100_15_DSM_17448:0.12593880735695606):0.10769118055534754[100],Bacteria_BacteroidetesChlorobi_group_Bacteroidetes_Cytophagia_Cytophagales_Cytophagaceae_Flectobacillus_major_VKMB_859_DSM_103:0.1601891607568917):0.028198105713933774[64]):0.026328914672181014[50],(((Bacteria_BacteroidetesChlorobi_group_Bacteroidetes_Cytophagia_Cytophagales_Cytophagaceae_Fibrisoma_limi_BUZ_3:0.05187738881885595,Bacteria_BacteroidetesChlorobi_group_Bacteroidetes_Cytophagia_Cytophagales_Cytophagaceae_Spirosoma_linguale_DSM_74:0.05952273832109789):0.03491249647745809[99],Bacteria_BacteroidetesChlorobi_group_Bacteroidetes_Cytophagia_Cytophagales_Cytophagaceae_Rudanella_lutea_DSM_19387:0.07065791293171175):0.03718615062084796[87],Bacteria_BacteroidetesChlorobi_group_Bacteroidetes_Cytophagia_Cytophagales_Cytophagaceae_Fibrella_aestuarina:0.11066632257469866):0.10111610731684673[100]):0.07381865142804545[100],((('Bacteria_BacteroidetesChlorobi_group_Bacteroidetes_Cytophagia_Cytophagales_Cytophagaceae_Adhaeribacter_aquaticus_MBRG1.5_DSM_16391':0.11823986471788173,Bacteria_BacteroidetesChlorobi_group_Bacteroidetes_Cytophagia_Cytophagales_Cytophagaceae_Pontibacter_roseus_DSM_17521:0.1073687030664261):0.04153120398038279[88],'Bacteria_BacteroidetesChlorobi_group_Bacteroidetes_Cytophagia_Cytophagales_Cytophagaceae_Hymenobacter_sp._APR13':0.14833682377703417):0.10269496020719471[100],(Bacteria_BacteroidetesChlorobi_group_Bacteroidetes_Cytophagia_Cytophagales_Cytophagaceae_Cytophaga_hutchinsonii_ATCC_33406:0.17605347920198477,Bacteria_BacteroidetesChlorobi_group_Bacteroidetes_Cytophagia_Cytophagales_Cytophagaceae_Sporocytophaga_myxococcoides_DSM_11118:0.15797637973271472):0.07350814754847468[100]):0.03199041709844552[74]):0.02158332002995733[50]):0.019794269093974748[13],Bacteria_BacteroidetesChlorobi_group_Bacteroidetes_Cytophagia_Cytophagales_Cytophagaceae_Microscilla_marina_ATCC_23134_unfinished_sequence:0.23974008956562054):0.034209310012394134[37],'Bacteria_BacteroidetesChlorobi_group_Bacteroidetes_Bacteroidetes_Order_III._Incertae_sedis_Thermonema_rossianum_DSM_10300':0.23490178814006324):0.031164866179150597[14],Bacteria_BacteroidetesChlorobi_group_Bacteroidetes_Cytophagia_Cytophagales_Cytophagaceae_Flexibacter_litoralis_Fx_l1_DSM_6794:0.3282913939990486):0.03615842862649554[85]):0.1873056571933427[100],((Bacteria_BacteroidetesChlorobi_group_Bacteroidetes_Sphingobacteriia_Sphingobacteriales_Chitinophagaceae_Balneola_vulgaris_DSM_17893:0.1074441074408452,Bacteria_BacteroidetesChlorobi_group_Bacteroidetes_Sphingobacteriia_Sphingobacteriales_Chitinophagaceae_Gracilimonas_tropica_DSM_19535:0.11761935221939668):0.30343682334402144[100],('Bacteria_BacteroidetesChlorobi_group_Bacteroidetes_Bacteroidetes_Order_II._Incertae_sedis_Rhodothermaceae_Rhodothermus_marinus_DSM_4252':0.23111648276290575,'Bacteria_BacteroidetesChlorobi_group_Bacteroidetes_Bacteroidetes_Order_II._Incertae_sedis_Rhodothermaceae_Salisaeta_longa_DSM_21114':0.2995847400529277):0.1441496158037774[100]):0.06300776115152873[91]):0.07914086899264294[99],((((((((Bacteria_Ignavibacteria_RBG_16_Ignavibacteria_36_9:0.16435454377182257,Bacteria_BacteroidetesChlorobi_group_Ignavibacteria_Ignavibacteria_Ignavibacteriales_Ignavibacteriaceae_Ignavibacterium_album_Mat9_16_JCM_16511:0.11960858324317591):0.04091340620145578[95],(Bacteria_Ignavibacteria_RIFOXYA2_FULL_Ignavibacteria_37_17:0.07433856577688491,Bacteria_Ignavibacteria_RBG_16_Ignavibacteria_34_14:0.06384822471785867):0.08456552547416063[100]):0.01799113990015222[40],(Bacteria_Ignavibacteria_CG_Ignavi_02:0.1333752798038934,Bacteria_Ignavibacteria_RIFOXYD12_FULL_Ignavibacteria_36_8:0.1586024978603402):0.027004912145609072[51]):0.03223543931199613[81],((Bacteria_Ignavibacteria_CG_Ignavi_01:0.033989791050216045,Ga0172379_10003379:0.033110484524001116):0.011653284373900341[41],Ga0172379_10016704:0.02360107251098098):0.1598858413070925[100]):0.051455345059196134[100],(((Bacteria_Ignavibacteria_RIFOXYC2_FULL_Ignavibacteria_38_25:0.012822266448631936,Ga0172380_10000801:0.021562453302022444):0.1288439562932231[100],Bacteria_BacteroidetesChlorobi_group_Ignavibacteria_Ignavibacteria_Ignavibacteriales_Ignavibacteriaceae_Melioribacter_roseus_P3M:0.10573010127870841):0.04994433597292813[100],(Bacteria_Ignavibacteria_GWB2_Ignavibacteria_35_6b:0.1706314070975199,Bacteria_Ignavibacteria_RBG_13_Ignavibacteria_36_8:0.1449928476087372):0.026930876117453373[83]):0.055403294072490006[100]):0.10139548441803292[100],Ga0172380_10001563:0.3837107349454394):0.03011928948420639[47],(((Bacteria_Ignavibacteria_GWA2_Ignavibacteriae_55_25:0.0958745720566756,Bacteria_Ignavibacteria_GWA2_Ignavibacteria_54_16:0.11237842274632337):0.06455523036104793[100],Bacteria_Ignavibacteria_RIFCSPLOWO2_02_FULL_Ignavibacteria_55_14:0.19253813198570668):0.09994391605378938[100],((Ga0172379_10002801:0.018575218943073235,Ga0172379_10014881:0.02787462867724244):0.06816705977340343[100],Ga0172379_10022656:0.094420649618014):0.16316351704159704[100]):0.10215138222726328[100]):0.04649924578535369[46],(((((Bacteria_BacteroidetesChlorobi_group_Chlorobi_Chlorobia_Chlorobiales_Chlorobiaceae_ChlorobiumPelodictyon_group_Chlorobium_chlorochromatii_CaD3:0.12099179261877335,Bacteria_BacteroidetesChlorobi_group_Chlorobi_Chlorobia_Chlorobiales_Chlorobiaceae_ChlorobiumPelodictyon_group_Pelodictyon_luteolum_DSM_273:0.11038914971834801):0.04634027021028775[100],Bacteria_BacteroidetesChlorobi_group_Chlorobi_Chlorobia_Chlorobiales_Chlorobiaceae_Chlorobaculum_parvum_NCIB_8327:0.1234206679621428):0.030539053959474316[89],Bacteria_BacteroidetesChlorobi_group_Chlorobi_Chlorobia_Chlorobiales_Chlorobiaceae_Prosthecochloris_aestuarii_DSM_271:0.1382678342513355):0.17926252048749625[100],Bacteria_BacteroidetesChlorobi_group_Chlorobi_Chlorobia_Chlorobiales_Chlorobiaceae_Chloroherpeton_thalassium_ATCC_35110:0.20507587278602912):0.2544635333749583[100],(((Bacteria_RIF_IGX_GWF2_RIF_IGX_33_9:0.3014459330316259,Ga0172380_10000203:0.33428076359180503):0.03518668674689662[44],Ga0172382_10016034:0.19479265331151385):0.04455393885622083[84],Bacteria_RIF_IGX_RIFOXYC2_FULL_RIF_IGX_35_21:0.23611225445811623):0.2104061423319057[100]):0.045028348450451894[36]):0.06991247325350924[81]):0.0793982158029185[100],((((Ga0172377_10002691:0.07590986644563102,Bacteria_WWE1X_GWF2_WWE1X_40_14:0.07932136335609252):0.1837698676248194[100],Bacteria_CP_WWE1_DOLJORAL78_WWE1_32_13:0.24119675278279962):0.3523271378186341[100],Bacteria_KSB1_uncultured_SMTZ_31:0.49297883311891244):0.054568060629990534[41],(Bacteria_unclassified_Bacteria_Caldithrix_abyssi_LF13_DSM_13497:0.27031063894110074,Bacteria_Caldithrix_RBG_13_Caldithrix_44_9:0.34048080021235183):0.10016710800399586[100]):0.046982555365642664[49]):0.02554384986994318[13],(((((((Ga0172377_10043414:0.000001,Ga0172382_10015259:0.000001):0.04264372053415899[100],(Ga0172377_10019957:0.000899,Ga0172382_10033238:0.000001):0.020790406367813485[100]):0.08702125690142015[100],Ga0172377_10002299:0.10327459438645059):0.27231792788166054[100],Bacteria_Deferribacteres_Deferribacteres_Deferribacterales_CG_Deferri_01:0.41124911153376154):0.06183519016789285[86],((((Bacteria_Marine_group_A_SAR406_SAR406_cluster_bacterium_JGI_0000113_G11:0.16021576522472003,Bacteria_Marine_group_A_SAR406_SAR406_cluster_bacterium_SCGC_AAA076_M08:0.007064835765040023):0.1110659698377412[100],Bacteria_Marine_group_A_SAR406_SAR406_cluster_bacterium_SCGC_AAA298_D23:0.12072963172211804):0.3866183334715503[100],Bacteria_Marine_group_A_SAR406_SAR406_cluster_bacterium_JGI_0000113_D11:0.3555309331808516):0.13052702017589812[100],'Bacteria_Marine_group_A_SAR406_SAR406_cluster_bacterium_sp._SCGC_AAA003_E22_Tropical_gyre_001_268':0.4450674934341996):0.08614382518511965[100]):0.04153268451231629[79],(((((Bacteria_Marine_group_A_SAR406_SAR406_cluster_bacterium_JGI_0000039_D08_TAsludge_001_149:0.000001,Bacteria_Marine_group_A_SAR406_SAR406_cluster_bacterium_JGI_0000059_L03_TAbiofilm_001_169:0.000001):0.000001[100],Bacteria_Marine_group_A_SAR406_SAR406_cluster_bacterium_JGI_0000039_E15_TAsludge_001_150:0.000001):0.005638358437406676[100],Bacteria_Marine_group_A_SAR406_SAR406_cluster_bacterium_JGI_0000039_D08_Combined_Assembly_SAR406_1__SAR406:0.06521224239979162):0.1620013645916205[100],((Ga0172382_10038420:0.0018525131509470327,Ga0172381_10002767:0.000798):0.08188050986885953[100],Ga0172382_10006824:0.07306921008462552):0.09937261135576003[100]):0.09815341875520156[100],Bacteria_Marine_group_A_SAR406_SAR406_cluster_bacterium_SCGC_AAA257_N23_Etoliko_001_146:0.23272484793421722):0.17304469687491464[100]):0.11315133191304749[100],(((Bacteria_KSB1_uncultured_SMTZ_57:0.3211520636470313,Ga0172380_10046808:0.4455650291910249):0.10392645053340432[99],Bacteria_Caldithrix_RBG_16_Caldithrix_48_16:0.3789929397861842):0.044633929881983114[60],Ga0172380_10000843:0.5466807970626686):0.04088666598552715[44]):0.02885532643930011[23]):0.08712613118358359[98],((((((((Bacteria_Zixibacteria_uncultured_SMTZ_73:0.06446827863273041,Bacteria_Zixibacteria_uncultured_SMTZ1_73:0.06546740931704464):0.07982132013430521[100],Bacteria_Zixibacteria_uncultured_SMTZ_73_3:0.15610594046940962):0.10509239147465532[100],(Bacteria_Zixibacteria_uncultured_SMTZ_73_2:0.24364083980121887,Bacteria_Zixibacteria_RBG_16_Zixibacteria_43_9:0.19341533344217998):0.039341845815204834[46]):0.06921865902104862[100],(Bacteria_CP_Zixibacteria_RBG1:0.20712573606486195,Bacteria_Zixibacteria_RBG_16_Zixibacteria_50_21:0.33967680626168173):0.11520201047561729[100]):0.07711424605914408[100],(Bacteria_Zixibacteria_RBG_16_Zixibacteria_53_22:0.4206760190296306,Bacteria_Zixibacteria_uncultured_DG_27:0.3252039496547212):0.06988607025941151[53]):0.03793493108259138[29],Bacteria_CP_TA06_BJP_IG2158_TA06_novel_48_33:0.4620150561480738):0.07021644572843133[99],Bacteria_Zixibacteria_uncultured_SMTZ_81:0.4125905191356538):0.03862867879424625[17],((((Bacteria_CP_WS3_candidate_division_WS3_bacterium_SCGC_AAA252_D10_SAK_001_39:0.17404476139702574,Bacteria_CP_WS3_candidate_division_WS3_bacterium_SCGC_AAA252_E07_SAK_001_42:0.12286841477213573):0.07705311012097749[100],Ga0172379_10025417:0.17176316185110085):0.000001[19],Ga0172379_10039479:0.16093443000433938):0.3073690269732201[100],Bacteria_RIFCSPLOWO2_12_FULL_PLX_64_10:0.5693330903915399):0.06284831648855027[27]):0.03675274821136565[13]):0.030184299754623556[9],(((((((Bacteria_Gemmatimonadetes_RBG_16_Gemmatimonadetes_66_8:0.12809689874972774,Bacteria_Gemmatimonadetes_RIFCSPLOWO2_12_FULL_Gemmatimonadetes_68_9:0.1252629528438134):0.06425445334550428[100],(Bacteria_Gemmatiomonas_uncultured_SG8_28:0.17584544976538563,Bacteria_Gemmatiomonas_uncultured_SG8_17:0.1482845283870322):0.06166066446518359[100]):0.08575576159154208[100],(Bacteria_Gemmatimonadetes_GWC2_Gemmatimonadetes_71_10:0.10095056574321548,Bacteria_Gemmatimonadetes_RIFCSPLOWO2_02_FULL_Gemmatimonadetes_71_11:0.11300191440311114):0.12780509697164932[100]):0.05176308256550799[89],(((Bacteria_Gemmatimonadetes_Gemmatimonadetes_Gemmatimonadales_Gemmatimonadaceae_Gemmatimonas_aurantiaca_T_27:0.0407482033797284,'Bacteria_Gemmatimonadetes_Gemmatimonadetes_Gemmatimonadales_Gemmatimonadaceae_Gemmatimonas_sp._AP64':0.037313452976854844):0.12911132415570759[100],Bacteria_Gemmatimonadetes_Gemmatimonadetes_Gemmatimonadetes_bacterium_KBS708:0.14608807579284244):0.02365615084565542[64],Ga0172379_10035221:0.1344053542900756):0.16811066944128372[100]):0.13257860065239102[100],((((Bacteria_Gemmatimonadetes_Gemmatimonadetes_Gemmatimonadetes_bacterium_SCGC_AB_629_I08:0.01450277269368394,Bacteria_Gemmatimonadetes_Gemmatimonadetes_Gemmatimonadetes_bacterium_SCGC_AAA007_O21:0.0161374928885043):0.14768834035699285[100],Bacteria_Gemmatiomonas_uncultured_SG8_23:0.1130138139560799):0.1017565486346439[100],Bacteria_Gemmatimonadetes_Gemmatimonadetes_Gemmatimonadetes_bacterium_SCGC_AAA003_K10:0.28129777811041823):0.11733499835524164[100],(Bacteria_Gemmatiomonas_uncultured_SMTZ_52:0.12234966918244394,Bacteria_Gemmatiomonas_uncultured_SG8_38_2:0.09265374513309776):0.19481272175507058[100]):0.07945994206672813[100]):0.16133279954062596[100],(Bacteria_RIF5_RBG_16_RIF05_58_8:0.4547618990241107,Bacteria_RIF5_RIFCSPLOWO2_12_FULL_RIF05_58_11:0.43883620765802434):0.046586152884923404[72]):0.1198710912141907[100],(((((Bacteria_WOR_3_RBG_13_WOR_3_43_14:0.22789260471988282,Bacteria_WOR_3_uncultured_SMTZ_60:0.1699504578822726):0.03903139155498403[57],Bacteria_WOR_3_uncultured_SMTZ_42:0.16882873873821413):0.39235880097720344[100],Ga0172381_10040007:0.5727772811240821):0.11044080377770049[82],((Bacteria_TA06_uncultured_SMTZ1_40:0.000001,Bacteria_TA06_uncultured_DG_24:0.000001):0.000001[50],Bacteria_TA06_uncultured_SMTZ_40:0.000001):0.41073442450073694[100]):0.06629353541715854[17],((Bacteria_RIF_WS3X_RBG_16_RIF_WS3X_71_46:0.4186104319746038,Bacteria_WS3_uncultured_DG_63:0.29542570521829425):0.1404878182775109[100],Bacteria_TA06_RIFOXYC12_FULL_TA06_54_24:0.48554804854828326):0.061319571417330465[26]):0.0367688870568581[3]):0.026485864774731915[1]):0.030128090643533945[6],(((((((((((((((Ga0172380_10031592:0.0012351659976324036,Ga0172378_10012130:0.000001):0.000001[74],Ga0172381_10015289:0.000001):0.000586[58],Ga0172382_10023060:0.000535):0.01624081518739562[100],Ga0172378_10028477:0.031262409363761456):0.021919100947946468[99],Ga0172377_10007227:0.03772501912473958):0.025019094730715175[100],(((Ga0172378_10010384:0.010399717322188806,Ga0172377_10042755:0.009508162002909604):0.023594244704714384[90],Ga0172377_10043542:0.038868079620796525):0.01194778734970292[93],Ga0172382_10012075:0.11996150899392166):0.021648181092425833[60]):0.013964607763019998[38],(((Ga0172378_10007287:0.000001,Ga0172377_10001838:0.000001):0.000001[94],Ga0172382_10044704:0.000931):0.045478998455501074[100],(Ga0172381_10005039:0.000619,Ga0172377_10012097:0.000001):0.039015586252471035[100]):0.05511679861131258[100]):0.03458143630812538[71],Ga0172382_10001224:0.13866216307122192):0.04925047525060178[100],(((Ga0172382_10005768:0.000870,Ga0172377_10013776:0.0017234292266876672):0.1583404659676826[100],(Ga0172382_10000689:0.09143463710338562,Ga0172377_10000722:0.0671334946319817):0.040969072061439515[100]):0.033355526519443846[69],Ga0172382_10001309:0.13860745231714144):0.04060164899422958[63]):0.028328372932219903[52],((((Bacteria_CP_WWE1_candidate_division_WWE1_bacterium_JGI_0000059_L07_TAbiofilm_001_170:0.000001,Bacteria_CP_WWE1_candidate_division_WWE1_bacterium_JGI_0000039_G13_Combined_Assembly_WWE1_1__WWE1:0.000001):0.000001[100],Bacteria_CP_WWE1_candidate_division_WWE1_bacterium_JGI_0000039_M09_TAsludge_001_159:0.000001):0.0052070421835170855[100],Bacteria_CP_WWE1_Candidatus_Cloacamonas_acidaminovorans:0.007124895218749483):0.06267128512355136[100],(Ga0172377_10002563:0.000001,Ga0172378_10000401:0.000001):0.06649705757467572[100]):0.06817609896217203[100]):0.2092492200647631[100],(Ga0172382_10006214:0.01246580094218297,Ga0172377_10033400:0.00999710657740005):0.2559414340290225[100]):0.05813570925266287[61],Bacteria_Cloacimonas_DOLZORAL124_Cloacimonas_38_13:0.26112301525913395):0.03947701590143682[60],Ga0172382_10008559:0.23140619168881882):0.18067541823225586[100],(((((Bacteria_CP_KSB1_candidate_division_KSB1_bacterium_SCGC_AAA255_B16_SAK_001_121:0.000001,Bacteria_CP_KSB1_candidate_division_KSB1_bacterium_SCGC_AAA252_F02_SAK_001_45:0.010845843576205105):0.000001[1],Bacteria_CP_KSB1_candidate_division_KSB1_bacterium_SCGC_AAA252_O17_SAK_001_87:0.000001):0.000001[1],(Bacteria_CP_KSB1_candidate_division_KSB1_bacterium_SCGC_AAA252_E13_SAK_001_43:0.000001,Bacteria_CP_KSB1_candidate_division_KSB1_bacterium_SCGC_AAA252_M08_SAK_001_72:0.000001):0.000001[21]):0.02108296250135311[100],(Bacteria_CP_KSB1_candidate_division_KSB1_bacterium_SCGC_AAA252_N14_SAK_002_78:0.000001,Bacteria_CP_KSB1_candidate_division_KSB1_bacterium_SCGC_AAA255_C20_SAK_001_123:0.000001):0.02115544924719881[100]):0.21073255515607237[100],(Bacteria_CP_WWE1_candidate_division_WWE1_bacterium_JGI_0000014_H19_Combined_Assembly_WWE1_4__WWE1:0.000001,Bacteria_CP_WWE1_candidate_division_WWE1_bacterium_JGI_0000014_H19_Etoliko_001_275:0.000001):0.20082414084832223[100]):0.21893020998738377[100]):0.2297447078032433[100],((((((Ga0172377_10004558:0.000685,Ga0172378_10006700:0.001262505318280649):0.09087182878577638[100],(Ga0172377_10003150:0.000001,Ga0172378_10000617:0.000001):0.09149722972808272[100]):0.02259082895707598[83],(Ga0172381_10009720:0.000001,Bacteria_FibrobacteresAcidobacteria_Fibrobacteres_Fibrobacteria_Fibrobacterales_CG_Fibrob_01:0.001764831815773693):0.05402713238195034[100]):0.05950755533707097[100],('Bacteria_FibrobacteresAcidobacteria_group_Fibrobacteres_Fibrobacteria_Fibrobacterales_Fibrobacteraceae_Fibrobacter_succinogenes_subsp._succinogenes_S85':0.0018944520923525054,Bacteria_FibrobacteresAcidobacteria_group_Fibrobacteres_Fibrobacteria_Fibrobacterales_Fibrobacteraceae_Fibrobacter_succinogenes_elongatus_HM2:0.0012189515857099487):0.10033308876354718[100]):0.4585733530073055[100],Bacteria_RIF7_RIFOXYB2_FULL_RIF07_49_35:0.4837760932885775):0.07559925434749548[63],Bacteria_CP_TG3_candidate_division_TG3_bacterium_ACht1:0.5870750807196807):0.11276163722118238[100]):0.07394578799369578[42]):0.061680905772496075[60],Bacteria_CG_CP01_CG_CP01_01:0.5848000321089288):0.028327909638190985[6]):0.019743101031843846[0],((((((((((((((((((((((((((((((((((((((((Bacteria_Proteobacteria_Gammaproteobacteria_Enterobacteriales_Enterobacteriaceae_Escherichia_coli_K12__W3110:0.000440,Bacteria_Proteobacteria_Gammaproteobacteria_Enterobacteriales_Enterobacteriaceae_Shigella_flexneri_2003036:0.0017862701398505543):0.0021716889887093416[99],Bacteria_Proteobacteria_Gammaproteobacteria_Enterobacteriales_Enterobacteriaceae_Cronobacter_sakazakii_CMCC_45402:0.007728626688258355):0.00159223381063045[47],(Bacteria_Proteobacteria_Gammaproteobacteria_Enterobacteriales_Enterobacteriaceae_Citrobacter_rodentium_ICC168:0.003263008348329599,'Bacteria_Proteobacteria_Gammaproteobacteria_Enterobacteriales_Enterobacteriaceae_Salmonella_enterica_Enterica_sv._Heidelberg_41578':0.0020188397884703946):0.0011739634755292272[67]):0.003728971139412174[65],Bacteria_Proteobacteria_Gammaproteobacteria_Enterobacteriales_Enterobacteriaceae_Kluyvera_ascorbata_ATCC_33433:0.012518988290151079):0.0020109268086203613[34],(Bacteria_Proteobacteria_Gammaproteobacteria_Enterobacteriales_Enterobacteriaceae_Lelliottia_amnigena_CHS_78:0.0022294228015948825,Bacteria_Proteobacteria_Gammaproteobacteria_Enterobacteriales_Enterobacteriaceae_Leclercia_adecarboxylata_Leclerc_1783_ATCC_23216:0.007347824710056905):0.009307734633234421[99]):0.0023259389488834437[26],(Bacteria_Proteobacteria_Gammaproteobacteria_Enterobacteriales_Enterobacteriaceae_Klebsiella_oxytoca_HKOPL1:0.004108084600558115,Bacteria_Proteobacteria_Gammaproteobacteria_Enterobacteriales_Enterobacteriaceae_Raoultella_ornithinolytica_B6:0.004830988314661777):0.003535901385453233[81]):0.001956669240946507[4],(Bacteria_Proteobacteria_Gammaproteobacteria_Enterobacteriales_Enterobacteriaceae_Enterobacter_cloacae_SCF1:0.002367223479267544,Bacteria_Proteobacteria_Gammaproteobacteria_Enterobacteriales_Enterobacteriaceae_Trabulsiella_guamensis_ATCC_49490:0.006169754054624832):0.000542[8]):0.0011530709460645383[8],Bacteria_Proteobacteria_Gammaproteobacteria_Enterobacteriales_Enterobacteriaceae_Yokenella_regensburgei_ATCC_43003:0.007418835887464947):0.0028210183907462216[16],Bacteria_Proteobacteria_Gammaproteobacteria_Enterobacteriales_Enterobacteriaceae_Kosakonia_radicincitans_UMEnt0112:0.0076266575619063914):0.009851920140086179[96],(Bacteria_Proteobacteria_Gammaproteobacteria_Enterobacteriales_Enterobacteriaceae_Cedecea_davisae_DSM_4568:0.009589482586985998,Bacteria_Proteobacteria_Gammaproteobacteria_Enterobacteriales_Enterobacteriaceae_Buttiauxella_agrestis_MCE:0.01128453012746311):0.006740694966826499[100]):0.007618174941665057[83],(((Bacteria_Proteobacteria_Gammaproteobacteria_Pseudomonadales_Pseudomonadaceae_Pseudomonas_flectens_ATCC_12775:0.037161936194033185,Bacteria_Proteobacteria_Gammaproteobacteria_Enterobacteriales_Enterobacteriaceae_Tatumella_ptyseos_ATCC_33301:0.017193934563071167):0.006172424983944769[62],Bacteria_Proteobacteria_Gammaproteobacteria_Enterobacteriales_Enterobacteriaceae_Pantoea_ananatis_LMG_5342:0.014374254881728277):0.004572296096759132[56],Bacteria_Proteobacteria_Gammaproteobacteria_Enterobacteriales_Enterobacteriaceae_Erwinia_amylovora_CFBP1430:0.02201666833905458):0.004954896519651086[52]):0.008904013558725499[75],((Bacteria_Proteobacteria_Gammaproteobacteria_Enterobacteriales_Enterobacteriaceae_Lonsdalea_quercina_quercina_NCCB_100489:0.009244097447335786,Bacteria_Proteobacteria_Gammaproteobacteria_Enterobacteriales_Enterobacteriaceae_Dickeya_dadantii_Ech703:0.021838190173841365):0.00841915810723437[87],(Bacteria_Proteobacteria_Gammaproteobacteria_Enterobacteriales_Enterobacteriaceae_Pectobacterium_carotovorum_carotovorum_PCC21:0.010725038009150367,Bacteria_Proteobacteria_Gammaproteobacteria_Enterobacteriales_Enterobacteriaceae_Brenneria_salicis_ATCC_15712:0.009391911669780928):0.004678767210988699[89]):0.007498275758777151[90]):0.005877310050972806[53],(((((Bacteria_Proteobacteria_Gammaproteobacteria_Enterobacteriales_Enterobacteriaceae_unclassified_Enterobacteriaceae_Candidatus_Hamiltonella_defensa_5AT_Acyrthosiphon_pisum:0.17901323581864892,Bacteria_Proteobacteria_Gammaproteobacteria_Enterobacteriales_Enterobacteriaceae_unclassified_Enterobacteriaceae_Candidatus_Regiella_insecticola_LSR1_genomic_scaffold_Scaffold1:0.14122358523229517):0.022924980869241995[26],Bacteria_Proteobacteria_Gammaproteobacteria_Enterobacteriales_Enterobacteriaceae_Yersinia_pestis_Nepal516:0.01418639993435411):0.009045077273641677[31],((Bacteria_Proteobacteria_Gammaproteobacteria_Enterobacteriales_Enterobacteriaceae_Rahnella_aquatilis_HX2:0.007866877825679186,'Bacteria_Proteobacteria_Gammaproteobacteria_Enterobacteriales_Enterobacteriaceae_Ewingella_americana_C.024_ATCC_33852':0.0058649031631632376):0.01753112016006897[100],Bacteria_Proteobacteria_Gammaproteobacteria_Enterobacteriales_Enterobacteriaceae_Serratia_fonticola_RB_25:0.011878016111567913):0.0031122893290569387[30]):0.004691418433464545[21],(Bacteria_Proteobacteria_Gammaproteobacteria_Enterobacteriales_Enterobacteriaceae_Edwardsiella_tarda_C07_087:0.02968243865991349,Bacteria_Proteobacteria_Gammaproteobacteria_Enterobacteriales_Enterobacteriaceae_Hafnia_alvei_FB1:0.01251631721709412):0.0074858337658954[61]):0.007415351751718369[19],(Bacteria_Proteobacteria_Gammaproteobacteria_Enterobacteriales_Enterobacteriaceae_Candidatus_Moranella_endobia_PCIT:0.1470371525114711,Bacteria_Proteobacteria_Gammaproteobacteria_Enterobacteriales_Enterobacteriaceae_Sodalis_glossinidius_morsitans:0.015521243274674301):0.03303789514888189[88]):0.004980759192778184[12]):0.008321679103835411[13],(((Bacteria_Proteobacteria_Gammaproteobacteria_Enterobacteriales_Enterobacteriaceae_Arsenophonus_nasoniae_DSM_15247:0.04077853117576824,Bacteria_Proteobacteria_Gammaproteobacteria_Enterobacteriales_Enterobacteriaceae_Providencia_stuartii_MRSN_2154:0.0219572429023831):0.009279209302092806[55],(Bacteria_Proteobacteria_Gammaproteobacteria_Enterobacteriales_Enterobacteriaceae_Xenorhabdus_nematophila_ATCC_19061:0.02400078681191431,Bacteria_Proteobacteria_Gammaproteobacteria_Enterobacteriales_Enterobacteriaceae_Photorhabdus_luminescens_laumondii_TTO1:0.02052480635634213):0.00736329751440401[62]):0.004870322774930713[38],(Bacteria_Proteobacteria_Gammaproteobacteria_Enterobacteriales_Enterobacteriaceae_Morganella_morganii_morganii_KT:0.03392427299136669,Bacteria_Proteobacteria_Gammaproteobacteria_Enterobacteriales_Enterobacteriaceae_Proteus_mirabilis_WGLW4:0.028546871973742505):0.013351467453939225[92]):0.013332623117384035[96]):0.013345131786661746[15],((Bacteria_Proteobacteria_Gammaproteobacteria_Enterobacteriales_Enterobacteriaceae_Pragia_fontium_DSM_5563:0.010775061292016552,Bacteria_Proteobacteria_Gammaproteobacteria_Enterobacteriales_Enterobacteriaceae_Budvicia_aquatica_DSM_5075:0.01092522280788355):0.013571010932867278[95],Bacteria_Proteobacteria_Gammaproteobacteria_Enterobacteriales_Enterobacteriaceae_Leminorella_grimontii_ATCC_33999:0.025564233233574818):0.029833556548695572[100]):0.01327455242877118[10],((((((Bacteria_Proteobacteria_Gammaproteobacteria_Enterobacteriales_Enterobacteriaceae_Wigglesworthia_glossinidia_endosymbiont_of_Glossina_brevipalpis:0.26244895295287796,Bacteria_Proteobacteria_Gammaproteobacteria_Enterobacteriales_Enterobacteriaceae_Candidatus_Riesia_pediculicola_USDA:0.5003707417389953):0.08422001936016521[97],Bacteria_Proteobacteria_Gammaproteobacteria_Enterobacteriales_Enterobacteriaceae_unclassified_Enterobacteriaceae_Candidatus_Blochmannia_floridanus:0.36653385364301316):0.11100801667736437[100],'Bacteria_Proteobacteria_Gammaproteobacteria_Enterobacteriales_Enterobacteriaceae_Buchnera_Buchnera_aphidicola_str._APS_Acyrthosiphon_pisum':0.1756050286130244):0.06838710308796836[99],Bacteria_Proteobacteria_Gammaproteobacteria_Enterobacteriales_Enterobacteriaceae_Halyomorpha_halys_symbiont:0.2009563587418528):0.056778875262610384[78],Bacteria_Proteobacteria_Gammaproteobacteria_Enterobacteriales_Enterobacteriaceae_Thorsellia_anophelis_DSM_18579:0.12301426639660162):0.02712737430293144[22],(Bacteria_Proteobacteria_Gammaproteobacteria_Orbales_Orbaceae_bacterium_Bimp:0.032713771953909454,Bacteria_Proteobacteria_Gammaproteobacteria_Orbales_Orbaceae_Gilliamella_apicola:0.04541550663853977):0.04595774338777758[100]):0.012167826583750596[6]):0.02037225775634477[97],((((((Bacteria_Proteobacteria_Gammaproteobacteria_Pasteurellales_Pasteurellaceae_Aggregatibacter_actinomycetemcomitans_D7S_1:0.027388929898969572,Bacteria_Proteobacteria_Gammaproteobacteria_Pasteurellales_Pasteurellaceae_Haemophilus_influenzae_KR494:0.0292840229206055):0.005815012642942374[58],Bacteria_Proteobacteria_Gammaproteobacteria_Pasteurellales_Pasteurellaceae_Pasteurella_multocida_43137:0.015612560229782524):0.004839455349725075[42],Bacteria_Proteobacteria_Gammaproteobacteria_Pasteurellales_Pasteurellaceae_Mannheimia_succiniciproducens_MBEL55E:0.02979916629129553):0.005103359414492026[47],(Bacteria_Proteobacteria_Gammaproteobacteria_Pasteurellales_Pasteurellaceae_Histophilus_somni_129P:0.0326733835373747,Bacteria_Proteobacteria_Gammaproteobacteria_Pasteurellales_Pasteurellaceae_Avibacterium_paragallinarum_AVPAR72:0.017946603555209162):0.0060706670455483724[35]):0.01037517322241266[49],((Bacteria_Proteobacteria_Gammaproteobacteria_Pasteurellales_Pasteurellaceae_Mannheimia_haemolytica_D174:0.013266492117814721,Bacteria_Proteobacteria_Gammaproteobacteria_Pasteurellales_Pasteurellaceae_Bibersteinia_trehalosi_USDA_ARS_USMARC_189:0.028532605740733796):0.010096911703457945[98],'Bacteria_Proteobacteria_Gammaproteobacteria_Pasteurellales_Pasteurellaceae_Actinobacillus_pleuropneumoniae_serovar_3_str._JL03':0.021861905694907424):0.018939657173423274[100]):0.023691808077437315[100],Bacteria_Proteobacteria_Gammaproteobacteria_Pasteurellales_Pasteurellaceae_Gallibacterium_anatis_UMN179:0.04010862578743124):0.06257877732898409[100]):0.011472104712409337[47],'Bacteria_Proteobacteria_Gammaproteobacteria_Enterobacteriales_Enterobacteriaceae_Plesiomonas_sp._ZOR0011':0.0539335617576393):0.05204877771532468[100],(((('Bacteria_Proteobacteria_Gammaproteobacteria_Vibrionales_Vibrionaceae_Enterovibrio_sp._AK16':0.013091259514097509,Bacteria_Proteobacteria_Gammaproteobacteria_Vibrionales_Vibrionaceae_Enterovibrio_norvegicus_FF_162:0.028014683144730324):0.02725507889548373[100],Bacteria_Proteobacteria_Gammaproteobacteria_Vibrionales_Vibrionaceae_Salinivibrio_costicola_costicola_ATCC_33508:0.05853926791155217):0.015997360335655575[69],Bacteria_Proteobacteria_Gammaproteobacteria_Vibrionales_Vibrionaceae_Photobacterium_profundum_SS9:0.068523789080142):0.022159272965690313[92],((Bacteria_Proteobacteria_Gammaproteobacteria_Vibrionales_Vibrionaceae_Candidatus_Photodesmus_katoptron_Akat1:0.24603493731679782,Bacteria_Proteobacteria_Gammaproteobacteria_Vibrionales_Vibrionaceae_Vibrio_anguillarum_775_I:0.029140847905264167):0.0187712061896379[97],Bacteria_Proteobacteria_Gammaproteobacteria_Vibrionales_Vibrionaceae_Vibrio_fischeri_MJ11_I:0.04458808167240136):0.02520482456562423[99]):0.044194780822320645[100]):0.03422316636563627[99],((((((Bacteria_Proteobacteria_Gammaproteobacteria_Aeromonadales_Succinivibrionaceae_Succinivibrio_dextrinosolvens_ACV_10:0.12081398150607647,'Bacteria_Proteobacteria_Gammaproteobacteria_Aeromonadales_Succinivibrionaceae_Succinatimonas_hippei_YIT_12066_S_hippeiYIT12066_1.0_Cont1069.1':0.07961016327651027):0.029692161809638407[79],Bacteria_Proteobacteria_Gammaproteobacteria_Aeromonadales_Succinivibrionaceae_Anaerobiospirillum_succiniciproducens_DSM_6400:0.06966729623148193):0.12930041707035977[100],(('Bacteria_Proteobacteria_Gammaproteobacteria_Aeromonadales_Succinivibrionaceae_Ruminobacter_sp._RM87':0.10868169606001032,Bacteria_Proteobacteria_Gammaproteobacteria_Aeromonadales_Succinivibrionaceae_Succinimonas_amylolytica_DSM_2873:0.13019565273936484):0.02848284345097074[51],Bacteria_Proteobacteria_Gammaproteobacteria_Aeromonadales_Succinivibrionaceae_WG_1_Wallaby_Group_1_isolate:0.07560951835652974):0.06779780816254544[100]):0.15446329688412863[100],Bacteria_Proteobacteria_Gammaproteobacteria_Aeromonadales_Aeromonadaceae_Tolumonas_auensis_DSM_9187:0.05634597467563962):0.03782438406695521[99],(Bacteria_Proteobacteria_Gammaproteobacteria_Aeromonadales_Aeromonadaceae_Aeromonas_hydrophila_AL09_71:0.005110361877726177,Ga0172380_10012504:0.008085506224065231):0.05826004958309472[100]):0.029014692177377732[100],'Bacteria_Proteobacteria_Gammaproteobacteria_Aeromonadales_Aeromonadaceae_Oceanimonas_sp._GK1':0.0711289704628233):0.023433249216052054[99]):0.016633929809282577[91],((((Ga0172380_10003013:0.000001,Ga0172382_10006854:0.000001):0.01234237393848936[100],Bacteria_Proteobacteria_Gammaproteobacteria_Alteromonadales_Shewanellaceae_Shewanella_oneidensis_MR_1:0.010984539634180113):0.12663163531724475[100],Bacteria_Proteobacteria_Gammaproteobacteria_Alteromonadales_Ferrimonadaceae_Ferrimonas_balearica_DSM_9799:0.08927172160884567):0.025042961492907967[79],((Bacteria_Proteobacteria_Gammaproteobacteria_Alteromonadales_Psychromonadaceae_Psychromonas_ingrahamii_37:0.13933954731568488,Bacteria_Proteobacteria_Gammaproteobacteria_Alteromonadales_Moritellaceae_Moritella_marina_ATCC_15381:0.1163445999077406):0.03706564546574498[98],Bacteria_Proteobacteria_Gammaproteobacteria_Alteromonadales_Alteromonadaceae_Agarivorans_albus_MKT_106:0.09928712274496077):0.029704515590580627[95]):0.008711048862994453[54]):0.02299507147278801[86],Bacteria_Proteobacteria_Gammaproteobacteria_unclassified_Gammaproteobacteria_Gallaecimonas_xiamenensis_3_C_1:0.09175511746190201):0.026364018006009182[98],(((((((Bacteria_Proteobacteria_Gammaproteobacteria_Alteromonadales_Alteromonadaceae_Alteromonas_macleodii_Black_Sea_11:0.034836273863343425,Bacteria_Proteobacteria_Gammaproteobacteria_Alteromonadales_Alteromonadaceae_Salinimonas_chungwhensis_DSM_16280:0.057946567621073974):0.022164887393116395[98],Bacteria_Proteobacteria_Gammaproteobacteria_Alteromonadales_Alteromonadaceae_Glaciecola_nitratireducens_FR1064:0.07784142606267608):0.008968447864924123[55],Bacteria_Proteobacteria_Gammaproteobacteria_Alteromonadales_Alteromonadaceae_Aestuariibacter_salexigens_DSM_15300:0.03955666720376305):0.020171559930123095[77],Bacteria_Proteobacteria_Gammaproteobacteria_Alteromonadales_Pseudoalteromonadaceae_Pseudoalteromonas_atlantica_T6c:0.06086631067134629):0.07873228060723836[100],Bacteria_Proteobacteria_Gammaproteobacteria_Alteromonadales_Idiomarinaceae_Idiomarina_loihiensis_L2TR:0.1207816038575582):0.023163183517434405[71],((Bacteria_Proteobacteria_Gammaproteobacteria_Alteromonadales_Alteromonadaceae_Catenovulum_agarivorans_YM01:0.040368113054254806,Bacteria_Proteobacteria_Gammaproteobacteria_Alteromonadales_Alteromonadaceae_Gayadomonas_joobiniege_G7:0.03580132097786004):0.1090759031118691[100],Bacteria_Proteobacteria_Gammaproteobacteria_Alteromonadales_Pseudoalteromonadaceae_Algicola_sagamiensis_DSM_14643:0.09615318328359779):0.01792696653914483[38]):0.017113079942219223[51],((Bacteria_Proteobacteria_Gammaproteobacteria_Alteromonadales_Colwelliaceae_Colwellia_psychrerythraea_34H:0.09340213617235005,Bacteria_Proteobacteria_Gammaproteobacteria_Alteromonadales_Colwelliaceae_Thalassotalea_agarivorans_DSM_19706:0.048379440562025255):0.10022119091716863[100],(Bacteria_Proteobacteria_Gammaproteobacteria_Alteromonadales_Alteromonadaceae_Alishewanella_agri_BL06:0.025762236765568325,Bacteria_Proteobacteria_Gammaproteobacteria_Chromatiales_Chromatiaceae_Rheinheimera_nanhaiensis_E407_8:0.017199347103933338):0.1090548560733211[100]):0.029527692423139573[83]):0.015360867437463455[81]):0.07463346032777762[100],Bacteria_Proteobacteria_Gammaproteobacteria_Oceanospirillales_Alcanivoracaceae_Kangiella_koreensis_DSM_16069:0.21703834962712953):0.052928506490673044[98],((((((((((Bacteria_Proteobacteria_Gammaproteobacteria_Alteromonadales_Alteromonadaceae_Marinimicrobium_agarilyticum_DSM_16975:0.07144511869003445,Bacteria_Proteobacteria_Gammaproteobacteria_Alteromonadales_Alteromonadales_genera_incertae_sedis_Gilvimarinus_chinensis_DSM_19667:0.062404458517728134):0.02301302097825486[88],Bacteria_Proteobacteria_Gammaproteobacteria_Pseudomonadales_Pseudomonadaceae_Cellvibrio_japonicus_Ueda107:0.08369667612364351):0.036632592824935806[100],Bacteria_Proteobacteria_Gammaproteobacteria_unclassified_Gammaproteobacteria_Simiduia_agarivorans_SA1:0.09108652351784619):0.018199288026928162[76],((('Bacteria_Proteobacteria_Gammaproteobacteria_Pseudomonadales_B._setacea_BS12':0.05336643436520694,Bacteria_Proteobacteria_Gammaproteobacteria_Alteromonadales_Alteromonadales_genera_incertae_sedis_Teredinibacter_turnerae_T7902:0.0579619228251782):0.030585763211978367[100],Bacteria_Proteobacteria_Gammaproteobacteria_Alteromonadales_Alteromonadaceae_Saccharophagus_degradans_2_40:0.07564226897711057):0.04490449929950291[100],'Bacteria_Proteobacteria_Gammaproteobacteria_Alteromonadales_Alteromonadaceae_Alteromonas_sp._S89':0.10300152469520318):0.015523393526217344[60]):0.03439347557220174[98],((((Ga0172382_10007517:0.002362436515510602,Ga0172377_10052151:0.028182902303549362):0.03633044712050948[100],Ga0172382_10006791:0.05233388822695373):0.05115807261135563[100],(Ga0172382_10010125:0.05322578558682789,Ga0172382_10009326:0.05495075650482173):0.04790634103586111[100]):0.06826828891858616[100],Bacteria_Proteobacteria_Gammaproteobacteria_unclassified_Gammaproteobacteria_Porticoccus_hydrocarbonoclasticus_MCTG13d:0.10422094413947791):0.05075594389138072[100]):0.02523658410817342[97],((((Bacteria_Proteobacteria_Gammaproteobacteria_Alteromonadales_Alteromonadaceae_Haliea_rubra_CM41_15a_DSM_19751:0.09562149927838037,Bacteria_Proteobacteria_Gammaproteobacteria_unclassified_Gammaproteobacteria_OMG_group_OM60_clade_Congregibacter_litoralis_KT71:0.10919739768205439):0.029956272670533135[97],Bacteria_Proteobacteria_Gammaproteobacteria_Alteromonadales_Alteromonadaceae_Haliea_salexigens_DSM_19537:0.08666021779588018):0.025989623710248377[97],Bacteria_Proteobacteria_Gammaproteobacteria_gamma_proteobacterium_NOR51_B_scf_1109846220923_genomic_scaffold:0.14010470694329502):0.06932716159793939[100],((Ga0172382_10000823:0.12842642224876277,Bacteria_Proteobacteria_Gammaproteobacteria_Pseudomonadales_unclassified_Pseudomonadales_Dasania_marina_DSM_21967:0.10135409048702915):0.05160407594649241[99],(Bacteria_Proteobacteria_Gammaproteobacteria_unclassified_Gammaproteobacteria_Spongiibacter_tropicus_DSM_19543:0.026202935222905843,Bacteria_Proteobacteria_Gammaproteobacteria_Alteromonadales_Alteromonadaceae_Melitea_salexigens_DSM_19753:0.02840510164767851):0.08757467140663833[100]):0.03136503151879122[94]):0.05415768709311486[96]):0.03442290122527503[76],(Ga0172380_10015246:0.20839331802132444,Bacteria_Proteobacteria_Gammaproteobacteria_Oceanospirillales_Oceanospirillales_bacterium_SCGC_AAA298_N10:0.5649710956917007):0.07475790014358319[36]):0.03262379053417375[33],((((Ga0172382_10008256:0.031119133647751873,Bacteria_Proteobacteria_Gammaproteobacteria_Pseudomonadales_Pseudomonadaceae_Azotobacter_group_Azotobacter_vinelandii_CA6:0.03529885865140159):0.018685526823533305[91],Bacteria_Proteobacteria_Gammaproteobacteria_Pseudomonadales_Pseudomonadaceae_Pseudomonas_aeruginosa_B136_33:0.040545418254755106):0.02991497217082184[100],(Ga0172378_10038911:0.04515066194230499,Ga0172382_10001832:0.06938367341389728):0.035962431342194456[100]):0.11066959020638789[100],(Bacteria_Proteobacteria_Gammaproteobacteria_Oceanospirillales_Hahellaceae_Endozoicomonas_montiporae_LMG_24815:0.1336906608279831,Bacteria_Proteobacteria_Gammaproteobacteria_Oceanospirillales_Hahellaceae_Zooshikella_ganghwensis_DSM_15267:0.10976691921013915):0.03897090621334787[100]):0.03963887584222636[100]):0.020141412210056053[9],((((((((Bacteria_Proteobacteria_Gammaproteobacteria_Oceanospirillales_Oceanospirillaceae_Amphritea_japonica_ATCC_BAA_1530:0.09374858081672155,Bacteria_Proteobacteria_Gammaproteobacteria_Oceanospirillales_Oceanospirillaceae_Neptuniibacter_caesariensis_MED92:0.06860124651992905):0.024291403740178463[83],(Bacteria_Proteobacteria_Gammaproteobacteria_Oceanospirillales_Oceanospirillaceae_Nitrincola_lacisaponensis_4CA:0.1057568530637587,Bacteria_Proteobacteria_Gammaproteobacteria_Alteromonadales_Alteromonadaceae_Marinobacterium_litorale_DSM_23545:0.06802181802553342):0.042532778471862986[100]):0.01671087274699401[71],Bacteria_Proteobacteria_Gammaproteobacteria_Oceanospirillales_Oceanospirillaceae_Neptunomonas_japonica_DSM_18939:0.10119790033537157):0.05713674981637862[100],Bacteria_Proteobacteria_Gammaproteobacteria_Oceanospirillales_Oceanospirillaceae_Balneatrix_alpica_DSM_16621:0.1662624478885566):0.024966846084355954[81],(((Bacteria_Proteobacteria_Gammaproteobacteria_Oceanospirillales_Oceanospirillaceae_Thalassolituus_oleivorans_R6_15:0.057632759079794926,Bacteria_Proteobacteria_Gammaproteobacteria_Oceanospirillales_Oceanospirillaceae_Oceanobacter_kriegii_DSM_6294:0.05852111163707052):0.08730944083314629[100],'Bacteria_Proteobacteria_Gammaproteobacteria_Oceanospirillales_Oceanospirillaceae_Oceanobacter_sp._RED65_unfinished_sequence':0.1298439667401703):0.062208035412877116[100],(Bacteria_Proteobacteria_Gammaproteobacteria_unclassified_Gammaproteobacteria_Reinekea_blandensis_MED297:0.1019081863145539,Bacteria_Proteobacteria_Gammaproteobacteria_Oceanospirillales_Saccharospirillaceae_Saccharospirillum_impatiens_DSM_12546:0.10240161754957722):0.09212758035233337[100]):0.04770374435192748[100]):0.020255342656334996[67],Bacteria_Proteobacteria_Gammaproteobacteria_Oceanospirillales_Oceanospirillaceae_Marinomonas_mediterranea_MMB_1:0.17940281256775004):0.019174401332058277[51],(((((Bacteria_Proteobacteria_Gammaproteobacteria_Oceanospirillales_Halomonadaceae_Halotalea_alkalilenta_DSM_17697:0.028714517933166928,Bacteria_Proteobacteria_Gammaproteobacteria_Oceanospirillales_Halomonadaceae_Carnimonas_nigrificans_ATCC_BAA_78:0.053176752620474055):0.025854498665164982[100],Bacteria_Proteobacteria_Gammaproteobacteria_Oceanospirillales_Halomonadaceae_Zymobacter_group_Zymobacter_palmae_DSM_10491:0.06186966753214396):0.030104721534930157[100],Bacteria_Proteobacteria_Gammaproteobacteria_Oceanospirillales_Halomonadaceae_Kushneria_aurantia_DSM_21353:0.1098718112229724):0.025212466904106723[100],((Bacteria_Proteobacteria_Gammaproteobacteria_Oceanospirillales_Halomonadaceae_Halomonas_elongata_DSM_2581:0.05591775613016825,Bacteria_Proteobacteria_Gammaproteobacteria_Oceanospirillales_Halomonadaceae_Chromohalobacter_salexigens_1H11_DSM_3043:0.06391344095362905):0.02006450537343829[98],Bacteria_Proteobacteria_Gammaproteobacteria_Oceanospirillales_Halomonadaceae_Cobetia_marina_KMM_296:0.08271889853693581):0.014151780330851071[65]):0.07317518140951451[100],(Bacteria_Proteobacteria_Gammaproteobacteria_Oceanospirillales_Oceanospirillaceae_Marinospirillum_insulare_DSM_21763:0.15448972868713717,Bacteria_Proteobacteria_Gammaproteobacteria_Oceanospirillales_Oceanospirillaceae_Oceanospirillum_maris_DSM_6286:0.10645628226454296):0.028262573148127856[72]):0.07767502544907234[100]):0.020979790035277723[65],Bacteria_Proteobacteria_Gammaproteobacteria_Oceanospirillales_Hahellaceae_Hahella_chejuensis_KCTC_2396:0.2052899477958805):0.025852091263328436[76]):0.02912023986546508[24],(((((Bacteria_Proteobacteria_Gammaproteobacteria_Pseudomonadales_Moraxellaceae_Psychrobacter_cryohalolentis_K5:0.08941712778755972,Bacteria_Proteobacteria_Gammaproteobacteria_Pseudomonadales_Moraxellaceae_Moraxella_Moraxella_Moraxella_catarrhalis_25240:0.07454578145181356):0.034113714962241914[72],Bacteria_Proteobacteria_Gammaproteobacteria_Pseudomonadales_Moraxellaceae_Enhydrobacter_aerosaccus_SK60:0.07679225989413618):0.12433141676620973[100],(Bacteria_Proteobacteria_Gammaproteobacteria_Pseudomonadales_Moraxellaceae_Acinetobacter_baumannii_AB30:0.07093426854394247,Bacteria_Proteobacteria_Gammaproteobacteria_Pseudomonadales_Moraxellaceae_Alkanindiges_illinoisensis_DSM_15370:0.05856254481145973):0.04395494842467196[98]):0.06831437160362963[100],Bacteria_Proteobacteria_Gammaproteobacteria_Pseudomonadales_Moraxellaceae_Perlucidibaca_piscinae_DSM_21586:0.11565986759859559):0.12769067503733922[100],Bacteria_Proteobacteria_Gammaproteobacteria_Oceanospirillales_Alcanivoracaceae_Alcanivorax_borkumensis_SK2:0.18165954158761544):0.08004790058033961[100]):0.04706680530303364[52]):0.030960942071181385[51],Bacteria_Proteobacteria_Gammaproteobacteria_Thiotrichales_Piscirickettsiaceae_Piscirickettsia_salmonis_LF_89:0.24314090455172588):0.0251641190683487[28],((((((Bacteria_Proteobacteria_Gammaproteobacteria_Thiotrichales_Piscirickettsiaceae_Thiomicrospira_CG_Thiomicrospira_01:0.07831910192478908,Bacteria_Proteobacteria_Gammaproteobacteria_Thiotrichales_Piscirickettsiaceae_Thiomicrospira_crunogena_XCL_2:0.04100643779989932):0.040145915468125626[100],Bacteria_Proteobacteria_Gammaproteobacteria_Thiotrichales_Piscirickettsiaceae_Hydrogenovibrio_marinus_MH_110:0.07952013377097034):0.0833283145958319[100],(Bacteria_Proteobacteria_Gammaproteobacteria_Thiotrichales_Piscirickettsiaceae_Thioalkalimicrobium_cyclicum_ALM1:0.07568239073529659,Ga0172377_10002145:0.09379994984568762):0.06993321362109572[100]):0.1687513455732481[100],'Bacteria_Proteobacteria_Gammaproteobacteria_unclassified_Gammaproteobacteria_Candidatus_Ruthia_magnifica_str._Cm_Calyptogena_magnifica':0.4066021957254278):0.045308568260679305[54],('Bacteria_Proteobacteria_Gammaproteobacteria_Thiotrichales_Francisellaceae_Francisella_philomiragia_subsp._philomiragia_ATCC_25017':0.2053562191762297,Bacteria_Proteobacteria_Gammaproteobacteria_Thiotrichales_Francisellaceae_Francisella_FSC776:0.1475731239691136):0.16969437236300688[100]):0.039073859259123545[33],(((Bacteria_Proteobacteria_Gammaproteobacteria_Legionellales_Coxiellaceae_Rickettsiella_grylli:0.20672590746472386,Bacteria_Proteobacteria_Gammaproteobacteria_Legionellales_Coxiellaceae_Diplorickettsia_massiliensis_20B:0.19533758929361422):0.27990570080744126[100],(Bacteria_Proteobacteria_Gammaproteobacteria_Legionellales_Coxiellaceae_Coxiella_burnetii_CbuK_Q154:0.28299066219093927,Ga0172379_10021597:0.33721465629472336):0.04368825905741458[85]):0.03317018167092112[76],(Bacteria_Proteobacteria_Gammaproteobacteria_Legionellales_Legionellaceae_Legionella_micdadei_ATCC_33218:0.0975420048317388,Bacteria_Proteobacteria_Gammaproteobacteria_Legionellales_Legionellaceae_Fluoribacter_dumoffii_Tex_KL_ATCC_33343:0.10525446782471848):0.3451254914747808[100]):0.05029012735990701[65]):0.02362387797597254[16]):0.030596748371107818[81],((((((((Ga0172379_10024831:0.01262316110809758,Bacteria_Proteobacteria_Gammaproteobacteria_Methylococcales_Methylococcaceae_Methylobacter_tundripaludum_2122:0.013805110893291062):0.007662842206923148[66],Ga0172379_10009056:0.03006920814461944):0.045336344232023595[100],(Bacteria_Proteobacteria_Gammaproteobacteria_Methylococcales_Methylococcaceae_Methyloglobulus_morosus_KoM1:0.11315182403686075,Bacteria_Proteobacteria_Gammaproteobacteria_Methylococcales_Methylococcaceae_Methylovulum_miyakonense_HT12:0.07628420425787663):0.021580246432649997[48]):0.0240650659586783[60],Bacteria_Proteobacteria_Gammaproteobacteria_Methylococcales_Methylococcaceae_Methylosarcina_lacus_LW14:0.09528461909466124):0.06360426179390721[100],(Bacteria_Proteobacteria_Gammaproteobacteria_Methylococcales_Methylococcaceae_Methylomonas_mecanica_MC09:0.12460706749484496,Bacteria_Proteobacteria_Gammaproteobacteria_Methylococcales_Methylococcaceae_Methylomarinum_vadi_IT_4:0.09242438378704865):0.05384904765949239[100]):0.11439820695344816[100],((Bacteria_Proteobacteria_Gammaproteobacteria_Methylococcales_Methylococcaceae_Methylocaldum_szegediense_O_12:0.1673988675190552,Bacteria_Proteobacteria_Gammaproteobacteria_Methylococcales_Methylococcaceae_Methylococcus_capsulatus_Bath:0.2510176297251445):0.08335000513465696[100],Bacteria_Proteobacteria_Gammaproteobacteria_Methylococcales_Methylococcaceae_Methylohalobius_crimeensis_10Ki:0.2649891093603678):0.03790830134526679[86]):0.04963798915328477[84],'Bacteria_Proteobacteria_Gammaproteobacteria_Thiotrichales_Piscirickettsiaceae_Cycloclasticus_sp._P1':0.3048633455466385):0.07244764909681312[100],(Bacteria_Proteobacteria_Gammaproteobacteria_Chromatiales_Chromatiaceae_Nitrosococcus_oceani_C_107_ATCC_19707:0.303712665823606,Bacteria_Proteobacteria_Gammaproteobacteria_Thiotrichales_Piscirickettsiaceae_Methylophaga_frappieri_JAM7:0.25003888872444957):0.07369322322997274[98]):0.03367069370096276[62]):0.021116873063526498[30],((((((Bacteria_Proteobacteria_Gammaproteobacteria_Chromatiales_Chromatiaceae_Thiocapsa_marina_5811_DSM_5653:0.10851878692989647,Bacteria_Proteobacteria_Gammaproteobacteria_Chromatiales_Chromatiaceae_Thiocystis_violascens_611_DSM_198:0.06073476483750451):0.02354102160403393[65],(Bacteria_Proteobacteria_Gammaproteobacteria_Chromatiales_Chromatiaceae_Allochromatium_vinosum_DSM_180:0.07720177226565195,Bacteria_Proteobacteria_Gammaproteobacteria_Chromatiales_Chromatiaceae_Thiorhodococcus_drewsii_AZ1:0.05124339704569003):0.014057479558933661[83]):0.028475093139648244[90],Bacteria_Proteobacteria_Gammaproteobacteria_Chromatiales_Chromatiaceae_Marichromatium_purpuratum_987:0.07391069683405949):0.03211596126746841[99],Bacteria_Proteobacteria_Gammaproteobacteria_Chromatiales_Chromatiaceae_Lamprocystis_purpurea_DSM_4197:0.14939278511331233):0.02833952433737874[93],('Bacteria_Proteobacteria_Gammaproteobacteria_Chromatiales_Chromatiaceae_Thiorhodovibrio_sp._970':0.21131920381539926,Bacteria_Proteobacteria_Gammaproteobacteria_Chromatiales_Chromatiaceae_Thioflavicoccus_mobilis_8321:0.13287265716914076):0.03573738113336766[81]):0.1082682909606647[100],Bacteria_Proteobacteria_Gammaproteobacteria_unclassified_Gammaproteobacteria_Dechloromarinus_chlorophilus_NSS:0.1792853196996984):0.07451606274747213[100]):0.015615993058516686[14],((((Ga0172382_10004261:0.08086143652827982,Ga0172382_10030854:0.0904682280002378):0.05764803362361315[100],Bacteria_Proteobacteria_Gammaproteobacteria_Thiothrichales_uncultured_SG8_50:0.1503750984591532):0.07871912610458187[100],Ga0172382_10027854:0.22974343092088167):0.025279430303858152[59],('Bacteria_Proteobacteria_Gammaproteobacteria_Thiotrichales_Thiotrichaceae_Thiomargarita_sp._Thio36':0.1526475825373379,'Bacteria_Proteobacteria_Gammaproteobacteria_Thiotrichales_Thiotrichaceae_Beggiatoa_sp._Orange_Guaymas':0.1950923786199379):0.1788470574337877[100]):0.03257106161037493[33]):0.015057982842753503[15],(((((((((((((Bacteria_Proteobacteria_Gammaproteobacteria_Xanthomonadales_Xanthomonadaceae_Xanthomonas_axonopodis_Xac29_1:0.003944393351101638,Bacteria_Proteobacteria_Gammaproteobacteria_Xanthomonadales_Xanthomonadaceae_Xanthomonas_oryzae_ATCC_35933:0.013803821984483822):0.031288933888586[100],(Bacteria_Proteobacteria_Gammaproteobacteria_Xanthomonadales_Xanthomonadaceae_Stenotrophomonas_maltophilia_K279a:0.00459422609495963,Bacteria_Proteobacteria_Gammaproteobacteria_Xanthomonadales_Xanthomonadaceae_Stenotrophomonas_maltophilia_ATCC_19867:0.003388087212942814):0.04842336272443015[100]):0.01438783586213166[54],(Bacteria_Proteobacteria_Gammaproteobacteria_Xanthomonadales_Xanthomonadaceae_Xylella_fastidiosa_sandyi_Ann_1:0.008369220155493018,Bacteria_Proteobacteria_Gammaproteobacteria_Xanthomonadales_Xanthomonadaceae_Xylella_fastidiosa_multiplex_ATCC_35871:0.009627691972305286):0.23618134270172852[100]):0.02706827793113442[100],(Bacteria_Proteobacteria_Gammaproteobacteria_Xanthomonadales_RIFOXYA1_FULL_Xanthomonadales_68_6:0.042347983345742524,Bacteria_Proteobacteria_Gammaproteobacteria_Xanthomonadales_Xanthomonadaceae_Pseudoxanthomonas_spadix_BD_a59:0.06827448716703621):0.028541677981949842[100]):0.031003023475070002[100],((('Bacteria_Proteobacteria_Gammaproteobacteria_Xanthomonadales_Xanthomonadaceae_Luteimonas_sp._J16':0.07081112029473058,Bacteria_Proteobacteria_Gammaproteobacteria_Xanthomonadales_Xanthomonadaceae_Luteimonas_mephitis_DSM_12574:0.060146337485496915):0.0224570641647186[99],Bacteria_Proteobacteria_Gammaproteobacteria_Xanthomonadales_Xanthomonadaceae_Thermomonas_fusca_DSM_15424:0.11037245515451888):0.02080990310366504[74],(Bacteria_Proteobacteria_Gammaproteobacteria_Xanthomonadales_Xanthomonadaceae_Lysobacter_defluvii_DSM_18482:0.10042806708994334,Bacteria_Proteobacteria_Gammaproteobacteria_Xanthomonadales_Xanthomonadaceae_Lysobacter_antibioticus_HS124:0.05683428300344273):0.024006247859595042[88]):0.010527363803848555[45]):0.05040891284923665[100],(((Bacteria_Proteobacteria_Gammaproteobacteria_Xanthomonadales_Xanthomonadaceae_Arenimonas_oryziterrae_DSM_21050:0.037821623318905484,Ga0172379_10032866:0.05474792910234694):0.05041526521589201[100],Bacteria_Proteobacteria_Gammaproteobacteria_Xanthomonadales_Xanthomonadaceae_Arenimonas_donghaensis_DSM_18148_HO3_R19:0.06142639295854124):0.04853595307565817[100],Bacteria_Proteobacteria_Gammaproteobacteria_Xanthomonadales_Xanthomonadaceae_Silanimonas_lenta_DSM_16282:0.12913314306426882):0.027867128641709815[97]):0.042241828878411436[97],((Ga0172382_10004726:0.045973965968745034,Ga0172382_10040126:0.07424355360515333):0.07756793011652663[100],Ga0172382_10001373:0.12079824263408456):0.02232835420123669[60]):0.044052087183534816[97],(((((Bacteria_Proteobacteria_Gammaproteobacteria_Xanthomonadales_RIFOXYA1_FULL_Rhodanobacter_67_6:0.016621007905981422,'Bacteria_Proteobacteria_Gammaproteobacteria_Xanthomonadales_Xanthomonadaceae_Rhodanobacter_sp._2APBS1':0.012834152532607224):0.04913277934650395[100],'Bacteria_Proteobacteria_Gammaproteobacteria_Xanthomonadales_Xanthomonadaceae_Luteibacter_sp._9145':0.0937604340373186):0.014220271354904224[33],(Bacteria_Proteobacteria_Gammaproteobacteria_Xanthomonadales_Xanthomonadaceae_Dyella_japonica_A8:0.041328560903189704,'Bacteria_Proteobacteria_Gammaproteobacteria_Xanthomonadales_Xanthomonadaceae_Frateuria_terrea_CGMCC_1.7053':0.03822035317392425):0.012526576663463906[76]):0.01722340101128461[54],Bacteria_Proteobacteria_Gammaproteobacteria_Xanthomonadales_Xanthomonadaceae_Frateuria_aurantia_Kondo_67_DSM_6220:0.06086796498800684):0.12020620104288904[100],(Bacteria_Proteobacteria_Gammaproteobacteria_Xanthomonadales_Xanthomonadaceae_Rudaea_cellulosilytica_DSM_22992:0.15210738590965445,Ga0172380_10004285:0.13440406726193777):0.041714772913083564[94]):0.05152289765063056[100]):0.060543928946208414[100],Ga0172382_10046531:0.20676513350163583):0.1582924422740719[100],((((Bacteria_Proteobacteria_Gammaproteobacteria_Xanthomonadales_Xanthomonadaceae_Wohlfahrtiimonas_chitiniclastica_SH04:0.000001,Bacteria_Proteobacteria_Gammaproteobacteria_Xanthomonadales_Xanthomonadaceae_Wohlfahrtiimonas_chitiniclastica_DSM_18708:0.000001):0.1700772338559502[100],Bacteria_Proteobacteria_Gammaproteobacteria_Xanthomonadales_Xanthomonadaceae_Ignatzschineria_larvae_DSM_13226:0.20875208008579937):0.15428719036110916[100],(Bacteria_Proteobacteria_Gammaproteobacteria_Cardiobacteriales_Cardiobacteriaceae_Dichelobacter_nodosus_VCS1703A:0.18925754142740336,Bacteria_Proteobacteria_Gammaproteobacteria_Cardiobacteriales_Cardiobacteriaceae_Cardiobacterium_valvarum_F0432:0.1994980035711733):0.22270835755564145[100]):0.0408759660861997[76],((Bacteria_Proteobacteria_Gammaproteobacteria_Chromatiales_Halothiobacillaceae_Halothiobacillus_neapolitanus_c2:0.009596895597316912,Ga0172382_10022933:0.011192964759275448):0.14222903269752196[100],Ga0172382_10000391:0.18542771725415097):0.22451278713153222[100]):0.03328322616554136[47]):0.040260401250711286[52],(((('Bacteria_Proteobacteria_Gammaproteobacteria_Chromatiales_Ectothiorhodospiraceae_Ectothiorhodospira_sp._PHS_1':0.07912558548102044,Bacteria_Proteobacteria_Gammaproteobacteria_Chromatiales_Ectothiorhodospiraceae_Halorhodospira_halochloris_A:0.08127826026475438):0.058572900854893195[100],Bacteria_Proteobacteria_Gammaproteobacteria_Chromatiales_Ectothiorhodospiraceae_Thiorhodospira_sibirica_A12_ATCC_700588:0.1592431652034847):0.05377951008163118[99],Bacteria_Proteobacteria_Gammaproteobacteria_Chromatiales_Ectothiorhodospiraceae_Thioalkalivibrio_nitratireducens_DSM_14787:0.2871857553776529):0.058218367250666425[100],(Bacteria_Proteobacteria_Gammaproteobacteria_Thiotrichales_Thiotrichaceae_Leucothrix_mucor_DSM_2157:0.21039305557985655,Bacteria_Proteobacteria_Gammaproteobacteria_Thiotrichales_Thiotrichaceae_Thiothrix_flexilis_DSM_14609:0.20454810678122293):0.10462696530326054[100]):0.04578077918831136[91]):0.02401499999761647[38],(((((((Bacteria_Proteobacteria_Gammaproteobacteria_Xanthomonadales_Sinobacteraceae_Singularimonas_variicoloris_DSM_15731:0.024281617373212327,Bacteria_Proteobacteria_Gammaproteobacteria_unclassified_Gammaproteobacteria_Solimonas_soli_DSM_21787:0.029083095551871718):0.12859116502467538[100],Bacteria_Proteobacteria_Gammaproteobacteria_Xanthomonadales_Sinobacteraceae_Hydrocarboniphaga_effusa_AP103:0.15495804691924864):0.03620131480994315[87],(Ga0172379_10023571:0.07466386057830032,Bacteria_Proteobacteria_Gammaproteobacteria_Xanthomonadales_Sinobacteraceae_Nevskia_ramosa_DSM_11499:0.06462293895509985):0.13493918233136126[100]):0.04043103901203837[81],Bacteria_Proteobacteria_Gammaproteobacteria_Xanthomonadales_Sinobacteraceae_Polycyclovorans_algicola_TG408:0.22365186910300805):0.09703996792177971[100],Bacteria_Proteobacteria_Gammaproteobacteria_Xanthomonadales_Algiphilaceae_Algiphilus_aromaticivorans_DG1253:0.26125630072613815):0.1069500874983702[100],Bacteria_Proteobacteria_Gammaproteobacteria_Salinisphaerales_Salinisphaeraceae_Salinisphaera_shabanensis_E1L3A:0.33080693546329965):0.06343708049146635[97],(((Bacteria_Proteobacteria_Gammaproteobacteria_Chromatiales_Ectothiorhodospiraceae_Ectothiorhodospiraceae_bacterium_M19_40:0.18914581217624038,Bacteria_Proteobacteria_Gammaproteobacteria_Chromatiales_Ectothiorhodospiraceae_Arhodomonas_aquaeolei_DSM_8974:0.14099782968391272):0.03531566689554211[79],Bacteria_Proteobacteria_Gammaproteobacteria_Chromatiales_Ectothiorhodospiraceae_Nitrococcus_mobilis_Nb_231:0.20933595928780635):0.0535286967013171[100],Bacteria_Proteobacteria_Gammaproteobacteria_Chromatiales_Ectothiorhodospiraceae_Alkalilimnicola_ehrlichii_MLHE_1:0.17323396849904382):0.10496856842130597[100]):0.035558890007648[45]):0.02154744023478994[37],(((((Ga0172382_10022963:0.021896733871089147,Ga0172382_10022940:0.015029973355324966):0.030760651452632626[100],Ga0172382_10000313:0.11122777905516834):0.11446556166184285[100],(Ga0172382_10002871:0.05301991415433127,Ga0172382_10026660:0.04026033033065213):0.14120880995504415[100]):0.02887956636585365[74],Bacteria_Proteobacteria_Gammaproteobacteria_unclassified_Gammaproteobacteria_Thiohalomonas_denitrificans_HLD2:0.1528556099935967):0.04963652222926962[99],Bacteria_Proteobacteria_Gammaproteobacteria_Chromatiales_Ectothiorhodospiraceae_Thiohalospira_halophilus_HL3:0.2857812687637673):0.03975977912092521[54]):0.017492825621662433[27]):0.06317332048087332[97],(((((((((((((((((((((((Ga0172381_10011334:0.010806416415736209,Bacteria_Proteobacteria_Betaproteobacteria_Burkholderiales_Comamonadaceae_Rhodoferax_CG_Rhodof_02:0.018527213235262074):0.014929757241045838[97],Bacteria_Proteobacteria_Betaproteobacteria_Burkholderiales_Comamonadaceae_Rhodoferax_CG_Rhodof_01:0.025257340536335438):0.010457517061359223[56],(Bacteria_Proteobacteria_Betaproteobacteria_Burkholderiales_Comamonadaceae_Rhodoferax_CG_Rhodof_04:0.027857138892179556,Bacteria_Proteobacteria_Betaproteobacteria_Burkholderiales_Comamonadaceae_Rhodoferax_CG_Rhodof_03:0.019583746848472305):0.017435436252026815[97]):0.030695406880016485[99],Bacteria_Proteobacteria_Betaproteobacteria_Burkholderiales_Comamonadaceae_Rhodoferax_ferrireducens_T118:0.044127930977157614):0.024028503679777824[91],(Bacteria_Proteobacteria_Betaproteobacteria_Burkholderiales_Comamonadaceae_Hylemonella_gracilis_ATCC_19624:0.04843442368312001,'Bacteria_Proteobacteria_Betaproteobacteria_Burkholderiales_Comamonadaceae_Comamonadaceae_sp._CR':0.298811195127235):0.020169074857768976[26]):0.01209054799845255[25],Bacteria_Proteobacteria_Betaproteobacteria_Burkholderiales_Comamonadaceae_Polaromonas_naphthalenivorans_CJ2:0.10039742008961028):0.011258023349700608[33],(((Bacteria_Proteobacteria_Betaproteobacteria_Burkholderiales_Comamonadaceae_Xenophilus_azovorans_DSM_13620:0.021627338719876654,Bacteria_Proteobacteria_Betaproteobacteria_Burkholderiales_Comamonadaceae_Pseudacidovorax_intermedius_NH_1:0.027738488199623212):0.02831983850603681[100],Bacteria_Proteobacteria_Betaproteobacteria_Burkholderiales_Comamonadaceae_Variovorax_paradoxus_B4:0.04635508958100498):0.01899516299469095[98],Bacteria_Proteobacteria_Betaproteobacteria_Burkholderiales_Comamonadaceae_Ramlibacter_tataouinensis_TTB310:0.06090323503301276):0.011291785080309591[49]):0.008377323334849862[27],((((((Bacteria_Proteobacteria_Betaproteobacteria_Burkholderiales_Comamonadaceae_Delftia_acidovorans_SPH_1:0.02540334255146881,Bacteria_Proteobacteria_Betaproteobacteria_Burkholderiales_Comamonadaceae_Comamonas_testosteroni_TK102:0.04146371383860181):0.03377306467903729[100],Bacteria_Proteobacteria_Betaproteobacteria_Burkholderiales_Comamonadaceae_Alicycliphilus_denitrificans_K601:0.03300070759127127):0.013346885323945124[52],'Bacteria_Proteobacteria_Betaproteobacteria_Burkholderiales_Comamonadaceae_Acidovorax_avenae_subsp._citrulli_AAC00_1':0.028493517569339488):0.01920985122688723[97],Bacteria_Proteobacteria_Betaproteobacteria_Burkholderiales_Comamonadaceae_Verminephrobacter_eiseniae_EF01_2:0.07271467317858127):0.005819919421215403[49],(Bacteria_Proteobacteria_Betaproteobacteria_Burkholderiales_Comamonadaceae_Giesbergeria_anulus_ATCC_35958:0.03644227799815347,Bacteria_Proteobacteria_Betaproteobacteria_Burkholderiales_Comamonadaceae_Simplicispira_psychrophila_DSM_11588:0.04486507823709873):0.019371366344625773[100]):0.017884217307445827[97],Bacteria_Proteobacteria_Betaproteobacteria_Burkholderiales_Comamonadaceae_Curvibacter_lanceolatus_ATCC_14669:0.05051708331913085):0.006973497143853535[28]):0.016649660921217002[49],'Bacteria_Proteobacteria_Betaproteobacteria_Burkholderiales_Comamonadaceae_Limnohabitans_sp._Rim28_Limnohabitans_sp._Rim28_73_Contigs':0.058940182814188):0.013406551428388536[51],((Ga0172382_10007599:0.22771962222336617,Bacteria_Proteobacteria_Betaproteobacteria_Burkholderiales_Comamonadaceae_Brachymonas_chironomi_DSM_19884:0.10595672681994062):0.030483171599891[58],Bacteria_Proteobacteria_Betaproteobacteria_Burkholderiales_Comamonadaceae_Ottowia_thiooxydans_DSM_14619:0.07007315821117999):0.021928342823200886[68]):0.02185836390724827[61],(Ga0172377_10004159:0.03695657468571145,'Bacteria_Proteobacteria_Betaproteobacteria_Burkholderiales_Comamonadaceae_Hydrogenophaga_sp._T4':0.034325271342306785):0.06392719000906855[100]):0.06938934771184746[99],(((((('Bacteria_Proteobacteria_Betaproteobacteria_Burkholderiales_unclassified_Burkholderiales_Burkholderiales_Genera_incertae_sedis_Ideonella_sp._B508_1':0.06949229759564401,'Bacteria_Proteobacteria_Betaproteobacteria_Burkholderiales_unclassified_Burkholderiales_Burkholderiales_Genera_incertae_sedis_Mitsuaria_sp._H24L5A':0.07739803253050814):0.01729840215927725[63],Bacteria_Proteobacteria_Betaproteobacteria_Burkholderiales_unclassified_Burkholderiales_Burkholderiales_Genera_incertae_sedis_Rubrivivax_gelatinosus_IL144:0.07789845103955617):0.009107814414738957[34],Bacteria_Proteobacteria_Betaproteobacteria_Burkholderiales_Alcaligenaceae_Azohydromonas_australica_DSM_1124:0.05715899649882372):0.02411363175279968[98],(Bacteria_Proteobacteria_Betaproteobacteria_Burkholderiales_unclassified_Burkholderiales_Burkholderiales_Genera_incertae_sedis_Sphaerotilus_natans_natans_DSM_6575:0.04728061960981211,Bacteria_Proteobacteria_Betaproteobacteria_Burkholderiales_unclassified_Burkholderiales_Burkholderiales_Genera_incertae_sedis_Leptothrix_cholodnii_SP_6:0.05804495767428142):0.03736976013440696[100]):0.01779185279664519[95],'Bacteria_Proteobacteria_Betaproteobacteria_Burkholderiales_unclassified_Burkholderiales_Burkholderiales_Genera_incertae_sedis_Methylibium_sp._T29':0.06580135035277435):0.02002056838972255[100],Bacteria_Proteobacteria_Betaproteobacteria_Burkholderiales_Comamonadaceae_Caldimonas_manganoxidans_ATCC_BAA_369:0.06847915418385275):0.03304621421369003[100]):0.034096221420349515[95],Bacteria_Proteobacteria_Betaproteobacteria_Burkholderiales_unclassified_Burkholderiales_Burkholderiales_Genera_incertae_sedis_Thiomonas_intermedia_K12:0.17702635636405306):0.06788640050188377[100],(('Bacteria_Proteobacteria_Betaproteobacteria_Burkholderiales_Sutterellaceae_Parasutterella_Parasutterella_excrementihominis_YIT_11859_P_excrementihominisYIT11859_1.0_Cont110.1_whole_genome_shotgun':0.15285401111927222,Bacteria_Proteobacteria_Betaproteobacteria_Burkholderiales_Sutterellaceae_Sutterella_wadsworthensis_HGA0223:0.17080737232596244):0.12322775021054788[100],'Bacteria_Proteobacteria_Betaproteobacteria_Burkholderiales_Burkholderiaceae_Limnobacter_sp._MED105_unfinished_sequence':0.25938140936612797):0.03914583512487724[44]):0.015021192017697693[26],((((((((Bacteria_Proteobacteria_Betaproteobacteria_Burkholderiales_Alcaligenaceae_Taylorella_asinigenitalis_MCE3:0.09510543258859805,'Bacteria_Proteobacteria_Betaproteobacteria_Burkholderiales_Alcaligenaceae_Pelistega_sp._HM_7':0.04163622724303817):0.023780227968220213[96],(Bacteria_Proteobacteria_Betaproteobacteria_Burkholderiales_Alcaligenaceae_Brackiella_oedipodis_DSM_13743:0.11119883444314116,Bacteria_Proteobacteria_Betaproteobacteria_Burkholderiales_Alcaligenaceae_Oligella_ureolytica_DSM_18253:0.12637160880355625):0.037278304401156426[98]):0.010397789512174516[72],Bacteria_Proteobacteria_Betaproteobacteria_Burkholderiales_Alcaligenaceae_Basilea_psittacipulmonis_DSM_24701:0.11825617626274587):0.02116615945182554[91],Bacteria_Proteobacteria_Betaproteobacteria_Burkholderiales_Alcaligenaceae_Tetrathiobacter_kashmirensis_WT001:0.049030353947891125):0.04883434128584385[100],((((Ga0172382_10003703:0.015577064023755582,Ga0172382_10003771:0.011397716174160522):0.09662978124386834[100],Ga0172382_10001964:0.1055634615904455):0.018053768581935525[68],Bacteria_Proteobacteria_Betaproteobacteria_Burkholderiales_Alcaligenaceae_Alcaligenes_faecalis_phenolicus_DSM_16503:0.08618016621804214):0.021883511792819554[94],'Bacteria_Proteobacteria_Betaproteobacteria_Burkholderiales_Alcaligenaceae_Pusillimonas_sp._T7_7':0.044451286114339794):0.02575749170342556[99]):0.01006371860267663[44],((Bacteria_Proteobacteria_Betaproteobacteria_Burkholderiales_Alcaligenaceae_Achromobacter_xylosoxidans_NBRC_15126_ATCC_27061:0.030080732781783137,Bacteria_Proteobacteria_Betaproteobacteria_Burkholderiales_Alcaligenaceae_Bordetella_bronchiseptica_RB50:0.02465879081587241):0.015240826101888594[90],Bacteria_Proteobacteria_Betaproteobacteria_unclassified_Betaproteobacteria_Candidatus_Kinetoplastibacterium_oncopeltii_TCC290E:0.2518430814299779):0.018716566362118048[92]):0.029956457950742532[87],(Ga0172382_10002302:0.07774576422033919,Ga0172382_10029646:0.10213129820205546):0.032085683642377294[71]):0.08443818729641484[100],(((Ga0172382_10007778:0.08519030154981078,Ga0172382_10023359:0.13691937553919953):0.05947338161530924[100],Ga0172382_10001437:0.1710274881515077):0.0527264669751375[100],Bacteria_Proteobacteria_Betaproteobacteria_Burkholderiales_Burkholderiaceae_Lautropia_mirabilis_ATCC_51599_contig00020:0.14618946275946154):0.05433712168774907[100]):0.027513091432823877[67]):0.028083680116449017[56],(((((((Bacteria_Proteobacteria_Betaproteobacteria_Burkholderiales_Oxalobacteraceae_Duganella_zoogloeoides_ATCC_25935:0.000001,Bacteria_Proteobacteria_Betaproteobacteria_Rhodocyclales_Rhodocyclaceae_Zoogloea_ramigera_ATCC_19544:0.000001):0.040703704195937274[100],Bacteria_Proteobacteria_Betaproteobacteria_Burkholderiales_Oxalobacteraceae_Massilia_alkalitolerans_DSM_17462:0.053092236831397965):0.05835286121592276[100],(('Bacteria_Proteobacteria_Betaproteobacteria_Burkholderiales_Oxalobacteraceae_Janthinobacterium_sp._Marseille':0.005420619083071898,Bacteria_Proteobacteria_Betaproteobacteria_Burkholderiales_Oxalobacteraceae_Herminiimonas_arsenicoxydans:0.027261377595614977):0.02385605101135546[99],Bacteria_Proteobacteria_Betaproteobacteria_Burkholderiales_Oxalobacteraceae_Collimonas_fungivorans_Ter331:0.03436507335332406):0.013930077244715289[92]):0.017960353088743553[60],Bacteria_Proteobacteria_Betaproteobacteria_Burkholderiales_Oxalobacteraceae_Herbaspirillum_seropedicae_SmR1:0.055457924012566995):0.022834792228189293[67],Bacteria_Proteobacteria_Betaproteobacteria_unclassified_Betaproteobacteria_Candidatus_Profftella_armatura_syncytium_symbiont_of_Diaphorina_citri:0.3857277983045151):0.019418735186755853[58],'Bacteria_Proteobacteria_Betaproteobacteria_Burkholderiales_Oxalobacteraceae_Oxalobacter_formigenes_OXCC13_genomic_scaffold_supercont1.1':0.1428711391369326):0.07202623238935013[100],((((Bacteria_Proteobacteria_Betaproteobacteria_Burkholderiales_Burkholderiaceae_Ralstonia_solanacearum_GMI1000:0.043058067014690504,Bacteria_Proteobacteria_Betaproteobacteria_Burkholderiales_Burkholderiaceae_Ralstonia_eutropha_JMP134:0.05347656655004496):0.0412472468295042[100],Bacteria_Proteobacteria_Betaproteobacteria_Burkholderiales_Burkholderiaceae_Polynucleobacter_necessarius_asymbioticus_MWH_MoK4_closed_genome:0.18418039836391298):0.04076317882355607[100],((Bacteria_Proteobacteria_Betaproteobacteria_Burkholderiales_Burkholderiaceae_Burkholderia_cenocepacia_AU_1054:0.044735053470136066,'Bacteria_Proteobacteria_Betaproteobacteria_Burkholderiales_Burkholderiaceae_Glomeribacter_sp._1016415':0.161540262685822):0.033350422958517534[99],Bacteria_Proteobacteria_Betaproteobacteria_Burkholderiales_Burkholderiaceae_Pandoraea_pnomenusa_RB38:0.08012773883651603):0.026875974972459638[79]):0.05831387822106926[100],Bacteria_Proteobacteria_Betaproteobacteria_Burkholderiales_Alcaligenaceae_Derxia_gummosa_DSM_723:0.16036037208443243):0.01974542690070269[17]):0.021787912496950668[27]):0.06917931495105378[94],((((((((Ga0172380_10013650:0.009972246515025152,Ga0172380_10032049:0.022244373532135153):0.05188574089693487[100],Ga0172380_10005901:0.07810898384508258):0.0015506693456885934[63],Ga0172380_10041160:0.03436352631290651):0.02580488029602357[99],((Ga0172380_10027279:0.03788723471592936,Ga0172380_10000480:0.03239908222435384):0.016475124613951397[100],Ga0172379_10012033:0.026202461454069237):0.043482862039696535[100]):0.058555139409229184[100],((((Ga0172379_10024059:0.01459855677865507,Ga0172379_10016308:0.018729923817528693):0.007945109630977498[66],Ga0172380_10012501:0.009014616948786358):0.07059010536760235[100],Ga0172380_10000324:0.08619012062655518):0.027138335847952444[99],Bacteria_Proteobacteria_Betaproteobacteria_Rhodocyclales_GWA2_Rhodocyclales_65_20:0.07705689031386598):0.025742488509264305[99]):0.025656329415546963[90],(Bacteria_Proteobacteria_Betaproteobacteria_Rhodocyclales_Rhodocyclaceae_CG_Rhodocyc_01:0.20382901723565627,Ga0172380_10010709:0.0886819416297433):0.0254642795803397[24]):0.03211244140023828[39],((((('Bacteria_Proteobacteria_Betaproteobacteria_Rhodocyclales_Rhodocyclaceae_Azoarcus_sp._KH32C':0.05669283677206938,Bacteria_Proteobacteria_Betaproteobacteria_Rhodocyclales_Rhodocyclaceae_Aromatoleum_aromaticum_EbN1:0.04373749406003258):0.025782187668908385[100],Ga0172382_10001332:0.08103878854180646):0.028468262611236916[100],(Ga0172382_10003620:0.0638761450252745,'Bacteria_Proteobacteria_Betaproteobacteria_Rhodocyclales_Rhodocyclaceae_Thauera_sp._MZ1T':0.05562957125235668):0.024930608079251293[99]):0.05355477124762231[100],Bacteria_Proteobacteria_Betaproteobacteria_Rhodocyclales_Rhodocyclaceae_Uliginosibacterium_gangwonense_DSM_18521:0.14267009130027875):0.018276507079285853[54],((Ga0172378_10036731:0.01006534137837356,Bacteria_Proteobacteria_Betaproteobacteria_Hydrogenophilales_Hydrogenophilaceae_Tepidiphilus_margaritifer_DSM_15129:0.003985537018658114):0.26711179482742864[100],Bacteria_Proteobacteria_Betaproteobacteria_Rhodocyclales_Rhodocyclaceae_Methyloversatilis_universalis_EHg5:0.12658446990658412):0.03441309375051338[26]):0.02337609377244121[40]):0.01865751314451769[97],(((('Bacteria_Proteobacteria_Betaproteobacteria_unclassified_Betaproteobacteria_Candidatus_Accumulibacter_phosphatis_clade_IIA_str._UW_1':0.12550644656932786,Bacteria_Proteobacteria_Betaproteobacteria_Rhodocyclales_Rhodocyclaceae_Propionivibrio_dicarboxylicus_DSM_5885:0.050997107640753825):0.04165461688948291[100],Ga0172380_10002176:0.101767256093658):0.02606562301329518[90],Bacteria_Proteobacteria_Betaproteobacteria_Rhodocyclales_Rhodocyclaceae_Dechlorosoma_suillum_PS:0.08463247792385031):0.02398731188192693[89],(((Bacteria_Proteobacteria_Betaproteobacteria_Rhodocyclales_Rhodocyclaceae_Dechloromonas_aromatica_RCB:0.020297393117351348,Bacteria_Proteobacteria_Betaproteobacteria_Rhodocyclales_Rhodocyclaceae_Dechlorobacter_hydrogenophilus_LT_1:0.04867717718593312):0.013240583145742502[97],Bacteria_Proteobacteria_Betaproteobacteria_Rhodocyclales_Rhodocyclaceae_Azonexus_hydrophilus_DSM_23864:0.04317913732436507):0.05985853788782247[100],Bacteria_Proteobacteria_Betaproteobacteria_Rhodocyclales_Rhodocyclaceae_Azovibrio_restrictus_DSM_23866:0.08899113834835859):0.03605833047292206[100]):0.052206845942468494[100]):0.0374910446273522[98]):0.03015552809586275[89],((Bacteria_Proteobacteria_Betaproteobacteria_uncultured_SG8_41:0.20901877114269363,Bacteria_Proteobacteria_Betaproteobacteria_uncultured_SG8_39:0.209949915079215):0.03740210269672506[94],Bacteria_Proteobacteria_Betaproteobacteria_uncultured_SG8_40:0.22521376682750383):0.04595653088750362[96]):0.027665606391237496[65],((((((Bacteria_Proteobacteria_Betaproteobacteria_Gallionellales_Gallionellaceae_Gallionella_capsiferriformans_ES_2:0.08757244359444272,Ga0172380_10016332:0.03515581442806903):0.015188972339988283[60],Ga0172379_10004001:0.03768647543735604):0.009085147930454784[43],Ga0172379_10029113:0.05736429798794296):0.05015971311409473[100],'Bacteria_Proteobacteria_Betaproteobacteria_Gallionellales_Gallionellaceae_Ferriphaselus_sp._R_1':0.06458635312603311):0.028213634605665838[85],((Ga0172379_10001330:0.024147916273638437,Ga0172380_10029645:0.051987397681239944):0.023012665670949062[99],Bacteria_Proteobacteria_Betaproteobacteria_Gallionellales_Gallionellaceae_Sideroxydans_lithotrophicus_ES_1:0.03806621980778946):0.0607619441379903[100]):0.09577380366685784[100],(Bacteria_Proteobacteria_Betaproteobacteria_Nitrosomonadales_Nitrosomonadaceae_Nitrosomonas_europaea_ATCC_19718:0.3936253580396145,Bacteria_Proteobacteria_Betaproteobacteria_Nitrosomonadales_Nitrosomonadaceae_Nitrosospira_multiformis_ATCC_25196:0.11544423971767648):0.06999241244017762[91]):0.0244438010128869[44]):0.013636593396407548[24],((Bacteria_Proteobacteria_Betaproteobacteria_Hydrogenophilales_Hydrogenophilaceae_CG_Hydro_01:0.07578250914983631,Ga0172380_10007595:0.05588793376522716):0.1297909693717898[100],(Bacteria_Proteobacteria_Betaproteobacteria_Hydrogenophilales_GWE1_Thiobacillus_62_9:0.039949525211915304,Bacteria_Proteobacteria_Betaproteobacteria_Hydrogenophilales_Hydrogenophilaceae_Thiobacillus_denitrificans_ATCC_25259:0.026964345928705225):0.14404742750140986[100]):0.05250455838739265[100]):0.0165786458776358[12],((Ga0172380_10016390:0.09823726088678875,Bacteria_Proteobacteria_Betaproteobacteria_Hydrogenophilales_Hydrogenophilaceae_Sulfuricella_denitrificans_skB26:0.08548272661058665):0.04174756841624783[100],Ga0172380_10001553:0.12357841654698509):0.03997975534178133[87]):0.024775446020862457[36],((((((((((Bacteria_Proteobacteria_Betaproteobacteria_Neisseriales_Neisseriaceae_Kingella_kingae_PYKK081:0.011314276911043475,Bacteria_Proteobacteria_Betaproteobacteria_Neisseriales_Neisseriaceae_Simonsiella_muelleri_ATCC_29453:0.014912268003121731):0.011392521787476184[100],Bacteria_Proteobacteria_Betaproteobacteria_Neisseriales_Neisseriaceae_Alysiella_crassa_DSM_2578:0.01827359981840626):0.01805482801855529[100],Bacteria_Proteobacteria_Betaproteobacteria_Neisseriales_Neisseriaceae_Conchiformibius_steedae_DSM_2580:0.03964688876320377):0.024315105232998402[100],(Bacteria_Proteobacteria_Betaproteobacteria_Neisseriales_Neisseriaceae_Eikenella_corrodens_ATCC_23834:0.11789459357110132,Bacteria_Proteobacteria_Betaproteobacteria_Neisseriales_Neisseriaceae_Neisseria_gonorrhoeae_FA_1090:0.06521443538869987):0.016600729916679047[87]):0.024729532944035792[99],(Bacteria_Proteobacteria_Betaproteobacteria_Neisseriales_Neisseriaceae_Stenoxybacter_acetivorans_DSM_19021:0.06444159702940366,Bacteria_Proteobacteria_Betaproteobacteria_Neisseriales_Neisseriaceae_Snodgrassella_alvi_wkB2:0.06029570631704706):0.0440326258096273[100]):0.03438132599524968[100],Bacteria_Proteobacteria_Betaproteobacteria_Neisseriales_Neisseriaceae_Chromobacterium_group_Vitreoscilla_stercoraria_DSM_513:0.11041817017166267):0.10798604782869958[100],((Bacteria_Proteobacteria_Betaproteobacteria_Neisseriales_Chromobacteriaceae_Lutiella_nitroferrum_2002_unfinished_sequence:0.042386653760823734,Bacteria_Proteobacteria_Betaproteobacteria_Neisseriales_Neisseriaceae_Paludibacterium_yongneupense_DSM_18731:0.06132306318102154):0.020309709741960447[77],Bacteria_Proteobacteria_Betaproteobacteria_Neisseriales_Neisseriaceae_Chromobacterium_group_Chromobacterium_violaceum_ATCC_12472:0.05848826427676279):0.03875694264253715[100]):0.04296458774016987[84],((Bacteria_Proteobacteria_Betaproteobacteria_Neisseriales_Neisseriaceae_Microvirgula_aerodenitrificans_DSM_15089:0.06445024608339667,Bacteria_Proteobacteria_Betaproteobacteria_Neisseriales_Neisseriaceae_Laribacter_hongkongensis_HLHK9:0.047929679279282045):0.04469461665294494[100],Ga0172380_10030831:0.35725223596629974):0.0234087388185098[30]):0.047680644328548905[51],((Bacteria_Proteobacteria_Betaproteobacteria_Neisseriales_Neisseriaceae_Deefgea_rivuli_DSM_18356:0.1310518597202961,Bacteria_Proteobacteria_Betaproteobacteria_Neisseriales_Neisseriaceae_Formivibrio_citricus_DSM_6150:0.07514157300330826):0.05960244347035237[86],Bacteria_Proteobacteria_Betaproteobacteria_Burkholderiales_Burkholderiaceae_Chitinimonas_koreensis_DSM_17726:0.10450784917361666):0.02862784283640929[72]):0.02686932561772215[72],Bacteria_Proteobacteria_Betaproteobacteria_Neisseriales_Neisseriaceae_Leeia_oryzae_DSM_17879:0.1787252515179918):0.05354320440424232[88]):0.036998664334299214[63],(((('Bacteria_Proteobacteria_Betaproteobacteria_Methylophilales_Methylophilaceae_Methylotenera_sp._301':0.02756288062718193,Ga0172380_10004297:0.023840877236092073):0.03437269755728112[100],Ga0172379_10032470:0.018847346956750943):0.04357608573066374[100],Bacteria_Proteobacteria_Betaproteobacteria_Methylophilales_Methylophilaceae_Methylophilus_methylotrophus_ATCC_53528:0.09684985498493903):0.041818996266488107[100],('Bacteria_Proteobacteria_Betaproteobacteria_Methylophilales_Methylophilaceae_Methylovorus_sp._SIP3_4':0.04503334391081504,Bacteria_Proteobacteria_Betaproteobacteria_Methylophilales_Methylophilaceae_Methylobacillus_flagellatus_KT:0.06830777099770557):0.02514277870116821[95]):0.11495677633275259[100]):0.17054552431030245[100]):0.06864710832137044[100],((((Ga0172379_10001800:0.02170051247382121,Bacteria_Proteobacteria_Gammaproteobacteria_Acidithiobacillales_Acidithiobacillaceae_Acidithiobacillus_ferrivorans_SS3:0.016211739566959427):0.015150805233677733[97],(Ga0172381_10000704:0.000001,Ga0172382_10011608:0.000455):0.02718494673406857[100]):0.03631717624405262[100],(Ga0172382_10001277:0.04508785509394997,Ga0172382_10001542:0.05016525147050599):0.019338306701111918[88]):0.28314199217937164[100],Bacteria_Proteobacteria_Gammaproteobacteria_Acidithiobacillales_Thermithiobacillaceae_Thermithiobacillus_tepidarius_DSM_3134:0.1608424757600857):0.13589965354326594[100]):0.11820916228189926[100],Bacteria_Proteobacteria_Gammaproteobacteria_unclassified_Gammaproteobacteria_Thiohalorhabdus_denitrificans_HL19:0.4171510300738914):0.15125193886972843[100],(((((((Bacteria_Proteobacteria_Zetaproteobacteria_zeta_proteobacterium_SCGC_AB_137_L23:0.000001,Bacteria_Proteobacteria_Zetaproteobacteria_zeta_proteobacterium_SCGC_AB_137_C09:0.000001):0.21807921114227602[100],(Bacteria_Proteobacteria_Zetaproteobacteria_zeta_proteobacterium_SCGC_AB_133_C04:0.032143816773072764,Bacteria_Proteobacteria_Zetaproteobacteria_zeta_proteobacterium_SCGC_AB_137_I08:0.028878923531311607):0.08804076076763856[100]):0.03617146350505518[93],Bacteria_Proteobacteria_Zetaproteobacteria_Zetaproteobacteria_bacterium_TAG_1:0.18551353450332053):0.03387690759598394[54],(((Bacteria_Proteobacteria_Zetaproteobacteria_zeta_proteobacterium_SCGC_AB_137_J06:0.08331506400721711,Bacteria_Proteobacteria_Zetaproteobacteria_zeta_proteobacterium_SCGC_AB_133_G06:0.058002330319837814):0.03386817528013397[99],Bacteria_Proteobacteria_Zetaproteobacteria_Mariprofundales_Mariprofundaceae_Mariprofundus_ferrooxydans_M34:0.09574648126717111):0.024355819246399513[74],'Bacteria_Proteobacteria_Zetaproteobacteria_Mariprofundales_Mariprofundaceae_Mariprofundus_sp._EKF_M39':0.09252813919560676):0.07515721239685202[100]):0.07495535458267977[100],Bacteria_Proteobacteria_Zetaproteobacteria_zeta_proteobacterium_SCGC_AC_673_C02:0.1621977510153947):0.047368011321718306[87],Bacteria_Proteobacteria_Zetaproteobacteria_zeta_proteobacterium_SCGC_AC_673_M07:0.2550104001931044):0.33992622537030215[100],Bacteria_Proteobacteria_CG_Proteo_01:0.4364790567524621):0.07366152372496337[72]):0.04658431313383282[44],(((((((((((((((((((((((((Bacteria_Proteobacteria_Alphaproteobacteria_Rhizobiales_Phyllobacteriaceae_Mesorhizobium_australicum_WSM2073:0.053149651154335675,Bacteria_Proteobacteria_Alphaproteobacteria_Rhizobiales_Phyllobacteriaceae_Aquamicrobium_defluvii_W13Z1:0.05881607486381135):0.03043406175299168[100],Bacteria_Proteobacteria_Alphaproteobacteria_Rhizobiales_Phyllobacteriaceae_Pseudaminobacter_salicylatoxidans_KCT001:0.04344666812477982):0.02264309805945297[97],('Bacteria_Proteobacteria_Alphaproteobacteria_Rhizobiales_Phyllobacteriaceae_Aliihoeflea_sp._2WW':0.0855297863345732,'Bacteria_Proteobacteria_Alphaproteobacteria_Rhizobiales_Phyllobacteriaceae_Aminobacter_sp._J41':0.07245071337421916):0.020153901798678486[66]):0.02243728952311619[79],(Bacteria_Proteobacteria_Alphaproteobacteria_Rhizobiales_Phyllobacteriaceae_Nitratireductor_aquibiodomus_JCM_21793:0.06720853534429283,'Bacteria_Proteobacteria_Alphaproteobacteria_Rhizobiales_Phyllobacteriaceae_Chelativorans_sp._BNC1':0.07338470474302516):0.040389038077242034[100]):0.034995265031002365[100],((((Bacteria_Proteobacteria_Alphaproteobacteria_Rhizobiales_Brucellaceae_Ochrobactrum_anthropi_ATCC_49188_1:0.013970722407476988,Bacteria_Proteobacteria_Alphaproteobacteria_Rhizobiales_Brucellaceae_Brucella_abortus_A13334:0.01359668211824383):0.03841493117494332[100],'Bacteria_Proteobacteria_Alphaproteobacteria_Rhizobiales_Brucellaceae_Pseudochrobactrum_sp._AO18b':0.06795072023641824):0.020058439600948663[89],'Bacteria_Proteobacteria_Alphaproteobacteria_Rhizobiales_Phyllobacteriaceae_Phyllobacterium_sp._YR531':0.07448661246335098):0.016316798864202564[62],Bacteria_Proteobacteria_Alphaproteobacteria_Rhizobiales_Bartonellaceae_Bartonella_clarridgeiae_73:0.16966663604832588):0.031107969309380312[100]):0.022473703981682025[92],((((((('Bacteria_Proteobacteria_Alphaproteobacteria_Rhizobiales_Rhizobiaceae_RhizobiumAgrobacterium_group_Agrobacterium_sp._H13_3':0.04123831562438962,Ga0172380_10003979:0.040229141408469005):0.011922446391106956[59],'Bacteria_Proteobacteria_Alphaproteobacteria_Rhizobiales_Rhizobiaceae_Neorhizobium_galegae_bv._orientalis_HAMBI_540':0.04426057013521989):0.014978358415911774[86],'Bacteria_Proteobacteria_Alphaproteobacteria_Rhizobiales_Rhizobiaceae_RhizobiumAgrobacterium_group_Rhizobium_leguminosarum_bv._trifolii_CB782':0.05373051665245754):0.01258700796031409[72],((Bacteria_Proteobacteria_Alphaproteobacteria_Rhizobiales_Rhizobiaceae_SinorhizobiumEnsifer_group_Ensifer_meliloti_GR4:0.02330145314638976,Bacteria_Proteobacteria_Alphaproteobacteria_Rhizobiales_Rhizobiaceae_SinorhizobiumEnsifer_group_Ensifer_adhaerens_OV14:0.017782390411224203):0.021370209850162247[100],Bacteria_Proteobacteria_Alphaproteobacteria_Rhizobiales_Rhizobiaceae_Shinella_zoogleoides_DD12_SHLA:0.048230888951672934):0.012287814811268394[50]):0.029103032864107092[99],Bacteria_Proteobacteria_Alphaproteobacteria_Rhizobiales_Phyllobacteriaceae_Hoeflea_phototrophica_DFL_43:0.09957597976891153):0.024591704964238126[53],(Bacteria_Proteobacteria_Alphaproteobacteria_Rhizobiales_Rhizobiaceae_Candidatus_Liberibacter_americanus_Sao_Paulo:0.5164103493025336,Bacteria_Proteobacteria_Alphaproteobacteria_Rhizobiales_Aurantimonadaceae_Martelella_mediterranea_DSM_17316:0.09660507743535574):0.012054951021121418[28]):0.03631890451095465[98],'Bacteria_Proteobacteria_Alphaproteobacteria_Rhodobacterales_Rhodobacteraceae_Ahrensia_sp._13_GOM_1096m':0.18764775418966106):0.029530507548451546[81]):0.02325320175140222[88],((Bacteria_Proteobacteria_Alphaproteobacteria_Rhizobiales_Aurantimonadaceae_Aurantimonas_manganoxydans_SI85_9A1:0.06498444399414671,Bacteria_Proteobacteria_Alphaproteobacteria_Rhizobiales_Aurantimonadaceae_Fulvimarina_pelagi_HTCC2506_unfinished_sequence:0.11251685754563034):0.03094184296505631[97],Bacteria_Proteobacteria_Alphaproteobacteria_Rhizobiales_Aurantimonadaceae_Aurantimonas_ureilytica_DSM_18598:0.09324436831372829):0.05632162373022753[100]):0.0833103703964273[100],((((((((Bacteria_Proteobacteria_Alphaproteobacteria_Rhizobiales_Beijerinckiaceae_Methylocapsa_acidiphila_B2:0.04558615452438808,Bacteria_Proteobacteria_Alphaproteobacteria_Rhizobiales_Beijerinckiaceae_Methylocella_silvestris_BL2:0.0645851548088272):0.02806870786783877[100],'Bacteria_Proteobacteria_Alphaproteobacteria_Rhizobiales_Beijerinckiaceae_Beijerinckia_indica_subsp._indica_ATCC_9039':0.07652887983015866):0.022527551854535144[67],Bacteria_Proteobacteria_Alphaproteobacteria_Rhizobiales_Beijerinckiaceae_Methyloferula_stellata_AR4:0.07213236799058187):0.034519996115618845[100],('Bacteria_Proteobacteria_Alphaproteobacteria_Rhizobiales_Methylocystaceae_Methylocystis_sp._SC2':0.07656535811147691,'Bacteria_Proteobacteria_Alphaproteobacteria_Rhizobiales_Methylocystaceae_Methylosinus_sp._PW1':0.08279930414503056):0.06708596301562197[100]):0.07370511406474067[100],((((Bacteria_Proteobacteria_Alphaproteobacteria_Rhizobiales_Bradyrhizobiaceae_Salinarimonas_rosea_DSM_21201:0.1338734822885228,Bacteria_Proteobacteria_Alphaproteobacteria_Rhizobiales_Methylobacteriaceae_Methylobacterium_extorquens_PA1:0.13178358803903745):0.03381885210862112[67],'Bacteria_Proteobacteria_Alphaproteobacteria_Rhizobiales_Methylobacteriaceae_Microvirga_sp._WSM3557':0.07084794526119076):0.0323729738286862[95],'Bacteria_Proteobacteria_Alphaproteobacteria_Rhizobiales_Bradyrhizobiaceae_Bosea_sp._UNC402CLCol':0.1297158202492681):0.03404116788838962[86],'Bacteria_Proteobacteria_Alphaproteobacteria_Rhizobiales_Beijerinckiaceae_Chelatococcus_sp._GW1':0.08726079889882099):0.0346548944460463[99]):0.03462335197867539[99],(((((Bacteria_Proteobacteria_Alphaproteobacteria_Rhizobiales_Bradyrhizobiaceae_Rhodopseudomonas_palustris_BisB18:0.049292320989806626,Bacteria_Proteobacteria_Alphaproteobacteria_Rhizobiales_Bradyrhizobiaceae_Bradyrhizobium_japonicum_SEMIA_5079:0.0658883179920644):0.015137430741121616[88],Bacteria_Proteobacteria_Alphaproteobacteria_Rhizobiales_Bradyrhizobiaceae_Nitrobacter_hamburgensis_X14:0.05304412265142444):0.020853210316049697[100],(Bacteria_Proteobacteria_Alphaproteobacteria_Rhizobiales_Bradyrhizobiaceae_Oligotropha_carboxidovorans_OM5:0.05523181043000447,'Bacteria_Proteobacteria_Alphaproteobacteria_Rhizobiales_Bradyrhizobiaceae_Afipia_sp._OHSU_I_C6':0.04620067215867918):0.019105143166885608[83]):0.10541379875467927[100],'Bacteria_Proteobacteria_Alphaproteobacteria_Rhodobacterales_Rhodobacteraceae_Rhodovulum_sp._PH10':0.14749697810236162):0.06929100402113786[100],((Bacteria_Proteobacteria_Alphaproteobacteria_Rhizobiales_Xanthobacteraceae_Azorhizobium_caulinodans_ORS_571:0.05604630219980189,Bacteria_Proteobacteria_Alphaproteobacteria_Rhizobiales_Xanthobacteraceae_Xanthobacter_autotrophicus_Py2:0.0718491233751477):0.040582706924470635[100],(Bacteria_Proteobacteria_Alphaproteobacteria_Rhizobiales_Xanthobacteraceae_Starkeya_novella_DSM_506:0.02891897436752844,'Bacteria_Proteobacteria_Alphaproteobacteria_Rhizobiales_Xanthobacteraceae_Ancylobacter_sp._FA202':0.04132556655301034):0.07540925340238047[100]):0.0647341822547487[100]):0.02393955027742578[67]):0.025912852763140926[69],'Bacteria_Proteobacteria_Alphaproteobacteria_Rhizobiales_Methylocystaceae_Methylopila_sp._M107':0.19571296559024898):0.04123008662112193[99],(Bacteria_Proteobacteria_Alphaproteobacteria_Rhizobiales_Methylocystaceae_Pleomorphomonas_oryzae_DSM_16300:0.16967130798713104,Bacteria_Proteobacteria_Alphaproteobacteria_Rhizobiales_Hyphomicrobiaceae_Prosthecomicrobium_hirschii_ATCC_27832:0.16917368673266386):0.034653177191933615[49]):0.021229118748166353[40]):0.021005100101460616[31],((((Bacteria_Proteobacteria_Alphaproteobacteria_Rhodobacterales_Rhodobacteraceae_Labrenzia_alexandrii_DFL_11:0.05724068231026669,'Bacteria_Proteobacteria_Alphaproteobacteria_Rhodobacterales_Rhodobacteraceae_Roseibium_sp._TrichSKD4_genomic_scaffold_scf_1119120597021':0.04070777461450836):0.033101978621064454[100],Bacteria_Proteobacteria_Alphaproteobacteria_Rhodobacterales_Rhodobacteraceae_Pannonibacter_phragmitetus_DSM_14782:0.054419359106239185):0.03334827689181319[99],(Bacteria_Proteobacteria_Alphaproteobacteria_Rhodobacterales_Rhodobacteraceae_Nesiotobacter_exalbescens_DSM_16456:0.04926767536557941,'Bacteria_Proteobacteria_Alphaproteobacteria_Rhodobacterales_Rhodobacteraceae_Pseudovibrio_sp._FO_BEG1':0.05468909654152254):0.06023157611160679[100]):0.025757313063683007[60],Bacteria_Proteobacteria_Alphaproteobacteria_Rhodobacterales_Rhodobacteraceae_Stappia_stellulata_DSM_5886:0.11209003668185691):0.0783493494062455[100]):0.01450023331820205[8],((Bacteria_Proteobacteria_Alphaproteobacteria_Rhizobiales_unclassified_Rhizobiales_Bauldia_litoralis_ATCC_35022:0.18291135753535004,Bacteria_Proteobacteria_Alphaproteobacteria_Rhizobiales_Rhizobiaceae_Kaistia_adipata_DSM_17808:0.1314889113315818):0.04810230663609083[96],(Bacteria_Proteobacteria_Alphaproteobacteria_Rhizobiales_Rhodobiaceae_Lutibaculum_baratangense_AMV1:0.17714331261904137,Bacteria_Proteobacteria_Alphaproteobacteria_Rhizobiales_unclassified_Rhizobiales_Amorphus_coralli_DSM_19760:0.17625308180892318):0.0438576443283627[56]):0.02207158421147426[22]):0.0210010566081964[54],(((((Bacteria_Proteobacteria_Alphaproteobacteria_Rhizobiales_unclassified_Rhizobiales_Vasilyevaea_enhydra_ATCC_23634:0.07455419275149344,Ga0172380_10033584:0.05361192864463238):0.033013571292481725[98],'Bacteria_Proteobacteria_Alphaproteobacteria_Rhizobiales_Hyphomicrobiaceae_Devosia_sp._17_2_E_8':0.09555285126675361):0.09338008006569698[100],Bacteria_Proteobacteria_Alphaproteobacteria_Rhizobiales_Hyphomicrobiaceae_Pelagibacterium_halotolerans_B2:0.10686676004290163):0.03948780904484828[92],(Bacteria_Proteobacteria_Alphaproteobacteria_Rhizobiales_Hyphomicrobiaceae_Maritalea_myrionectae_DSM_19524:0.11499006350493035,Bacteria_Proteobacteria_Alphaproteobacteria_Rhizobiales_Hyphomicrobiaceae_Cucumibacter_marinus_DSM_18995:0.09357290967823628):0.03921652128856268[100]):0.11109689129120914[100],Bacteria_Proteobacteria_Alphaproteobacteria_Rhizobiales_Rhodobiaceae_Afifella_pfennigii_DSM_17143:0.182387745034835):0.032889916600695734[78]):0.0342086868825362[85],((Ga0172382_10012200:0.05244780798259008,Bacteria_Proteobacteria_Alphaproteobacteria_Rhizobiales_Phyllobacteriaceae_Parvibaculum_lavamentivorans_DS_1:0.06190394170895619):0.1288974017590223[100],Bacteria_Proteobacteria_Alphaproteobacteria_unclassified_Alphaproteobacteriar_Canditatus_Phaeomarinobacter_ectocarpi_Ec32:0.19090560925296396):0.04435167272954921[99]):0.020717994749125168[44],((Bacteria_Proteobacteria_Alphaproteobacteria_Rhizobiales_Hyphomicrobiaceae_Hyphomicrobium_nitrativorans_NL23:0.12673270912749057,Ga0172380_10000385:0.14313519854756507):0.15022571687710906[100],Bacteria_Proteobacteria_Alphaproteobacteria_Rhizobiales_Hyphomicrobiaceae_Rhodomicrobium_vannielii_ATCC_17100:0.19692247292220966):0.06544517600664967[99]):0.02760820516981166[75],'Bacteria_Proteobacteria_Alphaproteobacteria_unclassified_Alphaproteobacteria_Thermopetrobacter_sp._TC1':0.2780946331203684):0.02784379683848881[65],(((((((((((((('Bacteria_Proteobacteria_Alphaproteobacteria_Rhodobacterales_Rhodobacteraceae_Sulfitobacter_sp._NAS_14.1':0.035230972751198575,Bacteria_Proteobacteria_Alphaproteobacteria_Rhodobacterales_Rhodobacteraceae_Roseobacter_litoralis_Och_149:0.056634605109561864):0.008160286116800197[35],Bacteria_Proteobacteria_Alphaproteobacteria_Rhodobacterales_Rhodobacteraceae_Oceanibulbus_indolifex_HEL_45_unfinished_sequence:0.0431113224241817):0.024271134718486742[98],Bacteria_Proteobacteria_Alphaproteobacteria_Rhodobacterales_Rhodobacteraceae_Sedimentitalea_cNanh_4411:0.04293840661345705):0.025242399146753858[99],Bacteria_Proteobacteria_Alphaproteobacteria_Rhodobacterales_Rhodobacteraceae_Nereida_ignava_DSM_16309:0.08603484770710956):0.007771513744076142[22],Bacteria_Proteobacteria_Alphaproteobacteria_Rhodobacterales_Rhodobacteraceae_Sediminimonas_qiaohouensis_DSM_21189:0.09341475920568376):0.012556319981656738[15],((((Bacteria_Proteobacteria_Alphaproteobacteria_Rhodobacterales_Rhodobacteraceae_cArct_4215:0.05900173510758444,Bacteria_Proteobacteria_Alphaproteobacteria_Rhodobacterales_Rhodobacteraceae_cMeth_4145:0.022701706845648317):0.020728213969888998[100],'Bacteria_Proteobacteria_Alphaproteobacteria_Rhodobacterales_Rhodobacteraceae_Phaeobacter_gallaeciensis_2.10':0.026697103865399807):0.02674121450564826[100],Bacteria_Proteobacteria_Alphaproteobacteria_Rhodobacterales_Rhodobacteraceae_Silicibacter_pomeroyi_DSS_3:0.054963119917118775):0.010131836237392555[53],Bacteria_Proteobacteria_Alphaproteobacteria_Rhodobacterales_Rhodobacteraceae_Donghicola_xiamenensis_DSM_18339:0.04866174972963444):0.015941204279714682[83]):0.011657598927528046[28],(((((Bacteria_Proteobacteria_Alphaproteobacteria_Rhodobacterales_Rhodobacteraceae_Loktanella_vestfoldensis_SKA53:0.11006869109737583,Bacteria_Proteobacteria_Alphaproteobacteria_Rhodobacterales_Rhodobacteraceae_Octadecabacter_arcticus_238:0.08880976295803489):0.020839448499326618[51],'Bacteria_Proteobacteria_Alphaproteobacteria_Rhodobacterales_Rhodobacteraceae_Roseobacter_Thalassiobium_sp._R2A62_genomic_scaffold_scf_1112329232034':0.06363842405384501):0.016724768379099864[58],Bacteria_Proteobacteria_Alphaproteobacteria_Rhodobacterales_Rhodobacteraceae_Thalassobacter_arenae_DSM_19593:0.08915889924079945):0.015267684836844264[53],(Bacteria_Proteobacteria_Alphaproteobacteria_Rhodobacterales_Rhodobacteraceae_Planktomarina_temperata_RCA23_DSM_22400_RCA23:0.09862984052835433,'Bacteria_Proteobacteria_Alphaproteobacteria_Rhodobacterales_Rhodobacteraceae_Jannaschia_sp._CCS1':0.09562937942850303):0.017954703566212427[37]):0.019381011925991842[48],((Bacteria_Proteobacteria_Alphaproteobacteria_Rhodobacterales_Rhodobacteraceae_Oceaniovalibus_guishaninsula_JLT2003:0.06786638054322802,'Bacteria_Proteobacteria_Alphaproteobacteria_Rhodobacterales_Rhodobacteraceae_Maribius_sp._MOLA_401':0.11068954600551395):0.04021390470819286[100],Bacteria_Proteobacteria_Alphaproteobacteria_Rhodobacterales_Rhodobacteraceae_Oceanicola_granulosus_HTCC2516:0.07480073689438038):0.02859894831795362[85]):0.013559441215695855[15]):0.00809796815734165[13],(((((Bacteria_Proteobacteria_Alphaproteobacteria_Rhodobacterales_Rhodobacteraceae_Pelagibaca_bermudensis_HTCC2601:0.01680836869642066,Bacteria_Proteobacteria_Alphaproteobacteria_Rhodobacterales_Rhodobacteraceae_Salipiger_mucosus_DSM_16094_scaffold_version:0.053148331491171596):0.01870512200376684[92],'Bacteria_Proteobacteria_Alphaproteobacteria_Rhodobacterales_Rhodobacteraceae_Citreicella_sp._SE45':0.02892013526776127):0.024155307753366717[96],Bacteria_Proteobacteria_Alphaproteobacteria_Rhodobacterales_Rhodobacteraceae_Sagittula_stellata_E_37_unfinished_sequence:0.07296266962007536):0.02667348512914991[99],'Bacteria_Proteobacteria_Alphaproteobacteria_Rhodobacterales_Rhodobacteraceae_Roseivivax_sp._22II_s10s':0.09638287256103428):0.02738104537703112[100],'Bacteria_Proteobacteria_Alphaproteobacteria_Rhodobacterales_Rhodobacteraceae_Roseovarius_sp._217':0.07750023027868336):0.027860700985407938[99]):0.011889804541918902[13],((Bacteria_Proteobacteria_Alphaproteobacteria_Rhodobacterales_Rhodobacteraceae_Actibacterium_mucosum_KCTC_23349:0.07266204482870586,Bacteria_Proteobacteria_Alphaproteobacteria_Rhodobacterales_Rhodobacteraceae_Dinoroseobacter_shibae_DFL_12:0.07782018199772489):0.026202760444763218[80],'Bacteria_Proteobacteria_Alphaproteobacteria_Rhodobacterales_Rhodobacteraceae_Maritimibacter_sp._HL_12':0.08598520058289649):0.006553262937736726[16]):0.011932196485437974[28],'Bacteria_Proteobacteria_Alphaproteobacteria_Rhodobacterales_Rhodobacteraceae_Celeribacter_sp._P73_complete_genome':0.08623992416446535):0.02937345837966232[79],((((Ga0172382_10011512:0.06015846091494481,Ga0172382_10042257:0.033945369565463235):0.06022869169039158[93],Bacteria_Proteobacteria_Alphaproteobacteria_Rhodobacterales_Rhodobacteraceae_Rubellimicrobium_thermophilum_DSM_16684:0.12851963715772508):0.041104247973964725[90],Bacteria_Proteobacteria_Alphaproteobacteria_Rhodobacterales_Rhodobacteraceae_Ketogulonigenium_vulgarum_WSH_001:0.08912243758725502):0.021812488852093637[85],Bacteria_Proteobacteria_Alphaproteobacteria_Rhodobacterales_Rhodobacteraceae_Wenxinia_marina_DSM_24838:0.08122855564735953):0.031140384275675892[83]):0.026034576922787878[88],((((Ga0172378_10014102:0.058340740451073625,Bacteria_Proteobacteria_Alphaproteobacteria_Rhodobacterales_Rhodobacteraceae_Rhodobacter_capsulatus_SB1003:0.053277759476180186):0.027677888309368015[99],(Bacteria_Proteobacteria_Alphaproteobacteria_Rhodobacterales_Rhodobacteraceae_CG_Rhodob_01:0.09353413334922278,Bacteria_Proteobacteria_Alphaproteobacteria_Rhodobacterales_Rhodobacteraceae_Pseudorhodobacter_ferrugineus_DSM_5888:0.07592714760618824):0.04123500102612976[100]):0.014146084972621153[46],(Ga0172382_10005338:0.0760381903229761,Bacteria_Proteobacteria_Alphaproteobacteria_Rhodobacterales_Rhodobacteraceae_Catellibacterium_nectariphilum_DSM_15620:0.08089146961731775):0.02431445426598655[82]):0.015136210834114205[50],((Bacteria_Proteobacteria_Alphaproteobacteria_Rhodobacterales_Rhodobacteraceae_Thioclava_pacifica_DSM_10166:0.06574590111212153,Bacteria_Proteobacteria_Alphaproteobacteria_Rhodobacterales_Rhodobacteraceae_Paracoccus_denitrificans_PD1222_1:0.0867053592610807):0.014370612353167722[28],('Bacteria_Proteobacteria_Alphaproteobacteria_Rhodobacterales_Rhodobacteraceae_Defluviimonas_sp._20V17':0.0705002480464465,Bacteria_Proteobacteria_Alphaproteobacteria_Rhodobacterales_Rhodobacteraceae_Haematobacter_missouriensis_CCUG_52307:0.07761528654106753):0.015620112585222223[64]):0.007363494440718377[14]):0.033796208165026265[99]):0.1318271195939782[100],Bacteria_Proteobacteria_Alphaproteobacteria_Rhizobiales_Methylobacteriaceae_Meganema_perideroedes_DSM_15528:0.21277402572924053):0.08918700468888963[100],((((((Bacteria_Proteobacteria_Alphaproteobacteria_Caulobacterales_Caulobacteraceae_Woodsholea_maritima_DSM_17123:0.11985376888368426,Bacteria_Proteobacteria_Alphaproteobacteria_Rhodobacterales_Hyphomonadaceae_Oceanicaulis_alexandrii_HTCC2633:0.106504243654447):0.05541363501409435[100],Bacteria_Proteobacteria_Alphaproteobacteria_Rhodobacterales_Hyphomonadaceae_Maricaulis_maris_MCS10:0.1303430167133275):0.0634442289078776[100],(Bacteria_Proteobacteria_Alphaproteobacteria_Rhodobacterales_Hyphomonadaceae_Robiginitomaculum_antarcticum_DSM_21748:0.14186949787145497,Bacteria_Proteobacteria_Alphaproteobacteria_Rhodobacterales_Hyphomonadaceae_Hellea_balneolensis_DSM_19091:0.12639071445004824):0.14189496807398871[100]):0.04473913667810292[96],((Bacteria_Proteobacteria_Alphaproteobacteria_Rhodobacterales_Hyphomonadaceae_Henriciella_marina_DSM_19595:0.13500631768122817,Bacteria_Proteobacteria_Alphaproteobacteria_Rhodobacterales_Hyphomonadaceae_Hyphomonas_neptunium_ATCC_15444:0.15519907838489644):0.1052403663911683[100],Bacteria_Proteobacteria_Alphaproteobacteria_Rhodobacterales_Hyphomonadaceae_Hirschia_baltica_ATCC_49814:0.18119088850890774):0.09816940381875972[100]):0.022251832508275005[53],(((Bacteria_Proteobacteria_Alphaproteobacteria_Caulobacterales_Caulobacteraceae_Phenylobacterium_zucineum_HLK1:0.056592929406928505,Ga0172382_10000703:0.06457585675086719):0.05248711561516339[100],(Bacteria_Proteobacteria_Alphaproteobacteria_Caulobacterales_Caulobacteraceae_Caulobacter_segnis_ATCC_21756:0.07637988203956336,Ga0172380_10000068:0.10033574586284377):0.026640878074909224[100]):0.03940621459630833[100],(Bacteria_Proteobacteria_Alphaproteobacteria_Caulobacterales_Caulobacteraceae_Asticcacaulis_excentricus_CB_48_1:0.11080401217570035,Bacteria_Proteobacteria_Alphaproteobacteria_Caulobacterales_Caulobacteraceae_Brevundimonas_subvibrioides_ATCC_15264:0.16825099453395786):0.04817397430998405[100]):0.15717979461306664[100]):0.04629356524887873[76],Bacteria_Proteobacteria_Alphaproteobacteria_Parvularculales_Parvularculaceae_Parvularcula_bermudensis_HTCC2503:0.3149022403154773):0.042976495632204514[99]):0.025569583663618722[58]):0.05107213973774938[100],(((((((((Bacteria_Proteobacteria_Alphaproteobacteria_Sphingomonadales_Erythrobacteraceae_Erythrobacter_litoralis_HTCC2594:0.03695406832600989,Bacteria_Proteobacteria_Alphaproteobacteria_Sphingomonadales_Erythrobacteraceae_Altererythrobacter_ishigakiensis_ATCC_BAA_2084:0.046026233885544166):0.02048959297733388[47],'Bacteria_Proteobacteria_Alphaproteobacteria_Sphingomonadales_Sphingomonadaceae_Citromicrobium_sp._JLT1363':0.06580889562879566):0.03618916505558811[100],'Bacteria_Proteobacteria_Alphaproteobacteria_Sphingomonadales_Erythrobacteraceae_Porphyrobacter_sp._AAP82':0.06471346312028992):0.05496530066335481[100],((Ga0172382_10027754:0.06549081590972117,Ga0172382_10000527:0.04727255676288511):0.028283140267459483[99],'Bacteria_Proteobacteria_Alphaproteobacteria_Sphingomonadales_Sphingomonadaceae_Novosphingobium_sp._PP1Y':0.06967634955401847):0.020347093509642367[47]):0.047328527902132045[100],'Bacteria_Proteobacteria_Alphaproteobacteria_Sphingomonadales_Sphingomonadaceae_Blastomonas_sp._AAP53':0.10779137699099683):0.03196104580649006[99],(Bacteria_Proteobacteria_Alphaproteobacteria_Sphingomonadales_Sphingomonadaceae_Sphingobium_japonicum_UT26S_1:0.10077174428205904,Bacteria_Proteobacteria_Alphaproteobacteria_Sphingomonadales_Sphingomonadaceae_Sphingopyxis_alaskensis_RB2256:0.09601964781492711):0.02233025746960049[81]):0.03138556647733637[95],(Bacteria_Proteobacteria_Alphaproteobacteria_Sphingomonadales_Sphingomonadaceae_Zymomonas_mobilis_mobilis_CP4:0.13412464444310013,Bacteria_Proteobacteria_Alphaproteobacteria_Sphingomonadales_Sphingomonadaceae_Sphingomonas_wittichii_RW1:0.08430979680614703):0.04137451815720894[100]):0.05470360868086077[97],(Ga0172382_10000096:0.1676304240071298,'Bacteria_Proteobacteria_Alphaproteobacteria_Sphingomonadales_Sphingomonadaceae_Sandarakinorhabdus_sp._AAP62':0.1412236891050287):0.0630992521459004[100]):0.176329964548962[100],Bacteria_Proteobacteria_Alphaproteobacteria_Kordiimonadales_Kordiimonas_gwangyangensis_DSM_19435:0.18485332361289952):0.09122091500432683[100]):0.04338094260717895[93],(Bacteria_Proteobacteria_Alphaproteobacteria_Rhodospirillales_Rhodospirillaceae_Tistrella_mobilis_KA081020_065:0.20928209827811006,Bacteria_Proteobacteria_Alphaproteobacteria_unclassified_Alphaproteobacteria_Geminicoccus_roseus_DSM_18922:0.3321283994453683):0.06930750562485466[68]):0.03794174271735207[22],((Bacteria_Proteobacteria_Alphaproteobacteria_Rhizobiales_Methylocystaceae_Terasakiella_pusilla_DSM_6293:0.21792778862896078,Bacteria_Proteobacteria_Alphaproteobacteria_Rhodospirillales_Rhodospirillaceae_Thalassospira_profundimaris_WP0211:0.21652553508553574):0.045831592105860075[86],Bacteria_Proteobacteria_Alphaproteobacteria_Sneathiellales_Sneathiellaceae_Sneathiella_glossodoripedis_JCM_23214:0.2709652721301432):0.026695479100129038[13]):0.02039997595241294[15],((((Bacteria_Proteobacteria_Alphaproteobacteria_Alpha_proteobacterium_IMCC1322:0.28819246917846675,Bacteria_Proteobacteria_Alphaproteobacteria_Rhodospirillales_Rhodospirillaceae_Nisaea_denitrificans_DSM_18348:0.15474582701120543):0.03738468500119163[53],(Bacteria_Proteobacteria_Alphaproteobacteria_Rhodospirillales_Rhodospirillaceae_Candidatus_Endolissoclinum_faulkneri_L5:0.23585264552295282,Bacteria_Proteobacteria_Alphaproteobacteria_Rhodospirillales_Rhodospirillaceae_Thalassobaculum_salexigens_DSM_19539:0.05078413582920538):0.12307965367928464[100]):0.051267890550164896[96],Bacteria_Proteobacteria_Alphaproteobacteria_Rhodospirillales_Rhodospirillaceae_Oceanibaculum_indicum_P24:0.22415480566353763):0.027431994539187432[49],((Bacteria_Proteobacteria_Alphaproteobacteria_Rhodospirillales_Rhodospirillaceae_Rhodovibrio_salinarum_DSM_9154:0.2703961828493746,Bacteria_Proteobacteria_Alphaproteobacteria_Rhodospirillales_Rhodospirillaceae_Fodinicurvata_sediminis_DSM_21159:0.19460891362146437):0.04396268949836335[67],Bacteria_Proteobacteria_Alphaproteobacteria_Kiloniellales_Kiloniellaceae_Kiloniella_laminariae_DSM_19542:0.2092916080323728):0.06665377041483733[99]):0.03118807471960494[29]):0.02275884842073106[12],(((((((((((((Bacteria_Proteobacteria_Alphaproteobacteria_Rhodospirillales_Acetobacteraceae_Gluconobacter_oxydans_621H:0.03696759657973958,Bacteria_Proteobacteria_Alphaproteobacteria_Rhodospirillales_Acetobacteraceae_Saccharibacter_floricola_DSM_15669:0.1097867734185094):0.035439410398633076[100],(Bacteria_Proteobacteria_Alphaproteobacteria_Rhodospirillales_Acetobacteraceae_Asaia_platycodi_JCM_25414:0.06449755513491962,Bacteria_Proteobacteria_Alphaproteobacteria_Rhodospirillales_Acetobacteraceae_Kozakia_baliensis_SR_745:0.028068631644093678):0.022273355911103465[100]):0.05735529871322065[100],Bacteria_Proteobacteria_Alphaproteobacteria_Rhodospirillales_Acetobacteraceae_Acetobacter_pasteurianus_386B:0.08677188984366735):0.012166534846625776[33],(Bacteria_Proteobacteria_Alphaproteobacteria_Rhodospirillales_Acetobacteraceae_Gluconacetobacter_xylinus_NBRC_3288:0.058561009494189964,Bacteria_Proteobacteria_Alphaproteobacteria_Rhodospirillales_Acetobacteraceae_Gluconacetobacter_diazotrophicus_PAl_5:0.0420720881507588):0.027730512548993058[100]):0.022580371041996283[71],'Bacteria_Proteobacteria_Alphaproteobacteria_Rhodospirillales_Acetobacteraceae_Commensalibacter_sp._MX_MONARCH01':0.15993723651692893):0.05454710043085109[100],Bacteria_Proteobacteria_Alphaproteobacteria_Rhodospirillales_Acetobacteraceae_Granulibacter_bethesdensis_CGDNIH1:0.11424263784827549):0.03417037956465485[99],(Bacteria_Proteobacteria_Alphaproteobacteria_Rhodospirillales_Acetobacteraceae_Acidiphilium_multivorum_AIU301:0.12051615878811583,Bacteria_Proteobacteria_Alphaproteobacteria_Rhodospirillales_Acetobacteraceae_Acidocella_facilis_ATCC_35904:0.13427238798457886):0.04741558936711554[100]):0.05306114677201812[100],((Bacteria_Proteobacteria_Alphaproteobacteria_Rhodospirillales_Acetobacteraceae_Belnapia_moabensis_DSM_16746:0.09692556367125871,Bacteria_Proteobacteria_Alphaproteobacteria_Rhodospirillales_Acetobacteraceae_Roseomonas_mucosa_ATCC_BAA_692:0.10249151323037475):0.03658809674850794[97],Bacteria_Proteobacteria_Alphaproteobacteria_Rhodospirillales_Acetobacteraceae_Rubritepida_flocculans_DSM_14296:0.11457571883376483):0.05038277144836334[100]):0.04557235135242088[98],Bacteria_Proteobacteria_Alphaproteobacteria_Rhodospirillales_unclassified_Rhodospirillales_Elioraea_tepidiphila_DSM_17972:0.13575678902607757):0.14569039323154653[100],Bacteria_Proteobacteria_Alphaproteobacteria_Rhodospirillales_unclassified_Rhodospirillales_Reyranella_Reyranella_massiliensis_521_DSM_23428:0.2620183750017002):0.04598346667435482[57],((((Bacteria_Proteobacteria_Alphaproteobacteria_Rhodospirillales_Rhodospirillaceae_Azospirillum_amazonense_CBAmc:0.12290417126140607,Bacteria_Proteobacteria_Alphaproteobacteria_Rhodospirillales_Rhodospirillaceae_Azospirillum_irakense_DSM_11586:0.10951793626593576):0.04958820716028978[100],Bacteria_Proteobacteria_Alphaproteobacteria_Rhodospirillales_Rhodospirillaceae_Skermanella_stibiiresistens_SB22:0.11456853329672656):0.0714739599502443[100],Bacteria_Proteobacteria_Alphaproteobacteria_Rhodospirillales_Rhodospirillaceae_Inquilinus_limosus_DSM_16000:0.1707810740544904):0.038706784668624206[81],((Ga0172382_10003179:0.2606163866552351,Bacteria_Proteobacteria_Alphaproteobacteria_unclassified_Alphaproteobacteria_Micavibrio_aeruginosavorus_ARL_13:0.1755921513743388):0.057038384307942724[96],Ga0172380_10000070:0.23583469107853228):0.17355471818010493[100]):0.02506610468306647[22]):0.025019445341080804[8],((Ga0172382_10007147:0.09627984451112992,Bacteria_Proteobacteria_Alphaproteobacteria_Rhodospirillales_Rhodospirillaceae_Novispirillum_itersonii_ATCC_12639:0.11918239575119172):0.05138481773839798[100],(Bacteria_Proteobacteria_Alphaproteobacteria_Rhodospirillales_Rhodospirillaceae_Caenispirillum_salinarum_AK4:0.12458890811578316,Bacteria_Proteobacteria_Alphaproteobacteria_Rhodospirillales_Rhodospirillaceae_Rhodospirillum_rubrum_S1_ATCC_11170:0.169177840122535):0.034467521850348515[77]):0.08157112247780418[100]):0.011956726802067497[2],('Bacteria_Proteobacteria_Alphaproteobacteria_Rhodospirillales_Rhodospirillaceae_Dongia_sp._URHE0060':0.22427394950397606,Bacteria_Proteobacteria_Alphaproteobacteria_Rhodospirillales_Rhodospirillaceae_Magnetospirillum_magneticum_AMB_1:0.16587278996570154):0.04261030533627208[21]):0.029196498265539716[8]):0.06703063327990622[23],Ga0172380_10018426:0.3329610218490009):0.05123158859681887[96],Bacteria_Proteobacteria_Alphaproteobacteria_Rickettsiales_Rickettsiales_genera_incertae_sedis_Candidatus_Odyssella_thessalonicensis_L13:0.42367133418672376):0.06161670926677765[78],(((((((Bacteria_Proteobacteria_Alphaproteobacteria_Rickettsiales_Anaplasmataceae_Ehrlichia_chaffeensis_Osceola:0.1901114870234042,'Bacteria_Proteobacteria_Alphaproteobacteria_Rickettsiales_Anaplasmataceae_Anaplasma_marginale_St._Maries':0.30955624042378327):0.1745140566369794[100],Bacteria_Proteobacteria_Alphaproteobacteria_Rickettsiales_Anaplasmataceae_Wolbachieae_Wolbachia_endosymbiont_of_Drosophila_melanogaster:0.3620997087610256):0.18144757027836667[100],(Bacteria_Proteobacteria_Alphaproteobacteria_Rickettsiales_Anaplasmataceae_Candidatus_Xenolissoclinum_pacificiensis_L6:0.7119418556109927,Bacteria_Proteobacteria_Alphaproteobacteria_Rickettsiales_Anaplasmataceae_Neorickettsia_sennetsu_Miyayama:0.653254101754372):0.22049237420290035[100]):0.1373618630675124[100],Ga0172378_10002796:0.7576693693142094):0.03449048112899611[40],Bacteria_Proteobacteria_Alphaproteobacteria_Rickettsiales_Holosporaceae_Holospora_undulata_HU1:0.8024452493858583):0.06043206848626248[35],(Bacteria_Proteobacteria_Alphaproteobacteria_Rickettsiales_Rickettsiaceae_Rickettsieae_Orientia_tsutsugamushi_Boryong:0.44157741262426153,Bacteria_Proteobacteria_Alphaproteobacteria_Rickettsiales_Rickettsiaceae_Rickettsieae_Rickettsia_conorii_Malish_7:0.2786226050070044):0.2554677714938012[100]):0.03498197466276931[24],Bacteria_Proteobacteria_Alphaproteobacteria_Rickettsiales_Candidatus_Midichloria_mitochondrii_IricVA:0.547781773926578):0.061452209581834616[35]):0.05536482390948905[50],'Bacteria_Proteobacteria_Alphaproteobacteria_unclassified_Alphaproteobacteria_SAR11_cluster_Candidatus_Pelagibacter_sp._IMCC9063':0.7694538888242786):0.08564866677857319[100],'Bacteria_Proteobacteria_Alphaproteobacteria_Magnetococcales_Magnetococcaceae_Magnetococcus_sp._MC_1':0.4214209680392216):0.06869693971296087[89]):0.0716071444981683[97],Ga0172380_10031473:0.6687002442436425):0.0514988011858426[31],(((((((((((((Bacteria_Proteobacteria_deltaepsilon_subdivisions_Deltaproteobacteria_Desulfobacterales_Desulfobacteraceae_Desulfatirhabdium_butyrativorans_DSM_18734:0.29767850624827163,Bacteria_Proteobacteria_deltaepsilon_subdivisions_Deltaproteobacteria_Desulfobacterales_Desulfobacteraceae_Desulfonema_limicola:0.14932839169694168):0.037514468957983826[49],Bacteria_Proteobacteria_deltaepsilon_subdivisions_Deltaproteobacteria_Desulfobacterales_Desulfobacteraceae_Desulfosarcina_variabilis:0.21686360660823034):0.024320706353844646[37],Ga0172382_10002694:0.2766191519775387):0.03342112348458404[67],((Bacteria_Proteobacteria_deltaepsilon_subdivisions_Deltaproteobacteria_Desulfobacterales_Desulfobacteraceae_Desulfobotulus_alkaliphilus_ASO4_4:0.21952390858633075,Bacteria_Proteobacteria_deltaepsilon_subdivisions_Deltaproteobacteria_Desulfobacterales_Desulfobacteraceae_Desulforegula_conservatrix_Mb1Pa_DSM_13527:0.21566928274891994):0.06973021254000988[96],Bacteria_Proteobacteria_deltaepsilon_subdivisions_Deltaproteobacteria_Desulfobacterales_Desulfobacteraceae_Desulfococcus_oleovorans_Hxd3:0.28883815308024685):0.018498193936595797[24]):0.026324508639349098[28],(Bacteria_Proteobacteria_deltaepsilon_subdivisions_Deltaproteobacteria_Desulfobacterales_Desulfobacteraceae_Desulfatibacillum_alkenivorans_AK_01_Desulfatibacillum_alkenivorans_AK_01:0.25233522692605836,Bacteria_Proteobacteria_deltaepsilon_subdivisions_Deltaproteobacteria_Desulfobacterales_Desulfobacteraceae_Candidatus_Magnetoglobus_multicellularis_Araruama:0.3626896790126879):0.03664715151771292[31]):0.036793153698752246[54],((((Bacteria_Proteobacteria_deltaepsilon_subdivisions_Deltaproteobacteria_Desulfobacterales_Desulfobacteraceae_Desulfobacter_postgatei_2ac9:0.10476560472792729,Bacteria_Proteobacteria_deltaepsilon_subdivisions_Deltaproteobacteria_Desulfobacterales_Desulfobacteraceae_Desulfobacula_toluolica_Tol2:0.09210679942544964):0.015241072577579118[49],Bacteria_Proteobacteria_deltaepsilon_subdivisions_Deltaproteobacteria_Desulfobacterales_Desulfobacteraceae_Desulfospira_joergensenii_DSM_10085:0.058180701310234895):0.028744845419091725[67],Bacteria_Proteobacteria_deltaepsilon_subdivisions_Deltaproteobacteria_Desulfobacterales_Desulfobacteraceae_Desulfotignum_phosphitoxidans_FiPS_3:0.09689787417383):0.1534253100343963[100],Bacteria_Proteobacteria_deltaepsilon_subdivisions_Deltaproteobacteria_Desulfobacterales_Desulfobacteraceae_Desulfobacterium_autotrophicum_HRM2:0.1729501566982301):0.1827061718639813[100]):0.1024330653925749[100],Bacteria_Proteobacteria_deltaepsilon_subdivisions_Deltaproteobacteria_Desulfobacterales_Desulfobacteraceae_Desulfobacterium_anilini_DSM_4660:0.3725551678982435):0.034367085036663525[78],Bacteria_Proteobacteria_deltaepsilon_subdivisions_Deltaproteobacteria_Syntrophobacterales_Syntrophaceae_Desulfobacca_acetoxidans_DSM_11109:0.38437086141376353):0.030526966404966327[40],(((((((Ga0172380_10000396:0.016849407537828043,Ga0172379_10040926:0.014846145790574639):0.010756222291403716[43],Ga0172379_10041150:0.01848916995891381):0.008951874610842125[74],Ga0172380_10001757:0.023890416496518352):0.14636950000831828[100],Bacteria_Proteobacteria_deltaepsilon_subdivisions_Deltaproteobacteria_Desulfobacterales_Desulfobulbaceae_Desulfurivibrio_alkaliphilus_AHT2:0.18871315556034185):0.0524697303098316[95],Ga0172380_10014835:0.19193533555438824):0.06202526377731443[99],(((Ga0172378_10000120:0.17980599190997681,Bacteria_Proteobacteria_deltaepsilon_subdivisions_Deltaproteobacteria_Desulfobacterales_Desulfobulbaceae_Desulfotalea_psychrophila_LSv54:0.10621786056139326):0.04827691403012002[99],Bacteria_Proteobacteria_deltaepsilon_subdivisions_Deltaproteobacteria_Desulfobacterales_Desulfobulbaceae_Desulfocapsa_sulfexigens_DSM_10523:0.17427064355158928):0.12434141886247962[100],(Bacteria_Proteobacteria_deltaepsilon_subdivisions_Deltaproteobacteria_Desulfobacterales_Desulfobulbaceae_Desulfobulbus_propionicus_DSM_2032:0.2028853080625983,Ga0172382_10003769:0.14916458890987183):0.10582158710383904[100]):0.06687453389466214[100]):0.19686602298438016[100],Bacteria_Proteobacteria_deltaepsilon_subdivisions_Deltaproteobacteria_Desulfarculales_Desulfarculaceae_Desulfarculus_baarsii_DSM_2075:0.33953602659350013):0.06257790930313467[79]):0.03832444290939119[51],((((((((Bacteria_Proteobacteria_deltaepsilon_subdivisions_Deltaproteobacteria_Desulfovibrionales_Desulfomicrobiaceae_Desulfomicrobium_baculatum_DSM_4028:0.2294684669606628,Ga0172377_10006355:0.30549284329375315):0.011605691380519367[79],Ga0172377_10000729:0.19607060228016326):0.0377094555297921[65],(Bacteria_Proteobacteria_deltaepsilon_subdivisions_Deltaproteobacteria_Desulfovibrionales_Desulfohalobiaceae_Desulfonatronospira_thiodismutans_ASO3_1_unfinished_sequence:0.17616226285695324,Bacteria_Proteobacteria_deltaepsilon_subdivisions_Deltaproteobacteria_Desulfovibrionales_Desulfohalobiaceae_Desulfonatronovibrio_hydrogenovorans_DSM_9292:0.1483350332588711):0.1425486500492692[100]):0.05591224941293493[97],(Bacteria_Proteobacteria_deltaepsilon_subdivisions_Deltaproteobacteria_Desulfovibrionales_Desulfohalobiaceae_Desulfohalobium_retbaense_DSM_5692:0.2089956277397027,Bacteria_Proteobacteria_deltaepsilon_subdivisions_Deltaproteobacteria_Desulfovibrionales_Desulfohalobiaceae_Desulfovermiculus_halophilus_DSM_18834:0.2674414367761502):0.09018228607932821[100]):0.032633982160595654[59],Bacteria_Proteobacteria_deltaepsilon_subdivisions_Deltaproteobacteria_Desulfovibrionales_Desulfonatronumaceae_Desulfonatronum_thioautotrophicum_ASO4_1:0.30543900527232193):0.03170039112311951[44],(((Bacteria_Proteobacteria_deltaepsilon_subdivisions_Deltaproteobacteria_Desulfovibrionales_Desulfovibrionaceae_Bilophila_wadsworthia_3_1_6:0.09543535096629485,'Bacteria_Proteobacteria_deltaepsilon_subdivisions_Deltaproteobacteria_Desulfovibrionales_Desulfovibrionaceae_Desulfovibrio_desulfuricans_subsp._desulfuricans_str._ATCC_27774':0.13952115151749567):0.051361179009532076[67],Bacteria_Proteobacteria_deltaepsilon_subdivisions_Deltaproteobacteria_Desulfovibrionales_Desulfovibrionaceae_Lawsonia_intracellularis_PHEMN1_00:0.20428197476270027):0.12283735822870723[100],(Ga0172382_10005917:0.009785538770188396,Bacteria_Proteobacteria_deltaepsilon_subdivisions_Deltaproteobacteria_Desulfovibrionales_Desulfovibrionaceae_Desulfocurvus_vexinensis_DSM_17965:0.023158492561713206):0.18656598633661847[100]):0.057512859834535135[100]):0.27925387628427156[100],(((((Bacteria_Thermodesulfobacteria_Thermodesulfobacteria_Thermodesulfobacteriales_Thermodesulfobacteriaceae_Thermodesulfobacterium_hveragerdense_DSM_12571:0.005855537045661219,Bacteria_Thermodesulfobacteria_Thermodesulfobacteria_Thermodesulfobacteriales_Thermodesulfobacteriaceae_Thermodesulfobacterium_thermophilum_DSM_1276:0.005819883626451805):0.03320191286178309[100],Bacteria_Thermodesulfobacteria_Thermodesulfobacteria_Thermodesulfobacteriales_Thermodesulfobacteriaceae_Thermodesulfobacterium_commune_DSM_2178:0.013888547537176166):0.07583228101900197[100],Bacteria_Thermodesulfobacteria_Thermodesulfobacteria_Thermodesulfobacteriales_Thermodesulfobacteriaceae_Thermodesulfobacterium_hydrogeniphilum_DSM_14290:0.05714510059622935):0.028656391107321433[98],'Bacteria_Thermodesulfobacteria_Thermodesulfobacteria_Thermodesulfobacteriales_Thermodesulfobacteriaceae_Thermodesulfobacterium_sp._OPB45':0.05696602647832538):0.21599007026826955[100],(Bacteria_Thermodesulfobacteria_Thermodesulfobacteria_Thermodesulfobacteriales_Thermodesulfobacteriaceae_Thermodesulfatator_atlanticus_DSM_21156:0.03393858440362907,Bacteria_Thermodesulfobacteria_Thermodesulfobacteria_Thermodesulfobacteriales_Thermodesulfobacteriaceae_Thermodesulfatator_indicus_CIR29812_DSM_15286:0.03906359220390376):0.11349177749212025[100]):0.24780068543904132[100]):0.04056780541328875[32],(Bacteria_Proteobacteria_deltaepsilon_subdivisions_Deltaproteobacteria_Syntrophobacterales_Syntrophobacteraceae_Thermodesulforhabdus_norvegica_DSM_9990:0.20547800582233755,Bacteria_Proteobacteria_deltaepsilon_subdivisions_Deltaproteobacteria_Syntrophobacterales_Syntrophobacteraceae_Syntrophobacter_fumaroxidans_MPOB:0.2618773875391187):0.14437079929948915[100]):0.03037969594939005[31]):0.04011186176209236[43],(Ga0172382_10008453:0.4393147591974733,Bacteria_Proteobacteria_deltaepsilon_subdivisions_Deltaproteobacteria_Syntrophobacterales_Syntrophaceae_Desulfomonile_tiedjei_DCB_1_DSM_6799:0.3815267579004997):0.11247676226328629[98]):0.04113812656130067[17],((((((((Bacteria_Proteobacteria_deltaepsilon_subdivisions_Deltaproteobacteria_Desulfuromonadales_Desulfuromonadaceae_Desulfuromonas_acetoxidans_DSM_684:0.06567832404777851,Ga0172377_10039431:0.12635317314920336):0.031156644110211573[95],Ga0172377_10004905:0.09889034914291628):0.11508534760187672[100],(Bacteria_Proteobacteria_deltaepsilon_subdivisions_Deltaproteobacteria_Desulfuromonadales_Pelobacteraceae_Pelobacter_carbinolicus_DSM_2380:0.15619774478233817,Ga0172382_10000800:0.14924798670672912):0.032634803613950325[84]):0.023173005736375263[56],Bacteria_Proteobacteria_deltaepsilon_subdivisions_Deltaproteobacteria_Desulfuromonadales_Geobacteraceae_Geoalkalibacter_ferrihydriticus_DSM_17813:0.16407843088937435):0.03771268609899403[78],(Bacteria_Proteobacteria_deltaepsilon_subdivisions_Deltaproteobacteria_Desulfuromonadales_Desulfuromonadaceae_Desulfuromusa_kysingii_DSM_7343:0.13013430922406677,Bacteria_Proteobacteria_deltaepsilon_subdivisions_Deltaproteobacteria_Desulfuromonadales_Geobacteraceae_Geopsychrobacter_electrodiphilus_DSM_16401:0.11603025735688233):0.10708928351236269[100]):0.13364391147102728[100],(((Ga0172380_10025022:0.05016500403687996,Ga0172380_10004725:0.014526735834749172):0.10226477443084203[100],Ga0172380_10009153:0.11242724046399699):0.06913636277372115[100],(Ga0172377_10008412:0.056246045495425,Bacteria_Proteobacteria_deltaepsilon_subdivisions_Deltaproteobacteria_Desulfuromonadales_Geobacteraceae_Geobacter_lovleyi_SZ:0.06267579353052755):0.06489160244602621[100]):0.19296506572144212[100]):0.1208250909483044[100],((((((Ga0172382_10002735:0.000001,Ga0172381_10002113:0.000001):0.17215123862004367[100],(Ga0172379_10039682:0.009782434272628038,Ga0172379_10009930:0.010541966946366887):0.14078920330746714[100]):0.031780092728726395[72],Ga0172382_10000503:0.22480799807005347):0.02967761287317927[54],((Ga0172382_10037484:0.12050699057140157,'Bacteria_Proteobacteria_deltaepsilon_subdivisions_Deltaproteobacteria_Syntrophobacterales_Syntrophaceae_Smithella_sp._F21':0.10845685749488876):0.09797797136057174[100],Bacteria_Proteobacteria_deltaepsilon_subdivisions_Deltaproteobacteria_Syntrophobacterales_Syntrophaceae_Syntrophus_aciditrophicus_SB:0.2230235326597656):0.04180757105032562[87]):0.04881495869195618[99],((Ga0172382_10000009:0.1506252508664585,Ga0172382_10000139:0.17499249366446312):0.12723566854103563[100],Ga0172382_10024341:0.22175292579830153):0.028492111733187997[40]):0.18545845882144008[100],Bacteria_Proteobacteria_RIFCSPLOWO2_02_FULL_Proteobacteria_53_8:0.5193695569691124):0.05180875930623774[10]):0.02838742552566753[4],((((Bacteria_CP_Dadabacteria_LAC_S2O1_bin1_lowgc_50_27:0.000001,Bacteria_CP_Dadabacteria_LAC_COMBO_lowCGbin1_like_51_6394_partial:0.000433):0.04781899361014119[100],Bacteria_CP_Dadabacteria_LAC_COMBO_lowcgbin1_52_3600:0.03008646679006244):0.29097662381389844[100],(Bacteria_CP_Dadabacteria_CSP1_2:0.22463970370452335,Bacteria_Dadabacteria_RIFCSPHIGHO2_12_FULL_Dadabacteria_53_21:0.25857438830080426):0.09145482284240636[96]):0.2779421468682446[100],(Ga0172382_10016968:0.1348035039504607,Bacteria_Proteobacteria_deltaepsilon_subdivisions_Deltaproteobacteria_Syntrophobacterales_Syntrophorhabdaceae_Syntrophorhabdus_aromaticivorans_UI:0.14477849885168137):0.3404073136718244[100]):0.06397024461129375[33]):0.023937803485685638[4]):0.04551052111639908[26],((((((Bacteria_Proteobacteria_deltaepsilon_subdivisions_Deltaproteobacteria_Myxococcales_Nannocystineae_Nannocystaceae_Plesiocystis_pacifica_SIR_1_unfinished_sequence:0.08077796233416068,Bacteria_Proteobacteria_deltaepsilon_subdivisions_Deltaproteobacteria_Myxococcales_Sorangiineae_Sandaracinaceae_Sandaracinus_amylolyticus_DSM_53668:0.11914366753465488):0.16887333696864237[100],Bacteria_Proteobacteria_deltaepsilon_subdivisions_Deltaproteobacteria_Myxococcales_Nannocystineae_Nannocystaceae_Nannocystis_exedens_ATCC_25963:0.2369427002333544):0.23330727256941586[100],Bacteria_Proteobacteria_deltaepsilon_subdivisions_Deltaproteobacteria_Myxococcales_Nannocystineae_Kofleriaceae_Haliangium_ochraceum_DSM_14365:0.3926679216130293):0.09635538342455341[100],(Bacteria_Proteobacteria_deltaepsilon_subdivisions_Deltaproteobacteria_Myxococcales_Sorangiineae_Polyangiaceae_Sorangium_cellulosum_So0157_2:0.07102296313898071,Bacteria_Proteobacteria_deltaepsilon_subdivisions_Deltaproteobacteria_Myxococcales_Sorangiineae_Polyangiaceae_Chondromyces_apiculatus_DSM_436:0.07571758594879219):0.383640837708052[100]):0.059825978310228844[99],((((Bacteria_Proteobacteria_deltaepsilon_subdivisions_Deltaproteobacteria_Myxococcales_Cystobacterineae_Myxococcaceae_Myxococcus_xanthus_DK_1622:0.04458089227199613,Bacteria_Proteobacteria_deltaepsilon_subdivisions_Deltaproteobacteria_Myxococcales_Cystobacterineae_Myxococcaceae_Corallococcus_coralloides_DSM_2259:0.04474285534799227):0.02125694401757361[94],Bacteria_Proteobacteria_deltaepsilon_subdivisions_Deltaproteobacteria_Myxococcales_Cystobacterineae_Cystobacteraceae_Stigmatella_aurantiaca_DW43_1:0.06652619477349386):0.021209141227824357[73],Bacteria_Proteobacteria_deltaepsilon_subdivisions_Deltaproteobacteria_Myxococcales_Cystobacterineae_Cystobacteraceae_Cystobacter_violaceus_Cb_vi76:0.06395309974727414):0.20848038522301504[100],Bacteria_Proteobacteria_deltaepsilon_subdivisions_Deltaproteobacteria_Myxococcales_Cystobacterineae_Myxococcaceae_Anaeromyxobacter_dehalogenans_2CP_C:0.26035678125858963):0.17784321068165987[100]):0.09325353589120233[100],((((Bacteria_Bdellovibrio_Bdellovibrionales_Bacteroivoracaceae_GWA2_Bdellovibrionales_49_15:0.3058129192672494,Bacteria_Proteobacteria_deltaepsilon_subdivisions_Deltaproteobacteria_Bdellovibrionales_Bacteriovoracaceae_Bacteriovorax_marinus_SJ:0.19439772357167096):0.06185272623793958[65],Bacteria_Bdellovibrio_Bdellovibrionales_Bacteroivoracaceae_RIFOXYA1_FULL_Bacteriovorax_38_20:0.23861862411095336):0.3906755487088134[100],(Bacteria_Bdellovibrio_Bdellovibrionales_Bdellovibrionaceae_GWA1_Bdellovibrionales_52_35:0.14596011955486876,Bacteria_Bdellovibrio_Bdellovibrionales_Bacteroivoracaceae_RIFOXYD1_FULL_Bdellovibrionales_53_11:0.17599866141478104):0.31140577251154467[100]):0.06754640341772822[88],(Bacteria_Bdellovibrio_Bdellovibrionales_RBG_16_Bdellovibrionales_40_8:0.31857738591255425,Bacteria_Proteobacteria_deltaepsilon_subdivisions_Deltaproteobacteria_Bdellovibrionales_Bdellovibrionaceae_Bdellovibrio_bacteriovorus_HD100:0.24688818769996868):0.308772519241717[100]):0.08567922292068664[94]):0.04674570942985801[54]):0.03394657807522039[24]):0.03155543135944905[10],((((((((((((((((Ga0172378_10018663:0.000001,Ga0172377_10022505:0.000001):0.03449454202242741[100],Ga0172377_10028432:0.05324594062452803):0.01843527010029611[99],((Ga0172378_10004952:0.000001,Ga0172377_10006028:0.000454):0.06278432692939528[100],'Bacteria_Spirochaetes_Spirochaetia_Spirochaetales_Spirochaetaceae_Spirochaeta_sp._Buddy':0.040291034092511335):0.02392829771052085[59]):0.02308598844025811[64],Ga0172382_10007934:0.16277433827129428):0.03623563896463455[100],(((Ga0172378_10040148:0.000001,Ga0172377_10008572:0.002016347661546458):0.015671588181597418[98],(Ga0172378_10000653:0.000001,Ga0172377_10001536:0.000001):0.03232324382784224[100]):0.09611353589743699[100],((Ga0172381_10005576:0.000858,Ga0172377_10012561:0.0012113301773908347):0.000012[68],Ga0172378_10000423:0.000001):0.06857996442600856[100]):0.0954709268357421[100]):0.029328674926566656[98],((Ga0172378_10008497:0.000001,Ga0172377_10001928:0.000001):0.15302652367270975[100],Ga0172378_10004238:0.20712720880375945):0.03398436349733469[98]):0.02309832460480621[86],(Ga0172378_10000313:0.000657,Ga0172377_10012415:0.000001):0.05952052833451171[100]):0.02523007819985157[92],((Ga0172378_10004802:0.14951606674777418,Ga0172377_10005663:0.1587087691682969):0.041048756562932276[97],Ga0172377_10047789:0.20418464559649774):0.06430622744171899[100]):0.02733659245185205[92],((((((((Ga0172377_10001516:0.000001,Ga0172382_10042815:0.000001):0.04660319013786074[100],Ga0172382_10044149:0.06446282965928551):0.06076108325103613[100],(Ga0172382_10040137:0.07673648608237693,Ga0172382_10003807:0.11378813572692703):0.025909768722200077[94]):0.017008148575951676[43],(Ga0172382_10002369:0.08507680322166156,Ga0172382_10017258:0.06643653830528162):0.02654705539595703[99]):0.030444875513767844[89],(Ga0172382_10023165:0.000001,Ga0172377_10037328:0.000823):0.08341301564593451[100]):0.013744613307524745[75],Ga0172382_10001824:0.025943003008843224):0.028697636092637424[96],((Ga0172382_10000175:0.000001,Ga0172377_10000248:0.000001):0.03962969434555319[100],Ga0172377_10019102:0.04968654140488482):0.031112174111167956[100]):0.01292046699564775[54],Ga0172381_10017249:0.07026863627904945):0.06138683502801756[100]):0.18674672174842577[100],(((((Bacteria_Spirochaetes_Spirochaetia_Spirochaetales_Spirochaetaceae_Treponema_denticola_ATCC_35405:0.18288062842281017,Ga0172377_10039566:0.12129698734188521):0.032768642487526645[74],Ga0172378_10001835:0.23263229060589508):0.04969135771949196[100],Ga0172379_10021142:0.17564760565346837):0.0402674354869994[99],((Bacteria_Spirochatetes_Spirochaetia_Spirochaetales_GWB1_Spirochaetes_59_5:0.07854527541308798,Bacteria_Spirochatetes_Spirochaetia_Spirochaetales_RIFOXYC1_FULL_Spirochaetes_54_7:0.06692111351277541):0.14900102876487153[100],Ga0172381_10010318:0.19180762508537352):0.09541222801202043[100]):0.08713849496136872[100],('Bacteria_Spirochaetes_Spirochaetia_Spirochaetales_Spirochaetaceae_Spirochaeta_sp._L21_RPul_D2':0.28545693348270795,Bacteria_Spirochatetes_Spirochaetia_Spirochaetales_RBG_16_Spirochaete_67_19:0.3647353479714597):0.04298484628039878[32]):0.03862065375312396[24]):0.09833743753272284[100],Bacteria_Spirochaetes_Spirochaetia_Spirochaetales_Spirochaetaceae_Borrelia_garinii_PBi_linear:0.4827334625802919):0.20736440033466996[100],(Bacteria_Spirochaetes_Spirochaetia_Spirochaetales_Brachyspiraceae_Brachyspira_murdochii_DSM_12563:0.5343628584106193,Bacteria_Spirochaetes_Spirochaetia_Spirochaetales_Brevinemataceae_Brevinema_andersonii_ATCC_43811:0.7628336214667106):0.06269268171076092[77]):0.07231054177972007[87],((Bacteria_Spirochaetes_Spirochaetia_Spirochaetales_Leptospiraceae_Leptospira_biflexa_serovar_Patoc_strain_Patoc_1_Paris_I:0.39130289587541744,Bacteria_Spirochaetes_Spirochaetia_Leptospirales_Leptospiraceae_Leptonema_illini_3055_DSM_21528:0.47513261617818126):0.11184578692716407[96],Bacteria_Spirochaetes_Spirochaetia_Spirochaetales_Leptospiraceae_Turneriella_parva_H_DSM_21527:0.586598449344292):0.22521135761691013[100]):0.06663255997132156[79],(((Bacteria_CP_NKB19_candidate_division_NKB19_bacterium_JGI_0000059_N10_TAbiofilm_001_172:0.0025480135675568505,Bacteria_CP_NKB19_candidate_division_NKB19_bacterium_JGI_0000039_J10_Combined_Assembly_NKB19_1__NKB19:0.000001):0.3479704720866308[100],Ga0172382_10003317:0.34845976265094114):0.2877404577420348[100],Bacteria_CG_CP02_CG_CP02_01:0.6666772404055226):0.07712626187053306[65]):0.030346433785689797[29],((((((((((((Ga0172377_10046131:0.0508006698391692,Ga0172377_10059128:0.054948270119849596):0.01874666083350629[55],Bacteria_Proteobacteria_deltaepsilon_subdivisions_Epsilonproteobacteria_Campylobacterales_Helicobacteraceae_Sulfurimonas_denitrificans_DSM_1251:0.05990779096702514):0.030530366181702462[77],Ga0172377_10000001:0.1460606816707184):0.08730901729509677[100],('Bacteria_Proteobacteria_deltaepsilon_subdivisions_Epsilonproteobacteria_Campylobacterales_Helicobacteraceae_Sulfuricurvum_sp._RIFRC_1':0.021893576493333455,Ga0172377_10005410:0.0198079306776342):0.10599641702214146[100]):0.09975224993554122[100],'Bacteria_Proteobacteria_deltaepsilon_subdivisions_Epsilonproteobacteria_Campylobacterales_Helicobacteraceae_Thiovulum_sp._ES':0.2608759844092896):0.05961950482000544[100],((((Ga0172382_10001944:0.007535806755022634,Ga0172378_10036470:0.02130059054401645):0.054664153048313846[100],Bacteria_Proteobacteria_deltaepsilon_subdivisions_Epsilonproteobacteria_Campylobacterales_Campylobacteraceae_Arcobacter_nitrofigilis_DSM_7299:0.1122412877045762):0.028363211588648074[78],Ga0172377_10019603:0.06939291842297424):0.0602244568297472[100],Ga0172377_10000018:0.1527115612942911):0.1170419929843951[100]):0.02914637085083882[36],(((Ga0172382_10029582:0.000001,Ga0172377_10019011:0.000001):0.152619937770877[100],Bacteria_Proteobacteria_deltaepsilon_subdivisions_Epsilonproteobacteria_Campylobacterales_Campylobacteraceae_Campylobacter_jejuni_jejuni_NCTC_11168:0.1703333441728634):0.03345826173696764[87],Bacteria_Proteobacteria_deltaepsilon_subdivisions_Epsilonproteobacteria_Campylobacterales_Campylobacteraceae_Sulfurospirillum_barnesii_SES_3:0.16459497624367225):0.06768999484127702[100]):0.025182782609951992[37],(Bacteria_Proteobacteria_deltaepsilon_subdivisions_Epsilonproteobacteria_Campylobacterales_Helicobacteraceae_Wolinella_succinogenes_DSM_1740:0.1008604910753168,Bacteria_Proteobacteria_deltaepsilon_subdivisions_Epsilonproteobacteria_Campylobacterales_Helicobacteraceae_Helicobacter_cetorum_MIT_99_5656:0.25381678489210735):0.1332614414194797[100]):0.02580620660821875[36],(((Ga0172377_10007996:0.000001,Ga0172378_10029462:0.000001):0.07456526694426646[100],'Bacteria_Proteobacteria_deltaepsilon_subdivisions_Epsilonproteobacteria_unclassified_Epsilonproteobacteria_Sulfurovum_sp._NBC37_1':0.06686492947929601):0.05578913067523272[100],Ga0172382_10017176:0.1028162169166138):0.1552729411445002[100]):0.053360154305096774[95],'Bacteria_Proteobacteria_deltaepsilon_subdivisions_Epsilonproteobacteria_unclassified_Epsilonproteobacteria_Nitratiruptor_sp._SB155_2':0.12808480708451064):0.07571719696800461[96],(('Bacteria_Proteobacteria_deltaepsilon_subdivisions_Epsilonproteobacteria_Nautiliales_Nautiliaceae_Lebetimonas_sp._JS032':0.05803898929590012,Bacteria_Proteobacteria_deltaepsilon_subdivisions_Epsilonproteobacteria_Nautiliales_Nautiliaceae_Nautilia_profundicola_AmH:0.04309544348236782):0.018727260671458357[85],Bacteria_Proteobacteria_deltaepsilon_subdivisions_Epsilonproteobacteria_Nautiliales_Nautiliaceae_Caminibacter_mediatlanticus_TB_2_unfinished_sequence:0.035339728498300804):0.20932621407409968[100]):0.4832664620031095[100],(Bacteria_Proteobacteria_deltaepsilon_subdivisions_Deltaproteobacteria_Desulfurellales_Desulfurellaceae_Desulfurella_acetivorans_A63_DSM_5264:0.2982118304219483,Bacteria_Proteobacteria_deltaepsilon_subdivisions_Deltaproteobacteria_Desulfurellales_Desulfurellaceae_Hippea_maritima_MH2_DSM_10411:0.24341677361966685):0.27203601761500584[100]):0.1121625154386523[85]):0.031103474207370585[8],(((((((Bacteria_Deferribacteres_Deferribacteres_Deferribacterales_Selenovibrio_woodruffii_S4:0.12845030191806428,Bacteria_Deferribacteres_Deferribacteres_Deferribacterales_Deferribacteraceae_Denitrovibrio_acetiphilus_DSM_12809:0.0866643652718948):0.10939290022668491[100],Bacteria_Deferribacteres_Deferribacteres_Deferribacterales_Deferribacteraceae_Geovibrio_thiophilus_L21_Ace_BES:0.1796469315266518):0.05920977743332978[100],Bacteria_Deferribacteres_Deferribacteres_Deferribacterales_Deferribacteraceae_Mucispirillum_schaedleri_ASF457:0.2962420505458354):0.04509426892796675[99],(Bacteria_Deferribacteres_Deferribacteres_Deferribacterales_Deferribacteraceae_Flexistipes_sinusarabici_MAS10_DSM_4947:0.2633316697504813,Bacteria_Deferribacteres_Deferribacteres_Deferribacterales_Deferribacteraceae_Calditerrivibrio_nitroreducens_DSM_19672:0.21138730043642484):0.04888760239331891[73]):0.03858287883354805[53],Bacteria_Deferribacteres_Deferribacteres_Deferribacterales_Deferribacteraceae_Deferribacter_desulfuricans_SSM1:0.14635926861871873):0.24587523253068833[100],(Bacteria_Chrysiogenetes_Chrysiogenetes_Chrysiogenales_Chrysiogenaceae_Desulfurispirillum_indicum_S5:0.13842231509991887,Bacteria_Chrysiogenetes_Chrysiogenetes_Chrysiogenales_Chrysiogenaceae_Chrysiogenes_arsenatis_DSM_11915:0.1799332147419057):0.296494671728992[100]):0.0532667772916664[98],Ga0172379_10026750:0.6476046678659495):0.04189547467327648[26]):0.039486591132785076[0]):0.033186303286733665[1],((((((((((((Bacteria_FibrobacteresAcidobacteria_group_Acidobacteria_Acidobacteriia_Acidobacteriales_Acidobacteriaceae_Edaphobacter_aggregans_DSM_19364:0.08687847519175129,'Bacteria_FibrobacteresAcidobacteria_group_Acidobacteria_Acidobacteriia_Acidobacteriales_Acidobacteriaceae_Acidobacterium_sp._MP5ACTX8':0.09361956876247435):0.032821946499445254[94],Bacteria_FibrobacteresAcidobacteria_group_Acidobacteria_Acidobacteriia_Acidobacteriales_Acidobacteriaceae_Terriglobus_saanensis_SP1PR4:0.13110800416648383):0.05846523469300413[100],Bacteria_FibrobacteresAcidobacteria_group_Acidobacteria_Acidobacteriia_Acidobacteriales_Acidobacteriaceae_Acidobacterium_capsulatum_ATCC_51196:0.1007855798784214):0.09358664863873178[100],Bacteria_FibrobacteresAcidobacteria_group_Acidobacteria_Acidobacteriia_Acidobacteriales_Acidobacteriaceae_Candidatus_Koribacter_versatilis_Ellin345:0.17650060110444832):0.15630886817394574[100],(Bacteria_FibrobacteresAcidobacteria_group_Acidobacteria_Solibacteres_Solibacterales_Solibacteraceae_Solibacter_usitatus_Ellin6076:0.20003545916593657,Bacteria_FibrobacteresAcidobacteria_group_Acidobacteria_unclassified_Acidobacteria_Bryobacter_aggregatus_DSM_18758:0.25551207424904643):0.11772302342475705[100]):0.051092791422981954[92],((((Bacteria_Acidobacteria_RIFCSPLOWO2_02_FULL_Acidobacteria_61_28:0.12624851078793498,Bacteria_Acidobacteria_RIFCSPLOWO2_12_FULL_Acidobacteria_60_22:0.10163490599842495):0.04784762808996934[96],(Bacteria_Acidobacteria_RIFCSPLOWO2_12_FULL_Acidobacteria_59_11:0.08985277389814961,Bacteria_Acidobacteria_RIFCSPLOWO2_12_FULL_Acidobacteria_54_10:0.1916933117253916):0.057012397619683686[100]):0.026563312212940726[64],Bacteria_Acidobacteria_RIFCSPLOWO2_02_FULL_Acidobacteria_60_20:0.1893840155990314):0.04296989923758954[94],Bacteria_Acidobacteria_RIFCSPLOWO2_02_FULL_Acidobacteria_59_13:0.17259051023216054):0.1451744395992569[100]):0.06849379231011099[85],Bacteria_Acidobacteria_RIFCSPHIGHO2_01_FULL_Acidobacteria_67_28:0.34756916606472643):0.09706755285735769[100],((((((Bacteria_Acidobacteria_RIFCSPLOWO2_12_FULL_Acidobacteria_67_14b:0.08356874446027795,Bacteria_Acidobacteria_RIFCSPLOWO2_12_FULL_Acidobacteria_65_11:0.08466554018200423):0.02134943746938278[51],Bacteria_Acidobacteria_RIFCSPLOWO2_02_FULL_Acidobacteria_65_29:0.09253010240532511):0.09234497067325131[100],(Bacteria_Acidobacteria_RBG_16_Acidobacteria_68_9:0.15439022017646176,Bacteria_Acidobacteria_RIFCSPLOWO2_12_FULL_Acidobacteria_66_21:0.12048493756945255):0.04615412299273336[85]):0.040185078841352695[53],(Bacteria_Acidobacteria_RIFCSPLOWO2_02_FULL_Acidobacteria_68_18:0.05418310137860116,Bacteria_Acidobacteria_RIFCSPLOWO2_02_FULL_Acidobacteria_67_21:0.06518890003986177):0.08117754195943139[100]):0.0445503927489348[42],Bacteria_Acidobacteria_RIFCSPLOWO2_12_FULL_Acidobacteria_66_10:0.17900682914368415):0.19775159773202988[100],Bacteria_Acidobacteria_RBG_16_Acidobacteria_70_10:0.326345209321496):0.10191557057349288[100]):0.048613416060562464[72],(((Bacteria_Acidobacteria_RBG_13_Acidobacteria_68_16:0.1875070652031745,Bacteria_FibrobacteresAcidobacteria_group_Acidobacteria_unclassified_Acidobacteria_Thermoanaerobaculum_aquaticum_MP_01:0.11982270673292694):0.2235370488149302[100],Bacteria_Acidobacteria_RBG_16_Acidobacteria_64_8:0.3748769516392554):0.10143350626247072[99],(Bacteria_FibrobacteresAcidobacteria_group_Acidobacteria_Holophagae_Holophagales_Holophagaceae_Holophaga_foetida_TMBS4_DSM_6591:0.10247537022231068,Bacteria_FibrobacteresAcidobacteria_group_Acidobacteria_Holophagae_Holophagales_Holophagaceae_Geothrix_fermentans_DSM_14018:0.07299236846115287):0.46245148631930766[100]):0.05229780642134241[65]):0.05373057000748105[99],(((((((Bacteria_Aminicenantes_OP8_RBG_19FT_COMBO_Aminicenantes_58_17:0.02726429263307173,Bacteria_Aminicenantes_OP8_RBG_19FT_COMBO_Aminicenantes_59_29:0.035135490108026524):0.07673548277005127[100],Bacteria_Aminicenantes_OP8_RBG_16_Aminicenantes_63_16:0.13371063871442868):0.04510650977763797[99],Bacteria_Aminicenantes_OP8_RBG_13_Aminicenantes_59_9:0.14135691876423273):0.04477854163141304[100],(Bacteria_Aminicenantes_OP8_RBG_13_Aminicenantes_63_10:0.18519554860116516,Bacteria_Aminicenantes_OP8_RBG_13_Aminicenantes_62_12:0.20585356852448866):0.060952653441046944[98]):0.03495972510920753[63],((Bacteria_Aminicenantes_OP8_RBG_13_Aminicenantes_64_14:0.07833928960247816,Bacteria_Aminicenantes_OP8_RBG_19FT_COMBO_Aminicenantes_65_30:0.03092604692745171):0.020078018634539507[82],Bacteria_Aminicenantes_OP8_RBG_16_Aminicenantes_66_30:0.05699703253904698):0.20462269313069426[100]):0.05417235803324516[98],(((Bacteria_CP_OP8_candidate_division_OP8_bacterium_SCGC_AAA252_O11_SAK_001_85:0.0011020287587721889,Bacteria_CP_OP8_candidate_division_OP8_bacterium_SCGC_AAA252_F08_Combined_Assembly_OP8_2__OP8:0.005231747373827478):0.0021494161508841536[69],Bacteria_CP_OP8_candidate_division_OP8_bacterium_SCGC_AAA252_A02_Combined_Assembly_OP8_1__OP8:0.001867111119087106):0.1576479363368457[100],(Bacteria_CP_OP8_candidate_division_OP8_bacterium_SCGC_AAA255_E10_SAK_001_126:0.01448251457677463,Bacteria_CP_OP8_candidate_division_OP8_bacterium_SCGC_AAA252_K06_SAK_001_60:0.00936597785164972):0.20930177556588747[100]):0.09012953898116871[100]):0.32041925114943126[100],Bacteria_OP8X_RBG_13_OP8X_37_8:0.5624593484464673):0.0773108442364272[100]):0.09065160564811814[99],(((((((Bacteria_CP_Rokubacteria_13_1_20CM_4_Rokubacteria_70_14:0.013120052902956036,Bacteria_CP_Rokubacteria_13_1_40CM_4_Rokubacteria_69_39:0.000001):0.04296161785870911[100],(Bacteria_CP_Rokubacteria_13_2_20CM_2_Rokubacteria_70_11:0.05100477197231568,Bacteria_CP_Rokubacteria_13_2_20CM_Rokubacteria19cls_69_15:0.04256596154797032):0.011897462239168988[78]):0.06134419810234393[100],(Bacteria_Rokubacteria_RIFCSPLOWO2_12_FULL_Rokubacteria_71_22:0.052684476349360576,Bacteria_Rokubacteria_GWA2_Rokubacteria_73_35:0.034641821409281626):0.059602985541867604[100]):0.024901255691127666[99],(Bacteria_CP_Rokubacteria_13_1_40CM_Rokubacteria_69_27:0.08862531293948717,Bacteria_CP_Rokubacteria_13_2_20CM_2_Rokubacteria_64_8:0.11780765574957996):0.03798350928411809[100]):0.043747963322938066[100],((Bacteria_Rokubacteria_GWC2_Rokubacteria_70_16:0.03510720422080427,Bacteria_Rokubacteria_GWA2_Rokubacteria_70_23:0.0310676228668747):0.1394463317392729[100],(Bacteria_Rokubacteria_RIFCSPLOWO2_02_FULL_Rokubacteria_68_19:0.012802708536621576,Bacteria_CP_Rokubacteria_CSP1_6:0.0065603722621681015):0.1748579336378122[100]):0.054480965900053135[100]):0.15068452166140434[100],Bacteria_Rokubacteria_RIFCSPLOWO2_02_FULL_Rokubacteria_71_18:0.248116339864632):0.23822701913505417[100],(((Bacteria_NC10_RIFCSPLOWO2_02_FULL_NC10_66_22:0.07102655798832869,Bacteria_NC10_RBG_16_NC10_65_8:0.11136420686031245):0.16651813964866502[100],Bacteria_CP_NC10_Candidatus_Methylomirabilis_oxyfera:0.30815641358821955):0.05397509260389599[76],Bacteria_CP_NC10_CSP1_5:0.29394950162090083):0.15220479694237765[100]):0.06921565846424693[71]):0.050292385312836085[27],((((((((((((Bacteria_Nitrospirae_RBG_13_Nitrospirae_41_22:0.1006330338963548,Bacteria_Nitrospirae_RBG_19FT_COMBO_Thermodesulfovibrio_42_12:0.11489823488049078):0.018345325490606967[54],(Bacteria_Nitrospirae_CG_Nitrosp_02:0.04486095131527312,Bacteria_Nitrospirae_RBG_19FT_COMBO_Thermodesulfovibrio_41_18:0.03833448327593025):0.048374649204951936[100]):0.022110508842596133[61],Bacteria_Nitrospirae_RBG_13_Nitrospirae_39_12:0.13741808939839562):0.02740719984017348[97],Bacteria_Nitrospira_uncultured_SMTZ_35:0.17936923317159037):0.037183652987688554[97],Bacteria_Nitrospirae_RBG_13_Nitrospirae_43_8:0.2064852225764131):0.0689342222603555[100],(((Bacteria_Nitrospirae_RBG_16_Nitrospirae_43_8:0.02641940554237765,Bacteria_Nitrospirae_GWF2_Nitrospirae_44_13:0.025445277587341053):0.036613746191781704[100],Bacteria_Nitrospirae_CG_Nitrosp_01:0.053389179553933186):0.14983988738561393[100],Bacteria_Nitrospirae_BJP_IG2158_Nitrospirae_45_240:0.2568546635025801):0.033306094782060924[60]):0.05099718961931332[96],((Bacteria_Nitrospirae_GWB2_Nitrospirae_47_37:0.19899288792865422,Bacteria_Nitrospirae_Nitrospira_Nitrospirales_Nitrospiraceae_Thermodesulfovibrio_yellowstonii_DSM_11347:0.29749576900398855):0.10345160436356915[100],'Bacteria_Nitrospirae_Nitrospira_Nitrospirales_Nitrospiraceae_Candidatus_Magnetobacterium_sp._MYR_1':0.3407151539970452):0.031193746346230178[91]):0.06803986276052276[100],((Bacteria_Nitrospira_uncultured_SG8_35_1:0.1380124498305788,Bacteria_Nitrospira_uncultured_SG8_35_4:0.1372662185471829):0.13571434821926198[100],Bacteria_Nitrospirae_RIFCSPHIGHO2_02_FULL_Nitrospirae_40_19:0.2210202956074956):0.0688153185741407[100]):0.13770496127026854[100],(((((((Bacteria_Nitrospirae_Nitrospira_Candidatus_Nitrospira_defluvii:0.12593826666884222,Bacteria_Nitrospirae_RBG_19FT_COMBO_Nitrospirae_58_9:0.16507017992277806):0.08099615631278834[100],Bacteria_Nitrospirae_RIFCSPLOWO2_02_FULL_Nitrospirae_62_14:0.178202928861956):0.24287637831783337[100],Bacteria_Nitrospirae_RIFCSPHIGHO2_01_FULL_Nitrospirae_66_17:0.3628217037480912):0.06607256672884088[94],Bacteria_Nitrospirae_Nitrospira_Nitrospirales_Nitrospiraceae_Leptospirillum_ferriphilum_YSK:0.5895708059835187):0.05427614003543724[81],(Bacteria_Nitrospirae_RBG_16_Nitrospirae_43_11:0.1971328773954384,Bacteria_Nitrospirae_RifCSPlowO2_12_Nitrospirae_42_9:0.18679891199278575):0.23299341244686245[100]):0.040887160186201754[74],Bacteria_Nitrospirae_RBG_19FT_COMBO_Nitrospirae_42_15:0.32245386314223223):0.044564760458353[78],((((Bacteria_Nitrospirae_GWC2_Nitrospirae_57_9:0.09492446648452857,Bacteria_Nitrospirae_GWC2_Nitrospirae_56_14:0.09565134987220292):0.0366446140075225[100],Bacteria_Nitrospirae_RBG_19FT_COMBO_Nitrospirae_55_12:0.10490477733973558):0.03955589841780549[96],Bacteria_Nitrospirae_GWC2_Nitrospirae_57_13:0.1363087430609946):0.19633550073013906[100],Bacteria_Nitrospirae_RBG_16_Nitrospirae_64_22:0.39897400870499):0.0640859836426575[83]):0.03779191900268586[64]):0.06039267261832526[93],(Bacteria_Rokubacteria_Deltaproteobacteria_CG_Delta_03:0.5281886122610748,Bacteria_Rokubacteria_Deltaproteobacteria_CG_Delta_01:0.3428945741917766):0.1122166183015092[94]):0.044729604426535285[65],((((((('Bacteria_Nitrospinae_Nitrospinia_Nitrospinales_Nitrospinaceae_Nitrospina_sp._SCGC_AAA288_L16':0.01947190019268419,'Bacteria_Nitrospinae_Nitrospinia_Nitrospinales_Nitrospinaceae_Nitrospina_sp._AB_629_B18':0.04828404030459943):0.030562989118923856[100],'Bacteria_Nitrospinae_Nitrospinia_Nitrospinales_Nitrospinaceae_Nitrospina_sp._AB_629_B06':0.06694121941236642):0.2117640982759479[100],Bacteria_Nitrospirae_RIFCSPLOWO2_12_FULL_Nitrospinae_47_7:0.22080401458487842):0.23233463933478626[100],(Bacteria_Nitrospinae_RIFCSPLOWO2_01_FULL_Nitrospinae_39_10:0.13475579988747421,Bacteria_Nitrospinae_RifCSPlowO2_12_Nitrospinae_39_15:0.1496417117139437):0.2153232143415429[100]):0.06230748037303924[96],Bacteria_Nitrospinae_RIFCSPLOWO2_12_FULL_Nitrospinae_45_22:0.3904025755332299):0.05009891220953255[87],(('Bacteria_CP_Tectomicrobia_Candidatus_Entotheonella_sp._TSY2':0.019282434016439254,'Bacteria_CP_Tectomicrobia_Candidatus_Entotheonella_sp._TSY1':0.02766182943657647):0.342104937047397[100],Bacteria_Entotheonella_RIFCSPLOWO2_12_FULL_Entotheonella_69_37:0.5274935814991499):0.09630503659141887[96]):0.057699486438840264[93],(((Bacteria_RIF3_GWA2_RIF03_38_11:0.0033746617732615114,Bacteria_RIF3_RBG_16_RIF03_38_11:0.001046273909361961):0.1495444562820989[100],(Bacteria_RIF03_RBG_16_RIF03_38_10:0.05245536997330058,Bacteria_RIF3_RIFCSPLOWO2_12_FULL_RIF03_38_15:0.05510079922121447):0.10324855860596127[100]):0.32198571787402974[100],Bacteria_RIF3_RBG_13_RIF03_48_7:0.5061484491643156):0.0710025075024765[38]):0.030659652883590294[35]):0.02520949102535619[14],(Bacteria_Modulibacteria_KSB3_bacterium_UASB270:0.12789006056260988,Bacteria_Modulibacteria_KSB3_bacterium_UASB14:0.12836216999908023):0.36343754264811157[100]):0.023738263742966836[6]):0.03581263589973771[35]):0.04305564461949318[2]):0.03283931075743318[1],(((((((((((((Bacteria_ChlamydiaeVerrucomicrobia_group_Lentisphaerae_Lentisphaeria_Victivallales_Victivallaceae_Victivallis_vadensis_ATCC_BAA_548:0.1608777992098811,Ga0172378_10005489:0.21629040578688796):0.05660408602882949[99],Ga0172377_10036462:0.1973671335203666):0.02752337774356839[30],(Ga0172380_10003548:0.13664456062982433,Ga0172379_10001103:0.16781833625898468):0.03898955391939518[71]):0.02733516888809584[38],Ga0172377_10000796:0.2373861417641301):0.07601368172530787[100],Bacteria_Lentisphaerae_GWF2_Lentisphaerae_45_14:0.28381331748792116):0.03378084289457517[48],(Bacteria_Lentisphaerae_GWF2_Lentisphaerae_44_16:0.23937344741973154,Bacteria_Lentisphaerae_GWF2_Lentisphaerae_52_8:0.20714937390227828):0.039692182477376825[51]):0.06376396016289032[100],(((Bacteria_Lentisphaerae_GWF2_Lentisphaerae_49_21:0.07710708202697303,Bacteria_Lentisphaerae_GWF2_Lentisphaerae_50_93:0.058250772242046):0.05494329260612263[100],Ga0172379_10007089:0.09120245276611438):0.21713955736194546[100],Bacteria_Lentisphaerae_GWF2_Lentisphaerae_38_69:0.3624846966535382):0.0411369076065875[47]):0.1632728880846357[100],(((((Ga0172378_10018642:0.09298958843531846,Ga0172377_10005810:0.17841011164934262):0.06567572859821746[100],(Ga0172378_10011956:0.000679,Ga0172377_10006708:0.000001):0.1572675532005725[100]):0.0452013298163596[94],(Ga0172377_10007324:0.003753715004934932,Ga0172378_10021932:0.000001):0.15747928638461106[100]):0.17094601131845666[100],Bacteria_Lentisphaerae_RIFOXYB12_FULL_Lentisphaerae_65_16:0.27220999659359757):0.12133544993946499[100],Bacteria_ChlamydiaeVerrucomicrobia_group_Lentisphaerae_Lentisphaerales_Lentisphaeraceae_Lentisphaera_araneosa_HTCC2155_unfinished_sequence:0.4523383064925932):0.05262670976780415[47]):0.15137116126298222[100],(((((((((Ga0172377_10002856:0.000001,Ga0172378_10043736:0.0019395976944514004):0.05962867528127491[100],Ga0172378_10047947:0.05282663904210727):0.020230658792032674[56],Ga0172377_10012290:0.10251904598421202):0.026218004501096015[47],Ga0172378_10018232:0.13808682155069985):0.05303761357182024[98],Ga0172377_10017500:0.1933301733494166):0.17407903279405357[100],Ga0172382_10008477:0.34328957459280884):0.12897487720483314[100],(Bacteria_Lentisphaerae_RIFOXYC12_FULL_Lentisphaerae_60_16:0.3911613538374952,Bacteria_Lentisphaerae_RifOxyA12_full_Lentisphaerae_48_11:0.3180064748518383):0.04876402903176702[72]):0.0718556185529784[100],(Ga0172380_10000303:0.04265682177883878,Ga0172380_10031525:0.044202180935294155):0.38461230259106394[100]):0.06370390243603508[100],(((Ga0172380_10009289:0.05177529884783816,Ga0172380_10022072:0.07023542085540324):0.000001[82],Ga0172380_10039565:0.04478923208756669):0.3413097716147111[96],Bacteria_Lentisphaerae_GWF2_Lentisphaerae_57_35:0.27562083800460435):0.061386019035194916[90]):0.1476078644105896[100]):0.03985957079885871[26],(((((((Bacteria_Verrucomicrobia_Opitutae_GWC2_Verrucomicrobia_42_7:0.26534420110161916,Bacteria_Verrucomicrobia_Opitutae_GWF2_Verrucomicrobia_51_19:0.25278192599518157):0.0906683462921909[100],(Ga0172378_10028750:0.06847173560401076,Ga0172381_10026661:0.09286757769997056):0.32636663370543006[100]):0.027180863551857115[28],Bacteria_Verrucomicrobia_CG_Verruco_01:0.5184723698339906):0.06753658297411702[63],Bacteria_ChlamydiaeVerrucomicrobia_group_Verrucomicrobia_Opitutae_Puniceicoccales_Puniceicoccaceae_Coraliomargarita_akajimensis_DSM_45221:0.30164144561194517):0.08279767770902247[99],((('Bacteria_ChlamydiaeVerrucomicrobia_group_Verrucomicrobia_Opitutae_Opitutales_Opitutaceae_Opitutaceae_sp._TAV5':0.04666031104557167,'Bacteria_ChlamydiaeVerrucomicrobia_group_Verrucomicrobia_Opitutae_Opitutales_Opitutaceae_Opitutaceae_sp._TAV2':0.05188950877735499):0.12519699236366666[100],Bacteria_ChlamydiaeVerrucomicrobia_group_Verrucomicrobia_Opitutae_Opitutales_Opitutaceae_Opitutus_terrae_PB90_1:0.13265732082834125):0.05163126810646945[84],Bacteria_Verrucomicrobia_Opitutae_RIFCSPLOWO2_12_FULL_Verrucomicrobia_64_8:0.1630644605637115):0.1853430041401749[100]):0.3100052696906177[100],(((((Bacteria_ChlamydiaeVerrucomicrobia_group_Verrucomicrobia_Verrucomicrobiae_Verrucomicrobiales_Verrucomicrobiaceae_Rubritalea_marina_DSM_17716_SAORIC_165:0.07375477238287065,Bacteria_ChlamydiaeVerrucomicrobia_group_Verrucomicrobia_Verrucomicrobiae_Verrucomicrobiales_Verrucomicrobiaceae_Rubritalea_marina_DSM_17716:0.026651790396779784):0.19076296117348202[100],Bacteria_ChlamydiaeVerrucomicrobia_group_Verrucomicrobia_Verrucomicrobiae_Verrucomicrobiales_Verrucomicrobiaceae_Akkermansia_muciniphila_ATCC_BAA_835:0.21873554131987127):0.1444914437776208[100],Bacteria_ChlamydiaeVerrucomicrobia_group_Verrucomicrobia_Verrucomicrobiae_Verrucomicrobiales_Verrucomicrobiaceae_Verrucomicrobium_spinosum_DSM_4136_unfinished_sequence:0.2406490382828661):0.13176472189270383[100],(((Ga0172380_10001127:0.023713084371614013,Ga0172380_10001568:0.024821597932522366):0.16427176962181012[100],Bacteria_Verrucomicrobia_Verrucomicrobiae_RIFCSPHIGHO2_12_FULL_Verrucumicrobia_41_10:0.2513823046378847):0.08937889281395561[100],Bacteria_ChlamydiaeVerrucomicrobia_group_Verrucomicrobia_Spartobacteria_Chthoniobacter_flavus_Ellin428_unfinished_sequence:0.20055994305810998):0.10544209150015593[100]):0.10887545185269287[99],Bacteria_ChlamydiaeVerrucomicrobia_group_Verrucomicrobia_unclassified_Verrucomicrobia_Methylacidiphilales_Methylacidiphilaceae_Methylacidiphilum_infernorum_V4:0.5969039116177886):0.059859498948303[89]):0.051740891405560596[77],(((Ga0172379_10003005:0.2014897775598019,Bacteria_ChlamydiaeVerrucomicrobia_group_Verrucomicrobia_Verrucomicrobiae_Verrucomicrobiales_Verrucomicrobia_subdivision_3_bacterium_Ellin514:0.17360538393086689):0.11284097726315068[100],Ga0172382_10014232:0.3899316739085199):0.14380830293741198[100],(Ga0172379_10000322:0.012977354291150434,Bacteria_Verrucomicrobia_Verrucomicrobiae_GWF2_Verrucomicrobia_62_7:0.01569584326669471):0.42716675428744466[100]):0.07439276837842401[99]):0.08046365818527068[99]):0.08886724722916384[100],(((((((Ga0172379_10033493:0.25357905068311704,Bacteria_Chlamydiae_Chlamydiia_RIFCSPHIGHO2_12_FULL_Chlamydiae_27_8:0.2433118315990419):0.10598701624069617[98],Bacteria_Chlymidae_uncultured_SMTZ_39:0.40956000306836415):0.10415442906671935[100],(Bacteria_Chlamydiae_Chlamydiia_RIFCSPLOWO2_02_FULL_Chlamydiae_45_22:0.19686934084681162,Bacteria_Chlamydiae_Chlamydiia_RIFCSPHIGHO2_12_FULL_Chlamydiae_49_9:0.14635164617392116):0.19064323773925196[100]):0.0450537279108425[82],Bacteria_Chlamydiae_Chlamydiia_Chlamydiales_RIFCSPLOWO2_02_FULL_Chlamydiae_49_12:0.3652172236558089):0.04155161337088398[37],'Bacteria_ChlamydiaeVerrucomicrobia_group_Chlamydiae_Chlamydiia_Chlamydiales_Simkaniaceae_Simkania_negevensis_Z_gsn.131':0.32989847722440535):0.04887898488594544[88],(((('Bacteria_ChlamydiaeVerrucomicrobia_group_Chlamydiae_Chlamydiia_Chlamydiales_Parachlamydiaceae_Neochlamydia_sp._S13':0.18997107092627719,Bacteria_ChlamydiaeVerrucomicrobia_group_Chlamydiae_Chlamydiia_Chlamydiales_Parachlamydiaceae_Parachlamydia_acanthamoebae_UV7:0.23560003013672048):0.035281473484379156[53],Bacteria_ChlamydiaeVerrucomicrobia_group_Chlamydiae_Chlamydiia_Chlamydiales_Parachlamydiaceae_Candidatus_Protochlamydia_amoebophila_UWE25:0.18583341959291344):0.047408062108587945[59],Bacteria_ChlamydiaeVerrucomicrobia_group_Chlamydiae_Chlamydiia_Chlamydiales_Waddliaceae_Waddlia_chondrophila_WSU_86_1044:0.2541250922104541):0.0999548309203302[100],(Bacteria_ChlamydiaeVerrucomicrobia_group_Chlamydiae_Chlamydiia_Chlamydiales_Chlamydiaceae_ChlamydiaChlamydophila_group_Chlamydophila_pneumoniae_AR39:0.08331237163308591,Bacteria_ChlamydiaeVerrucomicrobia_group_Chlamydiae_Chlamydiia_Chlamydiales_Chlamydiaceae_ChlamydiaChlamydophila_group_Chlamydophila_abortus_S263:0.086578715682192):0.4284042574505489[100]):0.046027167890699605[65]):0.14584479909941406[100],Bacteria_Chlamydiae_RIFCSPHIGHO2_12_FULL_Chlamydiae_49_11:0.5922162347406248):0.31837216317501005[100]):0.059796841682574264[86],(((((((((((Bacteria_Plantomycetes_Phycisphaerae_uncultured_SMTZ1_79:0.09230342186277918,Bacteria_Planctomycetes_Phycisphaerae_RBG_16_Planctomycetes_55_9:0.12053437720863869):0.0272518590540054[97],Bacteria_Plantomycetes_Phycisphaerae_uncultured_SG8_4:0.08936036494759403):0.088423339900086[100],(Ga0172381_10024386:0.16824864283740348,Ga0172382_10010858:0.16809901181268172):0.03477798213281158[78]):0.07160801668944128[99],Ga0172379_10000226:0.3217828858555434):0.14032021413409268[100],Bacteria_Plantomycetes_Phycisphaerae_uncultured_SMTZ_30:0.3044811455267782):0.13354643706938374[100],((Bacteria_Plantomycetes_Phycisphaerae_uncultured_SMTZ_33:0.26868955448467524,Bacteria_Planctomycetes_Phycisphaerae_uncultured_SMTZ_32_1:0.23816113913526538):0.14276529377564096[100],Bacteria_Planctomycetes_Phycisphaerae_Phycisphaerales_Phycisphaeraceae_Phycisphaera_mikurensis_NBRC_102666:0.665528967065709):0.05152035877965977[71]):0.10604243179356265[99],Bacteria_Planctomyce_uncultured_SMTZ_25:0.47470057540461497):0.07066591349015816[100],((((Bacteria_Planctomycetes_Planctomycetia_Planctomycetales_Planctomycetaceae_Blastopirellula_marina_SH_106T_DSM_3645:0.2540133865041705,Bacteria_Planctomycetes_Planctomycetia_Planctomycetales_Planctomycetaceae_Rhodopirellula_baltica_SH_1:0.2969275455056102):0.05420098672086793[49],Bacteria_Planctomycetes_Planctomycetia_Planctomycetales_Planctomycetaceae_Pirellula_staleyi_DSM_6068:0.23222239009133228):0.16015863104127437[100],(Bacteria_Planctomycetes_Planctomycetia_Planctomycetales_Planctomycetaceae_Planctomyces_limnophilus_DSM_3776:0.20232165214890685,Bacteria_Planctomycetes_Planctomycetia_Planctomycetales_Planctomycetaceae_Schlesneria_paludicola_DSM_18645:0.3329125904116266):0.21285342364509896[100]):0.056767091518889146[75],((Bacteria_Planctomycetes_Planctomycetia_Planctomycetales_Planctomycetaceae_Isosphaera_pallida_ATCC_43644:0.20680120809717772,Bacteria_Planctomycetes_Planctomycetia_Planctomycetales_Planctomycetaceae_Singulisphaera_acidiphila_MOB10_DSM_18658:0.13229466855104155):0.19870477435211376[100],Bacteria_Planctomycetes_Planctomycetia_Planctomycetales_Planctomycetaceae_Zavarzinella_formosa_DSM_19928:0.4852965110774119):0.05535375258910058[83]):0.1560591223855199[100]):0.0563495728397343[97],(Bacteria_Plantomycetes_uncultured_DG_58:0.45190958116402546,Bacteria_Plantomycetes_uncultured_DG_23:0.39598611768249636):0.08034450987925412[94]):0.05763073775086314[83],((Bacteria_Planctomycetes_Planctomycetia_Candidatus_Brocadiales_Candidatus_Brocadiaceae_Candidatus_Kuenenia_stuttgartiensis_RU1:0.24380993994587863,Bacteria_Planctomycetes_Brocadiales_GWB2_Planctomycetes_41_19:0.17251337176832449):0.264015255147497[100],Bacteria_Planctomyce_uncultured_SMTZ_32:0.5681377658639883):0.04158447438423796[54]):0.0674977857615735[97],Ga0172380_10024011:0.9240452173128997):0.14053156603157202[99]):0.03940359480730038[79],((((((((((((((((Ga0172381_10025194:0.0015974228575061922,Ga0172380_10006579:0.000001):0.12405370877504796[100],Ga0172381_10000005:0.09601071944876738):0.036464548826816934[92],(Ga0172381_10017845:0.0028079656775248374,Ga0172380_10005575:0.004661309512636436):0.07312967296686823[100]):0.07295332172006574[100],Ga0172380_10025001:0.18073917571357478):0.10521429629707901[100],(((Ga0172380_10002325:0.000001,Ga0172381_10014357:0.001442408476060919):0.04264314881439679[100],Ga0172380_10008690:0.05611658879901116):0.09058933424878424[100],(Ga0172381_10041022:0.12427814874174636,Ga0172381_10007720:0.09866951860299666):0.05462806354521277[100]):0.051885917434199236[100]):0.047936533997182895[100],(Bacteria_WOR_2_RBG_13_WOR_2_44_8b:0.1905920117404336,Bacteria_WOR_2_RIFCSPLOWO2_12_FULL_WOR_2_51_8:0.21182220857993395):0.0406536750181794[89]):0.03678451036233099[90],(Bacteria_WOR_2_RBG_13_WOR_2_41_10:0.20036535096925423,Bacteria_WOR_2_RIFCSPLOWO2_01_FULL_WOR_2_41_12:0.19100102385025153):0.04012215790425344[90]):0.0440300769113815[87],(Ga0172381_10018456:0.35901472910664856,Ga0172381_10002057:0.25952904306582214):0.04546532138015991[44]):0.15474768348948453[100],(((Bacteria_WOR_2_RIFCSPHIGHO2_02_FULL_WOR_2_45_21:0.0468825927025307,Bacteria_WOR_2_RIFCSPHIGHO2_02_FULL_WOR_2_46_37:0.04614197779046281):0.1434647102042974[100],Bacteria_WOR_2_GWF2_WOR_2_43_52:0.2138030731286653):0.04894770505138091[85],(Bacteria_WOR_2_GWB2_WOR_2_45_9:0.000510,Bacteria_WOR_2_RIFOXYC2_FULL_WOR_2_45_15:0.000001):0.1925317456638722[100]):0.21528168799465863[100]):0.04877394585666739[71],(((Ga0172381_10037696:0.066969712278524,Ga0172381_10010070:0.14848921250561897):0.17440022114779952[100],Ga0172381_10009481:0.2529134273416447):0.08219855004442245[99],Ga0172377_10028472:0.28192152899950873):0.3834815169760142[100]):0.041495774125818397[58],(((((((Bacteria_WOR_2_RIFCSPHIGHO2_01_FULL_WOR_2_52_10:0.02780386480276187,Bacteria_WOR_2_GWA2_WOR_2_53_43:0.03168851102436454):0.13856870868162519[100],Bacteria_WOR_2_RIFCSPHIGHO2_02_FULL_WOR_2_50_17:0.1555137428863076):0.06709145984436349[100],Bacteria_WOR_2_RIFCSPHIGHO2_02_FULL_WOR_2_52_10:0.21857794185303492):0.024953375355685647[52],(Bacteria_WOR_2_RIFOXYB2_FULL_WOR_2_38_16:0.20947163917852363,Bacteria_WOR_2_GWA2_WOR_2_45_18:0.18916115160460834):0.03692226521653241[62]):0.09427417405718375[100],(Bacteria_WOR_2_GWA2_WOR_2_47_8:0.22875178509498806,Bacteria_WOR_2_RIFCSPHIGHO2_02_FULL_WOR_2_48_11:0.20752113193311406):0.06389560799556993[99]):0.07553509662396429[100],Ga0172379_10013150:0.41585204667237763):0.19714021067010679[100],(Ga0172380_10042301:0.18143992631631045,Bacteria_Omnitrophica_CG_Omnitr_01:0.1833739024840324):0.27868610170905006[100]):0.04908685581090655[54]):0.06633641473163543[89],(Bacteria_WOR_2_RIFCSPHIGHO2_02_FULL_WOR_2_67_20:0.29792026286399675,Bacteria_WOR_2_RIFCSPHIGHO2_02_FULL_WOR_2_63_39:0.29883224663898833):0.4155091172306298[100]):0.034204769980158645[29],(((Bacteria_WOR_2_RIFCSPLOWO2_12_FULL_WOR_2_51_24:0.09183708375494914,Ga0172381_10014375:0.15876129903588065):0.1141904884983771[100],Bacteria_WOR_2_uncultured_SMTZ_29:0.18565851019632307):0.1726820679877168[100],Bacteria_WOR_2_RIFCSPHIGHO2_02_FULL_WOR_2_68_15:0.570729555307929):0.06943748412847217[48]):0.029990266733683324[40],(((((Ga0172381_10000599:0.21224585458256717,Bacteria_OP3X_RIFCSPLOWO2_02_FULL_OP3X_45_16:0.18260616255581397):0.04373640261471756[74],Bacteria_OP3X_RIFCSPLOWO2_01_FULL_OP3X_45_10:0.2153139477370094):0.183619421040349[100],(Ga0172377_10001206:0.5611950515374975,Bacteria_OP3X_RBG_13_OP3X_46_9:0.34761555754895035):0.09024251059184607[94]):0.10421098338797607[100],((Bacteria_OP3X_GWA2_OP3X_41_15:0.02470956329627727,Bacteria_Omnitrophica_CG_Omnitr_04:0.018368307125965266):0.43170191505541[100],Bacteria_Omnitrophica_CG_Omnitr_03:0.4235745358204479):0.0640204431285798[10]):0.026270523134197532[6],((((Bacteria_Omnitrophica_CG_Omnitr_06:0.07465122239872457,Bacteria_Omnitrophica_CG_Omnitr_05:0.10735253508703968):0.10717980988977027[100],Bacteria_OP3X_RIFCSPHIGHO2_02_FULL_OP3X_51_18:0.15907638169736105):0.06763577484411387[96],Bacteria_OP3X_RIFCSPHIGHO2_02_FULL_OP3X_63_14:0.16501687773170515):0.335856534384233[100],Bacteria_Omnitrophica_CG_Omnitr_07:0.47753951323584065):0.056964104874392074[5]):0.03494250544151889[42]):0.03522797192729232[63],Ga0172381_10027327:0.5561068318911091):0.06272316126115873[69],((((((Ga0172379_10000340:0.1966596958704212,Bacteria_Omnitrophica_GWA2_OP3_52_8:0.23849209868304744):0.06489841970934096[97],(Ga0172380_10000689:0.026214184267184137,Bacteria_Omnitrophica_RifOxyB12_full_OP3_50_7:0.026270987120805778):0.2650695917536168[100]):0.044853330983445794[87],Bacteria_Omnitrophica_GWA2_OP3_52_12:0.2831816525670878):0.035852003464790005[52],Bacteria_CP_OP3_candidate_division_OP3_bacterium_SCGC_AAA257_O07_Etoliko_001_147:0.2358181901607197):0.12022802894153939[100],(((Bacteria_Omnitrophica_RIFCSPLOWO2_12_FULL_Omnitrophica_50_11:0.21171564690733646,Bacteria_Omnitrophica_RIFCSPLOWO2_01_FULL_Omnitrophica_50_24:0.19578238442284635):0.0806100426562888[100],Bacteria_Omnitrophica_RIFCSPLOWO2_01_FULL_Omnitrophica_45_10b:0.20481059172201954):0.0510261280742883[99],Bacteria_Omnitrophica_RIFCSPLOWO2_02_FULL_Omnitrophica_44_11:0.2522088303583816):0.14119999889071666[100]):0.06067797582214318[98],(Bacteria_Omnitrophica_GWA2_OP3_50_21:0.026812939545329595,Bacteria_CP_OP3_candidate_division_OP3_bacterium_SCGC_AAA011_A17_Dusel_001_263:0.03367032363773381):0.281412241766815[100]):0.26873139384848566[100]):0.10439481885102708[100]):0.04540443592019239[25]):0.024807540571111453[1],(((((((((((Bacteria_Elusimicrobia_Elusimicrobia_CG_Elusi_01:0.05886624480190639,Bacteria_Elusimicrobia_RIFCSPHIGHO2_02_FULL_Elusimicrobia_61_10:0.21411748091167393):0.01783876607143231[79],Bacteria_Elusimicrobia_GWC2_Elusimicrobia_61_19:0.044515459307932215):0.014740084691850175[79],Bacteria_Elusimicrobia_GWA2_Elusimicrobia_61_42:0.042617127877205174):0.025529752390730742[96],((Bacteria_Elusimicrobia_GWB2_Elusimicrobia_63_16:0.05327077852309525,Bacteria_Elusimicrobia_GWD2_Elusimicrobia_63_28:0.04639467387660412):0.023834268629120636[99],Bacteria_Elusimicrobia_GWA2_Elusimicrobia_62_23:0.04210020050817498):0.03047219427512049[100]):0.048569727560803244[100],((Bacteria_Elusimicrobia_RifOxyA12_full_Elusimicrobia_57_11:0.06530580863534707,Ga0172379_10025848:0.048580564254637526):0.02826162021811207[90],Bacteria_Elusimicrobia_GWF2_Elusimicrobia_62_30:0.08879523579338144):0.04692831477375714[100]):0.10788194942051632[100],(((Bacteria_Elusimicrobia_RIFOXYA2_FULL_Elusimicrobia_53_38:0.050161574748847304,Bacteria_Elusimicrobia_GWC2_Elusimicrobia_56_31:0.05240526031990056):0.032279006599735816[100],Ga0172379_10000686:0.04022195963975461):0.07002283553970345[100],Bacteria_Elusimicrobia_RIFOXYB2_FULL_Elusimicrobia_62_6:0.14821527785039024):0.07120464346394151[100]):0.04609195684465606[90],Bacteria_Elusimicrobia_GWA2_Elusimicrobia_51_34:0.15907545047614446):0.20932769715959187[100],(Bacteria_Elusimicrobia_Elusimicrobia_Elusimicrobiales_Elusimicrobiaceae_Elusimicrobium_minutum_Pei191:0.3120042729895265,Ga0172379_10006206:0.3066880530650975):0.09787138652733285[100]):0.0942137890370165[100],(((((((Bacteria_Elusimicrobia_RBG_16_Elusimicrobia_66_12:0.021447761748196736,Bacteria_Elusimicrobia_RIFOXYD12_FULL_Elusimicrobia_66_9:0.021467339388063333):0.04017373590997275[100],Bacteria_Elusimicrobia_GWC2_Elusimicrobia_65_9:0.05268854367878362):0.03324391710430019[96],Ga0172380_10019579:0.11471034429339699):0.14693316480799234[100],Bacteria_Elusimicrobia_RIFOXYA2_FULL_Elusimicrobia_69_6:0.2278763793669123):0.05586271026529355[84],Bacteria_Elusimicrobia_RIFCSPHIGHO2_02_FULL_Elusimicrobia_57_9:0.18798271762151275):0.1478301168161802[100],((Bacteria_Elusimicrobia_GWA2_Elusimicrobia_69_24:0.2133339615695098,Bacteria_Elusimicrobia_CG_Elusi_04:0.247603914673852):0.06605335166538184[100],Ga0172379_10000405:0.214265151484057):0.09023877362168742[100]):0.07778634811652374[100],Bacteria_Elusimicrobia_RIFCSPLOWO2_12_FULL_Elusimicrobia_59_9:0.41446129397816556):0.056955811379547416[91]):0.16775837609561428[100],((((((Ga0172377_10036189:0.000001,Ga0172381_10021976:0.002465225207114674):0.20378410085761756[100],(Bacteria_Elusimicrobia_Endomicrobia_Candidatus_Endomicrobium_trichonymphae_submission_450:0.05174283280378589,Bacteria_Elusimicrobia_Elusimicrobia_uncultured_Termite_group_1_bacterium_phylotype_Rs_D17:0.05085633258186828):0.1947363262177655[100]):0.16139970357484978[100],((Bacteria_Elusimicrobia_RIFOXYA2_FULL_Elusimicrobia_47_53:0.1789202986892935,Bacteria_Elusimicrobia_RIFOXYB2_FULL_Elusimicrobia_50_12:0.15944716939719816):0.0440099584763578[92],Bacteria_Elusimicrobia_RIFOXYB2_FULL_Elusimicrobia_49_7:0.1807828935157323):0.08194458569535001[100]):0.11083855203371984[100],((((Bacteria_Elusimicrobia_RIFCSPLOWO2_01_FULL_Elusimicrobia_54_10:0.0982723430231478,Bacteria_Elusimicrobia_RIFCSPLOWO2_01_FULL_Elusimicrobia_60_11:0.08095885325987773):0.13272830799854773[100],Bacteria_Elusimicrobia_RIFCSPLOWO2_01_FULL_Elusimicrobia_64_13:0.22478770192030773):0.06050787501611232[94],Bacteria_Elusimicrobia_RIFCSPLOWO2_02_FULL_Elusimicrobia_39_32:0.28090550469550024):0.2265850528272404[100],Bacteria_Elusimicrobia_RIFCSPLOWO2_01_FULL_Elusimicrobia_59_12:0.47849601442974743):0.047366083860994834[63]):0.031083785214587323[33],(Bacteria_Elusimicrobia_RIFOXYD2_FULL_Elusimicrobia_34_15:0.06232096632301598,Bacteria_Elusimicrobia_RIFOXYD2_FULL_Elusimicrobia_34_30:0.06262952947507783):0.4164795329780677[100]):0.038360493290593656[49],(((Bacteria_Elusimicrobia_RIFOXYA2_FULL_Elusimicrobia_40_6:0.09495550826341947,Bacteria_Elusimicrobia_RIFOXYB2_FULL_Elusimicrobia_48_7:0.12491358749981218):0.10072153372432036[100],Bacteria_Elusimicrobia_RIFOXYA2_FULL_Elusimicrobia_39_19:0.18362027851907925):0.14546040226110835[100],Bacteria_Elusimicrobia_CG_Elusi_05:0.2740145927168438):0.18820767667932703[100]):0.06679534146112243[90]):0.1829395084285328[100],(Bacteria_Elusimicrobia_CG_Elusi_03:0.32905561604677747,Bacteria_Elusimicrobia_CG_Elusi_02:0.32541216155648245):0.24823110033373652[100]):0.0563581244415734[72]):0.04020698296771341[3],((((((((Bacteria_CP_TM6_gwf2_TM6_43_87:0.16333907594031327,Bacteria_CP_TM6_GWE2_TM6_42_60:0.17626244001210223):0.07814772458161601[100],Bacteria_CP_TM6_GWF2_TM6_43_17_partial:0.2897405744865966):0.16550295784115754[100],Bacteria_CP_TM6_GWE2_TM6_41_16:0.38870555109467686):0.05856698359977974[97],Ga0172381_10007264:0.32098793029314576):0.0702575728964665[99],(Bacteria_CP_TM6_GWF2_TM6_30_66:0.3394036563192877,Bacteria_TM6_bacterium_JCVI_TM6SC1:0.35447983172634334):0.06190598691006155[83]):0.03588417660286858[45],(((Bacteria_TM6_RIFCSPHIGHO2_12_FULL_TM6_32_22:0.33120577402963347,Bacteria_TM6_RIFCSPHIGHO2_12_FULL_TM6_36_22:0.3740809607636457):0.08540525384503672[97],(Bacteria_TM6_RIFCSPHIGHO2_12_FULL_TM6_38_8:0.31872825822675166,Ga0172380_10000189:0.2507911020801772):0.19981965479678188[100]):0.03668351340913967[55],Bacteria_CP_TM6_GWF2_TM6_32_72:0.3192796558071267):0.031816266950945415[42]):0.10397514071680813[99],(((((((Ga0172379_10001116:0.000001,Ga0172381_10033047:0.000001):0.0013126667493112976[88],Bacteria_CP_TM6_GWF2_TM6_36_6_partial:0.000001):0.17747267425162283[100],Bacteria_CP_TM6_GWF2_TM6_37_49:0.20276343197956148):0.10348707876785834[100],((Bacteria_CP_TM6_GWF2_TM6_38_10:0.15361727270935788,Ga0172379_10023036:0.13798809831405112):0.0869729234364649[100],Ga0172379_10002078:0.32883955680763455):0.030032318380575074[28]):0.03720081779007245[59],Ga0172379_10014369:0.2610365937938921):0.09425355878324382[99],Bacteria_CP_TM6_GWF2_TM6_28_16:0.3014255750230408):0.04468611177580817[37],Ga0172379_10020229:0.3431618612843099):0.1332249082194803[100]):0.45595556671215665[100],(((Bacteria_RIF1_GWA2_RIF01_43_8:0.021844041289428073,Bacteria_RIF1_RIFOXYA2_FULL_RIF01_40_8:0.03226144160770694):0.022509153686263605[97],Bacteria_RIF1_RIFOXYC2_FULL_RIF01_39_67:0.018304750053399488):0.6046465916354351[100],Bacteria_RIF2_RIFCSPLOWO2_12_FULL_RIF02_62_27:0.8008499524696524):0.05607695665140788[17]):0.0735071294135814[22]):0.028291613356539624[8],(((Ga0172382_10000367:0.000001,Ga0172377_10059698:0.000001):0.45811375165372903[100],(Ga0172377_10021940:0.26068988619634803,Ga0172382_10000355:0.24252079189093534):0.1874462718713632[100]):0.19146043503796895[100],(Bacteria_Omnitrophica_CG_Omnitr_02:0.4664684505641965,Ga0172380_10009798:0.6836318086923234):0.07006558808637164[25]):0.22920938753790265[100]):0.05481616134985057[74],((((((((((((((((((((((((((((((((((((Ga0172381_10016268:0.10772817132131163,Ga0172381_10005703:0.0682879412056625):0.09389853670415427[100],Ga0172378_10003465:0.1447895973569251):0.08027601371638937[100],Ga0172379_10000395:0.2021432804839307):0.06934022668627504[97],Ga0172380_10014668:0.22944732427090964):0.08519784270241892[100],(Bacteria_Parcubacteria_OD1_RIF_OD1_11_RIFOXYD2_FULL_RIF_OD1_11_36_9:0.09656793986886303,Ga0172380_10002453:0.08165418570805638):0.1489612745784985[100]):0.0472405111112959[59],Ga0172379_10011013:0.3221845721136605):0.03261710608781421[78],Bacteria_CPR_ParcubacteriaOD1_GWA1_OD1_40_21:0.2500889249122107):0.049779834100608156[91],(Ga0172379_10007898:0.39172812089210574,Ga0172379_10008460:0.3023068537682203):0.05311169758599066[71]):0.04513297467428723[87],Ga0172379_10000653:0.32401598414748856):0.024324969517793615[33],(((((Ga0172379_10000461:0.091228230198519,Ga0172380_10018950:0.12175838251604976):0.022869705612348135[74],Ga0172379_10015244:0.1100490930292426):0.06610134904793341[100],Bacteria_Parcubacteria_OD1_RIF_OD1_11_RIFCSPHIGHO2_12_FULL_RIF_OD1_11_45_16:0.1492170159855033):0.036136729283342106[81],((Ga0172379_10000185:0.15607553842213884,Bacteria_Parcubacteria_OD1_Yanofskybacteria_RIFCSPHIGHO2_02_FULL_OD1_Yanofskybacteria_44_12:0.2009391096192048):0.03318008608142309[42],Ga0172379_10000531:0.18795701552455446):0.0304301084969536[67]):0.21383062738215797[100],(Ga0172380_10030975:0.027433440561743794,Ga0172379_10008825:0.03561910104584198):0.3177571454992387[100]):0.06925296739343523[95]):0.0193222191023259[42],(((((((Bacteria_CPR_ParcubacteriaOD1_GWA2_OD1_47_64:0.10698413575754184,Bacteria_Parcubacteria_OD1_RIF_OD1_11_RIFCSPLOWO2_02_RIF_OD1_11_50_13:0.1439164012335823):0.06977986150331184[100],Bacteria_Parcubacteria_OD1_RIF_OD1_11_RIFCSPHIGHO2_01_FULL_RIF_OD1_11_51_15:0.13922551178600084):0.1476198403004232[100],(Bacteria_CPR_ParcubacteriaOD1_GWA2_OD1_47_12:0.022489914187761695,Bacteria_Parcubacteria_OD1_RIF_OD1_11_RIFCSPHIGHO2_02_FULL_RIF_OD1_11_47_18:0.030447641786889523):0.3329227941638311[100]):0.060099793514390765[95],Bacteria_Parcubacteria_OD1_RIF_OD1_11_RIFCSPHIGHO2_02_FULL_RIF_OD1_11_43_32b:0.42506035091338124):0.04965509316353112[79],(((Bacteria_CPR_ParcubacteriaOD1_GWA2_OD1_like_47_16:0.1205822947966313,Ga0172379_10007417:0.17677786350686997):0.045961576592030706[95],Bacteria_CPR_ParcubacteriaOD1_GWA2_OD1_44_15_partial:0.16228193684784564):0.1337775271781787[100],Ga0172379_10008454:0.29358385100437):0.053590282170282055[71]):0.04165727703524036[93],((((Bacteria_Parcubacteria_OD1_RIF_OD1_11_RIFCSPLOWO2_02_FULL_RIF_OD1_11_44_35:0.07780944370684884,Bacteria_CPR_ParcubacteriaOD1_GWB1_OD1_44_7:0.0759088802747443):0.03098691320786706[74],Ga0172379_10012751:0.08273544743903471):0.27427842850751993[100],Bacteria_Parcubacteria_OD1_RIF_OD1_11_RIFCSPLOWO2_12_FULL_RIF_OD1_11_47_20:0.3505476791547997):0.06757051276669435[37],(((Bacteria_Parcubacteria_OD1_RIF_OD1_11_RIFCSPHIGHO2_01_FULL_RIF_OD1_11_46_22b:0.19941990848898605,Bacteria_Parcubacteria_OD1_RIF_OD1_11_RIFCSPHIGHO2_02_FULL_RIF_OD1_11_46_13:0.1848855361871622):0.15738359736889507[100],Bacteria_CPR_ParcubacteriaOD1_GWA2_OD1_47_21_part:0.29719840524612806):0.05490582810970368[64],Bacteria_Parcubacteria_OD1_RIF_OD1_11_RIFCSPHIGHO2_02_FULL_RIF_OD1_11_45_35:0.27327344058124137):0.034934151666822455[13]):0.05522834260842968[99]):0.025763770792947227[38],(Bacteria_Parcubacteria_OD1_RIF_OD1_11_RIFCSPLOWO2_01_FULL_RIF_OD1_11_45_15b:0.4279985438569116,Bacteria_Parcubacteria_OD1_RIF_OD1_11_RIFCSPLOWO2_12_FULL_RIF_OD1_11_43_20:0.35925316947144736):0.09347667680383287[97]):0.028950701654290256[69]):0.029408674584014083[87],((((((((((((Bacteria_CPR_ParcubacteriaOD1_GWD1_OD1_44_9_partial:0.06315138077465088,Bacteria_Parcubacteria_OD1_RIF_OD1_1_RIFCSPLOWO2_01_FULL_RIF_OD1_10_47_33:0.07610524764490778):0.011738167026933866[53],Bacteria_CPR_ParcubacteriaOD1_GWA1_OD1_49_11_partial:0.09696050712689175):0.03641306211583517[99],Bacteria_Parcubacteria_OD1_RIF_OD1_1_RIFOXYD2_FULL_RIF_OD1_10_43_10:0.11921792280963617):0.031735655151987174[100],Bacteria_Parcubacteria_OD1_RIF_OD1_1_RIFCSPHIGHO2_01_FULL_RIF_OD1_10_43_60:0.10681019313537155):0.021833750232167937[71],Bacteria_Parcubacteria_OD1_RIF_OD1_1_RIFCSPLOWO2_02_FULL_RIF_OD1_10_44_12b:0.11101473099774006):0.0350415590050841[94],Bacteria_Parcubacteria_OD1_RIF_OD1_1_RIFCSPHIGHO2_02_FULL_RIF_OD1_10_39_16:0.12456592253062837):0.036233148720732355[91],Bacteria_Parcubacteria_OD1_RIF_OD1_1_RIFCSPHIGHO2_01_FULL_RIF_OD1_10_44_22b:0.1986940818631715):0.02686995559994987[88],(((Bacteria_Parcubacteria_OD1_RIF_OD1_1_RIFCSPHIGHO2_01_FULL_RIF_OD1_10_49_18:0.11881455416710507,Bacteria_Parcubacteria_OD1_RIF_OD1_1_RIFCSPHIGHO2_02_FULL_RIF_OD1_10_43_37:0.08766957534676356):0.046372223185086714[100],Bacteria_Parcubacteria_OD1_RIF_OD1_1_RIFCSPHIGHO2_01_FULL_RIF_OD1_10_46_25:0.11502689717013892):0.07842297349714444[100],Bacteria_CPR_ParcubacteriaOD1_GWA2_OD1_50_10_part:0.1242153708652709):0.03906296818809185[95]):0.09312443624724276[100],Ga0172379_10000927:0.206784402210193):0.14551008795465092[100],((Bacteria_Parcubacteria_OD1_RIF_OD1_1_RIFCSPHIGHO2_12_FULL_RIF_OD1_10_44_12b:0.3260743165263036,Bacteria_Parcubacteria_OD1_RIF_OD1_1_RIFCSPHIGHO2_12_FULL_RIF_OD1_10_43_12b:0.23294189499436113):0.05978084234986447[68],Ga0172379_10000646:0.4042579650783025):0.06079507007518181[98]):0.05263619619740201[90],(((((Bacteria_Parcubacteria_OD1_RIF_OD1_1_RIFCSPHIGHO2_02_FULL_RIF_OD1_10_39_19:0.0824654073295088,Ga0172379_10000764:0.11592031031738648):0.04569716153321757[99],Bacteria_Parcubacteria_OD1_RIF_OD1_1_RIFOXYD1_FULL_RIF_OD1_10_39_35:0.117576955662825):0.02443216427406103[51],Bacteria_Parcubacteria_OD1_RIF_OD1_1_RIFCSPLOWO2_12_FULL_RIF_OD1_10_39_16:0.1275033195131079):0.04372463995015696[86],Bacteria_Parcubacteria_OD1_RIF_OD1_1_RIFCSPLOWO2_01_FULL_RIF_OD1_10_35_19:0.13130443622563392):0.2619728554835805[100],((((Bacteria_CPR_ParcubacteriaOD1_GWC2_OD1_40_10:0.0926525761449839,Ga0172379_10000242:0.09427380129520468):0.05589033299872259[100],Bacteria_Parcubacteria_OD1_RIF_OD1_1_RIFCSPLOWO2_02_FULL_RIF_OD1_10_39_26:0.15049068611279193):0.034142403588217185[90],Bacteria_Parcubacteria_OD1_RIF_OD1_1_RIFCSPLOWO2_02_RIF_OD1_10_39_10:0.1692126736924875):0.08741552744859327[100],Ga0172380_10000054:0.21362643879347631):0.09031066276523836[100]):0.03647674691148328[74]):0.021625756085053904[48],(Ga0172380_10000146:0.275600061340596,Ga0172380_10004583:0.40647057675790554):0.06043686904142076[79]):0.05486576572817281[99]):0.023949785551550917[58],((((Ga0172380_10027162:0.4489292944010863,Ga0172379_10003823:0.21828193921292804):0.06465459900208836[77],Ga0172379_10007782:0.3016042976305311):0.05285229177599016[91],(Ga0172380_10007682:0.3001469710116407,Ga0172380_10000007:0.316000485281676):0.13522848536066512[100]):0.03850374790476385[45],Ga0172379_10005459:0.31672504088720643):0.0323854490554063[17]):0.024344283256414023[23],(((Bacteria_Parcubacteria_OD1_Campbellbacteria_RIFCSPLOWO2_02_FULL_OD1_Campbellbacteria_35_11:0.1092857869452657,Bacteria_CPR_ParcubacteriaOD1_Campbellbacteria_GWD2_OD1_35_24:0.10176626750862194):0.08357003413865094[100],Bacteria_Parcubacteria_OD1_Campbellbacteria_RIFOXYC2_FULL_OD1_Campbellbacteria_35_25:0.1567661851072759):0.16349658915504905[100],Bacteria_Parcubacteria_OD1_Campbellbacteria_RIFCSPLOWO2_02_OD1_Campbellbacteria_35_12:0.3166920728326965):0.06969121925420074[52]):0.019103105932100295[24],(((((((((((((((((Bacteria_Parcubacteria_OD1_Kaiserbacteria_RIFCSPHIGHO2_02_FULL_OD1_Kaiserbacteria_54_22:0.08961158540911951,Bacteria_CPR_ParcubacteriaOD1_GWB1_OD1_57_6:0.13736891493781966):0.027464967407609286[62],Bacteria_Parcubacteria_OD1_Kaiserbacteria_RIFCSPLOWO2_01_FULL_OD1_Kaiserbacteria_52_12b:0.10810398250502251):0.010816154311542814[30],Ga0172379_10041495:0.06886431058039078):0.01764176446166621[32],Bacteria_Parcubacteria_OD1_Kaiserbacteria_RIFCSPHIGHO2_02_FULL_OD1_Kaiserbacteria_55_17:0.1041993453880985):0.013208037505640036[23],Bacteria_Parcubacteria_OD1_Kaiserbacteria_RIFCSPLOWO2_01_FULL_OD1_Kaiserbacteria_55_19:0.13277965395170055):0.02821530707601827[37],(Bacteria_Parcubacteria_OD1_Kaiserbacteria_GWA2_OD1_Kaiserbacteria_50_9:0.08959172618333167,Ga0172379_10000203:0.07058148892632721):0.02159706445973031[71]):0.017516769004437105[10],(((((Ga0172379_10033490:0.04307663347680846,Ga0172379_10003079:0.03611690929779732):0.030259512662402788[77],Ga0172379_10001141:0.08852263170436103):0.02289832593250729[52],Ga0172379_10016367:0.09646137498602503):0.04402078942122323[100],Ga0172379_10013727:0.09633968736175058):0.025182711258805668[37],(Ga0172379_10003133:0.08102210210191263,Ga0172379_10002858:0.08061262618964582):0.028598223197433992[97]):0.021220999549418806[51]):0.02995965778779963[44],(((Ga0172379_10002172:0.08128495309148365,Ga0172379_10000418:0.05452425808909078):0.05240776310526485[100],(Ga0172379_10001251:0.07100275074746287,Bacteria_Parcubacteria_OD1_Kaiserbacteria_RIFOXYD1_FULL_OD1_Kaiserbacteria_47_14:0.06373434145010703):0.05120204331639977[100]):0.04075882996836366[100],Ga0172379_10002668:0.12318549390158173):0.019342514019322632[14]):0.020803397860739814[18],Bacteria_Parcubacteria_OD1_CG_CPR_09_CG_CPR09_01:0.1411489053676629):0.02946350514805296[47],Bacteria_Parcubacteria_OD1_Kaiserbacteria_RIFCSPHIGHO2_01_FULL_OD1_Kaiserbacteria_53_31:0.14816088032299035):0.10831381922448102[100],Bacteria_Parcubacteria_OD1_Kaiserbacteria_RIFCSPHIGHO2_12_FULL_OD1_Kaiserbacteria_56_13:0.24219849630486756):0.10265032442919608[100],Ga0172380_10000484:0.25496442525984264):0.15114082595170064[100],(((((((Bacteria_Parcubacteria_OD1_Kaiserbacteria_RIFCSPLOWO2_01_FULL_OD1_Kaiserbacteria_54_24:0.11909468752303098,Bacteria_Parcubacteria_OD1_Kaiserbacteria_RIFCSPHIGHO2_02_FULL_OD1_Kaiserbacteria_59_21:0.11308844313692967):0.04269668950631189[99],Bacteria_Parcubacteria_OD1_Kaiserbacteria_RIFCSPHIGHO2_01_FULL_OD1_Kaiserbacteria_55_17:0.15610725711456652):0.029562403049016694[76],Bacteria_Parcubacteria_OD1_Kaiserbacteria_RIFCSPLOWO2_01_FULL_OD1_Kaiserbacteria_54_13:0.16968023760072937):0.035055656723513184[83],(Bacteria_Parcubacteria_OD1_Kaiserbacteria_RIFCSPLOWO2_02_FULL_OD1_Kaiserbacteria_51_13:0.26077101963444616,Bacteria_CPR_ParcubacteriaOD1_Kaiserbacteria_GWB1_OD1_50_17_partial:0.21666417610329258):0.0589562290335226[66]):0.03559639365208378[65],((((Bacteria_Parcubacteria_OD1_Kaiserbacteria_RIFCSPHIGHO2_02_FULL_OD1_Kaiserbacteria_54_11b:0.13751329141563717,Bacteria_Parcubacteria_OD1_Kaiserbacteria_RIFCSPHIGHO2_02_FULL_OD1_Kaiserbacteria_55_20:0.0889323867399745):0.03700568804931015[97],Bacteria_Parcubacteria_OD1_Kaiserbacteria_RIFCSPLOWO2_01_FULL_OD1_Kaiserbacteria_54_22:0.10943121017299795):0.021979457527369295[60],Bacteria_Parcubacteria_OD1_Kaiserbacteria_RIFCSPHIGHO2_01_FULL_OD1_Kaiserbacteria_53_29:0.09953127606750245):0.051526509930449826[100],Bacteria_CPR_ParcubacteriaOD1_Kaiserbacteria_GWA2_OD1_52_12:0.1933379936623445):0.038300918346616086[96]):0.028557740233403184[56],(((Bacteria_Parcubacteria_OD1_RIFCSPHIGHO2_01_FULL_OD1_56_18:0.1121465623628195,Bacteria_Parcubacteria_OD1_Kaiserbacteria_RIFCSPHIGHO2_01_FULL_OD1_Kaiserbacteria_56_24:0.0846144910065254):0.03902670948245213[99],Bacteria_Parcubacteria_OD1_Kaiserbacteria_RIFCSPLOWO2_01_FULL_OD1_Kaiserbacteria_53_17:0.10525952925881654):0.14059687676178934[100],(Ga0172380_10027214:0.14630791464677584,Bacteria_Parcubacteria_OD1_Kaiserbacteria_RIFCSPLOWO2_01_FULL_OD1_Kaiserbacteria_55_25:0.1377345906125389):0.049791012004651414[95]):0.031630336204187426[70]):0.03913450276662633[52],(Bacteria_Parcubacteria_OD1_Kaiserbacteria_RIFCSPHIGHO2_12_FULL_OD1_Kaiserbacteria_53_13:0.11425017627069156,Bacteria_Parcubacteria_OD1_Kaiserbacteria_RIFCSPHIGHO2_02_FULL_OD1_Kaiserbacteria_49_16:0.17016291789812765):0.08042575264588292[100]):0.23581699508239584[100]):0.06038600969928387[68],((((((Ga0172380_10001055:0.1658224936045012,Ga0172380_10000543:0.1344567165928927):0.09868997356544806[100],Bacteria_CPR_ParcubacteriaOD1_L1_Parcubacteria_bacterium_SCGC_AAA040_L21_CrabSpa_001_179:0.1904190120671574):0.04454632049774343[97],(Bacteria_Parcubacteria_OD1_Kaiserbacteria_RIFCSPLOWO2_12_FULL_OD1_Kaiserbacteria_50_10:0.04008553813859894,Bacteria_Parcubacteria_OD1_Kaiserbacteria_RIFCSPHIGHO2_02_FULL_OD1_Kaiserbacteria_49_34:0.045455034315868836):0.361419269738843[100]):0.024776969489165968[56],((Bacteria_Parcubacteria_OD1_Kaiserbacteria_RIFOXYD1_FULL_OD1_Kaiserbacteria_42_15:0.18680097493443482,Bacteria_Parcubacteria_OD1_Kaiserbacteria_RIFCSPLOWO2_12_FULL_OD1_Kaiserbacteria_45_26:0.13323474669651691):0.08366718851719401[100],(Bacteria_Parcubacteria_OD1_RIF_OD1_11_RIFCSPHIGHO2_01_FULL_RIF_OD1_11_46_22:0.23815610003576504,Bacteria_Parcubacteria_OD1_CG_CPR_18_CG_CPR18_01:0.2153485983876413):0.03724975777703721[70]):0.04785388695130832[97]):0.07026179878587113[100],Ga0172380_10007488:0.27883810907602813):0.11050247357436893[100],Bacteria_Parcubacteria_OD1_Kaiserbacteria_RIFCSPHIGHO2_01_FULL_OD1_Kaiserbacteria_48_10:0.39506343160747504):0.0414806648785051[55]):0.034996405452192736[65],((Ga0172379_10038504:0.24254961002563302,Bacteria_CPR_ParcubacteriaOD1_GWA2_OD1_43_11:0.1956253709029614):0.211438913841147[100],Ga0172380_10002581:0.44539075134049844):0.04018722645318329[13]):0.03784710599037533[21],((Bacteria_Parcubacteria_OD1_Kaiserbacteria_RIFCSPLOWO2_01_FULL_OD1_Kaiserbacteria_51_21:0.2097769764144135,Bacteria_Parcubacteria_OD1_Kaiserbacteria_RIFCSPHIGHO2_01_FULL_OD1_Kaiserbacteria_49_13:0.1755061001975715):0.1657195310380084[100],Bacteria_Parcubacteria_OD1_Kaiserbacteria_RIFCSPHIGHO2_02_FULL_OD1_Kaiserbacteria_49_11:0.3247643113837002):0.0638186148045694[79]):0.03632992810713542[19],(((((Ga0172379_10000335:0.08289963376688236,Ga0172379_10004529:0.07080102442713443):0.1191521360234784[100],(Ga0172379_10017525:0.029687231315692397,Bacteria_Parcubacteria_OD1_Adlerbacteria_RIFOXYC1_FULL_OD1_Adlerbacteria_48_26:0.04765906283529997):0.15896417351366[100]):0.0495990959435022[92],(((Ga0172380_10019499:0.13801525040061158,Ga0172379_10008999:0.17695638569042593):0.03684821045496056[25],Bacteria_Parcubacteria_OD1_Adlerbacteria_RIFCSPLOWO2_01_FULL_OD1_Adlerbacteria_54_21b:0.19745655983561772):0.04173348964337498[49],Bacteria_Parcubacteria_OD1_Adlerbacteria_RIFCSPHIGHO2_01_FULL_OD1_Adlerbacteria_54_23:0.23381141205109968):0.053270425468481264[86]):0.024211191828340972[40],(Bacteria_Parcubacteria_OD1_Adlerbacteria_RIFCSPHIGHO2_02_FULL_OD1_Adlerbacteria_52_17:0.22963143540223108,Bacteria_Parcubacteria_OD1_Adlerbacteria_RIFCSPHIGHO2_12_FULL_OD1_Adlerbacteria_53_18:0.1452933335645299):0.0412926432324916[58]):0.041381361751429147[50],Bacteria_Parcubacteria_OD1_Adlerbacteria_RIFCSPLOWO2_01_FULL_OD1_Adlerbacteria_51_16:0.20581197173766919):0.24941085251221606[100]):0.05495124176848565[27]):0.0349697103602562[49],(((((Bacteria_Parcubacteria_OD1_RIF_OD1_9_RIFCSPLOWO2_02_FULL_RIF_OD1_09_54_12:0.09861606211462526,Bacteria_Parcubacteria_OD1_RIF_OD1_9_RIFCSPHIGHO2_02_FULL_RIF_OD1_09_50_13:0.09252602693003631):0.049653182719917144[100],Bacteria_Parcubacteria_OD1_RIF_OD1_9_RIFCSPLOWO2_01_FULL_RIF_OD1_09_50_20:0.11448244032801558):0.07285314772084384[100],(Bacteria_Parcubacteria_OD1_RIF_OD1_9_RifOxyC12_full_RIF_OD1_09_46_25:0.15413876144100858,Bacteria_Parcubacteria_OD1_RIF_OD1_9_RIFCSPHIGHO2_01_FULL_RIF_OD1_09_49_22:0.17574268994689834):0.053131206453757684[83]):0.27626346431623183[100],Bacteria_Parcubacteria_OD1_RIF_OD1_9_RIFCSPLOWO2_02_FULL_RIF_OD1_09_51_11:0.47915553792384946):0.06499903711558908[74],(((Ga0172379_10003279:0.046044078702540236,Ga0172379_10015281:0.048203398543617215):0.06537816469327451[100],(Ga0172379_10015886:0.05993624123270003,Ga0172379_10002346:0.07466229810395619):0.058613687235379075[99]):0.09385464681082833[100],Ga0172379_10010611:0.1416816067612663):0.19923595431329177[100]):0.026441352228403936[28]):0.01869485554795247[3],((((((((((((((((((Bacteria_CPR_ParcubacteriaOD1_Nomurabacteria_GWA2_OD1_43_15:0.060529566496612563,Ga0172379_10005375:0.10226596499228524):0.02569189789522275[95],Ga0172379_10003847:0.04412818008888708):0.020160059727084345[92],(Bacteria_CPR_ParcubacteriaOD1_Nomurabacteria_GWA2_OD1_41_25_partial:0.07096508210634411,Bacteria_CPR_ParcubacteriaOD1_Nomurabacteria_GWA1_OD1_43_17:0.08230263332947496):0.01667521827223073[74]):0.014322913932653059[67],((Bacteria_Parcubacteria_OD1_Nomurabacteria_GWB1_OD1_Nomurabacteria_40_6:0.030097814525889444,Bacteria_Parcubacteria_OD1_Nomurabacteria_RIFOXYB1_FULL_OD1_Nomurabacteria_39_16:0.023456742971756928):0.041637579479056885[100],(Ga0172379_10016044:0.04819144771364492,Ga0172379_10023455:0.02861133807685512):0.022960406576977288[74]):0.02202723696023101[87]):0.012765196204726603[64],(((Bacteria_CPR_ParcubacteriaOD1_Nomurabacteria_GWB1_OD1_47_6_part:0.07188666539407418,Ga0172379_10000042:0.06900295625476716):0.09304633091003245[100],(Bacteria_CPR_ParcubacteriaOD1_Nomurabacteria_GWB1_OD1_40_7:0.06913746908435447,Ga0172379_10000829:0.05049777508969422):0.030723344212399084[100]):0.013485608778272873[36],((Ga0172379_10000135:0.04951583610120247,Ga0172379_10022855:0.06137552132470203):0.017893566994562793[38],Ga0172379_10000084:0.049195816576039775):0.036963779307307654[88]):0.012781174479894464[42]):0.012444053328302562[81],((Ga0172379_10003307:0.04438856461177698,Ga0172379_10025747:0.058632562713498704):0.033408976858157136[100],(Ga0172379_10000740:0.05850188685744229,Ga0172379_10004386:0.04981169026127974):0.018247676561597093[82]):0.0214826167111819[97]):0.01430960930847247[92],Bacteria_Parcubacteria_OD1_Nomurabacteria_RIFCSPHIGHO2_01_FULL_OD1_Nomurabacteria_38_19:0.1645209707980424):0.008857415608062968[19],((((((((Bacteria_Parcubacteria_OD1_Nomurabacteria_RIFCSPLOWO2_01_FULL_OD1_Nomurabacteria_40_18:0.07506197399194647,Bacteria_Parcubacteria_OD1_Nomurabacteria_RIFCSPLOWO2_12_FULL_OD1_Nomurabacteria_40_42:0.09348056600192445):0.015214757653738697[58],Bacteria_Parcubacteria_OD1_Nomurabacteria_RIFCSPLOWO2_01_FULL_OD1_Nomurabacteria_42_17:0.07530268423870679):0.009786068185857921[42],((Ga0172379_10004827:0.03294803363655596,Bacteria_Parcubacteria_OD1_Nomurabacteria_RIFOXYC2_FULL_OD1_Nomurabacteria_36_19:0.03882905978976048):0.04418784427236844[100],Bacteria_Parcubacteria_OD1_Nomurabacteria_RIFCSPHIGHO2_01_FULL_OD1_Nomurabacteria_39_10:0.07324082234715057):0.015603425722131803[80]):0.011363138168330789[41],Bacteria_Parcubacteria_OD1_Nomurabacteria_RIFCSPLOWO2_01_FULL_OD1_Nomurabacteria_41_18:0.09012227438332232):0.015570930398119742[60],Bacteria_CPR_ParcubacteriaOD1_Nomurabacteria_GWA1_OD1_37_20_partial:0.09382157708274264):0.012350663640296133[55],(Bacteria_CPR_ParcubacteriaOD1_GWB1_OD1_36_5:0.06997868454801415,Bacteria_Parcubacteria_OD1_Nomurabacteria_RIFCSPHIGHO2_02_FULL_OD1_Nomurabacteria_36_29:0.09255823917293027):0.014897772273962495[71]):0.04318303579201599[100],(((Bacteria_Parcubacteria_OD1_Nomurabacteria_RIFCSPLOWO2_12_FULL_OD1_Nomurabacteria_37_8:0.0940431638290824,Ga0172379_10008958:0.10306073925630921):0.012252925261314829[31],(Bacteria_Parcubacteria_OD1_Nomurabacteria_RIFCSPLOWO2_01_FULL_OD1_Nomurabacteria_41_12:0.06117146252997818,Bacteria_Parcubacteria_OD1_Nomurabacteria_RIFCSPHIGHO2_01_FULL_OD1_Nomurabacteria_40_12:0.0655514114670055):0.04232642253008301[100]):0.012689681921393081[49],(Bacteria_Parcubacteria_OD1_Nomurabacteria_RIFCSPLOWO2_01_FULL_OD1_Nomurabacteria_37_25:0.0906693545351338,Bacteria_Parcubacteria_OD1_Nomurabacteria_RIFCSPLOWO2_01_FULL_OD1_Nomurabacteria_39_18:0.12145506962556762):0.024622942662126945[50]):0.010615983359147485[31]):0.015662295492808287[41],Bacteria_CPR_ParcubacteriaOD1_Nomurabacteria_GWF2_OD1_40_31:0.11095024001592169):0.01551409305745377[46]):0.024047936834386885[79],(((Bacteria_Parcubacteria_OD1_Nomurabacteria_RIFCSPHIGHO2_02_FULL_OD1_Nomurabacteria_42_19:0.09508467167129098,Bacteria_Parcubacteria_OD1_Nomurabacteria_RIFCSPLOWO2_12_FULL_OD1_Nomurabacteria_44_11:0.1208010583324386):0.04578912773410071[100],Bacteria_Parcubacteria_OD1_Nomurabacteria_RIFCSPLOWO2_01_FULL_OD1_Nomurabacteria_41_52b:0.18071389345372113):0.022133860275589168[58],(Bacteria_Parcubacteria_OD1_Nomurabacteria_RIFCSPLOWO2_12_FULL_OD1_Nomurabacteria_46_14:0.1840560459247338,Bacteria_Parcubacteria_OD1_Nomurabacteria_RIFCSPLOWO2_01_FULL_OD1_Nomurabacteria_46_18:0.1766239707655517):0.041461324726508764[79]):0.024831226257748362[33]):0.022928084886429723[36],((Ga0172379_10027120:0.09887522305482044,Bacteria_Parcubacteria_OD1_Nomurabacteria_RIFCSPLOWO2_01_FULL_OD1_Nomurabacteria_39_17:0.1153065034275329):0.0534243475156817[100],Bacteria_Parcubacteria_OD1_Nomurabacteria_RIFCSPLOWO2_01_FULL_OD1_Nomurabacteria_40_15:0.1262643603427982):0.019084443405898988[33]):0.01642277233353706[17],(((((((Ga0172379_10044635:0.020020452993966487,Bacteria_Parcubacteria_OD1_Nomurabacteria_CG_Nomura_03:0.02099822568946763):0.027391325109869857[100],Bacteria_CPR_ParcubacteriaOD1_Nomurabacteria_GWE2_OD1_31_40:0.06318606521797232):0.027024595238188454[100],Ga0172379_10039076:0.1040318304449368):0.017739449395765217[73],(Bacteria_CP_OD1_candidate_division_OD1_bacterium_SCGC_AAA011_A08_DUSEL_001_185:0.10450183645157685,Bacteria_Parcubacteria_OD1_Nomurabacteria_RIFCSPHIGHO2_02_FULL_OD1_Nomurabacteria_35_13:0.06272516341406265):0.02663935605328005[99]):0.007467829676068849[26],Ga0172379_10000566:0.07401028356632988):0.01945786810070871[83],Ga0172381_10015237:0.11971411417934252):0.03994831742871785[100],((Ga0172381_10000809:0.04488432336481818,Ga0172381_10003975:0.02712898460767832):0.08743291297299693[100],(Ga0172381_10021018:0.10033531498127113,Ga0172379_10001832:0.06209087142300307):0.020087517542301647[51]):0.025840191089186337[85]):0.02087928733055966[83]):0.025501716729520396[42],((Bacteria_CPR_ParcubacteriaOD1_Nomurabacteria_GWA2_OD1_40_9:0.11715831816420241,Ga0172380_10000274:0.17917597044421196):0.044953723836937964[94],Bacteria_Parcubacteria_OD1_Nomurabacteria_RIFCSPHIGHO2_01_FULL_OD1_Nomurabacteria_40_20:0.15104530250652815):0.02408150835187106[40]):0.036149585486504154[63],Ga0172381_10007855:0.19486094596037606):0.17196602491863144[100],((((((((((Ga0172380_10038413:0.21684493000579685,Ga0172379_10000112:0.06615703223449687):0.025787935977803045[49],(Ga0172379_10002584:0.024182900047202693,Ga0172379_10019035:0.037092514849426994):0.04446669443270945[100]):0.02370017869942398[51],Ga0172379_10000304:0.06032510607730224):0.02265528793845295[52],Ga0172379_10016364:0.10234871250249977):0.03207462821343743[56],(((Ga0172379_10000407:0.04436126052191991,Ga0172379_10005617:0.04852564287826455):0.02132659258432401[92],Ga0172379_10010764:0.04860542828207448):0.04698587271479315[100],Bacteria_CPR_ParcubacteriaOD1_Nomurabacteria_GWB1_OD1_36_6:0.056644620246068644):0.031665050397115646[100]):0.021916656963307357[51],Ga0172380_10004990:0.10055010892393978):0.030442972546461[51],Bacteria_CPR_ParcubacteriaOD1_Nomurabacteria_RAAC4_OD1:0.19835040982589192):0.019998024392438918[62],Ga0172380_10000373:0.12947836615339625):0.034859103487380416[61],Ga0172380_10000393:0.1329350359153123):0.1304847927345656[100],(Bacteria_Parcubacteria_OD1_Nomurabacteria_RIFCSPHIGHO2_02_FULL_OD1_Nomurabacteria_38_15:0.3266663346460006,Bacteria_Parcubacteria_OD1_Nomurabacteria_RIFCSPHIGHO2_02_FULL_OD1_Nomurabacteria_33_12:0.42734249044219785):0.09360901306246516[97]):0.05407754859619951[95]):0.04100220547004918[50],(((Bacteria_CPR_ParcubacteriaOD1_Nomurabacteria_GWB1_OD1_37_5:0.19763234807899321,Bacteria_Parcubacteria_OD1_Nomurabacteria_RIFCSPHIGHO2_01_FULL_OD1_Nomurabacteria_42_16:0.2646270754867821):0.08193342961095418[100],Bacteria_CPR_ParcubacteriaOD1_GWA2_OD1_42_18:0.3861204383161829):0.05813952484419849[67],(Bacteria_Parcubacteria_OD1_Nomurabacteria_RIFCSPLOWO2_01_FULL_OD1_Nomurabacteria_33_24:0.3507966857178406,Ga0172379_10000714:0.23135315312601445):0.052143882153781185[69]):0.03475414654844977[24]):0.03438565275401251[33],Bacteria_Parcubacteria_OD1_Nomurabacteria_RIFCSPHIGHO2_01_FULL_OD1_Nomurabacteria_39_9:0.4671224762447408):0.05055929128044889[75],(((Ga0172379_10000622:0.06065140693023352,Ga0172379_10027313:0.08998459979390905):0.36875479826554525[100],Bacteria_Parcubacteria_OD1_Nomurabacteria_RIFCSPLOWO2_01_FULL_OD1_Nomurabacteria_36_10b:0.5829947471050185):0.06305405423474353[29],(Ga0172379_10029338:0.2996441134988239,Bacteria_Parcubacteria_OD1_RIF_OD1_9_RIFCSPHIGHO2_01_FULL_RIF_OD1_09_41_20:0.2957775529381581):0.07275196138639739[74]):0.04242399242199868[32]):0.04925452520352591[75],((((((((Bacteria_CPR_ParcubacteriaOD1_Nomurabacteria_GWC2_OD1_42_20:0.02764245426650236,Bacteria_Parcubacteria_OD1_Nomurabacteria_CG_Nomura_02:0.05902310907798469):0.021492349198826588[96],Bacteria_Parcubacteria_OD1_RIF_OD1_9_RIFCSPHIGHO2_01_FULL_RIF_OD1_09_44_41:0.031226625478133485):0.05781045658541206[100],Bacteria_Parcubacteria_OD1_Nomurabacteria_CG_Nomura_01:0.0617385745527641):0.06266651205480489[100],Bacteria_Parcubacteria_OD1_RIF_OD1_9_RIFCSPLOWO2_01_FULL_RIF_OD1_09_47_33b:0.09501271412412038):0.03188980754273585[59],Ga0172381_10000001:0.19233950359955454):0.04458593094431729[76],Bacteria_Parcubacteria_OD1_RIF_OD1_9_RIFCSPLOWO2_01_FULL_RIF_OD1_09_43_27:0.1585256147903933):0.210945461690613[100],Bacteria_Parcubacteria_OD1_Nomurabacteria_CG_Nomura_04:0.34167286808780917):0.04364572326842531[36],((Bacteria_Parcubacteria_OD1_RIF_OD1_9_RIFCSPHIGHO2_01_FULL_RIF_OD1_09_51_10:0.1575845597096972,Bacteria_Parcubacteria_OD1_RIF_OD1_9_RIFOXYC1_FULL_RIF_OD1_09_52_10:0.157597186777084):0.16659055724041094[100],Ga0172379_10007670:0.2922153887973713):0.04314126978410249[31]):0.04514541542894879[42]):0.0264694977684643[13]):0.06320393458033147[96],((((((((Ga0172381_10000155:0.06615644419848765,Ga0172379_10000559:0.07161013458623522):0.10002452625850955[100],Ga0172381_10001249:0.15242417291479704):0.11875860000739547[100],Ga0172381_10005223:0.20697132636617832):0.046097386890757264[55],(Ga0172381_10008643:0.06738714657747691,Ga0172382_10005958:0.08856733924162308):0.18324305240710892[100]):0.09745725661519256[100],(((Bacteria_Parcubacteria_OD1_RIFOXYD2_FULL_OD1_44_9:0.04691916375374472,Ga0172379_10000494:0.037209475001515724):0.07045850983416324[100],Bacteria_Parcubacteria_OD1_RIFOXYB1_FULL_OD1_42_16:0.10213684452118743):0.22171497664696238[100],(Ga0172380_10003135:0.1212639755233198,Ga0172379_10000009:0.1532938551985068):0.15358445915480257[100]):0.0454366232541239[74]):0.03281987385305696[51],(((Bacteria_Parcubacteria_OD1_RIFOXYD1_FULL_OD1_44_32:0.19717244873926187,Bacteria_Parcubacteria_OD1_RIFOXYD1_FULL_OD1_46_19:0.1860907588826466):0.1442123629530907[100],Ga0172382_10002939:0.3554296094460079):0.052434840688720286[53],Bacteria_Parcubacteria_OD1_GWA1_OD1_51_14:0.4221255485203339):0.03296771572881152[31]):0.03912269320650763[55],Ga0172379_10001691:0.4166994036706626):0.04648157465441427[91],(Bacteria_Parcubacteria_OD1_RIFCSPHIGHO2_01_FULL_OD1_45_26:0.47120739346298235,Bacteria_Parcubacteria_OD1_RIFOXYD2_FULL_OD1_52_8:0.4000565017224731):0.08887653122230565[80]):0.02384141904343995[70]):0.1476874658673264[100],((Bacteria_CPR_ParcubacteriaOD1_GWB1_OD1_43_8:0.04646506421213381,Bacteria_Parcubacteria_OD1_RIFCSPHIGHO2_02_OD1_41_19:0.04294146955861011):0.2351465432690305[100],Bacteria_CPR_ParcubacteriaOD1_GWC2_OD1_40_31:0.23925440028042466):0.17226135842448542[100]):0.08114314021529667[100],((Bacteria_Parcubacteria_OD1_RIFCSPLOWO2_01_FULL_OD1_42_9:0.35045291279395574,Bacteria_CPR_ParcubacteriaOD1_GWB1_OD1_41_6_partial:0.3438923322042231):0.043233657603679365[81],Bacteria_Parcubacteria_OD1_RIFCSPLOWO2_01_FULL_OD1_39_11:0.40454013162518576):0.04077000010274068[51]):0.02818420312658798[38],(Bacteria_Parcubacteria_OD1_RIFCSPLOWO2_12_FULL_OD1_41_13:0.564044176223859,Bacteria_Parcubacteria_OD1_RIFCSPLOWO2_01_FULL_OD1_45_48:0.51042405940135):0.09173267879512625[67]):0.021419934638029936[27],((((((Bacteria_CPR_ParcubacteriaOD1_Giovannonibacteria_GWA2_OD1_rel_44_26:0.04509681219804751,Bacteria_CPR_ParcubacteriaOD1_Giovannonibacteria_GWA1_OD1_44_29:0.060808204959403866):0.10897792871233003[100],(Bacteria_Parcubacteria_OD1_Giovannonibacteria_RIFCSPLOWO2_01_FULL_OD1_Giovannonibacteria_45_140:0.15078713482193828,Bacteria_Parcubacteria_OD1_Giovannonibacteria_RIFCSPHIGHO2_01_FULL_OD1_Giovannonibacteria_45_23:0.13900311391494036):0.021517693041039454[54]):0.028465650216775717[65],Bacteria_Parcubacteria_OD1_Giovannonibacteria_RIFCSPLOWO2_01_FULL_OD1_Giovannonibacteria_46_32:0.1736027631748147):0.06776985397547763[100],((Bacteria_Parcubacteria_OD1_Giovannonibacteria_RIFCSPLOWO2_12_FULL_OD1_Giovannonibacteria_44_32:0.06108714811429694,Bacteria_Parcubacteria_OD1_Giovannonibacteria_RIFCSPLOWO2_01_FULL_OD1_Giovannonibacteria_44_16:0.05795246919377384):0.12359593049201623[100],Bacteria_Parcubacteria_OD1_Giovannonibacteria_RIFCSPHIGHO2_02_FULL_OD1_Giovannonibacteria_44_11:0.1675995099300649):0.11596613277580037[100]):0.07105161599196874[100],Bacteria_Parcubacteria_OD1_Giovannonibacteria_RIFCSPHIGHO2_02_FULL_OD1_Giovannonibacteria_46_20:0.3746177901424841):0.21351653943947824[100],((((((Bacteria_Parcubacteria_OD1_Giovannonibacteria_RIFCSPHIGHO2_02_OD1_Giovannonibacteria_43_16:0.06280523633905766,Bacteria_Parcubacteria_OD1_Giovannonibacteria_RIFCSPLOWO2_12_FULL_OD1_Giovannonibacteria_43_11c:0.06191003243164683):0.028013760104808494[82],Bacteria_Parcubacteria_OD1_RIF_OD1_2_RIFCSPLOWO2_12_FULL_RIF_OD1_02_44_15:0.05501697561878416):0.13871374150761184[100],Bacteria_Parcubacteria_OD1_Giovannonibacteria_RIFCSPLOWO2_01_FULL_OD1_Giovannonibacteria_46_13:0.16007621097302804):0.08160610191809248[100],Bacteria_Parcubacteria_OD1_RIFOXYD1_FULL_OD1_48_21:0.22384496718273805):0.08412109499119502[100],Bacteria_CPR_ParcubacteriaOD1_Giovannonibacteria_GWA2_OD1_53_7_partial:0.24746116506546612):0.04763469983068358[65],Bacteria_CPR_ParcubacteriaOD1_Giovannonibacteria_GWF2_OD1_42_19:0.2739133605953783):0.14906317718578777[100]):0.05313092401989028[71]):0.04535623812921408[99],((((Bacteria_Parcubacteria_OD1_RIF_OD1_12_RIFCSPHIGHO2_02_FULL_RIF_OD1_12_45_17b:0.05864964464441513,Bacteria_Parcubacteria_OD1_RIF_OD1_12_RIFCSPHIGHO2_02_FULL_RIF_OD1_12_45_13b:0.12042720722673961):0.025056338632339248[49],Bacteria_Parcubacteria_OD1_RIF_OD1_12_RIFCSPLOWO2_02_FULL_RIF_OD1_12_45_11c:0.09232569698192261):0.3144379722746562[100],Bacteria_Parcubacteria_OD1_RIF_OD1_12_RIFCSPHIGHO2_01_FULL_RIF_OD1_12_48_27:0.3197230896577281):0.14973897559161875[100],((Bacteria_Parcubacteria_OD1_RIF_OD1_12_RIFCSPLOWO2_01_FULL_RIF_OD1_12_48_26:0.2961734180536748,Bacteria_CPR_ParcubacteriaOD1_GWA2_OD1_47_10b:0.2639662516797925):0.1734918878999956[100],Bacteria_Parcubacteria_OD1_RIF_OD1_12_RIFCSPLOWO2_01_FULL_RIF_OD1_12_44_230:0.46581503007919833):0.115601692046849[100]):0.0500505196278489[97]):0.05151566115548878[99],(((((Bacteria_Parcubacteria_OD1_RIF_OD1_13_RIFCSPHIGHO2_02_FULL_RIF_OD1_13_49_12:0.2117349675565423,Bacteria_CPR_ParcubacteriaOD1_GWC2_OD1_52_8_partial:0.18850764870343228):0.05315348683492438[61],Bacteria_Parcubacteria_OD1_RIF_OD1_13_RIFCSPHIGHO2_02_FULL_RIF_OD1_13_49_20:0.19172173794684122):0.2535927595642442[100],(Bacteria_Parcubacteria_OD1_RIF_OD1_13_RIFCSPLOWO2_01_FULL_RIF_OD1_13_59_16:0.27008967384337,Bacteria_Parcubacteria_OD1_RIF_OD1_13_RIFCSPLOWO2_01_FULL_RIF_OD1_13_60_25:0.24888762853474544):0.17394256456750812[100]):0.04767929381491953[65],((Bacteria_Parcubacteria_OD1_RIF_OD1_13_RIFCSPLOWO2_01_FULL_RIF_OD1_13_47_10:0.1726607137763314,Bacteria_Parcubacteria_OD1_RIF_OD1_13_RIFCSPLOWO2_12_FULL_RIF_OD1_13_41_11:0.14594910075665934):0.11189715070003103[100],Bacteria_Parcubacteria_OD1_RIF_OD1_13_RIFCSPLOWO2_02_FULL_RIF_OD1_13_51_17:0.29498368581601797):0.11985933699124329[100]):0.03490342244173972[35],((((Bacteria_Parcubacteria_OD1_RIF_OD1_13_RIFCSPHIGHO2_02_FULL_RIF_OD1_13_53_17:0.24304502330759803,Bacteria_Parcubacteria_OD1_RIF_OD1_13_RIFCSPHIGHO2_02_FULL_RIF_OD1_13_52_23:0.2456123188554158):0.09863834458147158[100],Bacteria_Parcubacteria_OD1_RIF_OD1_13_RIFCSPHIGHO2_02_FULL_RIF_OD1_13_47_11:0.287033890941077):0.03582154947680127[36],(Bacteria_Parcubacteria_OD1_RIF_OD1_13_RIFCSPHIGHO2_01_FULL_RIF_OD1_13_50_25:0.16338183462089795,Bacteria_Parcubacteria_OD1_RIF_OD1_13_GWC2_RIF_OD1_13_49_10:0.15010808985817548):0.18303883774616203[100]):0.028276270100207945[31],Bacteria_CPR_ParcubacteriaOD1_GWA2_OD1_45_30:0.2879009867659881):0.10318553668288111[100]):0.1522880334977641[100]):0.07001175966607098[100],((Bacteria_Parcubacteria_OD1_RIFCSPLOWO2_01_FULL_OD1_58_14:0.45252426746097285,Bacteria_Parcubacteria_OD1_RIFCSPLOWO2_01_FULL_OD1_48_14:0.45273828326044097):0.16944021876867765[100],(Bacteria_Parcubacteria_OD1_Falkowbacteria_RIFCSPLOWO2_01_FULL_OD1_Falkowbacteria_40_23:0.18732027548988173,Bacteria_Parcubacteria_OD1_RIFCSPLOWO2_01_FULL_OD1_44_24:0.19004876837590956):0.31622230984799904[100]):0.14420573587609242[100]):0.03939051044634656[17],((((((((((((((Ga0172380_10003010:0.1503840321462091,Ga0172381_10025343:0.14696820110754993):0.18565421495856382[100],Bacteria_Parcubacteria_OD1_Wolfebacteria_RIFCSPLOWO2_01_FULL_OD1_Wolfebacteria_47_17b:0.37220628008952295):0.07231987770627857[84],Ga0172379_10004224:0.39114867757390925):0.03724951088837214[62],(Bacteria_Parcubacteria_OD1_Wolfebacteria_GWA1_OD1_Wolfebacteria_42_9:0.270189503124143,Ga0172379_10000945:0.25684489771505525):0.08091261325600341[100]):0.16267600728471265[100],((Bacteria_CPR_ParcubacteriaOD1_Wolfebacteria_GWA2_OD1_ACD81_47_9:0.049249106431664025,Bacteria_Parcubacteria_OD1_Wolfebacteria_RIFOXYB1_FULL_OD1_Wolfebacteria_54_12:0.029861649386408562):0.025583693292153153[68],Bacteria_CPR_ParcubacteriaOD1_Wolfebacteria_GWE2_OD1_ACD81_44_13:0.046956896876758414):0.35128041720490133[100]):0.04708842034108063[83],((Bacteria_CPR_ParcubacteriaOD1_Wolfebacteria_GWB1_OD1_41_12:0.20092777920219396,Bacteria_Parcubacteria_OD1_Wolfebacteria_RIFCSPLOWO2_01_FULL_OD1_Wolfebacteria_38_11:0.21475809001138124):0.054123696724211356[100],(Bacteria_Parcubacteria_OD1_Wolfebacteria_RBG_13_OD1_Wolfebacteria_41_7:0.27204573608177096,Bacteria_Parcubacteria_OD1_Wolfebacteria_CG_Wolfe_01:0.16275970448269295):0.08150841317174606[100]):0.07700692012961996[100]):0.06728954362850947[100],((((Bacteria_CPR_ParcubacteriaOD1_GWA1_OD1_47_11:0.305656300969269,Bacteria_Parcubacteria_OD1_RBG_13_OD1_48_8:0.2742532760604033):0.13848698374404078[100],Bacteria_Parcubacteria_OD1_RIFCSPLOWO2_02_FULL_OD1_45_11:0.3397707445555769):0.10183281779631592[100],((Bacteria_Parcubacteria_OD1_RIFCSPHIGHO2_12_FULL_OD1_43_12:0.20126464992837434,Bacteria_Parcubacteria_OD1_CG_CPR_07_CG_CPR07_01:0.18329586206276938):0.13872584804221777[100],Bacteria_Parcubacteria_OD1_RIFCSPLOWO2_01_FULL_OD1_48_10:0.36042152564834673):0.05419851548085308[78]):0.07314174926442796[100],(((((Ga0172381_10001153:0.000001,Ga0172377_10000730:0.000001):0.000439[65],Ga0172382_10002208:0.000001):0.14297240291570157[100],Ga0172381_10004607:0.12412133888718246):0.1155529027726474[100],Ga0172381_10006470:0.19840287160527126):0.21943977645133295[100],Bacteria_Parcubacteria_OD1_RIFCSPHIGHO2_12_FULL_OD1_44_17:0.4071652152573795):0.046655992989504735[58]):0.07888046441314644[100]):0.022332475354087222[12],((((Ga0172379_10021708:0.030999394582165163,Ga0172381_10008414:0.04810695585526714):0.3555689436821172[100],(Bacteria_Parcubacteria_OD1_Wolfebacteria_RIFCSPHIGHO2_02_FULL_OD1_Wolfebacteria_42_16:0.2659056389726171,Bacteria_Parcubacteria_OD1_Wolfebacteria_RIFCSPLOWO2_02_FULL_OD1_Wolfebacteria_45_10c:0.2753592874141586):0.13371043810157124[100]):0.10306220614481498[100],((Bacteria_Parcubacteria_OD1_Wolfebacteria_RIFCSPHIGHO2_12_FULL_OD1_Wolfebacteria_48_16:0.2836041705138346,Bacteria_Parcubacteria_OD1_Wolfebacteria_RIFCSPLOWO2_01_FULL_OD1_Wolfebacteria_44_18:0.22399569888851412):0.05301100959584959[71],Bacteria_Parcubacteria_OD1_Wolfebacteria_RIFCSPLOWO2_02_FULL_OD1_Wolfebacteria_41_13b:0.3262793020042545):0.06152748741093328[100]):0.06202961225837944[100],(Bacteria_CPR_ParcubacteriaOD1_GWB1_OD1_45_9:0.3474000386758731,Bacteria_Parcubacteria_OD1_RIFCSPLOWO2_01_FULL_OD1_40_28:0.4806801039234836):0.05848431573572377[26]):0.027081080780104294[13]):0.0312467390911082[15],((((((Bacteria_Parcubacteria_OD1_RIFCSPLOWO2_01_FULL_OD1_56_20:0.2640148737703081,Bacteria_CPR_ParcubacteriaOD1_GWA1_OD1_59_11:0.23320740446913213):0.060222230565949886[97],Bacteria_Parcubacteria_OD1_RIFCSPLOWO2_01_FULL_OD1_53_13:0.3493517727397655):0.05204815023210463[74],(Bacteria_Parcubacteria_OD1_GWB1_OD1_49_6:0.30727314867874256,Bacteria_Parcubacteria_OD1_RIFCSPHIGHO2_01_FULL_OD1_57_28:0.3586395875511319):0.057054662772407116[56]):0.026217359899439963[15],((Ga0172379_10038906:0.22084402425666605,Ga0172379_10000052:0.22928127120917718):0.10355933306185339[100],Bacteria_Parcubacteria_OD1_RIFCSPLOWO2_01_FULL_OD1_45_15:0.30936679046349935):0.0567150997791539[43]):0.0437668752049003[51],Bacteria_Parcubacteria_OD1_RIFCSPLOWO2_01_FULL_OD1_52_25:0.3163931448923125):0.15542024568869328[100],(Bacteria_Parcubacteria_OD1_RIFCSPLOWO2_12_FULL_OD1_60_15:0.38327181434559865,Bacteria_Parcubacteria_OD1_RIFOXYD1_FULL_OD1_36_11:0.4880079187678059):0.049403117498035165[57]):0.04261135050305409[54]):0.03094783862094852[47],((((((((((Ga0172379_10002192:0.018481708254033613,Ga0172379_10001176:0.03368860207145952):0.12549612700589297[100],Ga0172379_10026556:0.131067360743784):0.05237894939101384[99],Ga0172379_10041360:0.17588204969712384):0.13547995724720963[100],Ga0172379_10004358:0.39432160926188775):0.06330132593109239[95],(Ga0172379_10030090:0.19331373015399667,Ga0172379_10000518:0.20303395730655094):0.16114446385540582[100]):0.06484423426233565[97],((Bacteria_Parcubacteria_OD1_Jorgensenbacteria_RIFCSPHIGHO2_02_FULL_OD1_Jorgensenbacteria_45_20:0.34721747834336547,Bacteria_CPR_ParcubacteriaOD1_Jorgensenbacteria_GWA2_OD1_45_13:0.26443313764697196):0.11818395924976333[100],Bacteria_CPR_ParcubacteriaOD1_Jorgensenbacteria_GWB1_OD1_49_9:0.3093795447807297):0.043946982982188576[45]):0.03293816335470545[72],(Bacteria_CPR_ParcubacteriaOD1_Jorgensenbacteria_GWB1_OD1_50_10:0.29594544122684097,Bacteria_Parcubacteria_OD1_GWA1_OD1_49_17:0.27736189569557324):0.09428756275297179[100]):0.0552864179538739[93],Bacteria_Parcubacteria_OD1_Jorgensenbacteria_RIFCSPLOWO2_01_FULL_OD1_Jorgensenbacteria_45_25b:0.45328785231472724):0.04091998988580192[79],Bacteria_Parcubacteria_OD1_Jorgensenbacteria_GWA1_OD1_Jorgensenbacteria_54_12:0.7173777604590863):0.051235222499972366[99],Bacteria_Parcubacteria_OD1_Jorgensenbacteria_RIFCSPLOWO2_12_FULL_OD1_Jorgensenbacteria_42_11:0.4191880653956259):0.06105149630840012[100]):0.10064114312886785[100],Bacteria_Parcubacteria_OD1_RIFCSPLOWO2_01_FULL_OD1_48_18:0.5110546479310392):0.06077211745885469[97],((Ga0172380_10018290:0.01712190427873672,Ga0172381_10031147:0.01326466182009689):0.0748549808425949[100],Ga0172381_10007172:0.0991359189505423):0.454983577624128[100]):0.04560012848485506[91],((((((((Ga0172377_10038211:0.000933,Ga0172382_10008291:0.000001):0.000001[69],Ga0172381_10011584:0.000001):0.000881[70],Ga0172380_10003322:0.001129250765627976):0.1734160825775155[100],Ga0172382_10015681:0.14469626839355554):0.16688638651789933[100],Ga0172380_10004595:0.28880842315929023):0.0664783523971142[96],Ga0172377_10038339:0.40800569485382265):0.14514985808547776[100],(Ga0172380_10023349:0.11221984622694059,Ga0172379_10022705:0.10176332236745367):0.5774368304342334[100]):0.11450070333953688[100],((Bacteria_Parcubacteria_OD1_CG_CPR_08_CG_CPR08_01:0.45256912886710676,Bacteria_CPR_ParcubacteriaOD1_GWB1_OD1_rel_46_8:0.4560595776298726):0.08467213490456205[88],(Bacteria_Parcubacteria_OD1_RIFOXYD1_FULL_OD1_41_16:0.2177011562770481,Bacteria_CPR_ParcubacteriaOD1_GWB1_OD1_rel_45_10:0.18777698856692515):0.23929439414340203[100]):0.24471576153399432[100]):0.07036268632089904[99]):0.048215825134521495[98],(((((Bacteria_CPR_ParcubacteriaOD1_Azambacteria_GWA2_OD1_39_10_partial:0.05261898434821477,Bacteria_Parcubacteria_OD1_Azambacteria_RIFCSPHIGHO2_01_FULL_OD1_Azambacteria_40_24:0.051371303118860734):0.12630868839436227[100],Bacteria_CPR_ParcubacteriaOD1_Azambacteria_GWE1_OD1:0.24000391604256022):0.02905404960827207[49],Bacteria_CPR_ParcubacteriaOD1_Azambacteria_GWA1_OD1_42_19_partial:0.16481000615034658):0.11865564920666172[100],Bacteria_Parcubacteria_OD1_Azambacteria_RIFCSPLOWO2_02_FULL_OD1_Azambacteria_44_14:0.27943646653429743):0.20488261016192055[100],(Ga0172379_10002233:0.25161093290610825,Bacteria_CPR_ParcubacteriaOD1_L1_Parcubacteria_bacterium_SCGC_AAA011_A09_DUSEL_001_189:0.30438290428134307):0.36752013005239537[100]):0.05713389169379868[34]):0.03655032663214319[26]):0.029584175508226895[22],(((((((((((((Ga0172379_10038390:0.06537835986673457,Bacteria_Parcubacteria_OD1_Yanofskybacteria_RIFCSPHIGHO2_01_FULL_OD1_Yanofskybacteria_41_21:0.05390456278941347):0.1036192781011036[100],Bacteria_Parcubacteria_OD1_Yanofskybacteria_RIFCSPHIGHO2_01_FULL_OD1_Yanofskybacteria_48_25b:0.1612173491382829):0.10234864090198226[100],((Bacteria_Parcubacteria_OD1_Yanofskybacteria_RIFCSPLOWO2_01_FULL_OD1_Yanofskybacteria_49_17:0.1215341083581194,Bacteria_Parcubacteria_OD1_Yanofskybacteria_RIFCSPLOWO2_02_FULL_OD1_Yanofskybacteria_47_9b:0.11963240910659367):0.10298564417190148[100],Bacteria_Parcubacteria_OD1_Yanofskybacteria_RIFCSPHIGHO2_02_FULL_OD1_Yanofskybacteria_50_12:0.2194586267864631):0.03689060579936676[69]):0.13391769099724327[100],(((Ga0172379_10001397:0.07311160214410561,Bacteria_CPR_ParcubacteriaOD1_Yanofskybacteria_GWA1_OD1_39_13_partial:0.09133951151984254):0.017350191606304577[62],Bacteria_Parcubacteria_OD1_Yanofskybacteria_RIFCSPHIGHO2_01_FULL_OD1_Yanofskybacteria_43_32:0.050314959705702655):0.029871695733057813[98],Bacteria_Parcubacteria_OD1_Yanofskybacteria_RIFCSPLOWO2_01_FULL_OD1_Yanofskybacteria_41_34:0.08778982853903106):0.1502070071952688[100]):0.03310477238330556[49],(Bacteria_Parcubacteria_OD1_Yanofskybacteria_RIFCSPHIGHO2_01_FULL_OD1_Yanofskybacteria_44_22:0.19750589947177755,Bacteria_CPR_ParcubacteriaOD1_Yanofskybacteria_GWC2_OD1_41_9:0.15899524451409652):0.09348426275693633[100]):0.03449678608743412[57],(((((Bacteria_Parcubacteria_OD1_Yanofskybacteria_RIFCSPHIGHO2_02_FULL_OD1_Yanofskybacteria_41_29:0.14597655681531974,Bacteria_Parcubacteria_OD1_Yanofskybacteria_RIFCSPHIGHO2_02_FULL_OD1_Yanofskybacteria_39_10:0.18148072232266488):0.03275589040641114[49],Bacteria_Parcubacteria_OD1_Yanofskybacteria_RIFCSPHIGHO2_01_FULL_OD1_Yanofskybacteria_39_8b:0.12702721370159198):0.02752508035114687[35],Bacteria_Parcubacteria_OD1_Yanofskybacteria_RIFCSPHIGHO2_02_FULL_OD1_Yanofskybacteria_38_22b:0.1634627260362178):0.0854793516992256[100],Bacteria_Parcubacteria_OD1_Yanofskybacteria_RIFCSPHIGHO2_01_FULL_OD1_Yanofskybacteria_44_24:0.2188934804048861):0.08225147376066788[100],((Bacteria_Parcubacteria_OD1_Yanofskybacteria_RIFCSPHIGHO2_02_FULL_OD1_Yanofskybacteria_43_22:0.11247987112692037,Bacteria_Parcubacteria_OD1_Yanofskybacteria_RIFCSPHIGHO2_01_FULL_OD1_Yanofskybacteria_43_42:0.1040410635213731):0.03950767438373237[85],Bacteria_Parcubacteria_OD1_Yanofskybacteria_RIFCSPLOWO2_01_FULL_OD1_Yanofskybacteria_42_49:0.13527666641039637):0.13720895047005177[100]):0.02666242484028203[31]):0.028945203455980728[52],(((Ga0172379_10002198:0.07148860403201063,Bacteria_CPR_ParcubacteriaOD1_Yanofskybacteria_GWD1_OD1_39_16_partial:0.047133988667594995):0.140545031689145[100],Bacteria_CPR_ParcubacteriaOD1_Yanofskybacteria_GWF1_OD1_44_227:0.18871186397648065):0.09621923085138429[100],Bacteria_Parcubacteria_OD1_Yanofskybacteria_RIFCSPLOWO2_01_FULL_OD1_Yanofskybacteria_44_88:0.28371965344641614):0.06597628188409876[100]):0.019561526664172213[38],Bacteria_Parcubacteria_OD1_Yanofskybacteria_RIFCSPHIGHO2_02_FULL_OD1_Yanofskybacteria_41_11:0.25139473184198025):0.03722237546958551[69],Bacteria_Parcubacteria_OD1_Yanofskybacteria_RIFCSPLOWO2_02_FULL_OD1_Yanofskybacteria_45_18:0.3240677890811057):0.11394754679897146[100],((Bacteria_Parcubacteria_OD1_Yanofskybacteria_RIFCSPLOWO2_02_FULL_OD1_Yanofskybacteria_43_10b:0.22669298537420124,Bacteria_Parcubacteria_OD1_Yanofskybacteria_RIFCSPHIGHO2_12_FULL_OD1_Yanofskybacteria_45_19b:0.3545737206455568):0.14425811146431844[100],(Bacteria_Parcubacteria_OD1_Yanofskybacteria_RIFCSPLOWO2_01_FULL_OD1_Yanofskybacteria_49_25:0.3449367765642388,Bacteria_Parcubacteria_OD1_Yanofskybacteria_RIFCSPHIGHO2_01_FULL_OD1_Yanofskybacteria_44_17:0.23636158486808512):0.11710294711915115[100]):0.028338296915670913[45]):0.06110929159241296[93],Bacteria_Parcubacteria_OD1_RIFCSPHIGHO2_01_FULL_OD1_40_30:0.42124269661655367):0.13949732460617126[100],Bacteria_CPR_ParcubacteriaOD1_GWA2_OD1_rel_38_13:0.551244488692908):0.041042231881220914[23],((((Bacteria_Parcubacteria_OD1_RIFCSPLOWO2_01_FULL_OD1_46_25:0.2928016551925796,Bacteria_Parcubacteria_OD1_RBG_16_OD1_47_10:0.26841861058093563):0.1919775839868607[100],Bacteria_CPR_ParcubacteriaOD1_GWD1_OD1_38_16:0.4043604688727651):0.04001914448869881[22],Ga0172379_10001349:0.5435000386974278):0.04361610197864918[26],(Bacteria_CPR_ParcubacteriaOD1_GWA2_OD1_rel_39_18:0.5868533922445756,Bacteria_CPR_ParcubacteriaOD1_Azambacteria_GWD2_OD1_46_48:0.36171148170149703):0.06481065363952787[25]):0.04048258737775967[47]):0.022165418648714574[4]):0.029115631348289206[31],(((((((((((((((((((Bacteria_Parcubacteria_OD1_RIF_OD1_6_RIFOXYC1_FULL_RIF_OD1_06_38_18:0.09849171548835223,Ga0172379_10006715:0.11163772033140162):0.06565086377650076[100],(Ga0172381_10022300:0.09156682967633589,Ga0172381_10013793:0.09618329394100034):0.031164601309102924[90]):0.010216006629029994[41],(Ga0172379_10000676:0.06485226714995607,Bacteria_Parcubacteria_OD1_RIF_OD1_6_RIFOXYB1_FULL_RIF_OD1_06_37_44:0.06683148278560624):0.04985138320883831[100]):0.022869693823201143[88],((Ga0172379_10007576:0.09126689872736149,Ga0172380_10006143:0.16937937822924853):0.014948618311029449[22],Ga0172379_10029276:0.12276050989992848):0.023496506437162168[84]):0.0162979620107091[42],Ga0172380_10020722:0.10195040312205439):0.026466739667257322[82],((Ga0172381_10001530:0.12199005916657901,Ga0172381_10030851:0.15448978777538436):0.03193362469169525[94],(Ga0172381_10001241:0.027725918728606747,Ga0172380_10010234:0.055840365594695296):0.09995095285808553[100]):0.0212339398879835[81]):0.02847710309806306[96],((Ga0172381_10024713:0.10664766112748181,Ga0172381_10034376:0.18046994468149657):0.013831946198445522[54],(Ga0172381_10018726:0.056082351717285484,Ga0172381_10029588:0.08432063471080342):0.06840055028180947[100]):0.05425851157397377[99]):0.031331907702841555[88],(Bacteria_Parcubacteria_OD1_RIF_OD1_6_RIFCSPLOWO2_01_FULL_RIF_OD1_06_37_25b:0.1051025293016794,Bacteria_Parcubacteria_OD1_RIF_OD1_6_RIFOXYB1_FULL_RIF_OD1_06_38_37:0.11461564517271672):0.0756455599676209[100]):0.0458100809696389[99],((Bacteria_Parcubacteria_OD1_RIF_OD1_6_RIFOXYD1_FULL_RIF_OD1_06_32_13:0.2648029010006754,Ga0172379_10025364:0.13075366044375203):0.031130314220873423[28],Bacteria_CPR_ParcubacteriaOD1_GWA2_OD1_37_10:0.15828507658634683):0.036328811674373185[44]):0.024647887987605976[42],(((Ga0172380_10011352:0.0751783166834139,Bacteria_Parcubacteria_OD1_RIF_OD1_6_RIFCSPLOWO2_01_FULL_RIF_OD1_06_33_9:0.10563711203782589):0.02400233678125341[79],Ga0172379_10019406:0.07632072239383092):0.10847741332241156[100],Ga0172379_10003383:0.15914296348868007):0.04541823400932943[85]):0.025444854032888653[69],(((((Bacteria_Parcubacteria_OD1_RIF_OD1_6_RIFCSPLOWO2_01_FULL_RIF_OD1_06_38_12b:0.10793385303785108,Bacteria_Parcubacteria_OD1_RIF_OD1_6_RIFCSPHIGHO2_02_FULL_RIF_OD1_06_42_22:0.11814583414208624):0.0861150833320794[100],Bacteria_Parcubacteria_OD1_RIF_OD1_6_RIFCSPLOWO2_01_FULL_RIF_OD1_06_33_13:0.15210471375627233):0.02676287698050972[64],(Bacteria_Parcubacteria_OD1_RIF_OD1_6_RIFCSPHIGHO2_01_FULL_RIF_OD1_06_39_25:0.12159964633023135,Bacteria_Parcubacteria_OD1_RIF_OD1_6_RIFCSPLOWO2_01_FULL_RIF_OD1_06_43_17b:0.18957174516226472):0.0340621502228986[51]):0.013543359471825944[25],(Bacteria_Parcubacteria_OD1_RIF_OD1_6_RIFCSPHIGHO2_12_FULL_RIF_OD1_06_38_11:0.12451102860367058,Bacteria_Parcubacteria_OD1_RIF_OD1_6_RIFCSPHIGHO2_02_FULL_RIF_OD1_06_34_10:0.14523364691793006):0.03756688644066486[65]):0.038283758564349846[93],Bacteria_Parcubacteria_OD1_RIF_OD1_6_RIFCSPHIGHO2_02_FULL_RIF_OD1_06_34_9:0.23716907657868802):0.07971311385661073[100]):0.08947961980236485[100],(Ga0172380_10003391:0.21332089680611288,Ga0172379_10000016:0.2562015772551969):0.10569094519219435[100]):0.12393348896385348[100],((Bacteria_Parcubacteria_OD1_CG_CPR_03_CG_CPR03_01:0.39108793752941384,Bacteria_Parcubacteria_OD1_CG_CPR_03_CG_CPR03_02:0.38121177852208676):0.14698478261988823[100],Bacteria_Parcubacteria_OD1_CG_CPR_03_CG_CPR03_03:0.2758977376189895):0.08027918987638039[100]):0.0609140297455415[94],(((((((((Bacteria_OD1_uncultured_DG_74_3:0.2680735458795267,Bacteria_Parcubacteria_OD1_CG_CPR_10_CG_CPR10_02:0.2817995612908102):0.039225879828799215[51],(Bacteria_OD1_uncultured_DG_74_1:0.23730523961065497,Bacteria_Parcubacteria_OD1_CG_CPR_10_CG_CPR10_01:0.15919036069258174):0.02723003203188501[39]):0.019239309579175412[42],Bacteria_Parcubacteria_OD1_RIF_OD1_7_RIFCSPLOWO2_01_FULL_RIF_OD1_07_41_9:0.3728010363180738):0.021819809469025397[37],(Bacteria_CPR_ParcubacteriaOD1_L1_Parcubacteria_bacterium_SCGC_AAA255_P19_SAK_001_138:0.19184417416262312,Bacteria_Parcubacteria_OD1_CG_CPR_10_CG_CPR10_03:0.15285140722902124):0.07807133575721625[100]):0.03738632007611553[44],((((Ga0172377_10007593:0.008448968797043044,Ga0172381_10034926:0.000217):0.25898526784464115[100],Bacteria_Parcubacteria_OD1_RIF_OD1_7_RBG_13_RIF_OD1_07_38_11:0.20820381861918458):0.039147321194737295[91],Ga0172381_10008805:0.42467222317051556):0.04095760502246515[65],Bacteria_Parcubacteria_OD1_RIF_OD1_7_RBG_13_RIF_OD1_07_42_11:0.2761772476863169):0.052743837727169396[100]):0.03522422140170445[44],((Bacteria_Parcubacteria_OD1_RIF_OD1_7_RIFCSPHIGHO2_12_FULL_RIF_OD1_07_40_12:0.2111312644551,Bacteria_CPR_ParcubacteriaOD1_GWA1_OD1_36_12:0.21988581191087686):0.16233336349376692[100],Ga0172381_10017300:0.29957033149643575):0.03558641313727984[8]):0.026117307244663657[9],((((((Ga0172380_10008630:0.001693454684847051,Ga0172377_10006933:0.000001):0.000001[51],Ga0172381_10002577:0.000558):0.000716[67],Ga0172382_10006397:0.000001):0.10968201415220591[100],Ga0172381_10022228:0.1596961713253462):0.1844898117869218[100],((Bacteria_SM2F11_RIFCSPHIGHO2_02_FULL_SM2F11_43_13:0.08687553501688727,Bacteria_CPR_ParcubacteriaOD1_GWF2_OD1_rel_43_11_partial:0.09747483207468033):0.21104304849350752[100],Bacteria_Parcubacteria_OD1_RIF_OD1_7_RIFCSPLOWO2_01_FULL_RIF_OD107_43_32:0.2332454609512582):0.054351561771898815[99]):0.04119286740131445[99],Bacteria_Parcubacteria_OD1_RIF_OD1_7_RIFCSPHIGHO2_01_FULL_RIF_OD1_07_47_27:0.3261020842207287):0.03962503462435274[59]):0.02188665874255813[6],(Bacteria_Parcubacteria_OD1_RIF_OD1_7_RBG_13_RIF_OD1_07_36_15:0.24739043351637902,Bacteria_Parcubacteria_OD1_RIF_OD1_7_GWA2_RIF_OD1_07_46_15:0.4341667246408516):0.0557978295032866[20]):0.01581577115628008[17],((((Bacteria_OD1_uncultured_DG_72:0.21136155202809848,Bacteria_Parcubacteria_OD1_RIF_OD1_7_RIFOXYB1_FULL_RIF_OD1_07_40_15:0.3280828148543078):0.05592442037268208[92],Bacteria_Parcubacteria_OD1_RIF_OD1_7_RBG_13_RIF_OD1_07_37_56:0.25368437324445825):0.05947139913181543[97],Bacteria_CPR_ParcubacteriaOD1_GWA2_OD1_38_27:0.3300214942287236):0.03728273812041083[83],(Ga0172382_10005619:0.000001,Ga0172377_10000952:0.000827):0.4519512056667905[100]):0.049528391605353494[41]):0.02060353394893344[11]):0.03412260162659475[13],((((((((((Ga0172378_10019307:0.000001,Ga0172377_10021010:0.000001):0.028470694916727268[100],Ga0172380_10049579:0.01796104860159664):0.053329884087165436[100],Ga0172381_10002889:0.07858866174253754):0.0698728046449455[100],((Ga0172381_10027718:0.000001,Ga0172377_10005986:0.000001):0.044007950611866686[100],Ga0172382_10055737:0.03243428395653636):0.054809221799686725[100]):0.03946766575503036[100],(Ga0172381_10049690:0.060542499273068096,Ga0172377_10001677:0.043233715031819386):0.037304977323793675[99]):0.07020791107762969[100],(((Ga0172380_10026643:0.03157917861608439,Ga0172381_10010745:0.045155739406963935):0.060391683545034436[100],Ga0172381_10001390:0.059194456939683615):0.050670662067902494[100],Ga0172381_10005393:0.12329599512848999):0.05212376900462212[100]):0.11591314854521917[100],(((Ga0172381_10007167:0.027149566841513373,Ga0172381_10000133:0.05889517021572788):0.024370951841730992[83],Ga0172381_10000050:0.0560807083755579):0.19805826320870645[100],(Ga0172380_10000194:0.000001,Ga0172381_10000044:0.000001):0.2613165509297941[100]):0.056314682728881404[48]):0.08382835894276353[100],(Ga0172377_10055013:0.1420700875845311,Ga0172382_10030184:0.10997924847174323):0.2553825744026148[100]):0.0809738262758084[100],(((Ga0172381_10006557:0.10344277409676339,Ga0172380_10001694:0.137114528379628):0.05092274390022[79],Ga0172381_10000589:0.08981181343801525):0.05157975686349481[58],((Ga0172381_10000392:0.000001,Ga0172377_10043143:0.000001):0.0015161782034893712[90],Ga0172382_10021415:0.0019491549969825606):0.12083540081892785[100]):0.25456397515498885[100]):0.10759611598899754[100],((((Ga0172381_10000725:0.17300827122360918,Ga0172379_10001900:0.13672133214574522):0.025952299179190064[62],Ga0172381_10022019:0.11800446840650025):0.030538867879244425[88],((Ga0172380_10006501:0.008653720424713907,Ga0172377_10038217:0.010847080798154884):0.067604383875294[100],Ga0172381_10000097:0.08330583990755214):0.041003417323716373[99]):0.08609078595064412[98],((Ga0172380_10036434:0.004495197498223913,Ga0172382_10013448:0.0028084619350878803):0.1555804357297621[100],Ga0172382_10019877:0.14765087354963446):0.034523170148778703[49]):0.3570922369571785[100]):0.04635086033489744[52]):0.018736760893727666[3],((Ga0172382_10047813:0.000001,Ga0172378_10011436:0.000001):0.6226021803638688[100],(Ga0172377_10000595:0.000001,Ga0172378_10000061:0.000001):0.40278889752753466[100]):0.09708356513961824[54]):0.02016702918443647[19],(((Bacteria_Parcubacteria_OD1_RIF_OD1_7_RIFCSPHIGHO2_01_FULL_RIF_OD1_07_49_22b:0.38252761113899103,Bacteria_Parcubacteria_OD1_RIF_OD1_7_RIFCSPHIGHO2_02_FULL_RIF_OD1_07_47_12:0.38903103899918534):0.05488107255447172[84],(Bacteria_Parcubacteria_OD1_RIF_OD1_7_RIFCSPHIGHO2_02_FULL_RIF_OD1_07_48_16:0.17965623025780264,Bacteria_Parcubacteria_OD1_RIF_OD1_7_RIFCSPLOWO2_01_FULL_RIF_OD1_07_48_16:0.1867513169874826):0.2927827496236879[100]):0.05091053191698425[67],((Bacteria_CPR_ParcubacteriaOD1_GWB1_OD1_49_12:0.18983969964931413,Bacteria_Parcubacteria_OD1_RIF_OD1_7_RIFCSPHIGHO2_01_FULL_RIF_OD1_07_48_27b:0.15529233362542216):0.17211333204600932[100],Bacteria_Parcubacteria_OD1_RIF_OD1_7_RIFCSPHIGHO2_02_FULL_RIF_OD1_07_45_25:0.32476237636585203):0.08655021617293102[100]):0.11411054733643855[100]):0.09649350339930951[100],((((Bacteria_Parcubacteria_OD1_RIF_OD1_5_RIFCSPLOWO2_02_FULL_RIF_OD1_05_38_8:0.24415809187337834,Bacteria_Parcubacteria_OD1_RIF_OD1_5_RIFCSPLOWO2_12_FULL_RIF_OD1_05_38_22:0.2593679441454375):0.10658730508796577[100],Bacteria_Parcubacteria_OD1_RIF_OD1_5_RIFCSPLOWO2_01_FULL_RIF_OD1_05_43_12:0.3319076255984399):0.07064259219260727[96],(Ga0172380_10000345:0.4015909318739048,Bacteria_Parcubacteria_OD1_RIF_OD1_5_RIFCSPLOWO2_01_FULL_RIF_OD1_05_46_10:0.3644801367977175):0.08156722617750667[60]):0.21737176083707554[100],Bacteria_Parcubacteria_OD1_RIF_OD1_5_RIFCSPHIGHO2_02_FULL_RIF_OD1_05_43_15b:0.45696553672692186):0.12868514373982842[99]):0.04360127637106137[40],(((((((Ga0172379_10005099:0.20620278591453456,Bacteria_CPR_ParcubacteriaOD1_GWB1_OD1_42_6:0.24295039036149735):0.056549747801241335[99],Bacteria_Parcubacteria_OD1_RIF_OD1_8_RIFCSPLOWO2_01_FULL_RIF_OD1_08_43_11:0.28848245313973875):0.04397814913070697[91],Bacteria_CPR_ParcubacteriaOD1_GWF2_OD1_rel_44_7_partial:0.2893360677814232):0.10158538628599567[100],((Bacteria_Parcubacteria_OD1_RIF_OD1_8_RBG_13_RIF_OD1_08_41_18:0.13716734486963844,Bacteria_Parcubacteria_OD1_RIF_OD1_8_RBG_13_RIF_OD1_08_40_8:0.18120054053587387):0.03690657004582221[97],Bacteria_Parcubacteria_OD1_CG_CPR_04_CG_CPR04_01:0.09937344246915147):0.19288925404488566[100]):0.07532008881847885[100],(Bacteria_CPR_ParcubacteriaOD1_GWF2_OD1_rel_39_13_partial:0.19876622086489482,Bacteria_Parcubacteria_OD1_CG_CPR_05_CG_CPR05_02:0.27019560410541565):0.12175997876934197[100]):0.054566964953921104[96],(((Bacteria_Parcubacteria_OD1_RIF_OD1_8_RIFCSPLOWO2_01_FULL_RIF_OD1_08_38_39:0.12841077515314492,Bacteria_Parcubacteria_OD1_RIF_OD1_8_RIFCSPHIGHO2_01_FULL_RIF_OD1_08_40_12b:0.1610688767343329):0.09032731846232078[100],Bacteria_Parcubacteria_OD1_CG_CPR_01_CG_CPR01_01:0.35541594205711124):0.06984308957871699[99],Bacteria_Parcubacteria_OD1_CG_CPR_05_CG_CPR05_01:0.5132697455048363):0.03955696473801762[84]):0.05517055782136726[92],(Bacteria_Parcubacteria_OD1_RIF_OD1_8_RBG_13_RIF_OD1_08_37_53:0.2897626705236913,Ga0172379_10001204:0.2610881235532254):0.19272153359037025[100]):0.04219918922882604[62]):0.03056533341375811[21]):0.045768303844850866[34],((Bacteria_CPR_ParcubacteriaOD1_GWA2_OD1_31_28_partial:0.6244195817553071,Bacteria_Parcubacteria_OD1_RIFCSPHIGHO2_01_FULL_OD1_47_10b:0.5945234713081069):0.11555268192643187[99],Ga0172379_10000029:0.6663866315188729):0.12346887177783028[94]):0.044962239269584536[89],(((((((((((((Bacteria_Parcubacteria_OD1_Moranbacteria_CG_Moran_01:0.06544893998056267,Bacteria_Parcubacteria_OD1_Moranbacteria_CG_Moran_02:0.12021200045033709):0.05353735587972075[100],Bacteria_Parcubacteria_OD1_Moranbacteria_RIFCSPLOWO2_12_FULL_OD1_Moranbacteria_48_12:0.10876208771590079):0.015965492831942818[87],Bacteria_Parcubacteria_OD1_Moranbacteria_RIFCSPHIGHO2_01_FULL_OD1_Moranbacteria_54_31:0.09195843748307819):0.10931125677324838[100],Bacteria_Parcubacteria_OD1_Moranbacteria_RIFCSPHIGHO2_01_FULL_OD1_Moranbacteria_55_24:0.21092717458017418):0.11190953157456063[100],Ga0172380_10032432:0.33266583565078234):0.078546392008366[99],(Ga0172379_10027592:0.052623443317321605,Ga0172379_10007673:0.048628822842408326):0.22353849307975304[100]):0.1218737455024721[100],((((Bacteria_CPR_ParcubacteriaOD1_GWD2_OD1_38_11:0.020506630201516884,Ga0172380_10001243:0.03368162231445471):0.02954939332331641[100],Bacteria_CPR_ParcubacteriaOD1_MoranbacteriaOD1_i_GWE2_OD1_36_40:0.05058836305716641):0.02944899628535369[99],Bacteria_CPR_ParcubacteriaOD1_MoranbacteriaOD1_i_GWC2_OD1_i_37_82:0.04804767481775718):0.2066909220102171[100],((Bacteria_CPR_ParcubacteriaOD1_MoranbacteriaOD1_i_GWE1_OD1_i_ACD11_lin_49_15:0.08789673289779287,Bacteria_CPR_ParcubacteriaOD1_MoranbacteriaOD1_i_GWC2_OD1_i_45_10:0.07591823566345246):0.05008348178738675[100],Bacteria_CPR_ParcubacteriaOD1_Moranbacteria:0.146089796042574):0.10377436847041821[100]):0.07476093685522178[100]):0.046289435639956356[65],(((Ga0172379_10000254:0.038154123134751305,Ga0172379_10000452:0.04042447050237241):0.19429292291302458[100],Ga0172381_10022055:0.20927974152353768):0.0382262384155565[60],Bacteria_Parcubacteria_OD1_Moranbacteria_RIFCSPHIGHO2_12_FULL_OD1_Moranbacteria_40_10:0.2967110385991236):0.04318420788554578[67]):0.027730453090231588[36],(((((Bacteria_CPR_ParcubacteriaOD1_MoranbacteriaOD1_i_GWF1_OD1_34_10:0.050775107492093685,Bacteria_CPR_ParcubacteriaOD1_MoranbacteriaOD1_i_GWE1_OD1_ACD7_35_17:0.05199352501342824):0.051644885290103915[100],Bacteria_CP_OD1_ALUMROCK_MS4_OD1_33_19:0.1321987261712323):0.04212594862799124[100],Ga0172377_10011473:0.10763046273984767):0.14050429349824523[100],(Ga0172382_10001677:0.000001,Ga0172377_10046265:0.000001):0.24210297000341496[100]):0.10685267924434383[100],Bacteria_CPR_ParcubacteriaOD1_MoranbacteriaOD1_i_GWD1_OD1_37_17:0.30195448756138177):0.08300480811605793[100]):0.05456915053456157[97],((((((((Ga0172379_10006812:0.02749359769335946,Ga0172379_10037953:0.036527984097276534):0.02048620637987497[98],Ga0172379_10022602:0.03381401636810022):0.008290143484178891[61],Ga0172379_10007316:0.05330062386344947):0.011896832394731938[37],((Ga0172379_10029029:0.000001,Ga0172380_10000343:0.000001):0.027627939157210957[100],Ga0172379_10005034:0.035601881323881646):0.005526818339013584[36]):0.07146646703401283[100],Ga0172379_10018461:0.11748473413232885):0.1380296342813958[100],Ga0172379_10000467:0.21801087334362101):0.03309429411346265[55],(((Ga0172379_10009675:0.09493320079987688,Bacteria_Parcubacteria_OD1_Moranbacteria_RifOxyA12_full_OD1_Moranbacteria_35_19:0.09616003632011116):0.10757722960482585[100],(Ga0172380_10000743:0.028489869531475254,Ga0172380_10001422:0.015672054413309766):0.2115382522410214[100]):0.05810211274080146[99],Bacteria_Parcubacteria_OD1_Moranbacteria_RifOxyA12_full_OD1_Moranbacteria_44_15:0.2124327731047373):0.02406049592873627[38]):0.04807969650416366[97],(((Ga0172380_10003857:0.1780533220288465,Ga0172379_10000141:0.12448167280046984):0.058695987153653206[100],(Bacteria_CPR_ParcubacteriaOD1_MoranbacteriaOD1_i_GWA2_OD1_39_41:0.09461018853932535,Ga0172380_10000162:0.07739380461432077):0.15053068484874643[100]):0.03935220140033335[87],Bacteria_Parcubacteria_OD1_Moranbacteria_RBG_19FT_COMBO_OD1_Moranbacteria_42_6:0.23804045854928546):0.03944254628438282[90]):0.09699221055727225[100]):0.0830493192940378[100],((Ga0172379_10034219:0.11715395245277982,Bacteria_Parcubacteria_OD1_Moranbacteria_RBG_13_OD1_Moranbacteria_45_13:0.11019849614274779):0.09220545775927969[100],(Bacteria_Parcubacteria_OD1_Moranbacteria_RIFOXYB1_FULL_OD1_Moranbacteria_44_23:0.08205355016077975,Bacteria_Parcubacteria_OD1_Moranbacteria_RIFOXYC1_FULL_OD1_Moranbacteria_44_13:0.0941579634102312):0.12282675154814182[100]):0.18127709724594077[100]):0.17493469131619577[100],((((Ga0172377_10006437:0.000612,Ga0172378_10000894:0.000001):0.03441481410230329[100],(Ga0172377_10001345:0.000427,Ga0172381_10003606:0.000001):0.05972045186267527[100]):0.06782354803666513[100],(Ga0172377_10002152:0.000001,Ga0172381_10002099:0.000425):0.09057122397061512[100]):0.0859503847748817[100],Ga0172381_10000655:0.12280733840255476):0.3822547512612431[100]):0.03374775096454341[71],Ga0172379_10033528:0.6717624193392058):0.04245201722055336[34]):0.030871826942398872[53],(((Bacteria_Parcubacteria_OD1_RIF_OD1_4_RIFCSPHIGHO2_01_FULL_RIF_OD1_04_46_36:0.18341514130054204,Bacteria_Parcubacteria_OD1_RIF_OD1_4_RIFCSPHIGHO2_12_FULL_RIF_OD1_04_45_11b:0.197470151350299):0.30993313133145106[100],(Ga0172379_10008471:0.045784057745288376,Bacteria_Parcubacteria_OD1_RIF_OD1_4_RIFCSPHIGHO2_02_FULL_RIF_OD1_04_46_16:0.013489862426451005):0.44750999169873173[100]):0.3177458617868121[100],Ga0172379_10017988:0.5724999406469324):0.21205847572112813[100]):0.028002515571456943[62],((((((((((((((((((((((((((Ga0172381_10032228:0.14298030135252393,Bacteria_CPR_ParcubacteriaOD1_Falkowbacteria_GWF2_OD1_43_32:0.11825312434709456):0.05056655219760664[90],(Ga0172381_10008498:0.058136058211789354,Ga0172379_10000024:0.05040587238115313):0.05315068026123404[100]):0.017465378437421908[7],((Ga0172382_10054022:0.12858337003467835,Ga0172381_10002569:0.102185004415011):0.037546791775221244[46],Ga0172381_10003557:0.10612423856207087):0.01921242216035468[13]):0.009784604024194898[3],Ga0172379_10007887:0.11786804435124276):0.015644345824072925[15],((Bacteria_Parcubacteria_OD1_Falkowbacteria_CG_Falkow_01:0.009352089980018352,Ga0172380_10001696:0.016888074868220748):0.20846926643621266[100],Ga0172380_10000265:0.1685460921003692):0.036398120659988376[34]):0.01459968382618193[31],Ga0172379_10003152:0.13905364425320954):0.026232198815613117[59],Ga0172381_10002970:0.11942186918117015):0.03742620245520989[96],(((Ga0172379_10001636:0.039652556705818576,Ga0172379_10023552:0.02316525349817722):0.07169570895701893[100],Ga0172379_10000122:0.07671368346241492):0.0360724527811187[100],(Ga0172377_10000304:0.000001,Ga0172382_10005982:0.000621):0.19440526170250116[100]):0.042304821641638846[99]):0.06669116000093567[100],((((((Ga0172381_10011391:0.0016678022236042978,Ga0172382_10004065:0.000001):0.000001[62],Ga0172377_10011410:0.000653):0.02720312836789418[100],Ga0172381_10016997:0.023439731453517965):0.02814932670356951[99],Ga0172377_10024235:0.07371279671340414):0.18677918987368258[100],((Ga0172379_10022518:0.032553110101627425,Ga0172379_10000261:0.03841007495183968):0.033147907780465946[100],Ga0172381_10006949:0.0497015672037473):0.07629393450544386[100]):0.07026011640022345[100],((Ga0172382_10050735:0.0650860771587527,Ga0172377_10000679:0.1391825204909094):0.06107705082712611[100],Ga0172378_10015479:0.12211692849652378):0.13387824150976613[100]):0.027279016010813173[69]):0.08684526372612567[100],((((Ga0172377_10014385:0.000001,Ga0172382_10006596:0.003329281773680748):0.11001961357337642[100],Ga0172381_10032640:0.11610285925071873):0.06446974058117183[100],Ga0172382_10004218:0.1922884266074667):0.11879330878295669[100],(Bacteria_Parcubacteria_OD1_Falkowbacteria_CG_Falkow_03:0.05993416911874938,Ga0172381_10006104:0.04813665181554461):0.19573911686417444[100]):0.03569918617951684[56]):0.05388265007692672[99],Ga0172380_10003562:0.26090851790660086):0.01733700571580643[35],Ga0172379_10009664:0.2944173685651785):0.025011775030462147[88],((((Bacteria_CPR_ParcubacteriaOD1_Falkowbacteria_GWE2_OD1_38_254:0.24488892877447466,Bacteria_Parcubacteria_OD1_Falkowbacteria_RIFOXYD2_FULL_OD1_Falkowbacteria_34_120:0.253479699722424):0.035232567891719135[72],Bacteria_Parcubacteria_OD1_Falkowbacteria_RIFOXYA2_FULL_OD1_Falkowbacteria_47_19:0.24265223659407154):0.026865605056444863[53],(Bacteria_CPR_ParcubacteriaOD1_Falkowbacteria_Parcubacteria_bacterium_SCGC_AAA036_E14_CrabSpa_001_178:0.2735129093997588,Ga0172380_10017511:0.18753787666681188):0.04373858738075809[69]):0.023068493439401205[54],Ga0172379_10005354:0.2563258480384252):0.057101651700090184[100]):0.026959238029768517[78],((((Ga0172379_10000380:0.044220588237635194,Ga0172379_10024999:0.06691942808118156):0.08809084405389633[100],Ga0172379_10011144:0.15141879756076948):0.23196825019122924[100],(Ga0172379_10000738:0.24664797810364192,Bacteria_CPR_ParcubacteriaOD1_GWE2_OD1_38_18:0.18869760188838436):0.07080212535387309[100]):0.02227913374208157[50],Bacteria_Parcubacteria_OD1_Falkowbacteria_CG_Falkow_02:0.30631227697802066):0.01914195095812188[15]):0.017625507853761935[18],((((((Ga0172379_10009643:0.07976588255228556,Ga0172379_10023765:0.06973159646613647):0.019384356556263693[83],Bacteria_CPR_ParcubacteriaOD1_GWC2_OD1_42_12:0.07961283236106853):0.024322078450362383[97],Bacteria_Parcubacteria_OD1_Falkowbacteria_RIFCSPLOWO2_12_FULL_OD1_Falkowbacteria_45_13:0.12177141959715154):0.050510694309117365[100],Bacteria_Parcubacteria_OD1_Falkowbacteria_CG_Falkow_04:0.13449520286575112):0.02544310372362979[66],((Ga0172381_10013715:0.06799968604882345,Ga0172379_10001372:0.051085926342620436):0.06098790504128093[100],Ga0172380_10015356:0.1351982342289264):0.012711702925463975[42]):0.03235505486702195[96],Ga0172381_10004876:0.09650200527755004):0.16884300660593476[100]):0.026056727592723927[60],(((((Ga0172379_10034640:0.0532338919200992,Ga0172379_10002019:0.03325224728937215):0.23421199988889585[100],Ga0172379_10002947:0.2618570593924918):0.06874614412154711[97],Bacteria_CPR_ParcubacteriaOD1_Falkowbacteria_GWA2_OD1_39_24:0.2590227856567875):0.062277510971429084[100],Ga0172381_10000028:0.3173965087090771):0.05418598992044332[100],((((Ga0172382_10001521:0.000393,Ga0172377_10005060:0.000393):0.000001[54],Ga0172381_10001250:0.000392):0.001394709293148555[75],Ga0172378_10020061:0.0037085892357962003):0.08060325288182613[100],Ga0172381_10037004:0.12440292877902914):0.3192332007939531[100]):0.054301395769911665[100]):0.06824564712393455[100],((Bacteria_Parcubacteria_OD1_Falkowbacteria_RIFCSPHIGHO2_02_FULL_OD1_Falkowbacteria_45_15:0.17078480879585634,Ga0172379_10004465:0.14932301922141455):0.10643194596237127[100],Bacteria_Parcubacteria_OD1_Falkowbacteria_RIFOXYA2_FULL_OD1_Falkowbacteria_47_9:0.27218975246759225):0.14416277994274562[100]):0.11112784643684925[100],(((Bacteria_Parcubacteria_OD1_Falkowbacteria_RIFCSPHIGHO2_02_FULL_OD1_Falkowbacteria_39_13:0.07732637283444799,Bacteria_Parcubacteria_OD1_Kuenenbacteria_CG_Kuenen_02:0.07128816602880006):0.2293648136253177[100],Bacteria_Parcubacteria_OD1_Falkowbacteria_RIFCSPLOWO2_12_FULL_OD1_Falkowbacteria_42_13:0.3118315255808639):0.06591685933922342[92],Bacteria_Parcubacteria_OD1_Kuenenbacteria_CG_Kuenen_01:0.33814588519700584):0.2053661431389795[100]):0.027621160456009264[60],((((Ga0172379_10003919:0.24389050583149086,Bacteria_Parcubacteria_OD1_Falkowbacteria_RIFOXYC2_FULL_OD1_Falkowbacteria_48_21:0.32879967022604184):0.07767898983839494[97],(Bacteria_CPR_ParcubacteriaOD1_GWC2_OD1_38_7:0.08750027692625295,Bacteria_CPR_ParcubacteriaOD1_GWA2_OD1_40_23:0.08334001416237102):0.28120382010967626[100]):0.05428780295768787[92],Ga0172379_10016970:0.3562581905856379):0.08576841530335466[100],Bacteria_Parcubacteria_OD1_Falkowbacteria_RIFOXYC2_FULL_OD1_Falkowbacteria_36_12:0.38276687155387856):0.11634349138439815[100]):0.031332543334472884[46],((Bacteria_Parcubacteria_OD1_Falkowbacteria_RIFOXYA2_FULL_OD1_Falkowbacteria_38_12:0.23242917441840127,Ga0172380_10002404:0.20112323084139616):0.21368565336536127[100],(Ga0172379_10018808:0.4791624130615131,Bacteria_Parcubacteria_OD1_Falkowbacteria_RBG_13_OD1_Falkowbacteria_39_14:0.4237723420128465):0.0604764319211788[25]):0.044162431306023375[16]):0.036272091212689705[16],(((((((((Ga0172379_10000224:0.17550513532016243,Ga0172379_10004202:0.1798254455004571):0.04632699730818457[97],(Ga0172381_10000613:0.2047015495064275,Bacteria_Parcubacteria_OD1_Falkowbacteria_RIFCSPLOWO2_02_FULL_OD1_Falkowbacteria_46_11b:0.17602091807453712):0.038715846833546674[89]):0.05084080780506639[99],Bacteria_Parcubacteria_OD1_Falkowbacteria_RIFCSPHIGHO2_02_FULL_OD1_Falkowbacteria_39_17:0.26635655238753175):0.05616157989105508[100],((Ga0172379_10026892:0.24960702367513932,Bacteria_Parcubacteria_OD1_Falkowbacteria_RIFCSPHIGHO2_01_FULL_OD1_Falkowbacteria_39_8:0.19111078841832985):0.03269271697919196[71],Bacteria_Parcubacteria_OD1_Falkowbacteria_RIFCSPHIGHO2_02_FULL_OD1_Falkowbacteria_56_16:0.3463125518643464):0.036819841886619464[35]):0.027501677161064553[29],Bacteria_Parcubacteria_OD1_Falkowbacteria_RIFCSPHIGHO2_01_FULL_OD1_Falkowbacteria_44_11:0.2930114929150882):0.04426340236246018[78],Bacteria_Parcubacteria_OD1_Falkowbacteria_RIFCSPHIGHO2_01_FULL_OD1_Falkowbacteria_47_11b:0.35071074609218345):0.06948628908349397[100],Bacteria_CPR_ParcubacteriaOD1_GWA2_OD1_38_13:0.4741660300662067):0.07339327630307624[100],(Bacteria_Parcubacteria_OD1_Falkowbacteria_RBG_13_OD1_Falkowbacteria_39_9:0.20569619125613592,Bacteria_Parcubacteria_OD1_Falkowbacteria_RBG_13_OD1_Falkowbacteria_36_9:0.21605502883008976):0.21057727504339274[100]):0.030814704532341697[37],((((Ga0172379_10011052:0.17837973941228213,Ga0172379_10005411:0.1816168246198977):0.06938927880809054[98],(Ga0172379_10001051:0.1838107854383746,Ga0172379_10001510:0.18389818438939676):0.1598889287312497[100]):0.04616209657597681[54],Bacteria_Parcubacteria_OD1_Falkowbacteria_RIFCSPLOWO2_01_FULL_OD1_Falkowbacteria_39_33:0.2884567008256105):0.18291672523646874[100],Ga0172379_10000928:0.44265661800253353):0.0800410794502322[99]):0.02563221756335121[16]):0.034011207395918674[31],(((((((((((((((((Bacteria_Parcubacteria_OD1_Magasanikbacteria_RifOxyC12_full_OD1_Magasanikbacteria_33_11:0.07687612789032672,Bacteria_Parcubacteria_OD1_Magasanikbacteria_RIFOXYD12_FULL_OD1_Magasanikbacteria_33_17:0.05618206599898601):0.033204002224497486[99],(Bacteria_Parcubacteria_OD1_Magasanikbacteria_CG_Magasa_01:0.060586225612557776,Ga0172380_10004091:0.050727388253187566):0.08545208617225208[100]):0.0292976217754064[95],Ga0172380_10000262:0.09785352337419129):0.16615597523870873[100],Bacteria_Parcubacteria_OD1_Magasanikbacteria_RIFOXYC2_FULL_OD1_Magasanikbacteria_39_8:0.17411595885493902):0.07754621138671691[100],Ga0172379_10000368:0.3113797950331292):0.043662990103928045[85],Ga0172379_10005188:0.2777620924618951):0.050869202682273684[99],((Bacteria_Parcubacteria_OD1_Magasanikbacteria_RIFCSPHIGHO2_12_FULL_OD1_Magasanikbacteria_41_16:0.3024743010892683,Bacteria_CPR_ParcubacteriaOD1_Magasanikbacteria_GWA2_OD1_56_11:0.27525629757660575):0.0967893637875652[100],Bacteria_CPR_ParcubacteriaOD1_Magasanikbacteria_Parcubacteria_bacterium_SCGC_AAA011_J21_DUSEL_001_191:0.31466535351942904):0.05481367170933016[96]):0.0643563305229149[100],Bacteria_Parcubacteria_OD1_Magasanikbacteria_RIFCSPHIGHO2_02_FULL_OD1_Magasanikbacteria_47_14:0.3588142312576639):0.026122505007844854[37],(((Bacteria_Parcubacteria_OD1_Magasanikbacteria_RIFCSPHIGHO2_02_FULL_OD1_Magasanikbacteria_33_17:0.152638658315166,Bacteria_Parcubacteria_OD1_Magasanikbacteria_RIFOXYB1_FULL_OD1_Magasanikbacteria_40_15:0.1054558230884779):0.18879407253912683[100],(Bacteria_Parcubacteria_OD1_Magasanikbacteria_RIFCSPHIGHO2_02_FULL_OD1_Magasanikbacteria_51_14:0.25499018907124205,Bacteria_Parcubacteria_OD1_Magasanikbacteria_RIFCSPLOWO2_02_FULL_OD1_Magasanikbacteria_47_16:0.3607220405840872):0.04422384986526007[64]):0.032553210338376104[31],((Bacteria_CPR_ParcubacteriaOD1_Magasanikbacteria_GWD2_OD1_43_18:0.09199117730463735,Bacteria_Parcubacteria_OD1_Magasanikbacteria_CG_Magasa_02:0.08250046003482758):0.25723721870031735[100],Bacteria_Parcubacteria_OD1_Magasanikbacteria_RIFCSPHIGHO2_02_FULL_OD1_Magasanikbacteria_41_13:0.3236568760440375):0.0648937255528863[58]):0.02944046919947052[32]):0.04598515059277908[89],((Bacteria_CPR_ParcubacteriaOD1_Magasanikbacteria_GWC2_OD1_37_14:0.07427325004004226,Ga0172379_10028515:0.06755392637322055):0.19000938384503696[100],Bacteria_Parcubacteria_OD1_Magasanikbacteria_RIFCSPLOWO2_12_FULL_OD1_Magasanikbacteria_43_12:0.2686643612479016):0.08276255708411506[100]):0.12177578343277107[100],((((((((Ga0172380_10034031:0.10694330992184309,Ga0172379_10000361:0.14127835445199377):0.03745599877394756[60],Ga0172381_10001071:0.11987251575888314):0.10394931289311415[100],Bacteria_Parcubacteria_OD1_Magasanikbacteria_RIFOXYA2_FULL_OD1_Magasanikbacteria_44_8:0.19765502420026637):0.061771275438651685[99],((Bacteria_Parcubacteria_OD1_Magasanikbacteria_RIFOXYD2_FULL_OD1_Magasanikbacteria_36_9:0.08233774885056055,Ga0172379_10000192:0.04555318522212781):0.05327686207565385[100],Ga0172379_10021825:0.11336087339718759):0.16655621049555247[100]):0.018868940915635957[21],Ga0172379_10042187:0.2729867128536787):0.02862419508401537[10],((((Bacteria_Parcubacteria_OD1_Magasanikbacteria_RIFCSPHIGHO2_01_FULL_OD1_Magasanikbacteria_47_8:0.14396624139324077,Bacteria_CPR_ParcubacteriaOD1_Magasanikbacteria_GWA2_OD1_50_22_partial:0.19390824134431428):0.0306432029622572[53],Bacteria_Parcubacteria_OD1_Magasanikbacteria_RIFCSPLOWO2_02_FULL_OD1_Magasanikbacteria_44_11:0.17734946123353446):0.02271873548269765[67],(Ga0172379_10002809:0.14689715541507864,Ga0172379_10001076:0.1415341332322777):0.0625771119762435[100]):0.08096053705993356[100],Ga0172381_10000532:0.3217384485569812):0.030247586270208515[38]):0.01948495064175315[3],(((Bacteria_Parcubacteria_OD1_Magasanikbacteria_RIFOXYC2_FULL_OD1_Magasanikbacteria_42_28:0.20368337128046488,Bacteria_Parcubacteria_OD1_Magasanikbacteria_RIFOXYD2_FULL_OD1_Magasanikbacteria_41_14:0.22436905163468834):0.0698825683245432[100],Ga0172380_10029908:0.29149894070126736):0.07242131631956417[99],(Ga0172379_10031363:0.09004295877636936,Ga0172379_10035163:0.050516215201307446):0.1643778406562193[100]):0.03334739496106742[24]):0.027480557978095455[33],((Bacteria_CPR_ParcubacteriaOD1_Magasanikbacteria_GWA2_OD1_40_10_partial:0.12256246110274338,Bacteria_Parcubacteria_OD1_Magasanikbacteria_RIFOXYD1_FULL_OD1_Magasanikbacteria_40_23:0.11858885297611277):0.12280356739717035[100],Bacteria_CPR_ParcubacteriaOD1_Magasanikbacteria_GWA2_OD1_46_17_plus:0.2622353099381787):0.050176017418602115[97]):0.16469705317297878[100]):0.07472537187942274[100],Bacteria_Parcubacteria_OD1_Uhrbacteria_RIFCSPLOWO2_02_FULL_OD1_Uhrbacteria_51_9:0.574751559586085):0.03502281142458363[81],Bacteria_Parcubacteria_OD1_Magasanikbacteria_RIFCSPHIGHO2_02_FULL_OD1_Magasanikbacteria_50_9b:0.5832767909995455):0.04241002010990602[58],((Bacteria_CPR_ParcubacteriaOD1_Magasanikbacteria_GWC2_OD1_41_17:0.32794367088669363,Bacteria_CPR_ParcubacteriaOD1_Magasanikbacteria_GWA2_OD1_42_32:0.3387795158863951):0.11585023669110805[100],Bacteria_CPR_ParcubacteriaOD1_Magasanikbacteria_GWA2_OD1_45_39_plus:0.4496367978438225):0.11073827188241525[100]):0.04871017441032288[64],(((((((((Bacteria_CPR_ParcubacteriaOD1_Uhrbacteria_GWF2_OD1_39_13:0.14444647367546626,Bacteria_CPR_ParcubacteriaOD1_Uhrbacteria_GWA2_OD1_52_8b:0.13691873614488737):0.138904219695831[100],Bacteria_Parcubacteria_OD1_Uhrbacteria_RIFOXYB2_FULL_OD1_Uhrbacteria_45_11:0.27505850988437475):0.06205843690222235[100],(Bacteria_CPR_ParcubacteriaOD1_Uhrbacteria_GWF2_OD1_46_218:0.33306640278279875,Bacteria_Parcubacteria_OD1_Uhrbacteria_RIFOXYB2_FULL_OD1_Uhrbacteria_41_18:0.2400029011692677):0.06386086253570644[100]):0.06692198385776926[100],((((Ga0172379_10001466:0.1015092541847511,Bacteria_Parcubacteria_OD1_Uhrbacteria_RIFOXYB2_FULL_OD1_Uhrbacteria_57_15:0.08737917486541047):0.02058337786709208[59],Ga0172379_10001855:0.11027129487285947):0.1660780890379736[100],Bacteria_Parcubacteria_OD1_Uhrbacteria_RIFCSPHIGHO2_01_FULL_OD1_Uhrbacteria_63_20:0.2618858300174751):0.029229019120211586[35],Bacteria_CPR_ParcubacteriaOD1_Uhrbacteria_GWA2_OD1_53_10:0.2726793526751332):0.026609751932923142[74]):0.05703190102946776[100],(((Ga0172379_10010746:0.1331549838312842,Ga0172379_10002453:0.12897103906414076):0.038597221692282524[67],Bacteria_CPR_ParcubacteriaOD1_Uhrbacteria_GWE2_OD1_45_35:0.15224465554160949):0.12248850872202333[100],Bacteria_CPR_ParcubacteriaOD1_Uhrbacteria_GWA2_OD1_41_10:0.27153835010267446):0.11070575506293112[100]):0.053511855771618766[99],(((((Ga0172379_10004987:0.12420577814533296,Ga0172380_10006519:0.09768858832364025):0.1398533165108189[100],(Ga0172379_10000595:0.19611846738010597,Bacteria_CPR_ParcubacteriaOD1_Uhrbacteria_GWD2_OD1_52_7:0.14951270808702644):0.05431916264793202[97]):0.14015049929254575[100],(Ga0172379_10000573:0.3194164360850009,Bacteria_CPR_ParcubacteriaOD1_Uhrbacteria_GWB2_OD1_ACD66_rel_41_36:0.35178075406985343):0.068935571245611[97]):0.1425379208919635[100],Bacteria_Parcubacteria_OD1_Uhrbacteria_RIFCSPHIGHO2_02_FULL_OD1_Uhrbacteria_53_13:0.4539985740979544):0.04258577875977121[34],(Bacteria_CPR_ParcubacteriaOD1_GWA2_OD1_56_7_partial:0.29129946556709063,Bacteria_Parcubacteria_OD1_Uhrbacteria_RIFCSPHIGHO2_02_FULL_OD1_Uhrbacteria_54_11:0.2912818107011894):0.1745027767395797[100]):0.04202067927095632[31]):0.031078260340826613[29],Bacteria_CPR_ParcubacteriaOD1_Uhrbacteria_ACD76:0.46484806186398453):0.07003085631408013[100],(((((Ga0172379_10000102:0.09012883799182969,Ga0172379_10015280:0.10418683451860256):0.11817339465049637[100],Ga0172379_10002629:0.1825972838785952):0.14909381484516437[100],(Ga0172379_10014835:0.21123335085043626,Ga0172379_10037372:0.20159700850417028):0.07596874310606383[100]):0.045770019883816904[66],Bacteria_Parcubacteria_OD1_Uhrbacteria_RIFCSPHIGHO2_12_FULL_OD1_Uhrbacteria_60_25:0.2918768778420824):0.04125177150669446[81],((Ga0172379_10012385:0.29832307862166685,Ga0172379_10000151:0.25553770386156716):0.049118063303117854[85],Ga0172379_10033567:0.2683183544818637):0.06674592475742402[99]):0.21808989939618506[100]):0.04871734339921607[74],(((((Ga0172381_10020300:0.29757046382925134,Bacteria_Parcubacteria_OD1_Uhrbacteria_RIFCSPHIGHO2_02_FULL_OD1_Uhrbacteria_60_10:0.2305718059364339):0.000001[31],Ga0172381_10010333:0.27323953489546327):0.05541109827404833[64],Ga0172379_10000486:0.2822669095471677):0.04458832395734058[79],((Bacteria_Parcubacteria_OD1_CG_CPR_02_CG_CPR02_01:0.04701082789592537,Bacteria_Parcubacteria_OD1_Uhrbacteria_RIFOXYC2_FULL_OD1_Uhrbacteria_47_19:0.04381026807956134):0.242394728419971[100],Bacteria_OD1_uncultured_SG8_24:0.2231087391494304):0.06923316932940438[100]):0.18093941037380068[100],(Ga0172379_10001282:0.34425440626981985,Bacteria_Parcubacteria_OD1_Uhrbacteria_RIFCSPHIGHO2_02_FULL_OD1_Uhrbacteria_57_19:0.3240238793897241):0.13467392456491734[100]):0.05929992000561768[82]):0.0521728528828862[75]):0.030998818917695736[31],((((Bacteria_Parcubacteria_OD1_Uhrbacteria_RIFCSPLOWO2_01_FULL_OD1_Uhrbacteria_47_25:0.1429545147841602,Bacteria_Parcubacteria_OD1_Uhrbacteria_RIFCSPLOWO2_02_FULL_OD1_Uhrbacteria_48_12:0.1111588783090034):0.20719477205930747[100],Ga0172380_10000551:0.4018463247923969):0.16703796994645792[100],(Bacteria_Parcubacteria_OD1_Uhrbacteria_RIFCSPLOWO2_02_FULL_OD1_Uhrbacteria_49_11:0.23561946697112868,Bacteria_Parcubacteria_OD1_Uhrbacteria_RIFCSPLOWO2_02_FULL_OD1_Uhrbacteria_54_37:0.35155896371041795):0.3162200167218021[100]):0.06216484278985668[13],((Bacteria_CPR_ParcubacteriaOD1_GWA2_OD1_47_26:0.27306997672131805,Bacteria_Parcubacteria_OD1_Uhrbacteria_RIFCSPLOWO2_02_FULL_OD1_Uhrbacteria_46_25:0.24081709762677272):0.2582174087849394[100],Bacteria_CPR_ParcubacteriaOD1_Parcubacteria_bacterium_SCGC_AAA011_N16_Dusel_001_262:0.5075787111975902):0.04966146639975966[24]):0.05543724193572075[12]):0.03037512702661216[12],Bacteria_Parcubacteria_OD1_CG_CPR_12_CG_CPR12_01:0.5740534950547809):0.04885171936512078[13]):0.014071923857542412[3],(((Bacteria_CPR_ParcubacteriaOD1_Parcubacteria_bacterium_SCGC_AB_164_E21_SAK_001_209:0.37452917642929595,Ga0172379_10016841:0.3821539882177185):0.08597048005541552[98],Bacteria_Parcubacteria_OD1_Kuenenbacteria_CG_Kuenen_03:0.617213329702142):0.11549707220106198[100],Ga0172381_10004584:0.4169185931445907):0.09896366604608042[92]):0.02371950568830794[1],(((((((Ga0172379_10000833:0.10005098416401514,Ga0172379_10000109:0.07874064245127821):0.03568643800168081[100],Bacteria_Parcubacteria_OD1_RIF_OD1_3_RIFOXYC1_FULL_RIF_OD1_03_37_11:0.1153936348062139):0.17875911662736144[100],(Ga0172379_10004769:0.28796116411305084,Bacteria_CPR_ParcubacteriaOD1_GWA2_OD1_36_10:0.24180931568593378):0.05410931685428366[51]):0.15165548625245195[100],(Bacteria_Parcubacteria_OD1_RIF_OD1_3_RIFCSPLOWO2_01_FULL_RIF_OD1_03_52_15:0.26471788628247683,Bacteria_Parcubacteria_OD1_RIF_OD1_3_RIFCSPLOWO2_01_FULL_RIF_OD1_03_53_11:0.26412737164076416):0.19981117284540373[100]):0.10678089205807595[100],(((((Ga0172379_10035620:0.16316533051649795,Ga0172379_10013959:0.14878832305807377):0.16679163568565203[100],Ga0172379_10000043:0.2393971750875541):0.056813731353795305[96],Bacteria_Parcubacteria_OD1_RIF_OD1_3_RIFCSPLOWO2_01_FULL_RIF_OD1_03_45_10:0.27159038321216533):0.04257673913375726[96],Bacteria_CPR_ParcubacteriaOD1_GWA2_OD1_43_17_A823:0.28323815054002655):0.12030068431811225[100],Bacteria_Parcubacteria_OD1_CG_CPR_13_CG_CPR13_01:0.5049383852086966):0.0670552306395309[99]):0.044918505730923375[100],Ga0172381_10022356:0.5898449488018414):0.03552127351668677[56],(((Bacteria_Parcubacteria_OD1_RIF_OD1_2_RIFOXYD2_FULL_RIF_OD1_02_43_21:0.341728360629459,Bacteria_Parcubacteria_OD1_RIF_OD1_2_RIFCSPLOWO2_02_FULL_RIF_OD1_02_44_20:0.3797302350084628):0.2568801715098967[100],(Bacteria_CPR_ParcubacteriaOD1_GWA2_OD1_44_12:0.4782998776885363,Bacteria_Parcubacteria_OD1_RIF_OD1_2_RIFCSPLOWO2_01_FULL_RIF_OD1_02_44_13:0.5029971992281004):0.07933294415678871[97]):0.06837198625832874[96],(Bacteria_Parcubacteria_OD1_CG_CPR_14_CG_CPR14_01:0.38708656850904033,Bacteria_Parcubacteria_OD1_RIF_OD1_3_RIFCSPLOWO2_02_FULL_RIF_OD1_03_48_11:0.31712842859012635):0.31425533448174514[100]):0.044509718580338475[32]):0.021338411486113706[3]):0.024787815077326858[3],((((((((Ga0172379_10020044:0.026448130193795105,Ga0172379_10001796:0.02489187892483713):0.017562102456046613[94],Ga0172379_10017492:0.04846539849054432):0.14994185622102663[100],Ga0172379_10000131:0.251298007714011):0.0872291968310086[100],Ga0172379_10000091:0.2508647883270081):0.2760353443457024[100],(((Ga0172379_10007936:0.2718360239584179,Ga0172379_10008244:0.2656190440384756):0.06788163436570249[95],Bacteria_Parcubacteria_OD1_RIF_OD1_3_RIFOXYC2_FULL_RIF_OD1_03_42_11:0.27224410673876864):0.11237553594062843[100],Bacteria_CPR_ParcubacteriaOD1_GWA2_OD1_46_39_partial:0.4078939823248655):0.20319329840177636[100]):0.06815005929145013[71],Ga0172379_10000499:0.5287881961319463):0.056740655898628756[37],(((Ga0172379_10000035:0.2893280412243815,Ga0172379_10003224:0.32100710948847366):0.12698282961742224[100],(Ga0172379_10023512:0.31279408752019044,Bacteria_Parcubacteria_OD1_RIF_OD1_1_RIFCSPLOWO2_01_FULL_RIF_OD1_01_48_11:0.34302463864641064):0.1279530234972519[100]):0.07711185392968467[97],Ga0172379_10000123:0.5039627185525446):0.035277749673888525[35]):0.04443445101722121[22],(((((Ga0172379_10008472:0.3222661925969872,Ga0172379_10000249:0.2741342152759665):0.14796055899457583[100],Ga0172379_10021310:0.36412478534452175):0.08462208568363705[99],Bacteria_Parcubacteria_OD1_RIF_OD1_1_RIFOXYA2_FULL_RIF_OD1_01_38_24:0.5509993893566287):0.06582389583833859[97],(Bacteria_Parcubacteria_OD1_RIF_OD1_1_RIFCSPHIGHO2_12_FULL_RIF_OD1_01_48_17:0.3846839567769571,Bacteria_Parcubacteria_OD1_RIF_OD1_1_RIFCSPLOWO2_02_FULL_RIF_OD1_01_42_19:0.41170392761586383):0.15084284471791376[100]):0.046302487677765125[96],Ga0172379_10000485:0.4655844631448556):0.04565534984677022[28]):0.03957355699294807[20]):0.0663293088921515[99],((((((((Bacteria_SM2F11_RIFCSPHIGHO2_12_FULL_SM2F11_48_16:0.16496338332749882,Bacteria_SM2F11_RIFCSPHIGHO2_02_FULL_SM2F11_43_13b:0.17377196719473043):0.02521174929992842[90],Bacteria_SM2F11_RIFCSPHIGHO2_01_FULL_SM2F11_45_18:0.13365176419214508):0.037932082878244344[98],Bacteria_SM2F11_RIFCSPHIGHO2_12_FULL_SM2F11_42_22:0.1837569014418099):0.0476063439915988[97],Bacteria_SM2F11_RIFCSPHIGHO2_01_FULL_SM2F11_46_24:0.23669179822711772):0.0477427597165625[97],Ga0172379_10000106:0.2731554867365311):0.18196923844962232[100],(((Bacteria_SM2F11_RIFCSPHIGHO2_01_FULL_SM2F11_46_14:0.1424012422544223,Bacteria_SM2F11_RIFCSPLOWO2_02_FULL_SM2F11_48_13:0.11503283065320291):0.06709850075930124[99],Bacteria_SM2F11_RIFCSPHIGHO2_01_FULL_SM2F11_49_9:0.20206024002462764):0.12591319773503695[100],(Bacteria_SM2F11_RIFCSPHIGHO2_01_SM2F11_52_17:0.10997646454271237,Bacteria_CPR_ParcubacteriaOD1_GWA2_OD1_rel_51_12:0.12030593021565128):0.2505174486424333[100]):0.18131274401219732[100]):0.052439251505120854[47],(Ga0172379_10002482:0.4105747992826587,Bacteria_SM2F11_RIFCSPHIGHO2_02_FULL_SM2F11_46_11:0.4602018976873712):0.06608652597534492[84]):0.052552908755817196[67],(Bacteria_SM2F11_RIFCSPHIGHO2_01_FULL_SM2F11_43_23:0.35872882380013005,Bacteria_SM2F11_RIFCSPHIGHO2_01_FULL_SM2F11_50_11:0.36219817890179584):0.2361202267039344[100]):0.1544582336981466[100]):0.04589258174217381[81]):0.07445954461147286[98],((((((((((((((Ga0172381_10041452:0.000001,Ga0172381_10034789:0.000001):0.23920131957698665[100],Ga0172379_10003673:0.31853293092297985):0.04831487186231387[76],Bacteria_CP_Peregrinibacteria_BJP_IG2102_PER_44_74_final:0.3069849993820659):0.025796144528855702[27],Ga0172379_10000172:0.31091718735569085):0.031818782671497736[33],Bacteria_Peregrinibacteria_RIFCSPLOWO2_01_FULL_Peregrinibacteria_39_12:0.27136200396332955):0.05731609444865837[100],(Ga0172380_10016271:0.2762399586082318,Ga0172381_10008667:0.24743527952193345):0.12048587278017697[100]):0.07215661595113643[100],((Ga0172379_10004188:0.012722996236102624,Bacteria_CPR_PeregrinibacteriaPER_PER_GWC2_39_14:0.026717629700542034):0.3730364354356963[100],Ga0172380_10001881:0.3744451930775541):0.09042429937656493[99]):0.043375261531514386[76],(Ga0172379_10009818:0.45498856184749403,Ga0172379_10000274:0.31571104974353936):0.08251125932605241[90]):0.034554136844909156[78],(((((Ga0172379_10004329:0.10095996549714581,Bacteria_Peregrinibacteria_RIFOXYC2_FULL_Peregrinibacteria_41_22:0.07992785851815754):0.19391580944826092[100],Bacteria_CPR_PeregrinibacteriaPER_PER_GWF2_43_17:0.24747048169257368):0.0454524819703761[83],Bacteria_Peregrinibacteria_CG_PER_02:0.20502511751810637):0.17083036692152254[100],Bacteria_Peregrinibacteria_RIFCSPLOWO2_02_FULL_Peregrinibacteria_48_14:0.4807010875456581):0.06771301613573355[91],((Bacteria_CPR_PeregrinibacteriaPER_GWA2_PER_44_7:0.24272605151377968,Bacteria_CPR_PeregrinibacteriaPER_PER_GWF2_39_17:0.20359687319110575):0.0992884854086502[100],Ga0172379_10005525:0.2605367558502354):0.14060587214211928[100]):0.06347176137409782[87]):0.03476820034795791[45],((Bacteria_CPR_PeregrinibacteriaPER_PER_GWF2_33_10:0.2526704689118269,Bacteria_Peregrinibacteria_RIFOXYB2_FULL_Peregrinibacteria_32_7:0.22509038884943733):0.3291009930854547[100],Bacteria_Peregrinibacteria_RIFOXYA2_FULL_Peregrinibacteria_33_7:0.4345011670826806):0.05631151556863356[58]):0.05263051169160926[87],(Ga0172379_10000116:0.5789071526883838,Bacteria_Peregrinibacteria_CG_PER_03:0.6159940039233112):0.08540289141857249[30]):0.0389957656416966[27],(((((((((Ga0172381_10025748:0.000001,Ga0172381_10014107:0.0029602734915323836):0.07929827052529159[97],Bacteria_PER_ii_RIFOXYC2_FULL_PER_ii_58_32:0.083304444999873):0.04106899849497481[92],Bacteria_Peregrinibacteria_CG_PER_01:0.07533433642612364):0.1928444700526879[100],((Bacteria_CPR_PeregrinibacteriaPER_GWB1_PER_54_5:0.261492922700727,Ga0172381_10015912:0.21924831787255306):0.03482774267363409[42],Bacteria_PER_ii_RIFOXYD2_FULL_PER_ii_58_15:0.23641503448186674):0.045479669369416165[90]):0.029808342013573075[46],(Bacteria_PER_ii_RIFCSPLOWO2_12_FULL_PER_ii_53_10:0.05667685473973583,Bacteria_PER_ii_RIFCSPHIGHO2_01_FULL_PER_ii_55_13:0.06226975101541665):0.2284822468601595[100]):0.026213051765604067[49],((Ga0172379_10000137:0.16958965441458496,Ga0172379_10002516:0.14855437350007294):0.0810065959937809[100],Bacteria_PER_ii_RIFCSPHIGHO2_01_FULL_PER_ii_51_35:0.20310328839299707):0.07096669885844253[100]):0.029763090449770324[73],((Bacteria_PER_ii_RIFCSPHIGHO2_02_FULL_PER_ii_49_16:0.26795613737167523,Bacteria_PER_ii_RIFCSPLOWO2_12_FULL_PER_ii_55_15:0.3026578700150546):0.06988132793942681[100],Bacteria_PER_ii_RIFCSPHIGHO2_01_FULL_PER_ii_51_9:0.27672368435149775):0.041304191571020166[79]):0.05977599495588404[94],((Ga0172379_10007139:0.08468844046056923,Bacteria_PER_ii_RIFCSPHIGHO2_02_FULL_PER_ii_51_15:0.12734916516094286):0.05765262551646577[99],Ga0172379_10034304:0.10507610130767064):0.17321115511692264[100]):0.31785587556817774[100],Bacteria_CP_Peregrinibacteria_ALUMROCK_MS4_Peregrinibacteria_34_52:0.6288697548860536):0.09425442325195288[62]):0.04141251147345093[74],(((((Bacteria_GN02_CG_GN02_02:0.056170639272831835,Bacteria_GN02_CG_GN02_01:0.04855136777152458):0.23036149120130833[100],Bacteria_CPR_GracilibacteriaBD1_5_ACD49:0.31227471248269545):0.1061227333650816[100],(Ga0172380_10005288:0.18836271422271889,Ga0172380_10014356:0.18798110764857157):0.5361127223552686[100]):0.07514444873823667[61],((Bacteria_CP_GN02_candidate_division_GN02_bacterium_JGI_0000069_P22_CrabSpa_001_278:0.1710967056397985,Bacteria_CP_GN02_candidate_division_GN02_bacterium_JGI_0000069_K10_CrabSpa_001_277:0.2047880534340023):0.04800328982677193[73],Bacteria_uncultured_bacterium_SCGC_AB_137_L08:0.20168876692997761):0.17054837938973888[100]):0.22423621711359898[100],(((Bacteria_CP_SR1_RAAC1_SR1_1:0.27094457791096405,Bacteria_CP_SR1_candidate_division_SR1_bacterium_taxon_345_1OR1_recoded_II:0.30583417794917134):0.17948107598053964[100],Bacteria_CP_SR1_bacterium_Aalborg_AAW_1:0.5118925248768322):0.10309905878843306[96],Ga0172380_10002717:0.529614695381492):0.4187680494984174[100]):0.10465426532628674[99]):0.04825362537573463[78],(Bacteria_RIFCSPHIGHO2_01_FULL_PER_46_8:0.5127793460842822,Bacteria_RBG_16_CPR_42_10:0.6196826558510757):0.14671937537452928[100]):0.10256672496505637[100]):0.03126019806620217[35],(((((((((((((((((Ga0172377_10021106:0.000001,Ga0172378_10000360:0.000567):0.06416514612305857[100],(Ga0172377_10019903:0.02629668345085756,Bacteria_CP_Saccharibacteria_BJP_IG2103_TM7_39_1400:0.02672484782879092):0.0191280141066712[99]):0.01754544562170013[78],Ga0172377_10015176:0.0665579299504322):0.018192873615613614[81],Ga0172381_10017724:0.18452528279190394):0.0184592278327127[79],(Ga0172379_10002830:0.1234620434905449,Bacteria_TM7_CG_TM7_01:0.08706361713344002):0.043063388701085614[100]):0.054183232315048624[100],Ga0172382_10016929:0.1585869631251806):0.039573980341536696[92],(Bacteria_Saccharibacteria_TM7_RIFCSPHIGHO2_01_FULL_Saccharibacteria_45_15:0.06648022418652699,Bacteria_CPR_SaccharibacteriaTM7_GWC2_TM7_44_17_complete:0.05808222715613143):0.03677754722512061[100]):0.024527891673416402[77],(((Ga0172380_10031428:0.01884288802841194,Ga0172380_10033508:0.017678103458557803):0.008497306491809464[51],Ga0172380_10014320:0.012274481084636246):0.0382453274210941[100],Ga0172379_10013525:0.031104902005751):0.05101750325949794[100]):0.023453714945322002[70],((Ga0172380_10000066:0.0706390275090314,Ga0172380_10044341:0.1239974532504009):0.029314520056605176[98],Bacteria_CPR_SaccharibacteriaTM7_RAAC3_TM7_complete:0.09303288394524678):0.05155650373302478[100]):0.027280270957046326[78],Bacteria_CPR_SaccharibacteriaTM7_GWC2_TM7_48_9:0.1541483443896663):0.04020341051093501[87],((((Bacteria_unclassified_Bacteria_Candidatus_Saccharibacteria_Candidatus_TM7_s1_Draft1_Illumina_assembly:0.08324801317118169,Bacteria_unclassified_Bacteria_Candidatus_Saccharibacteria_Candidatus_TM7_s7_Draft1_Illumina_assembly:0.05897466043719124):0.07259472568758785[100],Bacteria_unclassified_Bacteria_Candidatus_Saccharibacteria_Candidatus_TM7_SBR2:0.14168231846656276):0.0249377436133158[42],Bacteria_unclassified_Bacteria_Candidatus_Saccharibacteria_Candidatus_TM7_SBR4:0.07241592117497886):0.022660950030396254[37],(Bacteria_CPR_SaccharibacteriaTM7_Candidatus_Saccharimonas_aalborgensis_complete:0.10313160567358448,Bacteria_unclassified_Bacteria_Candidatus_Saccharibacteria_Candidatus_TM7_SBR3:0.08551468027478526):0.028227337326281354[95]):0.07074781513644268[100]):0.09548787716529628[100],(Bacteria_Saccharibacteria_TM7_RIFCSPHIGHO2_12_FULL_Saccharibacteria_42_8:0.2399623574874452,'Bacteria_CP_TM7_candidate_division_TM7_genomosp._GTL1_unfinished_sequence':0.2139185765062681):0.03013324165615261[43]):0.18408219750726929[100],((((((Bacteria_Saccharibacteria_TM7_RIFCSPHIGHO2_12_FULL_Saccharibacteria_48_21:0.2842804681829536,Bacteria_Saccharibacteria_TM7_RIFCSPHIGHO2_12_FULL_Saccharibacteria_49_19:0.2028500001084108):0.09812267925727447[100],(Bacteria_Saccharibacteria_TM7_RIFCSPHIGHO2_12_FULL_Saccharibacteria_47_17:0.1445727498918088,Bacteria_Saccharibacteria_TM7_RIFCSPHIGHO2_12_FULL_Saccharibacteria_47_16b:0.17750080315971672):0.14674479002786756[100]):0.09103686989684778[100],Ga0172381_10039769:0.2598185148020149):0.03940696603978022[67],Bacteria_Saccharibacteria_TM7_RIFCSPHIGHO2_12_FULL_Saccharibacteria_41_12:0.31491237269003136):0.0444246708350704[75],Bacteria_Saccharibacteria_TM7_RIFCSPHIGHO2_02_FULL_Saccharibacteria_47_12:0.29478044204670617):0.037955066073462884[71],((Bacteria_CP_Saccharibacteria_DOLZORAL124_TM7_51_29:0.13386403200943242,Ga0172381_10002576:0.11865306566889178):0.1258826034146847[100],(Ga0172379_10002169:0.16393095149336734,Ga0172380_10009850:0.1909730560600602):0.030952069533012327[40]):0.05179213814937267[85]):0.11237978767304435[99]):0.36176788847748886[100],(((Bacteria_CPR_CPR2_GWC2_CPR2_39_10:0.03559634910416998,Bacteria_CPR2_CG_CPR17_01:0.04703002905779652):0.15605747262512848[100],Bacteria_CPR_CPR2_GWD2_CPR2_39_7:0.16178206741693568):0.2690959527503618[100],Ga0172381_10000335:0.4801496151458333):0.12162349012285922[99]):0.051054954856203594[50],Bacteria_CG_CPR_16_CG_CPR16_01:0.5847115617626666):0.0734101177420583[87],(((((((((Ga0172381_10000604:0.340952596924883,Ga0172379_10000618:0.29313533464649577):0.1494082441112372[100],(Ga0172377_10000874:0.001141195631872094,Ga0172381_10012358:0.000001):0.5096007158535514[100]):0.06996392980864918[91],Ga0172381_10000565:0.458389126197821):0.053572199657664576[71],((Ga0172381_10027617:0.2434272260496635,Ga0172380_10000791:0.2115982004246053):0.21330065179447244[100],Ga0172380_10000211:0.3703620616392471):0.0705544967076972[53]):0.4017972950612658[100],((Bacteria_CPR_BerkelbacteriaACD58_GWA2_Berkelbacteria_35_9:0.6963839071681828,Bacteria_Berkelbacteria_CG_Berkel_03:0.5536330460242413):0.1201566498902853[90],Bacteria_Berkelbacteria_CG_Berkel_02:0.752922596808332):0.10856082766111408[80]):0.04631764454176057[26],(Bacteria_CPR_BerkelbacteriaACD58_GWA2_ACD58_46_7:0.383067209616784,Bacteria_Berkelbacteria_RIFCSPLOWO2_01_FULL_Berkelbacteria_50_28:0.38111080646956275):0.44382279967104266[100]):0.05842065944143737[48],((((Bacteria_CPR_BerkelbacteriaACD58_GWA1_Berkelbacteria_36_9_partial:0.13751640444070645,Ga0172379_10001871:0.1882946876305338):0.1716342657036236[100],Bacteria_CPR_BerkelbacteriaACD58_GWB1_Berkelbacteria_38_5:0.3960107165379778):0.12736800667635872[100],((Bacteria_Berkelbacteria_RBG_13_Berkelbacteria_40_8:0.27143701799687037,Bacteria_CPR_BerkelbacteriaACD58_GWE1_Berkelbacteria_39_12_complete:0.2895097550634915):0.07917888790729588[99],Ga0172379_10001373:0.31875056965555393):0.10585793695691814[100]):0.17982032494594824[100],((Ga0172379_10032720:0.24235849434021528,Bacteria_CP_Berkelbacteria_ACD58:0.269982091325212):0.26294082889690973[100],Bacteria_Berkelbacteria_RIFCSPHIGHO2_12_FULL_Berkelbacteria_36_9:0.410996398532145):0.10257622609953954[96]):0.07995089545850576[99]):0.039074255163940474[35],Ga0172379_10000484:0.659905045728423):0.04829888690159079[87],(((((((Ga0172379_10004904:0.32071322523779777,Ga0172380_10000187:0.2004071997284549):0.10215894609683707[100],(Ga0172380_10025595:0.2547938009739461,Ga0172380_10021443:0.23851975734914443):0.07403060208996237[99]):0.058372481565687195[99],(Ga0172381_10002933:0.20938015020896605,Ga0172381_10006113:0.18408815167462178):0.1314900713694973[100]):0.051389983294646946[88],Ga0172379_10000011:0.352392694247873):0.09404725816905302[100],(Bacteria_Berkelbacteria_RIFOXYA2_FULL_Berkelbacteria_43_10:0.6076467394591987,Ga0172380_10000050:0.3496452404253172):0.09576122590036862[89]):0.09630407960928933[100],(((Ga0172377_10003053:0.0012455523682604053,Ga0172382_10024508:0.000681):0.07220134369159803[100],Ga0172381_10010933:0.06621708061714404):0.2969883990776361[100],(Ga0172381_10024235:0.2555318573720431,Bacteria_Berkelbacteria_CG_Berkel_01:0.23698368690382932):0.10241504709832805[100]):0.27811515391609554[100]):0.14137247448394863[100],(Bacteria_CPR_BerkelbacteriaACD58_GWA2_Berkelbacteria_38_9:0.780447248270534,Ga0172379_10000850:0.7951702581543674):0.18348668376402566[93]):0.0742777831915662[79]):0.06521802294956336[90]):0.0324674929547748[37],(((((Bacteria_Kazan_RIFCSPLOWO2_01_FULL_KAZAN_48_13:0.08593520406598554,Ga0172380_10004084:0.05502839294717754):0.2508481652549528[100],Bacteria_Kazan_RBG_13_KAZAN_50_9:0.3280360963144249):0.04780595122659248[57],(Ga0172379_10000651:0.21035543126396794,Bacteria_CPR_Kazan_GWA1_Kazan_rel_44_22_partial:0.23234807570262772):0.1653712732383772[100]):0.22047190604031952[100],(Bacteria_CPR_Kazan_GWA1_Kazan_50_15_complete:0.19658153668968747,Bacteria_Kazan_RIFCSPLOWO2_01_FULL_KAZAN_45_19:0.20371227696086613):0.35715273157809513[100]):0.2733509523543356[100],Ga0172380_10000011:0.7311445440632922):0.10850396549099539[71]):0.026846721810349194[23]):0.05110601509205992[88],((((((((((((((((((((((((Ga0172379_10048404:0.05784961773108055,Bacteria_CPR_MicrogenomatesOP11_Woesebacteria_GWA1_OP11_41_7:0.0470233605844923):0.07972409647953649[100],Bacteria_CPR_MicrogenomatesOP11_Woesebacteria_GWD2_OP11_40_19:0.09687117130236311):0.023885115599230566[80],Ga0172379_10020539:0.13393303312167504):0.028678698808855874[100],Ga0172381_10042627:0.18104619409562472):0.051427220922820815[99],((Ga0172379_10000004:0.07393625801773895,Bacteria_CPR_MicrogenomatesOP11_Woesebacteria_GWA1_OP11_44_23:0.08313606411069197):0.0429541981798085[99],Ga0172379_10041896:0.08463474765224221):0.06586916153983724[100]):0.06043697992208541[99],Bacteria_CPR_MicrogenomatesOP11_Woesebacteria_GWA2_OP11_40_7:0.23872403994262603):0.03540604065345132[74],Bacteria_CPR_MicrogenomatesOP11_Woesebacteria_GWB1_OP11_45_5_partial:0.20066326261529932):0.07601146463160058[99],Bacteria_CPR_MicrogenomatesOP11_Woesebacteria_GWB1_OP11_41_10:0.32007845182506456):0.0853045742156473[100],Ga0172379_10000394:0.3252160918911513):0.05184469414976833[88],((((Ga0172379_10000724:0.014330426752602765,Bacteria_Microgenomates_OP11_Woesebacteria_RIFOXYA1_FULL_OP11_Woesebacteria_31_71:0.019078562362648377):0.13086978673728522[100],Ga0172379_10016269:0.23345370697542034):0.048870578429458966[69],Ga0172379_10028887:0.25383856864747356):0.288736613004406[100],Ga0172379_10000074:0.30944137557890317):0.060807379038141374[49]):0.04113585699206945[57],((((Bacteria_CPR_MicrogenomatesOP11_Woesebacteria_GWA2_ACD61_rel_44_33:0.04638675545230564,Bacteria_CPR_MicrogenomatesOP11_Woesebacteria_GWC2_OP11_47_16:0.055576400811347515):0.15541604252844632[100],Bacteria_Microgenomates_OP11_Woesebacteria_RIFCSPHIGHO2_12_FULL_OP11_Woesebacteria_46_16:0.20892888819677502):0.07603266684535104[100],Bacteria_Microgenomates_OP11_Woesebacteria_RBG_13_OP11_Woesebacteria_46_13:0.26558171635739614):0.036705370676089366[62],(((Bacteria_Microgenomates_OP11_Woesebacteria_RBG_16_OP11_Woesebacteria_42_24:0.146444542229478,Bacteria_Microgenomates_OP11_Woesebacteria_RIFCSPHIGHO2_12_FULL_OP11_Woesebacteria_42_9:0.14645946293598922):0.15937173386258952[100],Bacteria_CPR_MicrogenomatesOP11_Woesebacteria_GWA1_OP11_45_8_very_partial:0.32667594996002736):0.08525958375740439[100],Bacteria_Microgenomates_OP11_Woesebacteria_RIFOXYD1_FULL_OP11_Woesebacteria_40_21:0.2769078130770848):0.02536146677780282[21]):0.0360452428218041[78]):0.05966075411572502[72],(((((((Ga0172379_10020919:0.27619886579697406,Bacteria_Microgenomates_OP11_Woesebacteria_RIFCSPHIGHO2_02_FULL_OP11_Woesebacteria_39_13:0.19188330080090354):0.04426871301990021[73],Bacteria_Microgenomates_OP11_Woesebacteria_RIFCSPHIGHO2_01_FULL_OP11_Woesebacteria_38_26b:0.27874992716027736):0.06315853977401398[76],(Bacteria_Microgenomates_OP11_Woesebacteria_RIFCSPHIGHO2_01_FULL_OP11_Woesebacteria_37_10:0.3549154111910835,Bacteria_Microgenomates_OP11_Woesebacteria_RIFCSPLOWO2_01_FULL_OP11_Woesebacteria_39_10b:0.3435944034248335):0.03222121528166033[34]):0.02607833159583617[47],((Bacteria_Microgenomates_OP11_Woesebacteria_RIFCSPLOWO2_01_FULL_OP11_Woesebacteria_39_10:0.2548807847694756,Bacteria_Microgenomates_OP11_Woesebacteria_RIFCSPLOWO2_01_FULL_OP11_Woesebacteria_39_25:0.22751508828923317):0.044211455148984946[64],(Bacteria_Microgenomates_OP11_Woesebacteria_RIFCSPLOWO2_01_FULL_OP11_Woesebacteria_39_14:0.2317035899860147,Bacteria_Microgenomates_OP11_Woesebacteria_RBG_13_OP11_Woesebacteria_34_9:0.3383256869512228):0.028237225312358838[30]):0.07092509130574465[99]):0.021233425684812346[34],Bacteria_Microgenomates_OP11_Woesebacteria_GWA1_OP11_Woesebacteria_41_8:0.3529430872113881):0.0366888606927378[37],((Bacteria_Microgenomates_OP11_Woesebacteria_GWB1_OP11_Woesebacteria_43_5:0.42005245263293656,Bacteria_Microgenomates_OP11_Woesebacteria_RBG_16_OP11_Woesebacteria_39_8b:0.34965713939366827):0.03302333242702016[44],Bacteria_Microgenomates_OP11_Woesebacteria_RBG_16_OP11_Woesebacteria_34_12:0.3399990454329389):0.06542903112037379[64]):0.025082970231077972[44],(((Bacteria_Microgenomates_OP11_Woesebacteria_RIFOXYA1_FULL_OP11_Woesebacteria_43_9:0.18401991525525885,Ga0172379_10023463:0.11089628087501646):0.5607254971852038[100],Bacteria_Microgenomates_OP11_Woesebacteria_RBG_16_OP11_Woesebacteria_36_11:0.28704989758299826):0.08877460531819548[72],(Bacteria_Microgenomates_OP11_Woesebacteria_RIFCSPHIGHO2_01_FULL_OP11_Woesebacteria_40_22:0.3082874124410542,Ga0172379_10008721:0.40180108790610314):0.10888185889838864[99]):0.033391755221746866[35]):0.03493346955093157[62]):0.05061457332701247[97],((((Bacteria_Microgenomates_OP11_Woesebacteria_RIFCSPLOWO2_01_FULL_OP11_Woesebacteria_44_24b:0.31793788771732157,Bacteria_Microgenomates_OP11_Woesebacteria_RIFCSPHIGHO2_01_FULL_OP11_Woesebacteria_41_10:0.34391965229268395):0.09946733866371149[100],Bacteria_CPR_MicrogenomatesOP11_Woesebacteria_GWB1_OP11_43_14:0.3768203388089306):0.0398496881273922[63],Bacteria_Microgenomates_OP11_Woesebacteria_RIFOXYB1_FULL_OP11_Woesebacteria_38_16:0.4411270543394106):0.05249818439827392[94],(Bacteria_Microgenomates_OP11_Woesebacteria_RIFCSPLOWO2_01_FULL_OP11_Woesebacteria_44_14:0.34389064389732393,Bacteria_CPR_MicrogenomatesOP11_Woesebacteria_GWB1_OP11_44_11_partial:0.31343184933048907):0.06521473347188467[85]):0.07643859933350328[100]):0.045483713225148215[61],((Bacteria_Microgenomates_OP11_RIF_OP11_2_RIFCSPLOWO2_01_FULL_RIF_OP11_02_39_21:0.22424103416625885,Bacteria_Microgenomates_OP11_RIF_OP11_2_RBG_16_RIF_OP11_02_41_13:0.2034916633661732):0.2203547203584213[100],Bacteria_Microgenomates_OP11_RIF_OP11_2_RIFCSPLOWO2_01_FULL_RIF_OP11_02_38_20:0.4272600121141332):0.0646944260236455[88]):0.19244251107462862[100],((Bacteria_Microgenomates_OP11_RIF_OP11_1_RIFCSPHIGHO2_01_FULL_RIF_OP11_01_43_15b:0.2709203196208363,Bacteria_Microgenomates_OP11_RIF_OP11_1_RIFCSPHIGHO2_12_FULL_RIF_OP11_01_44_25:0.2521504323712356):0.2007035285101657[100],(Bacteria_Microgenomates_OP11_RIF_OP11_1_RIFCSPLOWO2_01_FULL_RIF_OP11_01_40_20:0.32426231772415326,Bacteria_Microgenomates_OP11_RIF_OP11_1_RIFCSPHIGHO2_12_FULL_RIF_OP11_01_41_13b:0.3241726594526866):0.10084651982970527[100]):0.1580962522208047[100]):0.10254102187636116[100],(((((((((Ga0172379_10000352:0.24300183772328987,Ga0172379_10015582:0.1850828343886608):0.10560225391872624[100],Bacteria_Microgenomates_OP11_Amesbacteria_RIFOXYB1_FULL_OP11_Amesbacteria_44_23:0.2839882880303697):0.06457670481162436[98],Ga0172379_10015640:0.36264833730422064):0.1012430664146895[100],((Bacteria_Microgenomates_OP11_Amesbacteria_RIFCSPHIGHO2_12_FULL_OP11_Amesbacteria_48_14:0.2412048939376339,Bacteria_CPR_MicrogenomatesOP11_Amesbacteria_GWB1_OP11_47_19:0.2316676708031271):0.08879571537240771[100],Bacteria_CPR_MicrogenomatesOP11_Amesbacteria_GWA1_OP11_47_16:0.2919818319257903):0.04846941376022729[93]):0.029957569098078007[62],Ga0172379_10019678:0.31773377200440267):0.036117083766430635[83],(Bacteria_Microgenomates_OP11_Amesbacteria_RIFCSPHIGHO2_01_FULL_OP11_Amesbacteria_48_32b:0.31270923880323,Bacteria_Microgenomates_OP11_Amesbacteria_RIFCSPHIGHO2_01_FULL_OP11_Amesbacteria_48_32:0.32923344147401234):0.0748831206633338[100]):0.03908070135251984[61],((Bacteria_CPR_MicrogenomatesOP11_Amesbacteria_GWB1_OP11_47_26:0.1171805498590266,Bacteria_CPR_MicrogenomatesOP11_Amesbacteria_GWC2_OP11_47_8:0.12307124005499892):0.08622911997809046[100],Bacteria_CPR_MicrogenomatesOP11_Woesebacteria_GWA1_OP11_41_13b:0.2009831824316941):0.17726716081167826[100]):0.04423856390586067[52],(Bacteria_CPR_MicrogenomatesOP11_Amesbacteria_GWA2_OP11_42_12:0.35569503434204464,Bacteria_CPR_MicrogenomatesOP11_Amesbacteria_Microgenomates_bacterium_SCGC_AAA011_B20_DUSEL_001_197:0.34300309862810563):0.08974195317297129[98]):0.17241736340527591[100],Bacteria_CPR_MicrogenomatesOP11_GWA1_OP11_45_10_partial:0.620657719628058):0.07900720912922798[100]):0.05501887955746021[88],(((((((((((Ga0172379_10000191:0.03180258945254488,Ga0172379_10000713:0.025490923664167386):0.4466736770988633[100],Ga0172379_10000162:0.2447181590137255):0.09009678351997108[100],Bacteria_Microgenomates_OP11_Shapirobacteria_RBG_13_OP11_Shapirobacteria_44_7:0.28350499716898403):0.04770174553993156[75],((Ga0172379_10021858:0.20579467185212552,Ga0172381_10005105:0.17911669566989064):0.0465664847302234[24],Ga0172379_10000045:0.2474135361011629):0.05749985561984383[91]):0.0299994568616766[17],((((Bacteria_Microgenomates_OP11_Shapirobacteria_CG_Saphiro_01:0.04882793239721117,Ga0172379_10039814:0.0762672263475821):0.017256695796146193[60],(Ga0172379_10035323:0.06183918043351255,Ga0172380_10000001:0.035671718907314354):0.013342897330456793[29]):0.16065872576910856[100],Bacteria_CPR_MicrogenomatesOP11_GWA2_OP11_40_6:0.24114896037160882):0.06178576829915716[100],Bacteria_CPR_MicrogenomatesOP11_Shapirobacteria_GWF2_OP11_rel_37_20:0.2162037934554033):0.11320192866017642[100]):0.027670756319009282[28],Ga0172379_10029326:0.24729716828402415):0.021261978887986643[30],((((Ga0172378_10000023:0.07489574642213714,Ga0172379_10001133:0.05480374332780924):0.03505472400437881[100],Ga0172381_10052990:0.07150331798057463):0.024510724223642644[96],Ga0172381_10030958:0.052279432054336716):0.07289224349596513[100],(Ga0172382_10022888:0.14946776460567612,Ga0172381_10007382:0.12090910597542592):0.05377548669703147[100]):0.102726646548152[100]):0.06655274315633442[81],(Bacteria_Microgenomates_OP11_Shapirobacteria_RIFOXYA1_FULL_OP11_Shapirobacteria_39_17:0.0069624460367117,Ga0172380_10007250:0.011017186236781384):0.23596634234525027[100]):0.07104885471499722[88],Ga0172381_10049003:0.3104564491165238):0.30324242899946485[100],Ga0172381_10007292:0.6065484998745063):0.10855815266371671[98],Bacteria_CPR_MicrogenomatesOP11_Shapirobacteria_Microgenomates_bacterium_SCGC_AAA255_J07_SAK_001_132:0.584484341612109):0.13716062293148656[100]):0.035465468140686074[62],(((((((((((((((((Bacteria_CPR_MicrogenomatesOP11_Collierbacteria_GWA2_OP11_ACD61_44_13:0.033964079266711966,Ga0172379_10037604:0.0238778574114491):0.011131637395941585[83],(Bacteria_Microgenomates_OP11_Collierbacteria_CG_Collier_01:0.051445971734656926,Bacteria_Microgenomates_OP11_Collierbacteria_RIFOXYC2_FULL_OP11_Collierbacteria_43_15:0.03117558541699239):0.014440079427997077[81]):0.025456872595496893[99],Ga0172379_10028166:0.05719171088496955):0.05090237954763843[100],Bacteria_CPR_MicrogenomatesOP11_Collierbacteria_GWA2_OP11_44_99:0.07127410698509662):0.024839479968425504[75],Bacteria_CPR_MicrogenomatesOP11_Collierbacteria_GWB1_OP11_44_6:0.08722715301765716):0.019409537690662226[52],Ga0172379_10006326:0.10294826746469399):0.07655794889486645[100],Bacteria_Microgenomates_OP11_Collierbacteria_RIFOXYD1_FULL_OP11_Collierbacteria_40_9:0.19035226530568128):0.03809012692880964[96],Bacteria_CPR_MicrogenomatesOP11_Collierbacteria_GWA2_OP11_42_17:0.15527530516358423):0.062236367522852554[100],((Ga0172379_10004777:0.06547247773814346,Bacteria_CPR_MicrogenomatesOP11_Collierbacteria_GWE1_OP11_ACD61_46_18:0.047492687179636395):0.04333204520209444[100],Ga0172379_10013612:0.09662557763897528):0.1319158000444327[100]):0.18793805996956703[100],(Bacteria_CPR_MicrogenomatesOP11_Collierbacteria_Microgenomates_bacterium_SCGC_AAA011_I21_DUSEL_001_204:0.15045898603958108,Bacteria_Microgenomates_OP11_Collierbacteria_RIFCSPHIGHO2_01_FULL_OP11_Collierbacteria_50_25:0.1612794566102842):0.21004999569918015[100]):0.20653191624184952[100],Bacteria_Microgenomates_OP11_Collierbacteria_RIFOXYB1_FULL_OP11_Collierbacteria_49_13:0.45430795880698804):0.18251351947602146[100],((((Ga0172380_10003230:0.0740601175052853,Ga0172379_10005585:0.06808512232977249):0.12246439048317015[100],(Bacteria_CPR_MicrogenomatesOP11_GWA2_OP11_46_16_partial:0.05918524574085504,Bacteria_CPR_MicrogenomatesOP11_GWA2_OP11_46_7:0.05499802097768214):0.14189365905441775[100]):0.10467302508858012[100],(Ga0172379_10000851:0.0026877115074888103,Bacteria_CPR_MicrogenomatesOP11_GWF1_OP11_46_12:0.000001):0.26381403921439794[100]):0.09801938325427262[100],Bacteria_CPR_MicrogenomatesOP11_GWF2_OP11_47_9:0.2767865876492914):0.27642756025751014[100]):0.0751637753395693[99],((((((Bacteria_CPR_MicrogenomatesOP11_Pacebacteria_GWB1_OP11_rel_47_8:0.18611310110342627,Bacteria_Microgenomates_OP11_Pacebacteria_RIFOXYA1_FULL_OP11_Pacebacteria_38_18:0.1841253750353804):0.04898808657984555[96],Bacteria_Microgenomates_OP11_Pacebacteria_CG_Pacebac_02:0.19834548158542686):0.10717851533234324[100],((Ga0172380_10010814:0.21843232371183907,Ga0172382_10002483:0.19484305422789516):0.20001109143863882[100],Ga0172377_10000622:0.35351038909987853):0.06482057702094979[99]):0.05664543225719498[98],Ga0172379_10000262:0.3425967359789044):0.03211729317881895[41],(Bacteria_Microgenomates_OP11_Pacebacteria_RIFCSPHIGHO2_02_FULL_OP11_Pacebacteria_46_9:0.4284835526044395,Bacteria_Microgenomates_OP11_Pacebacteria_CG_Pacebac_01:0.2874075123560922):0.07676182555349476[99]):0.17688405931347528[100],((Ga0172379_10024579:0.2324697258666144,Bacteria_CPR_MicrogenomatesOP11_GWF1_OP11_44_10:0.25548917662655324):0.07732130576373741[99],Bacteria_CPR_MicrogenomatesOP11_GWB1_OP11_45_17:0.280075994845403):0.20442509931389186[100]):0.1083818575338058[100]):0.04787572771538828[58],((Bacteria_Microgenomates_OP11_RIFCSPHIGHO2_01_FULL_OP11_45_11:0.4819108638981171,Bacteria_Microgenomates_OP11_RIFCSPLOWO2_01_FULL_OP11_47_10:0.43530338237412636):0.076669510215996[95],Bacteria_Microgenomates_OP11_RBG_16_OP11_45_19:0.5405684431678996):0.08268244282749881[95]):0.039563868476224506[59],((Bacteria_Microgenomates_OP11_RIF_OP11_3_RIFCSPLOWO2_01_FULL_RIF_OP11_03_50_28:0.3334621319839557,Bacteria_Microgenomates_OP11_RIF_OP11_3_RIFCSPLOWO2_01_FULL_RIF_OP11_03_49_14:0.29767391415089106):0.13964987068674883[100],Bacteria_Microgenomates_OP11_RIF_OP11_3_RIFCSPHIGHO2_01_FULL_RIF_OP11_03_48_12:0.5580398553074919):0.07737242519539[92]):0.047727211471391584[95],(((((Bacteria_CPR_MicrogenomatesOP11_Beckwithbacteria_GWA2_OP11_43_10:0.07240791912529154,Bacteria_Microgenomates_OP11_Beckwithbacteria_CG_Beckwith_02:0.09689376154467277):0.22396276871530585[100],(Bacteria_Microgenomates_OP11_Beckwithbacteria_RIFCSPLOWO2_02_FULL_OP11_Beckwithbacteria_47_23:0.12756105328044232,Bacteria_Microgenomates_OP11_Beckwithbacteria_CG_Beckwith_01:0.159347432903584):0.20770564621283283[100]):0.08652600878357974[100],Bacteria_CPR_MicrogenomatesOP11_Beckwithbacteria_GWC1_OP11_49_16_complete:0.30215209703465984):0.20853351935557285[100],Bacteria_Microgenomates_OP11_Beckwithbacteria_RBG_13_OP11_Beckwithbacteria_35_6:0.44677279513174106):0.03530888908830265[44],Bacteria_Microgenomates_OP11_Beckwithbacteria_RBG_13_OP11_Beckwithbacteria_42_9:0.5000407347650504):0.05128806246674422[83]):0.052488709976231895[87],(Bacteria_Microgenomates_OP11_RBG_19FT_COMBO_OP11_39_10:0.13393084001912214,Bacteria_OP11_uncultured_DG_75:0.12452367760738436):0.32957813952345116[100]):0.0375134704508846[66]):0.08379084075484267[100],((((((((((((((((Ga0172379_10003545:0.028181108784114173,Ga0172379_10003078:0.030598954598216377):0.03734047231065851[100],Bacteria_CPR_MicrogenomatesOP11_Roizmanbacteria_GWC2_OP11_ACD12_rel_34_23:0.0699668667204727):0.014538634884410762[55],Ga0172379_10005655:0.06539737864631734):0.042719740634218795[100],(Bacteria_CPR_MicrogenomatesOP11_Roizmanbacteria_Microgenomates_bacterium_SCGC_AAA011_L05_DUSEL_001_205:0.060737299458599736,Bacteria_CPR_MicrogenomatesOP11_Roizmanbacteria_OP11_GWA2_32_13:0.0683154708532916):0.039303069660069134[93]):0.2059042687279602[100],(Bacteria_CPR_MicrogenomatesOP11_Roizmanbacteria_GWC2_OP11P
[truncated: 476,250 more chars]
